# Proteomic Response Revealed Signaling Pathways Involving in the Mechanism of Polymyxin B-Induced Melanogenesis

**DOI:** 10.1128/spectrum.02730-21

**Published:** 2022-04-04

**Authors:** Chuhan Zhang, Xiaofen Liu, Hailan Wu, Yu Wang, Yaxin Fan, Beining Guo, Xingchen Bian, Xin Li, Jing Zhang

**Affiliations:** a Institute of Antibiotics, Huashan Hospital, Fudan University, Shanghai, China; b Key Laboratory of Clinical Pharmacology of Antibiotics, National Health Commission of the People’s Republic of China, Shanghai, China; c National Clinical Research Centre for Aging and Medicine, Huashan Hospital, Fudan University, Shanghai, China; d Phase I Clinical Trial Center, Huashan Hospital, Fudan University, Shanghai, China; University of Guelph

**Keywords:** hyperpigmentation, melanogenesis, polymyxin B, signaling pathway

## Abstract

Polymyxin B is a last-line antibiotic for extensively resistant Gram-negative bacterial infection. Skin hyperpigmentation is a serious side effect induced by polymyxin B that severely compromises the psychological health and compliance of patients. The literature lacks mechanistic studies that explain how hyperpigmentation occurs, and this substantially hinders the development of intervention strategies and improved compliance. SK-MEL-2 cells were used for the polymyxin B-induced hyperpigmentation mechanism study. Melanin content and tyrosinase activity were measured after polymyxin B treatment. Tandem mass tag (TMT)-labeling quantitative proteomics was employed to investigate the response of SK-MEL-2 cells to polymyxin B treatment. Real-time quantitative PCR and Western blot were applied to validate the mRNA and protein levels of related genes and proteins. The melanin content and tyrosinase activity were significantly upregulated after polymyxin B treatment in SK-MEL-2 cells at 48 h and 72 h. Quantitative proteomics showed that 237 proteins were upregulated and 153 proteins were downregulated in the 48 h group, and 49 proteins were upregulated and 49 proteins were downregulated in the 72 h group. The differentially expressed proteins were involved in pathways such as lysosome, PI3K/Akt signaling pathway, and calcium signaling pathway. The upregulation of melanogenic enzymes and microphthalmia-associated transcription factor (MITF) was validated by qPCR and Western blot. Meanwhile, phosphorylation of PI3K, β-catenin, and cyclic-AMP response binding protein (CREB) in response to polymyxin B treatment was observed. The present study reveals the proteomic response of polymyxin B-induced melanogenesis in SK-MEL-2 cells for the first time. Signaling pathways, including melanin biosynthesis, PI3K/Akt, and calcium signaling pathways may be involved in the mechanism of melanogenesis.

**IMPORTANCE** Polymyxin B-induced skin hyperpigmentation seriously affects the psychological health and compliance of patients. This study provides a proteomic clue to the mechanism at the cellular level for understanding polymyxin B-induced hyperpigmentation, contributing to a follow-up investigation of the corresponding PI3K/Akt signaling transduction pathway and calcium signaling pathway. The elucidation of its underlying mechanism is of great significance for patients' compliance improvement, intervention strategy, and new drug development.

## INTRODUCTION

Polymyxin B (PMB), a polypeptide antibiotic, is considered a last-line effective antibiotic for the treatment of infections caused by extensively resistant Gram-negative bacteria (1). Skin hyperpigmentation was reported related to polymyxin B treatment as it applied in clinics (2–6). Two cohort studies have shown that the incidence of polymyxin B-induced skin hyperpigmentation (PMB-iSH) was 8 to 15% (7, 8). During the outbreak of the coronavirus disease 2019 (COVID-19) in 2020, two frontline doctors in Wuhan, China, had been diagnosed with COVID-19 and suffered from secondary infections caused by multidrug-resistant bacteria. Polymyxin B was applied to treat infections. However, hyperpigmentation on the head and neck and the prognosis, as well as pathogenesis, has raised public awareness (9). Patients who suffered from hyperpigmentation, showed a diffused darkening after intravenous polymyxin B treatment, particularly on the head and neck. Despite the hyperpigmentation associated with no pain or pruritus (4), it seriously affected patients’ compliance and psychological health. So far, no effective precaution or intervention strategy has been found in clinics.

To date, the mechanism of PMB-iSH is not well understood. Skin darkening usually occurs on the 3^rd^ to 7^th^ day after commencing intravenous polymyxin B treatment, with no significant difference in light exposure and sites of infections among patients (7). Excessive cumulation of polymyxin B may be an important factor for aberrant pigmentation because neonates and infants with immature kidney function were more likely to suffer (6). Skin histopathology showed an abundant melanocyte dendritic network, Langerhans cells’ hyperplasia, and dermal interleukin-6 (IL-6) overexpression, suggesting that PMB-iSH may be associated with an inflammatory process and subsequent melanocyte activation (8). It has been speculated that polymyxin B stimulates the release of histamine by mast cells (10) and then the released histamine recognizes H_2_ receptors on melanocytes and induces melanogenesis through the protein kinase A pathway (11). However, this particular hypothesis may be controversial. First, histamine-induced hyperpigmentation is generally localized rather than diffused across the head and neck (12, 13). Second, polymyxin E (also known as colistin, the active component of colistimethate), which is the other polypeptide antibiotic with structure and antimicrobial activity similar to polymyxin B, can also release histamine equivalent to polymyxin B (10). However, skin hyperpigmentation induced by polymyxin E has not yet been reported. Therefore, it is necessary to further investigate the mechanism of polymyxin B-induced melanogenesis.

SK-MEL-2 cell line belongs to a series of melanoma cell lines established from patient-derived tumor samples, which is usually applied in melanin-related mechanism studies (14–16). TMT-labeling quantitative proteomics, which is the state-of-art LC-MS/MS technique for large-scale protein quantitation, has been widely applied in different fields to reveal the underlying mechanisms (17–20). In the present study, TMT-labeling quantitative proteomics has been applied to reveal the overall response of proteins involved in the polymyxin B-induced melanogenesis in SK-MEL-2 cells. The signaling pathways were investigated to uncover the mechanism of PMB-iSH.

## RESULTS

### Effects of polymyxin B on tyrosinase activity and melanin synthesis.

**FIG 1:**
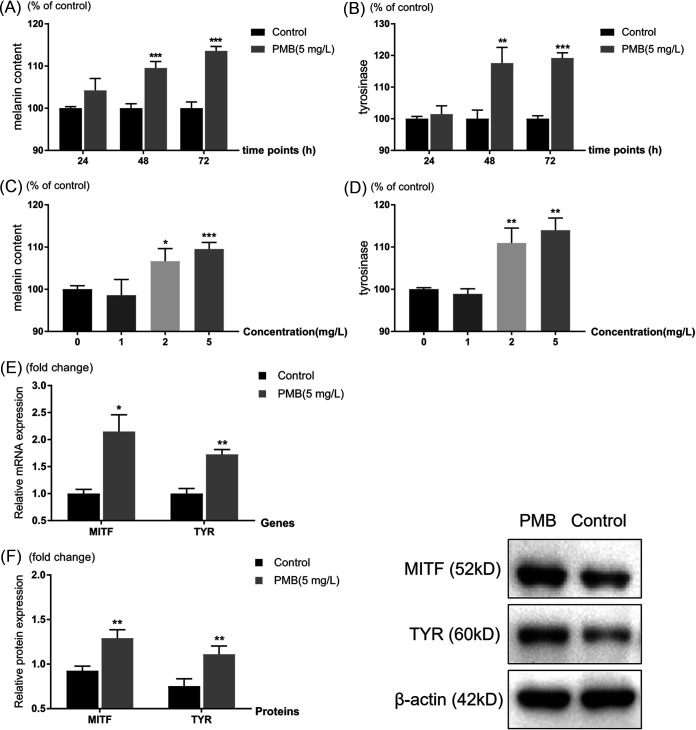
Effects of polymyxin B on the melanogenesis in SK-MEL-2 cells. Melanin content (A) and tyrosinase activity (B) measured in SK-MEL-2 cells after 5 mg/liter polymyxin B treatment were compared with control for various durations (24 to 72 h). Melanin content (C) and tyrosinase activity (D) determined in SK-MEL-2 cells exposed to the indicated concentrations (0 to 5 mg/liter) of polymyxin B were compared with control at 48 h. Data were normalized as a percentage of the control group. The mRNA (E) and protein (F) levels of MITF and TYR expressed for 5 mg/liter polymyxin B-treated cells were compared with control at 48 h. Data were expressed as fold changes of treatment to the control group. *, *P* < 0.05; **, *P* < 0.01; ***, *P* < 0.001, compared to the control. Three replicates were repeated for all experiments. MITF, microphthalmia-associated transcription factor; TYR, tyrosinase.

A higher concentration of polymyxin B (10 mg/liter) showed cellular toxicity to SK-MEL-2 cells by CCK-8 testing (Fig. S1), thus 1 to 5 mg/liter were served as experimental concentrations. Both melanin content and tyrosinase activity revealed increased levels in the 5 mg/liter polymyxin B-treated group compared with the control (PBS treatment) group at 48 and 72 h significantly (*P* < 0.01) and the ratios were increasing as treatment duration increased from 24 to 72 h, indicating a time-dependent manner (Fig. 1A and B). The melanin content and tyrosinase activity were upregulated as concentrations of polymyxin B increased from 1 to 5 mg/liter (*P* < 0.01 for 2 and 5 mg/liter polymyxin B treatment) in a concentration-dependent manner (Fig. 1C and D) in SK-MEL-2 cells. Noticed that 5 mg/liter of polymyxin B treatment showed a more significant increase in melanogenic level with improved cell activity, we use 5 mg/liter as an effective concentration of polymyxin B in the subsequent experiments.

Additionally, the mRNA and protein levels of tyrosinase (TYR) were validated upregulated in the polymyxin B treatment group compared to the control group (Fig. 1E and F) at 48 h. Furthermore, the mRNA and protein levels of the upstream regulator gene of MITF were dramatically upregulated upon polymyxin B treatment (Fig. 1E and F).

### Polymyxin B-induced proteome perturbations in SK-MEL-2 cells.

A total of 451,178 spectrums were acquired and 166,684 spectrums were matched, with an identification rate of 36.9%. The matched spectrums were identified as 89,158 peptides and 8,072 proteins, of which 7,271 proteins were quantifiable. Both principal component analysis (PCA) and clustering analysis (Fig. S2) showed good reproducibility among triplicates in different groups. Compared with untreated controls, 237 proteins were upregulated and 153 proteins were downregulated in the 48 h group, and 49 proteins were upregulated and 49 proteins were downregulated in the 72 h group (Fig. S3A). The volcano plot of the differentially expressed proteins is shown in Fig. S3B. The differentially expressed proteins are listed in Table S1.

**FIG 2:**
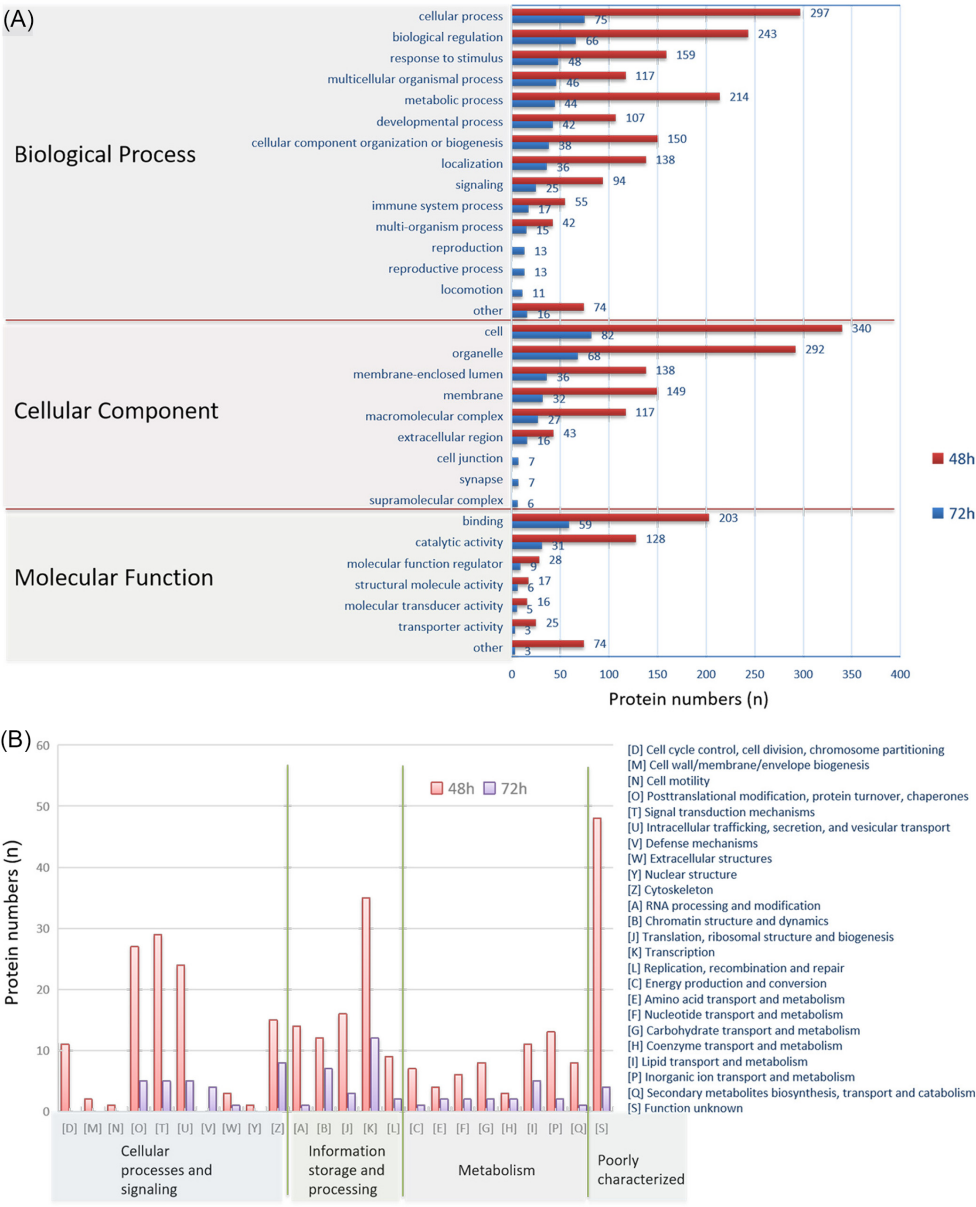
GO and COG analysis for the differentially expressed proteins. (A) The distributions of differentially expressed proteins in GO secondary annotations are divided into 3 categories: biological process, cellular component, and molecular function. (B) COG functional classification statistics for differentially expressed proteins are performed through database comparison and analysis.

Although larger numbers of proteins were differentially expressed in the 48 h group than in the 72 h group, gene ontology (GO) analysis classifications (Fig. 2A) showed these proteins were only in slightly different patterns from the aspect of biological process in both treatment groups. The differentially expressed proteins in 48 h group were mainly involved in cellular processes, biological regulation, metabolic process, cellular component organization, or biogenesis. However, unlike the 48 h group, the differentially expressed proteins in the 72 h group were mostly identified involved in the cellular process, biological regulation, response to stimulus, and multicellular organism process in the aspect of biological process. The differentially expressed proteins in both treatment groups showed similar patterns in cellular component and molecular function, and they were mostly involved in the organelle, membrane, and membrane-enclosed lumen from the aspect of cellular component, and binding, catalytic activity from the aspect of molecular function. Clusters of orthologous groups of proteins (COG) analysis (Fig. 2B) showed proteins identified in transcription, signal transduction mechanism and posttranslational modification, protein turnover, and chaperones were top three functional groups in the differentially expressed proteins in 48 h group while transcription, cytoskeleton, and chromatin structure and dynamics were top three functional groups in the differentially expressed proteins in 72 h group. Both GO and COG analyses showed that the majority of differentially expressed proteins were in similar functional groups for both treatment groups.

**FIG 3:**
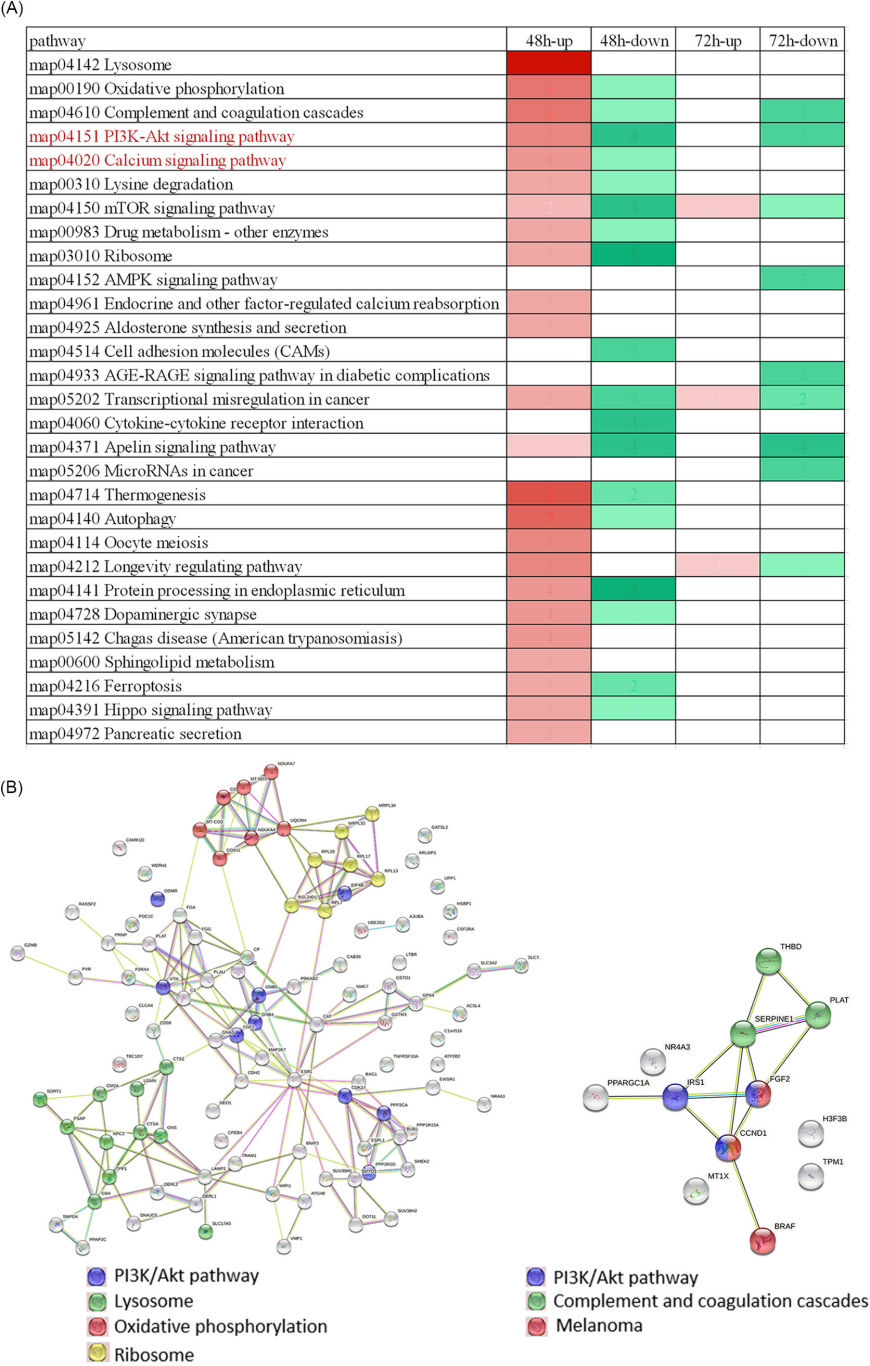
Cluster analysis heat map based on KEGG pathway and protein interaction network. For KEGG pathway analysis, areas in red represent differentially upregulated pathways, areas in green represent differentially downregulated pathways (above). The interaction relationship of the differential proteins at 48 h group (left, below) and 72 h group (right, below).

KEGG pathway analysis of the differentially expressed proteins in both treatment groups showed pathways in response to polymyxin B treatment (Fig. 3). Several pathways were changed in both treatment groups, such as complement and coagulation cascades pathway, PI3K/Akt signaling pathway, mTOR signaling pathway, indicating these pathways were actively responding to polymyxin B during 48 h to 72 h. Certain pathways were changed only in the 48 h group, such as lysosome, oxidative phosphorylation, and calcium signaling pathway, while pathways such as AMPK pathway and microRNAs in cancer were only changed in the 72 h group. These unique pathways in the 48 h group or 72 h group indicated the time-dependent response of SK-MEL-2 cells to polymyxin B. The protein-protein interaction analysis showed complex interaction maps and the pathway enrichment also showed PI3K/Akt signaling pathway, lysosome, oxidative phosphorylation, and ribosome were the most interacted proteins in 48 h group, while PI3K/Akt signaling pathway, complement, and coagulation cascades and melanoma pathways were in 72 h group.

### Polymyxin B induces melanogenesis by activating signaling pathways.

**FIG 4:**
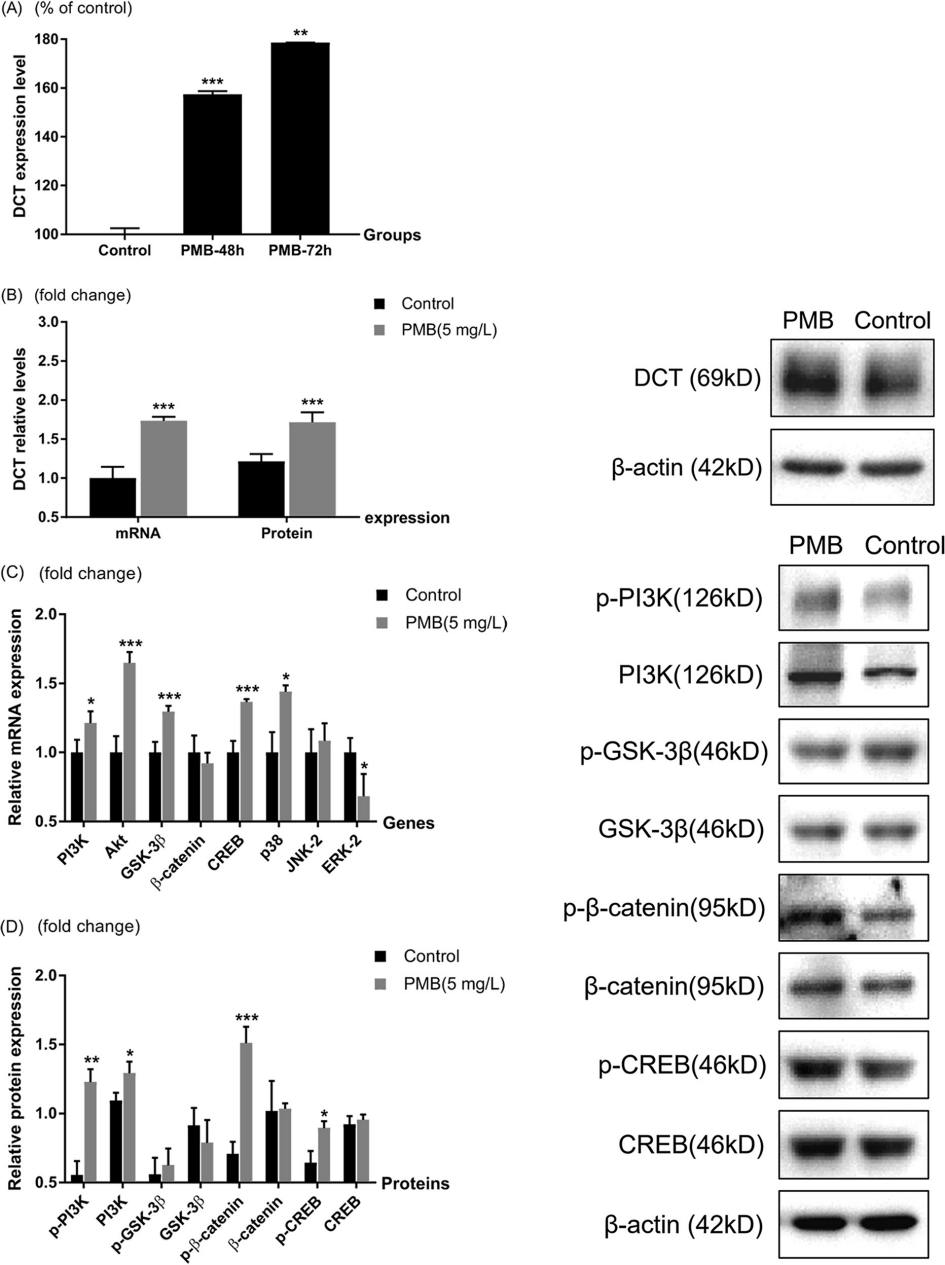
Differential expression of DCT in both 48 h and 72 h groups (proteomic analysis results, [A]) were validated in SK-MEL-2 cells for polymyxin B treatment (B). Potential signaling pathways involved in PMB-induced melanogenesis in SK-MEL-2 cells were explored via the mRNA (C) and protein (D) levels of PI3K, AKT, GSK-3β, CREB, β-catenin, p38, ERK-2, and JNK-2 for 5 mg/liter polymyxin B treated cells compared with control. Data were expressed as fold changes over the control group. *, *P* < 0.05; **, *P* < 0.01; ***, *P* < 0.001, compared to control. Three replicates were repeated for all experiments. PI3K, phosphatidylinositol 3-kinase; AKT, RAC-alpha serine/threonine-protein kinase; GSK-3β, glycogen synthase kinase-3β; CREB, cyclic AMP-responsive element-binding protein; ERK-2, extracellular regulated protein kinase 2; JNK-2, c-Jun N-terminal kinase 2.

Melanogenic protein l-dopachrome tautomerase (DCT), which is a key protein in melanogenesis, was upregulated in both the 48 h and 72 h group in the proteomic analysis (Fig. 4A). Both mRNA and protein levels of DCT validated significant upregulation in polymyxin B treatment in 48 h (Fig. 4B).

**FIG 5:**
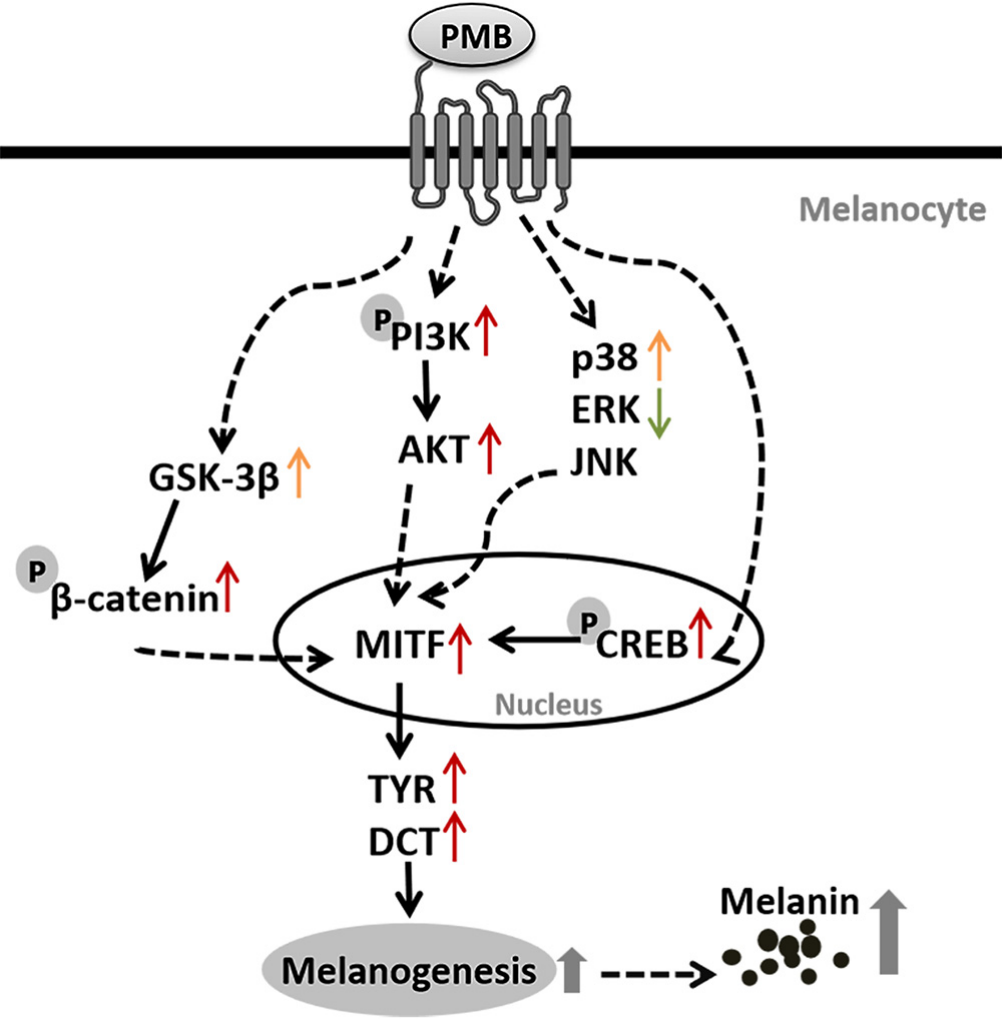
The schematic proposed diagram of the mechanism of polymyxin B-induced melanogenesis pathway in melanocytes. Dashed arrows represent the intermediate path hidden or unknown may exist, and “p” in a circle indicates protein phosphorylation. The red up arrows indicate the upregulation of corresponding proteins. Arrows in orange represent upregulation, only on the transcriptional level, and the green down arrows indicate the downregulated gene transcription. PMB, polymyxin B; PI3K, phosphatidylinositol 3-kinase; AKT, RAC-alpha serine/threonine-protein kinase; GSK-3β, glycogen synthase kinase-3β; CREB, cyclic AMP-responsive element-binding protein; MITF, microphthalmia-associated transcription factor; TYR, tyrosinase; DCT, tyrosinase-related protein-2; ERK-2, extracellular regulated protein kinase 2; JNK-2, c-Jun N-terminal kinase 2.

To validate the signaling pathways of polymyxin B-induced melanogenesis, we measured the mRNA levels of genes in the PI3K/Akt signaling pathway, which was shown to be differentially regulated in proteomic pathway analysis. Several well-studied melanogenesis-related pathways of MAPK family (including p38, ERK, and JNK signaling pathway) and GSK-3β/β-catenin pathway and an important regulated protein CREB were investigated. Cells treated with 5 mg/liter polymyxin B at 48 h showed a remarkable increase in mRNA levels of PI3K, Akt, GSK-3β, CREB, and p38. In contrast, the ERK-2 mRNA level was significantly decreased (Fig. 4C). Western blot analysis showed the ratios of phosphorylated protein to total protein levels of PI3K, β-catenin and CREB were all significantly upregulated in polymyxin B-treated cells relative to untreated cells, while the total protein levels remained unchanged except PI3K. No significant change in GSK-3β or phosphorylated GSK-3β level following the polymyxin B treatment was observed (Fig. 4D). These results indicated that hyperpigmentation induced by polymyxin B was associated with upregulation in signaling pathways involved in phosphorylation of PI3K, β-catenin, and CREB (Fig. 5).

## DISCUSSION

Generally, skin pigmentation, attributed to melanin produced by melanocytes, is regarded as protection of the skin against UV from sunlight (21). However, skin hyperpigmentation induced by polymyxin B, affecting the appearance of children, adults, or generic patients, seriously took a toll on the psychological health and the life quality of patients (3–6). The lack of ideal interventions and the uncertainty of whether skin color will reverse to the baseline naturally, let alone any inconvenience caused during the long recovery period, can bring on severe mental stress and treatment refusal. The pathological mechanism is not yet clearly elucidated, and further investigation is required, which benefits the intervention strategy, new drug development, and improving compliance.

Skin biopsy specimens taken from hyperpigmented skin of patients treated by intravenous polymyxin B showed hyper-melanosis in the basal layer (8, 22), and histology and immunohistochemistry revealed abundant melanocyte-pigmented dendritic network (8, 21). This aberrant increase in skin melanin biosynthesis could be the culprit. The pigment is synthesized in melanocytes, which involves several oxidation steps, and tyrosinase (TYR) is a rate-limiting enzyme participating in the initial step of l-tyrosine oxidation and the last step of eumelanin synthesis in melanogenesis (23). Thus, the activity and amount of TYR directly reflect the level of melanin synthesis. The upregulation of both melanin content and TYR activity in response to polymyxin B in SK-MEL-2 cells showed a time and concentration-dependent manner. The mRNA expression and protein levels of TYR were significantly upregulated, indicating the stimulation of melanin synthesis due to polymyxin B.

Besides TYR, the regulation of skin pigmentation involves two other key structurally related enzymes that catalyze the synthesis of melanin within the melanocytes: tyrosinase-related protein-1 (TYP-1) and tyrosinase-related protein-2 (TYP-2/DCT) (24). Quantitative proteomics showed the upregulation of DCT in both 48 h and 72 h groups in polymyxin B treated SK-MEL-2 cells, confirmed by the upregulated levels of mRNA and protein in real-time quantitative PCR and Western blot. The melanogenic genes (TYR, TYP-1, and DCT) are transcriptionally regulated by MITF, which is a central regulator in melanogenesis, regulating the expression of tyrosinase, melanosome structural proteins, and proteins involved in melanosome trafficking (25, 26). CREB is a transcriptional regulator of the MITF gene, capable of binding to specific DNA sequences located in the MITF promoter region (27). The upregulation of phosphorylated CREB and MITF indicates polymyxin B could induce melanogenesis in SK-MEL-2 melanoma cells by activating CREB followed by MITF and its downstream melanogenic genes.

The expression levels of MITF and CREB are regulated by a series of transcriptional factors associated with signaling pathways in various cellular processes and signal transduction pathways (28), including the cyclic AMP/protein kinase A (cAMP/PKA) (29), WNT/β-catenin (30), extracellular signal-regulated kinase (ERK/MAPK) (31, 32) and PI3K/Akt (33) signaling pathways. Quantitative proteomics revealed the differentially expressed proteins involved in both processes of melanin biosynthesis and signaling pathways. The PI3K/Akt pathway, calcium signaling pathway, mTOR signaling pathway, etc. were differentially regulated in the polymyxin B treated SK-MEL-2 cells. Among these pathways, PI3K/Akt signaling pathway extensively exists in a variety of physiological processes and is a critical pathway in melanogenesis, especially active in melanoma cells (34). In this pathway, Akt binds to and activates PI3K on the plasma membrane by phosphorylation, triggering the phosphorylation of various downstream proteins such as mTOR, GSK-3β (25). Phosphorylation levels of proteins such as PI3K, CREB, and β-catenin in the PI3K/Akt pathway showed increased expressions after polymyxin B treatment, indicating the possible signaling transduction role in polymyxin B-induced melanogenesis. Besides PI3K/Akt pathway, other signaling pathways may also involve in the polymyxin B-induced melanogenesis because the mRNA levels of GSK-3β, p38 showed significant upregulation while the mRNA level of ERK-2 was significantly downregulated (Fig. 5). It is implied that the mechanism of polymyxin B-induced melanogenesis could be a complex and multipathway regulated process.

Melanosomes, where melanin pigment is synthesized and stored before they are distributed to surrounding keratinocytes, are subcellular lysosome-like organelles (35–37). The process of melanin biosynthesis involves a series of oxidative and phosphorylation processes. Proteomic results from this study also showed the oxidation phosphorylation status was differentially affected in SK-MEL-2 cells (38). Protein-protein interaction analysis showed lysosome pathway and oxidative phosphorylation pathways were upregulated in the 48 h group, suggesting the activation of melanin synthesis.

In the present study, proteomic analysis and biological analysis were conducted in melanoma cells. However, hyperpigmentation is a complicated process that involves many cell types. Melanin is synthesized in melanocytes in the epidermis and then translocated to neighboring keratinocytes. Close interactions between melanocytes and keratinocytes are served as intrinsic key players that regulate skin pigmentation (39–41). Follow-up studies should place importance on its interactions with other epidermal cells. Furthermore, clinical cohort studies of risk factors, biomarkers, and intervention strategies for PMB-iSH are also in severe need of implementation.

Polymyxin B could directly upregulate melanogenesis in SK-MEL-2 cells through increased tyrosinase activity and melanin content. TMT-labeled quantitative proteomics revealed several signaling pathways such as PI3K/Akt pathway, calcium pathway, lysosome involved in the polymyxin B-induced hyperpigmentation. This study provides a new viewpoint of the mechanism at the cellular level for understanding the hyperpigmentation of polymyxin B in clinics. Further investigations are needed to comprehensively understand the underlying mechanism.

## MATERIALS AND METHODS

### Materials.

Polymyxin B was obtained from USP Reference Standards (lot no. R046V0). Dulbecco's modified eagle medium (DMEM), fetal bovine serum (FBS), penicillin-streptomycin solution, and 0.25% trypsin were purchased from GIBCO Invitrogen (CA, USA). All chemicals and reagents were purchased from MedChemExpress (NJ, USA) at analytical grade. Gene-specific primers were obtained from Sangon Biotech (Shanghai, China). MITF (ab20663), TYR (ab18075), DCT (ab221144), PI3K (ab154598), p-PI3K (ab182651), GSK3β (ab32391), p-GSK3β (ab75814), β-catenin (ab16051), p-β-catenin (ab75777), p-CREB (ab32096), CREB (ab32515) and β-actin (ab8227) primary antibodies and goat anti-rabbit IgG secondary antibodies (ab205718) were purchased from Abcam (Cambridge, MA, USA).

### Cell culture and treatment.

Human melanoma cells, SK-MEL-2, were purchased from iCell Bioscience Inc. (ATCC catalog number HTB-68, RRID:CVCL_0069). The cells were seeded at the density of 2 × 10^5^ cells per well and cultured in 6-well flat-bottomed plates in DMEM and incubated at 37°C and 5% CO_2_ for 24 h. Polymyxin B or PBS (control) was treated to the cells and collected at 24, 48, and 72 h following the commencement of treatments. The collected cells were washed twice by cold PBS and stored in the fridge at −80°C until analysis.

### Melanin content and cellular tyrosinase activity assay.

Melanin content was measured by the hot alkali lysis method modified slightly (42). SK-MEL-2 cells were treated with polymyxin B at different concentrations (1, 2, 5, and 10 mg/liter) for different durations (24, 48, and 72 h). Cell pellets were dissolved by 100 μL of 1 M sodium hydroxide (NaOH) containing 10% DMSO at 80°C for 2 h and then centrifuged for 10 min at 12,000 g. The supernatants were transferred into a 96-well microplate, and the absorbance was measured at 405 nm. The mean ratios of melanin to cell numbers in the control group were normalized to 100% and were compared by the treatment groups.

Cellular tyrosinase activity was measured using the dopa-quinone oxidation method with minor modification (42). Cell pellets were lysed in RIPA lysis and extraction buffer (Thermofisher, Rockford, IL, USA) for 15 min on ice. Afterward, the cell lysate was centrifuged for 10 min at 12,000 × *g*, and the supernatant was transferred into Eppendorf tubes for total protein concentration measurement by BCA protein assay kit (Thermofisher, Rockford, IL, USA). Protein concentrations were adjusted to 2000 mg/liter with lysis buffer, and 70 μL of each sample was transferred into a 96-well plate by subsequently adding 30 μL of l-DOPA solution (0.1%) and reacting at 37°C for 30 min. Tyrosinase activity was measured by the absorbance at 450 nm. The mean absorbance in the control group was normalized to 100% and compared by the treatment groups.

### Real-time quantitative PCR analysis.

Total cellular RNA was extracted using an RNA Extraction kit (TaKaRa, Japan, 9767), and cDNA was reverse transcribed using a PrimeScript RT Master Mix kit (TaKaRa, RR036B). Quantitative PCR (qPCR) was conducted on a CFX96 Real-Time PCR Detection System (Bio-Rad, CA, USA) in a reaction mixture containing TB Green Premix Ex Taq (TaKaRa, RR820B), 1 μg of cDNA, and 10 μM gene-specific primer sets (Sangon Biotech, Table 1). Reactions were performed using the following protocol: DNA polymerase activation at 95°C for 30 s, 40 cycles of denaturation at 95°C for 5 s, and annealing and extension at 60°C for 30 s. The expression levels of mRNA of target genes were normalized to levels of internal control, ACTB mRNA, and calculated using the comparative threshold cycle method.

**TABLE 1:** Gene primers for real-time PCR analysis

Target	Forward (5′–3′)	Reverse (5′–3′)
PI3K	GAGATTGCAAGCAGTGATAGTG	TAATTTTGGCAGTGATTGTGGG
Akt	ATTTCCCTCTTTGGAGGCTGT	CTGCGCCACAGAGAAGTTGT
GSK-3β	CTACTCCAGTGGTGAGAAGAAAGA	CCAACAAGAGGTTCTGCGGT
β-catenin	CTAGGAATGAAGGTGTGGCGA	AGTCCAAGATCAGCAGTCTCATTC
CREB	GAAGCGGAGTGTTGGTAACT	GCATCTCCACTCTGCTGGTT
p38	TCCAGACCATTTCAGTCCATC	CGTCCAACAGACCAATCACA
ERK-2	ACCCACACAAGAGGATTGAAGT	AAAAGCCACAACTACCAGAAAC
JNK-2	AGCTCCACCACCAAAGATCC	TCACTGCTGCACCTAAAGGA
MITF	AGGCATGAACACACATTCACGAG	CAGGATCCATCAAGCCCAAGA
TYR	TTCCATATTGGGACTGGCGG	CGGCTACAGACAATCTGCCAA
DCT	GTTCCTTTCTTCCCTCCAGTG	TTCCTTTATTGTCAGCGTCAGA
β-actin	ATGGGGAAGGTGAAGGTCG	GGGGTCATTGATGGCAACAA

### Western blot.

In brief, cells pellets were lysed in RIPA buffer containing protease inhibitor and phosphatase inhibitor cocktail and centrifuged at 4°C for supernatants. An aliquot of 50 μg of total proteins from each sample was loaded on a 10% SDS-PAGE gel and transferred onto polyvinylidene difluoride (PVDF) membrane (Millipore, USA). After blocking nonspecific sites with protein-free rapid blocking buffer, the PVDF membrane was incubated with primary antibodies against MITF (ab20663, 1:1000), TYR (ab180753, 1:500), DCT (ab221144, 1:1000), PI3K (ab154598, 1:500), p-PI3K (ab182651, 1:500), GSK3β (ab32391, 1:1000), p-GSK3β (ab75814, 1:500), β-catenin (ab16051, 1:2000), p-β-catenin (ab75777, 1:500), p-CREB (ab32096, 1:500), CREB (ab32515, 1:1000) and β-actin (ab8227, 1:1000) overnight at 4°C. The membrane was further incubated with HRP-conjugated secondary antibodies (ab205718) at a 1:5000 dilution for 1 h and was detected by the enhanced chemiluminescence (ECL) reagent (Millipore, USA) and scanned by a chemiluminescence imaging system (Tanon 5200, China). The protein levels were quantified by the band intensities (Image J analysis software, NIH), and normalized against β-actin.

### TMT-labeling proteomics analysis.

**(i) Protein extraction and trypsin digestion.** Cells were harvested at 0 h (before polymyxin B dosing) for the control group and at 48, 72 h for the polymyxin B treatment group after dosing. All the samples were sonicated on ice in lysis buffer containing 8 M urea and a 1% protease inhibitor cocktail. The supernatant was collected by centrifugation at 12,000 g at 4°C for 10 min, and the total protein concentration was determined using a BCA protein quantification kit (Yeasen, China). The proteins in the supernatant were precipitated by adding 20% trichloracetic acid slowly at 4°C for 2 h and washed with precooled acetone three times. The precipitation was dried and reconstituted in 200 mM triethylammonium bicarbonate (TEAB) for digestion. After being reduced by dithiothreitol (DTT) at a final concentration of 5 mM for 30 min at 56°C and alkylated with iodoacetamide (IAA) at a final concentration of 11 mM for 15 min at room temperature in darkness, trypsin was added at a ratio of 1:50 (protease: protein, wt/wt) for digestion overnight at 37°C.

**(ii) TMT labeling and HPLC fractionation.** The digested peptides were desalted with Strata X C18 (Phenomenex) and labeled with TMT reagents according to the manufacturer’s protocol for the TMT kit. Briefly, the reagent was reconstituted in acetonitrile and then mixed with the peptide and incubated at room temperature for 2 h for labeling. The labeled peptide was desalted for HPLC fractionation, performed by high pH reversed-phase HPLC using an Agilent 300Extend C18 column (5 μm in size, 4.6 mm in diameter, 250 mm in length) with gradient elution of 8% to 32% acetonitrile (pH 9). In total, 60 fractions were collected in 60 min, and then were combined into 14 fractions and freeze-dried in a vacuum for LC-MS/MS analysis.

**(iii)** **Liquid **c**hromatography with tandem mass spectrometry (LC-MS/MS) analysis.** Standard TMT-labeling quantitative proteomics techniques were performed on a Q Exactive HF-X mass spectrometry platform (Thermo Fisher Scientific, Bremen, Germany). The mass spectrometer was interfaced to a nano-electrospray ion source coupled to an EASY-NLC 1200 UPLC-system. The peptides fractions were injected and separated by gradient elution. Mobile phase A was 2% acetonitrile in water (containing 0.1% formic acid) and mobile phase B was 90% acetonitrile in water (containing 0.1% formic acid). The resolution of MS^1^ and MS^2^ scans was 120,000 and 30,000, respectively. Each MS^1^ was followed by 20 MS^2^ of the top 20 most intense ions, and dynamic exclusion was set as 30 s.

The mass spectrometry proteomics data have been deposited to the ProteomeXchange Consortium via the PRoteomics IDEntifications Database (PRIDE) (43) partner repository with the data set identifier PXD030206.

**(iv) Database search.** MS^2^ data were searched against a database containing HOMO_SAPIENS_9606 (20366 sequences) with reverse decoy database, and the common contamination database using MaxQuant 1.6.15.0. Trypsin/P was specified as cleavage enzyme, and the number of missed cleavages was set as 2. The mass tolerance for precursor ions and fragment ions was set as 20 ppm. Cysteine alkylation Carbamidomethyl (C) was set as fixed modification, and the variable modification was set to ('Acetyl [Protein N-term]', 'Oxidation [M]', 'Deamidation [NQ]'). The quantitative method was set as TMT-11PLEX, and the FDR for protein identification and PSM identification was adjusted to 1%.

**(v) Bioinformatic analysis.** The differentially expressed proteins were defined by *t* test *P*-value (*P* < 0.05) and fold change >1.3 or <0.77. MetaboAnalyst 5.0 was employed for the PCA and cluster analysis (44). GO and COG analysis was conducted for the differentially expressed proteins (45–47). Pathway analysis and protein-protein interaction were performed by searching against KEGG and STRING database (47, 48).

### Statistical analysis.

All the treatment groups were conducted in triplicates. One-way ANOVA analysis was performed for the statistical analysis. A probability *P* value was used as the criterion for statistical significance.

### Availability of data.

The mass spectrometry proteomics data are available via ProteomeXchange with identifier PXD030206.
