# Supplementary material for: Proteomic Response Revealed Signaling Pathways Involving in the Mechanism of Polymyxin B-Induced Melanogenesis
Source: Microbiol Spectr. 2022 Apr 4;10(2):e02730-21. doi: 10.1128/spectrum.02730-21 (PMC9045165; doi:10.1128/spectrum.02730-21)
Supplement: SUPPLEMENTAL FILE 1 — Supplemental material. Download SPECTRUM02730-21_Supp_1_seq9.pdf, PDF file, 1.7 MB [file spectrum02730-21_supp_1_seq9.pdf]

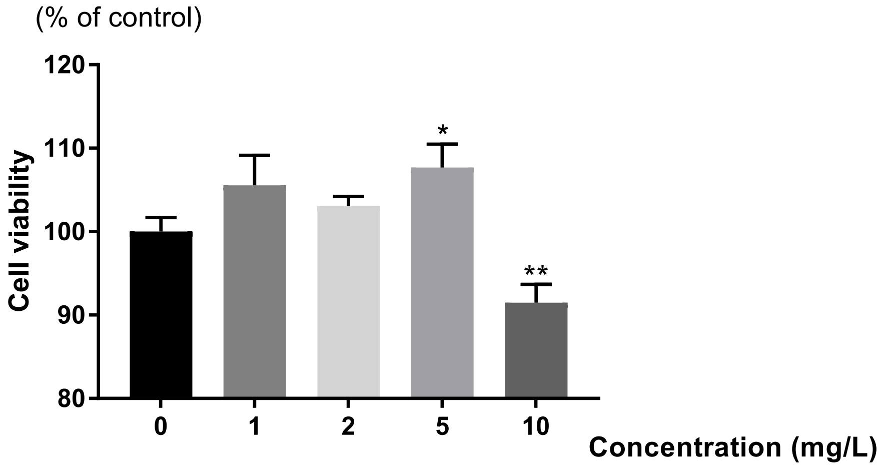

Supplementary Figure S1. Effects of polymyxin B on SK-MEL-2 cell viability. The change of SK-MEL-2 cells activity after exposure to the indicated concentrations(0~10 mg/L) of polymyxin B for 72 h was observed with CCK8 colorimeter. Data were normalized as percentage of the control group. *p<0.05, **p<0.01, ***p<0.001, compared to the blank control. All the treatment groups were conducted in triplicates.

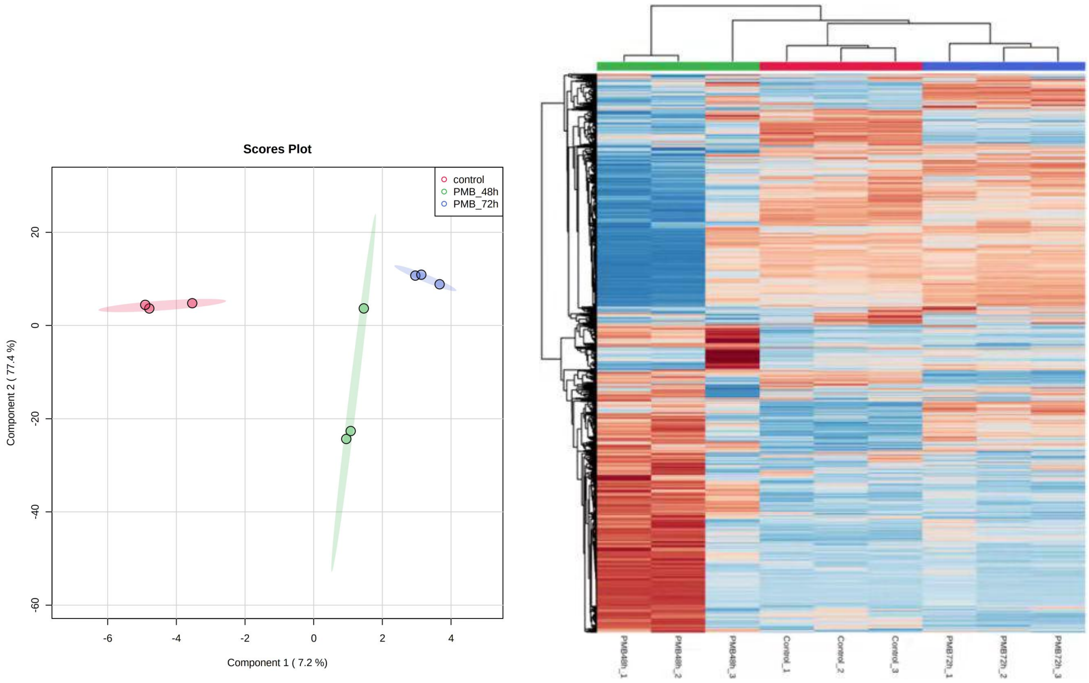

Supplementary Figure S2. Principal component analysis (PCA, left) and clustering analysis between repeated samples (right)

(a)

Compared sample name	Up-regulated	Down-regulated
PMB48h/Control	237	153
PMB72h/Control	49	49

(b)

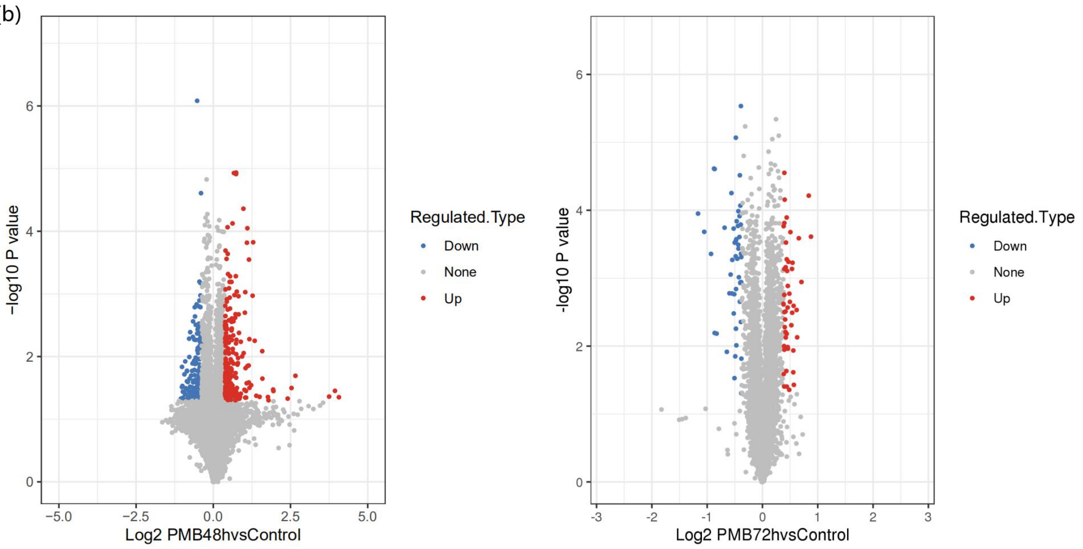

Supplementary Figure S3. Differentially expressed proteins of polymyxin B treated SK-MEL-2 cells compared with control group at 48 and 72 h. (a) Summary information of all differentially expressed proteins. (b) Volcanic map of differential proteins in SK-MEL-2 cells treated with polymyxin B for 48 h (left) and 72 h (right). The blue dots were down-regulated proteins, the red dots were up-regulated proteins, and the gray dots were proteins with no significant difference.

PMB48h *vs* Control

Protein accession	Protein description	Gene name	PMB48h/Control Ratio	PMB48h/Control P value	Regulated Type	KEGG KO No.	KEGG Gene	KEGG pathway	Biological Process	Cellular Component	Molecular Function	Pfam ID	Domain description	KOG category	KOG NO.	KOG description
Q01814	Plasma membrane calcium-transporting ATPase 2 OS=Homo sapiens OX=9606 GN=ATP2B2 PE=1 SV=2	ATP2B2	16.758	0.044544138	Up	K05850	ATP2B; P-type Ca2+ transporter type 2B [EC:7.2.2.10]	map04020 Calcium signaling pathway; map04022 cGMP-PKG signaling pathway; map04024 cAMP signaling pathway; map04261 Adrenergic signaling in cardiomyocytes; map04925 Aldosterone synthesis and secretion; map04961 Endocrine and other factor-regulated calcium reabsorption; map04970 Salivary secretion; map04972 Pancreatic secretion; map04978 Mineral absorption	GO:0000902 cell morphogenesis; GO:0000904 cell morphogenesis involved in differentiation; GO:0001505 regulation of neurotransmitter levels; GO:0001654 eye development; GO:0002064 epithelial cell development; GO:0002065 columnar/c	GO:0005622 intracellular; GO:0005623 cell; GO:0005737 cytoplasm; GO:0005783 endoplasmic reticulum; GO:0005886 plasma membrane; GO:0005887 integral component of plasma membrane; GO:0005929 cilium; GO:0012505 endomembrane system; GO:0014069	GO:0000166 nucleotide binding; GO:0003824 catalytic activity; GO:0005102 receptor binding; GO:0005215 transporter activity; GO:0005388 calcium-transporting ATPase activity; GO:0005488 binding; GO:0005509 calcium ion binding; GO:0005515 protein binding; GO:0005516	PF00690; PF08282	Cation transporter/ATPase, N-terminus; haloacid dehalogenase-like hydrolase	P	KOG0204	Calcium transporting ATPase
Q96M89	Coiled-coil domain-containing protein 138 OS=Homo sapiens OX=9606 GN=CCDC138 PE=1 SV=1	CCDC138	15.348	0.035248774	Up											
Q9HCG1	Zinc finger protein 160 OS=Homo sapiens OX=9606 GN=ZNF160 PE=2 SV=3	ZNF160	13.46	0.043574471	Up	K09228	KRAB; KRAB domain-containing zinc finger protein	map05168 Herpes simplex virus 1 infection	GO:0002376 immune system process; GO:0002520 immune system development; GO:0006355 regulation of transcription, DNA-templated; GO:0006357 regulation of transcription from RNA polymerase II promoter; GO:0007275 multicellular organism		GO:0000981 RNA polymerase II transcription factor activity, sequence-specific DNA binding; GO:0003700 transcription factor activity, sequence-specific DNA binding;	PF00096	Zinc finger, C2H2 type	K	KOG1721	FOG: Zn-finger
O15243	Leptin receptor gene-related protein OS=Homo sapiens OX=9606 GN=LEPROT PE=1 SV=1	LEPROT	6.32	0.02029383	Up				GO:0006810 transport; GO:0007034 vacuolar transport; GO:0009966 regulation of signal transduction; GO:0009968 negative regulation of signal transduction; GO:0010646 regulation of cell communication; GO:0010648 negative regulation of cell communication;	GO:0005622 intracellular; GO:0005623 cell; GO:0005737 cytoplasm; GO:0005768 endosome; GO:0005794 Golgi apparatus; GO:0012505 endomembrane system; GO:0031410 cytoplasmic vesicle; GO:0031982 vesicle; GO:0043226 organelle; GO:0043227	GO:0005102 receptor binding; GO:0005488 binding; GO:0005515 protein binding;			T	KOG2174	Leptin receptor gene-related protein
P21926	CD9 antigen OS=Homo sapiens OX=9606 GN=CD9 PE=1 SV=4	CD9	5.788	0.031755032	Up	K06460	CD9, TSPAN29; CD9 antigen	map04640 Hematopoietic cell lineage	GO:0000003 reproduction; GO:0001101 response to acid chemical; GO:0001775 cell activation; GO:0002576 platelet degranulation; GO:0006810 transport; GO:0006887 exocytosis; GO:0006897 endocytosis; GO:0006898 receptor-mediated endocytosi	GO:0005576 extracellular region; GO:0005615 extracellular space; GO:0005622 intracellular; GO:0005623 cell; GO:0005737 cytoplasm; GO:0005886 plasma membrane; GO:0005887 integral component of plasma membrane; GO:0009897 external side of plasma membrane; GO:00099	GO:0005102 receptor binding; GO:0005178 integrin binding; GO:0005488 binding; GO:0005515 protein binding; GO:0044877 macromolecular complex binding; GO:0050839 cell adhesion molecule binding;			S	KOG3882	Tetraspanin family integral membrane protein

P10144	Granzyme B OS=Homo sapiens OX=9606 GN=GZMB PE=1 SV=2	GZMB	5.307	0.046910209	Up	K01353	GZMB; granzyme B [EC:3.4.21.79]	map04210 Apoptosis; map04650 Natural killer cell mediated cytotoxicity; map04940 Type I diabetes mellitus; map05202 Transcriptional misregulation in cancer; map05320 Autoimmune thyroid disease; map05330 Allograft rejection; map05332 Graft-versus-host disease	GO:0001906 cell killing; GO:0001909 leukocyte mediated cytotoxicity; GO:0001913 T cell mediated cytotoxicity; GO:0002228 natural killer cell mediated immunity; GO:0002250 adaptive immune response; GO:0002252 immune effector process; GO:0002376 immune system process;	GO:0000323 lytic vacuole; GO:0001772 immunological synapse; GO:0005622 intracellular; GO:0005623 cell; GO:0005634 nucleus; GO:0005737 cytoplasm; GO:0005764 lysosome; GO:0005768 endosome; GO:0005769 early endosome; GO:0005773 vacuole; GO:00058	GO:0003824 catalytic activity; GO:0004175 endopeptidase activity; GO:0004252 serine-type endopeptidase activity; GO:0008233 peptidase activity; GO:0008236 serine-type peptidase activity; GO:0016787 hydrolase activity; GO:0017171 serine hydrolase activity;	PF00089	Trypsin	O	KOG3627	Trypsin
Q5BJF2	Sigma intracellular receptor 2 OS=Homo sapiens OX=9606 GN=TMEM97 PE=1 SV=1	TMEM97	3.852	0.035480166	Up				GO:0001558 regulation of cell growth; GO:0040008 regulation of growth; GO:0042592 homeostatic process; GO:0042632 cholesterol homeostasis; GO:0048878 chemical homeostasis; GO:0050789 regulation of biological process; GO:0050794	GO:0000323 lytic vacuole; GO:0005622 intracellular; GO:0005623 cell; GO:0005634 nucleus; GO:0005635 nuclear envelope; GO:0005737 cytoplasm; GO:0005764 lysosome; GO:0005773 vacuole; GO:0005783 endoplasmic reticulum; GO:0005791 rough endoplasmic						
P03372	Estrogen receptor OS=Homo sapiens OX=9606 GN=ESR1 PE=1 SV=2	ESR1	3.833	0.033647889	Up	K08550	ESR1, NR3A1; estrogen receptor alpha	map01522 Endocrine resistance; map04915 Estrogen signaling pathway; map04917 Prolactin signaling pathway; map04919 Thyroid hormone signaling pathway; map04961 Endocrine and other factor-regulated calcium reabsorption; map05200 Pathways in cancer; map05205 Proteoglycans in cancer; map05224 Breast cancer	GO:0000122 negative regulation of transcription from RNA polymerase II promoter; GO:0001505 regulation of neurotransmitter levels; GO:0006139 nucleobase-containing compound metabolic process; GO:0006325 chromatin organization; GO:0006338	GO:0000228 nuclear chromosome; GO:0000785 chromatin; GO:0000790 nuclear chromatin; GO:0005622 intracellular; GO:0005623 cell; GO:0005634 nucleus; GO:0005654 nucleoplasm; GO:0005694 chromosome; GO:0005737 cytoplasm; GO:0005794 Golgi	GO:0000976 transcription regulatory region sequence-specific DNA binding; GO:0000977 RNA polymerase II regulatory region sequence-specific DNA binding; GO:0000978 RNA polymerase II core promoter proximal region sequence-specific DNA binding;	PF00105; PF00104; PF12743	Zinc finger, C4 type (two domains); Ligand-binding domain of nuclear hormone receptor; Oestrogen-type nuclear receptor final C-terminal	K	KOG3575	FOG: Hormone receptors
Q9NYP7	Elongation of very long chain fatty acids protein 5 OS=Homo sapiens OX=9606 GN=ELOVL5 PE=1 SV=1	ELOVL5	3.454	0.049841514	Up	K10244	ELOVL5; elongation of very long chain fatty acids protein 5 [EC:2.3.1.199]	map00062 Fatty acid elongation; map01040 Biosynthesis of unsaturated fatty acids; map01100 Metabolic pathways; map01212 Fatty acid metabolism	GO:0000038 very long-chain fatty acid metabolic process; GO:0001676 long-chain fatty acid metabolic process; GO:0006082 organic acid metabolic process; GO:0006139 nucleobase-containing compound metabolic process; GO:0006163 purine nucleotide metabolic process; GO:0006164 purine	GO:0005622 intracellular; GO:0005623 cell; GO:0005737 cytoplasm; GO:0005783 endoplasmic reticulum; GO:0005789 endoplasmic reticulum membrane; GO:0012505 endomembrane system; GO:0016020 membrane; GO:0016021 integral component of	GO:0003824 catalytic activity; GO:0004312 fatty acid synthase activity; GO:0009922 fatty acid elongase activity; GO:0016740 transferase activity; GO:0016746 transferase activity, transferring acyl groups; GO:0016747 transferase activity, transferring acyl groups other than			I	KOG3071	Fatty acyl-CoA elongase/Polyunsaturated fatty acid specific elongation enzyme
Q5U3C3	Transmembrane protein 164 OS=Homo sapiens OX=9606 GN=TMEM164 PE=2 SV=1	TMEM164	3.405	0.044296552	Up											
O43688	Phospholipid phosphatase 2 OS=Homo sapiens OX=9606 GN=PLPP2 PE=1 SV=1	PLPP2	3.01	0.022471013	Up	K01080	PLPP1_2_3; phosphatidate phosphatase [EC:3.1.3.4]	map00561 Glycerolipid metabolism; map00564 Glycerophospholipid metabolism; map00565 Ether lipid metabolism; map00600 Sphingolipid metabolism; map01100 Metabolic pathways; map04072 Phospholipase D signaling pathway; map04666 Fc gamma R-mediated phagocytosis; map04975 Fat digestion and absorption; map05231 Choline metabolism in cancer	GO:0006464 cellular protein modification process; GO:0006470 protein dephosphorylation; GO:0006629 lipid metabolic process; GO:0006643 membrane lipid metabolic process; GO:0006644 phospholipid metabolic process; GO:0006665 sphingolipid metabolic process;	GO:0005623 cell; GO:0005886 plasma membrane; GO:0005887 integral component of plasma membrane; GO:0016020 membrane; GO:0016021 integral component of membrane; GO:0031224 intrinsic component of membrane; GO:0031226 intrinsic component of plasma membrane; GO:0044425 membrane	GO:0003824 catalytic activity; GO:0004721 phosphoprotein phosphatase activity; GO:0008195 phosphatidate phosphatase activity; GO:0016787 hydrolase activity; GO:0016788 hydrolase activity, acting on ester bonds; GO:0016791 phosphatase activity; GO:00423			I	KOG3030	Lipid phosphate phosphatase and related enzymes of the PAP2 family

Q9UBR2	Cathepsin Z OS=Homo sapiens OX=9606 GN=CTSZ PE=1 SV=1	CTSZ	2.999	0.008200064	Up	K08568	CTSZ; cathepsin X [EC:3.4.18.1]	map04142 Lysosome; map04210 Apoptosis	GO:0001763 morphogenesis of a branching structure; GO:0001775 cell activation; GO:0001990 regulation of systemic arterial blood pressure by hormone; GO:0001991 regulation of systemic arterial blood pressure by circulatory renin-angiotensin; GO:0002002 regulation	GO:0000323 lytic vacuole; GO:0005576 extracellular region; GO:0005615 extracellular space; GO:0005622 intracellular; GO:0005623 cell; GO:0005737 cytoplasm; GO:0005764 lysosome; GO:0005773 vacuole; GO:0005783 endoplasmic reticulum; GO:00057	GO:0003824 catalytic activity; GO:0004175 endopeptidase activity; GO:0004180 carboxypeptidase activity; GO:0004197 cysteine-type endopeptidase activity; GO:0008233 peptidase activity; GO:0008234 cysteine-type peptidase activity; GO:00082	PF00112	Papain family cysteine protease	O	KOG1543	Cysteine proteinase Cathepsin L
Q9Y519	Transmembrane protein 184B OS=Homo sapiens OX=9606 GN=TMEM184B PE=1 SV=2	TMEM184B	2.827	0.043982373	Up					GO:0016020 membrane; GO:0016021 integral component of membrane; GO:0031224 intrinsic component of membrane; GO:0044425 membrane part;	GO:0005215 transporter activity;			T	KOG2641	Predicted seven transmembrane receptor - rhodopsin family
O00400	Acetyl-coenzyme A transporter 1 OS=Homo sapiens OX=9606 GN=SLC33A1 PE=1 SV=1	SLC33A1	2.626	0.042289106	Up	K03372	ACATN, SLC33A1; MFS transporter, PAT family, solute carrier family 33 (acetyl-CoA transportor), member 1 [EC:2.3.1.-]	map00604 Glycosphingolipid biosynthesis - ganglio series; map01100 Metabolic pathways	GO:0006355 regulation of transcription, DNA-templated; GO:0006357 regulation of transcription from RNA polymerase II promoter; GO:0006810 transport; GO:0006811 ion transport; GO:0006812 cation transport; GO:0006820 anion transport; GO:0006862	GO:0000139 Golgi membrane; GO:0005622 intracellular; GO:0005623 cell; GO:0005737 cytoplasm; GO:0005783 endoplasmic reticulum; GO:0005789 endoplasmic reticulum membrane; GO:0005794 Golgi apparatus; GO:0005886 plasma membrane; GO:0005887 integral component	GO:0000295 adenine nucleotide transmembrane transporter activity; GO:0005215 transporter activity; GO:0005346 purine ribonucleotide transmembrane transporter activity; GO:0008324 cation transmembrane transporter activity; GO:0008509 anion transmembrane transporter activity;			P	KOG3574	Acetyl-CoA transporter
Q9UPU7	TBC1 domain family member 2B OS=Homo sapiens OX=9606 GN=TBC1D2B PE=1 SV=2	TBC1D2B	2.531	0.005601893	Up	K20166	TBC1D2B; TBC1 domain family member 2B		GO:0006810 transport; GO:0006886 intracellular protein transport; GO:0008104 protein localization; GO:0015031 protein transport; GO:0015833 peptide transport; GO:0031338 regulation of vesicle fusion; GO:0032879 regulation of localization; GO:0033036	GO:0005622 intracellular; GO:0005623 cell; GO:0005737 cytoplasm; GO:0005829 cytosol; GO:0012505 endomembrane system; GO:0044424 intracellular part; GO:0044444 cytoplasmic part; GO:0044464 cell part;	GO:0005096 GTPase activator activity; GO:0005488 binding; GO:0005515 protein binding; GO:0008047 enzyme activator activity; GO:0017016 Ras GTPase binding; GO:0017137 Rab GTPase binding; GO:0019899 enzyme binding; GO:0030234 enzyme regulator activity; GO:0030695 GTPase regulator	PF00169	PH domain	U	KOG2058	Ypt/Rab GTPase activating protein
O14602	Eukaryotic translation initiation factor 1A, Y-chromosomal OS=Homo sapiens OX=9606 GN=EIF1AY PE=1 SV=4	EIF1AY	2.447	0.000149911	Up	K03236	EIF1A; translation initiation factor 1A	map03013 RNA transport				PF01176	Translation initiation factor 1A / IF-1	J	KOG3403	Translation initiation factor 1A (eIF-1A)
P60602	Reactive oxygen species modulator 1 OS=Homo sapiens OX=9606 GN=ROMO1 PE=1 SV=1	ROMO1	2.417	0.001069496	Up				GO:0000302 response to reactive oxygen species; GO:0001302 replicative cell aging; GO:0001906 cell killing; GO:0002376 immune system process; GO:0006950 response to stress; GO:0006952 defense response; GO:0006955 immune response; GO:0006959 humoral immune response;	GO:0005622 intracellular; GO:0005623 cell; GO:0005737 cytoplasm; GO:0005739 mitochondrion; GO:0043226 organelle; GO:0043227 membrane-bounded organelle; GO:0043229 intracellular organelle; GO:0043231 intracellular membrane-bounded				S	KOG4096	Uncharacterized conserved protein

Q04828	Aldo-keto reductase family 1 member C1 OS=Homo sapiens OX=9606 GN=AKR1C1 PE=1 SV=1	AKR1C1	2.338	0.028630128	Up	K00212	AKR1C1; 20alpha/3alpha-hydroxysteroid dehydrogenase / dihydrodiol dehydrogenase [EC:1.1.1.149 1.1.1.357 1.3.1.20]	map00140 Steroid hormone biosynthesis; map00980 Metabolism of xenobiotics by cytochrome P450	GO:0000003 reproduction; GO:0000060 protein import into nucleus, translocation; GO:0000302 response to reactive oxygen species; GO:0001101 response to acid chemical; GO:0001516 prostaglandin biosynthetic process; GO:0001523 retinoid metabolic process;	GO:0005622 intracellular; GO:0005623 cell; GO:0005634 nucleus; GO:0005737 cytoplasm; GO:0005829 cytosol; GO:0043226 organelle; GO:0043227 membrane-bounded organelle; GO:0043229 intracellular organelle; GO:0043231 intracellular membrane-bounded	GO:0000166 nucleotide binding; GO:0001758 retinal dehydrogenase activity; GO:0003824 catalytic activity; GO:0004032 alditol:NADP+ 1-oxidoreductase activity; GO:0004033 aldo-keto reductase (NADP) activity; GO:0004303 estradiol 17-beta-dehydrogenase	PF00248	Aldo/keto reductase family	C	KOG1577	Aldo/keto reductase family proteins
P52926	High mobility group protein HMGI-C OS=Homo sapiens OX=9606 GN=HMGA2 PE=1 SV=1	HMGA2	2.251	0.031630421	Up											
Q8TCT8	Signal peptide peptidase-like 2A OS=Homo sapiens OX=9606 GN=SPPL2A PE=1 SV=2	SPPL2A	2.247	0.005271528	Up	K09596	SPPL2A; signal peptide peptidase-like 2A [EC:3.4.23.-]		GO:0001959 regulation of cytokine-mediated signaling pathway; GO:0002682 regulation of immune system process; GO:0006508 proteolysis; GO:0006509 membrane protein ectodomain proteolysis; GO:0006807 nitrogen compound metabolic process; GO:0008152 metabolic	GO:0000323 lytic vacuole; GO:0005622 intracellular; GO:0005623 cell; GO:0005737 cytoplasm; GO:0005764 lysosome; GO:0005765 lysosomal membrane; GO:0005768 endosome; GO:0005770 late endosome; GO:0005773 vacuole; GO:0005774 vacuolar membrane;	GO:0003824 catalytic activity; GO:0004175 endopeptidase activity; GO:0004190 aspartic-type endopeptidase activity; GO:0005488 binding; GO:0005515 protein binding; GO:0008233 peptidase activity; GO:0016787 hydrolase activity; GO:0042500 aspartic endopeptid			S	KOG2443	Uncharacterized conserved protein
Q9NRA2	Sialin OS=Homo sapiens OX=9606 GN=SLC17A5 PE=1 SV=2	SLC17A5	2.228	0.000283138	Up	K12301	SLC17A5; MFS transporter, ACS family, solute carrier family 17 (sodium-dependent inorganic phosphate cotransporter), member 5	map04142 Lysosome	GO:0006810 transport; GO:0006811 ion transport; GO:0006812 cation transport; GO:0006820 anion transport; GO:0008643 carbohydrate transport; GO:0009605 response to external stimulus; GO:0009607 response to biotic stimulus; GO:0009617 response to bacterium;	GO:0000323 lytic vacuole; GO:0005622 intracellular; GO:0005623 cell; GO:0005737 cytoplasm; GO:0005764 lysosome; GO:0005765 lysosomal membrane; GO:0005773 vacuole; GO:0005774 vacuolar membrane; GO:0005829 cytosol; GO:0005886 plasma membrane; GO:00058	GO:0005215 transporter activity; GO:0005342 organic acid transmembrane transporter activity; GO:0005351 sugar:proton symporter activity; GO:0005402 cation:sugar symporter activity; GO:0008324 cation transmembrane transporter activity; GO:0008509 anion transmemb			P	KOG2532	Permease of the major facilitator superfamily
Q14CN2	Calcium-activated chloride channel regulator 4 OS=Homo sapiens OX=9606 GN=CLCA4 PE=1 SV=2	CLCA4	2.218	0.025899558	Up	K05030	CLCA3_4; calcium-activated chloride channel regulator 3/4	map04924 Renin secretion; map04972 Pancreatic secretion	GO:0006508 proteolysis; GO:0006807 nitrogen compound metabolic process; GO:0006810 transport; GO:0006811 ion transport; GO:0006820 anion transport; GO:0006821 chloride transport; GO:0008152 metabolic process; GO:0015698 inorganic anion transport; GO:0019538 protein	GO:0005576 extracellular region; GO:0005623 cell; GO:0005886 plasma membrane; GO:0005887 integral component of plasma membrane; GO:0016020 membrane; GO:0016021 integral component of membrane; GO:0016324 apical plasma membrane; GO:0031224 intrinsic component of membrane;	GO:0003824 catalytic activity; GO:0004175 endopeptidase activity; GO:0004222 metalloendopeptidase activity; GO:0005215 transporter activity; GO:0005216 ion channel activity; GO:0005229 intracellular calcium activated chloride channel activity; GO:0005253 anion	PF00092	von Willebrand factor type A domain			
P14543	Nidogen-1 OS=Homo sapiens OX=9606 GN=NID1 PE=1 SV=3	NID1	2.211	0.013891975	Up	K06826	NID; nidogen (entactin)		GO:0001655 urogenital system development; GO:0001822 kidney development; GO:0007155 cell adhesion; GO:0007160 cell-matrix adhesion; GO:0007275 multicellular organism development; GO:0009987 cellular process; GO:0010810 regulation of cell-	GO:0005576 extracellular region; GO:0005604 basement membrane; GO:0005605 basal lamina; GO:0005623 cell; GO:0031012 extracellular matrix; GO:0044420 extracellular matrix component; GO:0044421 extracellular region part; GO:0044464 cell part;	GO:0005488 binding; GO:0005515 protein binding; GO:0005518 collagen binding; GO:0043236 laminin binding; GO:0043237 laminin-1 binding; GO:0043394 proteoglycan binding; GO:0044877 macromolecular complex binding; GO:0050840 extracellular matrix binding; GO:0097367	PF07474; PF07645; PF12662; PF12947; PF00086	G2F domain; Calcium-binding EGF domain; Complement Clr-like EGF-like; EGF domain; Thyroglobulin type-1 repeat	MW	KOG1214	Nidogen and related basement membrane protein proteins

Q9BUE6	Iron-sulfur cluster assembly 1 homolog, mitochondrial OS=Homo sapiens OX=9606 GN=ISCA1 PE=1 SV=1	ISCA1	2.151	0.014170178	Up	K22063	ISCA1; iron-sulfur cluster assembly 1		GO:0003008 system process; GO:0006790 sulfur compound metabolic process; GO:0006807 nitrogen compound metabolic process; GO:0008152 metabolic process; GO:0009987 cellular process; GO:0010467 gene expression; GO:0016043 cellular component organization; GO:0016226 iron-sulfur	GO:0005622 intracellular; GO:0005623 cell; GO:0005737 cytoplasm; GO:0005739 mitochondrion; GO:0005759 mitochondrial matrix; GO:0031974 membrane-enclosed lumen; GO:0043226 organelle; GO:0043227 membrane-bounded organelle; GO:00432	GO:0005488 binding; GO:0005506 iron ion binding; GO:0008198 ferrous iron binding; GO:0043167 ion binding; GO:0043169 cation binding; GO:0046872 metal ion binding; GO:0046914 transition metal ion binding; GO:0048037 cofactor binding; GO:0051536 iron-sulfur cluster			P	KOG1120	Fe-S cluster biosynthesis protein ISA1 (contains a HesB-like domain)
O15162	Phospholipid scramblase 1 OS=Homo sapiens OX=9606 GN=PLSCR1 PE=1 SV=1	PLSCR1	2.148	8.95143E-05	Up				GO:0001775 cell activation; GO:0002252 immune effector process; GO:0002376 immune system process; GO:0002526 acute inflammatory response; GO:0002682 regulation of immune system process; GO:0002684 positive regulation of immune system process; GO:0002694 regulation	GO:0005576 extracellular region; GO:0005622 intracellular; GO:0005623 cell; GO:0005634 nucleus; GO:0005730 nucleolus; GO:0005737 cytoplasm; GO:0005794 Golgi apparatus; GO:0005829 cytosol; GO:0005886 plasma membrane; GO:0005887 integral component of plasma membrane;	GO:0000981 RNA polymerase II transcription factor activity, sequence-specific DNA binding; GO:0000982 transcription factor activity, RNA polymerase II core promoter proximal region sequence-specific binding; GO:0001077 transcriptional activator activity,	PF03803	Scramblase	K	KOG0621	Phospholipid scramblase
Q9NRC6	Spectrin beta chain, non-erythrocytic 5 OS=Homo sapiens OX=9606 GN=SPTBN5 PE=1 SV=2	SPTBN5	2.127	0.000152465	Up	K06115	SPTB; spectrin beta		GO:0000165 MAPK cascade; GO:0000902 cell morphogenesis; GO:0000904 cell morphogenesis involved in differentiation; GO:0006464 cellular protein modification process; GO:0006468 protein phosphorylation; GO:0006793 phosphorus metabolic process; GO:0006796 phosphate-	GO:0001750 photoreceptor outer segment; GO:0005622 intracellular; GO:0005623 cell; GO:0005737 cytoplasm; GO:0005829 cytosol; GO:0005856 cytoskeleton; GO:0005886 plasma membrane; GO:0005929 cilium; GO:0005938 cell cortex; GO:0008091 spectrin;	GO:0003779 actin binding; GO:0005085 guanyl-nucleotide exchange factor activity; GO:0005088 Ras guanyl-nucleotide exchange factor activity; GO:0005488 binding; GO:0005515 protein binding; GO:0008092 cytoskeletal protein binding; GO:0017016 Ras GTPase binding; GO:00170	PF00307; PF00435	Calponin homology (CH) domain; Spectrin repeat	Z	KOG0517	Beta-spectrin
Q16831	Uridine phosphorylase 1 OS=Homo sapiens OX=9606 GN=UPP1 PE=1 SV=1	UPP1	2.114	0.026648542	Up	K00757	udp, UPP; uridine phosphorylase [EC:2.4.2.3]	map00240 Pyrimidine metabolism; map00983 Drug metabolism - other enzymes; map01100 Metabolic pathways	GO:0006139 nucleobase-containing compound metabolic process; GO:0006213 pyrimidine nucleoside metabolic process; GO:0006218 uridine catabolic process; GO:0006220 pyrimidine nucleotide metabolic process; GO:0006725 cellular aromatic compound metabolic process; GO:0006753	GO:0005622 intracellular; GO:0005623 cell; GO:0005634 nucleus; GO:0005654 nucleoplasm; GO:0005737 cytoplasm; GO:0005829 cytosol; GO:0031974 membrane-enclosed lumen; GO:0031981 nuclear lumen; GO:0043226 organelle; GO:0043227 membrane-	GO:0003824 catalytic activity; GO:0004850 uridine phosphorylase activity; GO:0016740 transferase activity; GO:0016757 transferase activity, transferring glycosyl groups; GO:0016763 transferase activity, transferring pentosyl groups;	PF01048	Phosphorylase superfamily	F	KOG3728	Uridine phosphorylase
Q9BUN8	Derlin-1 OS=Homo sapiens OX=9606 GN=DERL1 PE=1 SV=1	DERL1	2.087	0.045156305	Up	K11519	DERL1; Derlin-1	map04141 Protein processing in endoplasmic reticulum; map05014 Amyotrophic lateral sclerosis (ALS)	GO:0006457 protein folding; GO:0006464 cellular protein modification process; GO:0006508 proteolysis; GO:0006511 ubiquitin-dependent protein catabolic process; GO:0006515 misfolded or incompletely synthesized protein catabolic process; GO:0006807 nitrogen compound	GO:0005622 intracellular; GO:0005623 cell; GO:0005737 cytoplasm; GO:0005768 endosome; GO:0005769 early endosome; GO:0005770 late endosome; GO:0005783 endoplasmic reticulum; GO:0005789 endoplasmic reticulum membrane; GO:0012505 endomemb	GO:0002020 protease binding; GO:0005102 receptor binding; GO:0005488 binding; GO:0005515 protein binding; GO:0019899 enzyme binding; GO:0031625 ubiquitin protein ligase binding; GO:0038023 signaling receptor activity; GO:0042287 MHC protein binding; GO:0042288 MHC			U	KOG0858	Predicted membrane protein

P61916	NPC intracellular cholesterol transporter 2 OS=Homo sapiens OX=9606 GN=NPC2 PE=1 SV=1	NPC2	2.079	0.035470812	Up	K13443	NPC2; Niemann-Pick C2 protein	map04142 Lysosome; map04979 Cholesterol metabolism	GO:0001775 cell activation; GO:0002252 immune effector process; GO:0002263 cell activation involved in immune response; GO:0002274 myeloid leukocyte activation; GO:0002275 myeloid cell activation involved in immune response; GO:0002283 neutrophil activation involved in immune response;	GO:0000323 lytic vacuole; GO:0005576 extracellular region; GO:0005622 intracellular; GO:0005623 cell; GO:0005737 cytoplasm; GO:0005764 lysosome; GO:0005766 primary lysosome; GO:0005773 vacuole; GO:0005775 vacuolar lumen; GO:0012505 endomemb	GO:0005215 transporter activity; GO:0005319 lipid transporter activity; GO:0005488 binding; GO:0005496 steroid binding; GO:0005515 protein binding; GO:0008289 lipid binding; GO:0015248 sterol transporter activity; GO:0015485 cholesterol binding; GO:0017127 cholesterol transporter	PF02221	ML domain	S	KOG4063	Major epididymal secretory protein HE1
Q6NXT1	Ankyrin repeat domain-containing protein 54 OS=Homo sapiens OX=9606 GN=ANKRD54 PE=1 SV=2	ANKRD54	2.06	0.015325724	Up	K21442	ANKRD54; ankyrin repeat domain-containing protein 54		GO:0001932 regulation of protein phosphorylation; GO:0002682 regulation of immune system process; GO:0002684 positive regulation of immune system process; GO:0006810 transport; GO:0006913 nucleocytoplasmic transport; GO:0009966 regulation of signal transductio	GO:0005622 intracellular; GO:0005623 cell; GO:0005634 nucleus; GO:0005737 cytoplasm; GO:0030496 midbody; GO:0043226 organelle; GO:0043227 membrane-bounded organelle; GO:0043229 intracellular organelle; GO:0043231 intracellular membrane-	GO:0005488 binding; GO:0019207 kinase regulator activity; GO:0019887 protein kinase regulator activity; GO:0030234 enzyme regulator activity; GO:0044877 macromolecular complex binding; GO:0098772 molecular function regulator;			S	KOG0504	FOG: Ankyrin repeat
P47813	Eukaryotic translation initiation factor 1A, X-chromosomal OS=Homo sapiens OX=9606 GN=EIF1AX PE=1 SV=2	EIF1AX	2.049	0.000937519	Up	K03236	EIF1A; translation initiation factor 1A	map03013 RNA transport	GO:0006412 translation; GO:0006413 translational initiation; GO:0006518 peptide metabolic process; GO:0006807 nitrogen compound metabolic process; GO:0008152 metabolic process; GO:0009058 biosynthetic process; GO:0009059 macromolecule biosynthetic process; GO:00099	GO:0005622 intracellular; GO:0005623 cell; GO:0005737 cytoplasm; GO:0005829 cytosol; GO:0044424 intracellular part; GO:0044444 cytoplasmic part; GO:0044464 cell part;	GO:0003676 nucleic acid binding; GO:0003723 RNA binding; GO:0005488 binding; GO:0008135 translation factor activity, RNA binding; GO:0097159 organic cyclic compound binding; GO:1901363 heterocyclic compound binding;	PF01176	Translation initiation factor 1A / IF-1	J	KOG3403	Translation initiation factor 1A (eIF-1A)
Q8N806	Putative E3 ubiquitin-protein ligase UBR7 OS=Homo sapiens OX=9606 GN=UBR7 PE=1 SV=2	UBR7	2.049	0.04516257	Up	K11979	UBR7; E3 ubiquitin-protein ligase UBR7 [EC:2.3.2.27]							O	KOG2752	Uncharacterized conserved protein, contains N-recognin-type Zn-finger
P03897	NADH-ubiquinone oxidoreductase chain 3 OS=Homo sapiens OX=9606 GN=MT-ND3 PE=1 SV=1	MT-ND3	2.032	0.001996411	Up	K03880	ND3; NADH-ubiquinone oxidoreductase chain 3 [EC:7.1.1.2]	map00190 Oxidative phosphorylation; map01100 Metabolic pathways; map04714 Thermogenesis; map04723 Retrograde endocannabinoid signaling; map05012 Parkinson disease	GO:0006091 generation of precursor metabolites and energy; GO:0006119 oxidative phosphorylation; GO:0006120 mitochondrial electron transport, NADH to ubiquinone; GO:0006139 nucleobase-containing compound metabolic process; GO:0006163 purine nucleotide metabolic	GO:0005622 intracellular; GO:0005623 cell; GO:0005737 cytoplasm; GO:0005739 mitochondrion; GO:0005740 mitochondrial envelope; GO:0005743 mitochondrial inner membrane; GO:0005746 mitochondrial respiratory chain; GO:0005747	GO:0003824 catalytic activity; GO:0003954 NADH dehydrogenase activity; GO:0008137 NADH dehydrogenase (ubiquinone) activity; GO:0016491 oxidoreductase activity; GO:0016651 oxidoreductase activity, acting on NAD(P)H; GO:0016655 oxidoreductase activity,			C	KOG4662	NADH dehydrogenase subunit 3 and related proteins
Q6PII5	Hydroxyacylglutathione hydrolase-like protein OS=Homo sapiens OX=9606 GN=HAGHL PE=2 SV=1	HAGHL	2.012	0.033991186	Up							PF00753	Metallo-beta-lactamase superfamil	S	KOG0813	Glyoxylase
Q9H8K7	ATPase PAAT OS=Homo sapiens OX=9606 GN=PAAT PE=1 SV=2	PAAT	1.976	0.008809518	Up						GO:0005488 binding; GO:0005515 protein binding; GO:0042802 identical protein binding;					

Q2T9J0	Peroxisomal leader peptide-processing protease OS=Homo sapiens OX=9606 GN=TYSND1 PE=1 SV=3	TYSND1	1.966	4.36792E-05	Up	K23012	TYSND1; peroxisomal leader peptide-processing protease [EC:3.4.21.-]		GO:0006508 proteolysis; GO:0006605 protein targeting; GO:0006625 protein targeting to peroxisome; GO:0006807 nitrogen compound metabolic process; GO:0006810 transport; GO:0006886 intracellular protein transport; GO:0006996 organelle organization; GO:0007031	GO:0005622 intracellular; GO:0005623 cell; GO:0005737 cytoplasm; GO:0005777 peroxisome; GO:0005782 peroxisomal matrix; GO:0005829 cytosol; GO:0031907 microbody lumen; GO:0031974 membrane-enclosed lumen; GO:0042579 microbody;	GO:0002020 protease binding; GO:0003824 catalytic activity; GO:0004175 endopeptidase activity; GO:0004252 serine-type endopeptidase activity; GO:0005488 binding; GO:0005515 protein binding; GO:0008233 peptidase activity; GO:0008234 cysteine-type peptidase	PF13365	Trypsin-like peptidase domain	O	KOG1320	Serine protease
Q8IYI0	Shieldin complex subunit 1 OS=Homo sapiens OX=9606 GN=SHLD1 PE=1 SV=1	SHLD1	1.909	0.009951421	Up	K19991	CHGB; chromogranin-B		GO:0000018 regulation of DNA recombination; GO:0002637 regulation of immunoglobulin production; GO:0002639 positive regulation of immunoglobulin production; GO:0002682 regulation of immune system process; GO:0002684 positive regulation of immune system	GO:0005622 intracellular; GO:0005623 cell; GO:0005694 chromosome; GO:0035861 site of double-strand break; GO:0043226 organelle; GO:0043228 non-membrane-bounded organelle; GO:0043229 intracellular organelle; GO:0043232 intracellular non-						
Q9HCE0	Ectopic P granules protein 5 homolog OS=Homo sapiens OX=9606 GN=EPG5 PE=1 SV=2	EPG5	1.856	0.039216153	Up				GO:0006810 transport; GO:0006914 autophagy; GO:0008152 metabolic process; GO:0009056 catabolic process; GO:0009987 cellular process; GO:0016043 cellular component organization; GO:0016192 vesicle-mediated transport; GO:0016197 endosomal transport; GO:00162	GO:0005622 intracellular; GO:0005623 cell; GO:0005737 cytoplasm; GO:0044424 intracellular part; GO:0044464 cell part;				S	KOG3622	Uncharacterized conserved protein
P13473	Lysosome-associated membrane glycoprotein 2 OS=Homo sapiens OX=9606 GN=LAMP2 PE=1 SV=2	LAMP2	1.854	0.005801744	Up	K06528	LAMP1_2, CD107; lysosomal-associated membrane protein 1/2	map04140 Autophagy - animal; map04142 Lysosome; map04145 Phagosome; map05152 Tuberculosis	GO:0001775 cell activation; GO:0002252 immune effector process; GO:0002263 cell activation involved in immune response; GO:0002274 myeloid leukocyte activation; GO:0002275 myeloid cell activation involved in immune response; GO:0002283 neutrophil activation involved in immune response;	GO:0000323 lytic vacuole; GO:0000421 autophagosome membrane; GO:0005576 extracellular region; GO:0005615 extracellular space; GO:0005622 intracellular; GO:0005623 cell; GO:0005737 cytoplasm; GO:0005764 lysosome; GO:0005765 lysosomal	GO:0005488 binding; GO:0005515 protein binding; GO:0019899 enzyme binding; GO:0019904 protein domain specific binding;			S	KOG4818	Lysosomal-associated membrane protein
P23276	Kell blood group glycoprotein OS=Homo sapiens OX=9606 GN=KEL PE=1 SV=2	KEL	1.821	0.044291036	Up	K06577	KEL, CD238; Kell blood group glycoprotein [EC:3.4.24.-]		GO:0003008 system process; GO:0003013 circulatory system process; GO:0003018 vascular process in circulatory system; GO:0006508 proteolysis; GO:0006807 nitrogen compound metabolic process; GO:0006873 cellular ion homeostasis; GO:0006874 cellular calcium ion homeostasis;	GO:0005623 cell; GO:0005886 plasma membrane; GO:0016020 membrane; GO:0016021 integral component of membrane; GO:0031224 intrinsic component of membrane; GO:0044425 membrane part; GO:0044464 cell part; GO:0071944 cell periphery;	GO:0003824 catalytic activity; GO:0004175 endopeptidase activity; GO:0004222 metalloendopeptidase activity; GO:0008233 peptidase activity; GO:0008237 metallopeptidase activity; GO:0016787 hydrolase activity; GO:0070011 peptidase activity, acting on			E	KOG3624	M13 family peptidase

P52735	Guanine nucleotide exchange factor VAV2 OS=Homo sapiens OX=9606 GN=VAV2 PE=1 SV=2	VAV2	1.796	0.047058796	Up	K05730	VAV; guanine nucleotide exchange factor VAV	map04015 Rap1 signaling pathway; map04024 cAMP signaling pathway; map04062 Chemokine signaling pathway; map04510 Focal adhesion; map04650 Natural killer cell mediated cytotoxicity; map04660 T cell receptor signaling pathway; map04662 B cell receptor signaling pathway; map04664 Fc epsilon RI signaling pathway; map04666 Fc gamma R-mediated phagocytosis; map04670 Leukocyte transendothelial migration; map04810 Regulation of actin cytoskeleton; map05135 Yersinia infection; map05205 Proteoglycans in cancer	GO:0001775 cell activation; GO:0002252 immune effector process; GO:0002253 activation of immune response; GO:0002376 immune system process; GO:0002429 immune response-activating cell surface receptor signaling pathway; GO:0002431 Fc receptor mediated stimulatory signaling pathway;	GO:0005622 intracellular; GO:0005623 cell; GO:0005737 cytoplasm; GO:0005829 cytosol; GO:0044424 intracellular part; GO:0044444 cytoplasmic part; GO:0044464 cell part;	GO:0001784 phosphotyrosine binding; GO:0005085 guanyl-nucleotide exchange factor activity; GO:0005088 Ras guanyl-nucleotide exchange factor activity; GO:0005089 Rho guanyl-nucleotide exchange factor activity; GO:0005488 binding; GO:0005515 protein binding; GO:00170	PF11971; PF00621; PF00169; PF00130; PF07653; PF00017	CAMSAP CH domain; RhoGEF domain; PH domain; Phorbol esters/diacylglycerol binding domain (C1 domain); Variant SH3 domain; SH2 domain	T	KOG2996	Rho guanine nucleotide exchange factor VAV3
Q9Y3A3	MOB-like protein phocein OS=Homo sapiens OX=9606 GN=MOB4 PE=1 SV=1	MOB4	1.78	0.004191787	Up	K04078	groES, HSPE1; chaperonin GroES		GO:0006810 transport; GO:0006900 membrane budding; GO:0006996 organelle organization; GO:0009987 cellular process; GO:0016043 cellular component organization; GO:0016050 vesicle organization; GO:0016192 vesicle-mediated transport; GO:0051179 localization	GO:0000139 Golgi membrane; GO:0005622 intracellular; GO:0005623 cell; GO:0005737 cytoplasm; GO:0005794 Golgi apparatus; GO:0005795 Golgi stack; GO:0005829 cytosol; GO:0012505 endomembrane system; GO:0016020 membrane; GO:0030425 dendrite;	GO:0005488 binding; GO:0005515 protein binding; GO:0019899 enzyme binding; GO:0019900 kinase binding;			D	KOG1852	Cell cycle-associated protein
Q9Y6N1	Cytochrome c oxidase assembly protein COX11, mitochondrial OS=Homo sapiens OX=9606 GN=COX11 PE=1 SV=3	COX11	1.766	0.002119723	Up	K02258	COX11, ctaG; cytochrome c oxidase assembly protein subunit 11	map00190 Oxidative phosphorylation; map01100 Metabolic pathways; map04714 Thermogenesis	GO:0006091 generation of precursor metabolites and energy; GO:0006810 transport; GO:0006811 ion transport; GO:0006812 cation transport; GO:0007585 respiratory gaseous exchange; GO:0008152 metabolic process; GO:0008535 respiratory chain complex IV	GO:0005622 intracellular; GO:0005623 cell; GO:0005737 cytoplasm; GO:0005739 mitochondrion; GO:0005740 mitochondrial envelope; GO:0005743 mitochondrial inner membrane; GO:0016020 membrane; GO:0016021 integral component of membrane;	GO:0003824 catalytic activity; GO:0004129 cytochrome-c oxidase activity; GO:0005215 transporter activity; GO:0008324 cation transmembrane transporter activity; GO:0009055 electron carrier activity; GO:0015002 heme-copper terminal oxidase activity; GO:0015075 ion transmemb			C	KOG2540	Cytochrome oxidase assembly factor COX11
Q9NRP0	Oligosaccharyltransferase complex subunit OSTC OS=Homo sapiens OX=9606 GN=OSTC PE=1 SV=1	OSTC	1.76	0.001089207	Up					GO:0005622 intracellular; GO:0005623 cell; GO:0005737 cytoplasm; GO:0005783 endoplasmic reticulum; GO:0005789 endoplasmic reticulum membrane; GO:0008250 oligosaccharyltransferase complex; GO:0012505 endomembrane system; GO:00160				S	KOG3356	Predicted membrane protein
Q99584	Protein S100-A13 OS=Homo sapiens OX=9606 GN=S100A13 PE=1 SV=1	S100A13	1.754	0.04213595	Up	K23765			GO:0001775 cell activation; GO:0001816 cytokine production; GO:0002252 immune effector process; GO:0002263 cell activation involved in immune response; GO:0002274 myeloid leukocyte activation; GO:0002275 myeloid cell activation involved in immune response; GO:0002279 mast cell activation	GO:0005576 extracellular region; GO:0005615 extracellular space; GO:0005622 intracellular; GO:0005623 cell; GO:0005634 nucleus; GO:0005730 nucleolus; GO:0005737 cytoplasm; GO:0005829 cytosol; GO:0005886 plasma membrane; GO:0016020 membrane; GO:00319	GO:0005102 receptor binding; GO:0005488 binding; GO:0005507 copper ion binding; GO:0005509 calcium ion binding; GO:0005515 protein binding; GO:0008144 drug binding; GO:0008270 zinc ion binding; GO:0017134 fibroblast growth factor binding; GO:0019838 growth factor	PF01023	S-100/ICaBP type calcium binding domain			

Q5TAP6	U3 small nucleolar RNA-associated protein 14 homolog C OS=Homo sapiens OX=9606 GN=UTP14C PE=1 SV=1	UTP14C	1.75	0.044828902	Up	K14567	UTP14; U3 small nucleolar RNA-associated protein 14	map03008 Ribosome biogenesis in eukaryotes	GO:0000003 reproduction; GO:0006139 nucleobase-containing compound metabolic process; GO:0006364 rRNA processing; GO:0006396 RNA processing; GO:0006725 cellular aromatic compound metabolic process; GO:0006807 nitrogen compound metabolic process; GO:0007276 gamete generation;	GO:0005622 intracellular; GO:0005623 cell; GO:0005634 nucleus; GO:0005654 nucleoplasm; GO:0005730 nucleolus; GO:0005737 cytoplasm; GO:0005829 cytosol; GO:0030684 preribosome; GO:0031974 membrane-enclosed lumen; GO:0031981 nuclear				S	KOG2172	Uncharacterized conserved protein
P33552	Cyclin-dependent kinases regulatory subunit 2 OS=Homo sapiens OX=9606 GN=CKS2 PE=1 SV=1	CKS2	1.747	0.040281489	Up	K02219	CKS1; cyclin-dependent kinase regulatory subunit CKS1	map05200 Pathways in cancer; map05222 Small cell lung cancer	GO:0000079 regulation of cyclin-dependent protein serine/threonine kinase activity; GO:0001932 regulation of protein phosphorylation; GO:0001934 positive regulation of protein phosphorylation; GO:0006355 regulation of transcription, DNA-templated; GO:0007346	GO:0000151 ubiquitin ligase complex; GO:0000307 cyclin-dependent protein kinase holoenzyme complex; GO:0005622 intracellular; GO:0005623 cell; GO:0019005 SCF ubiquitin ligase complex; GO:0031461 cullin-RING ubiquitin ligase complex; GO:0032991	GO:0005488 binding; GO:0005515 protein binding; GO:0008047 enzyme activator activity; GO:0016538 cyclin-dependent protein serine/threonine kinase regulator activity; GO:0019207 kinase regulator activity; GO:0019209 kinase activator activity; GO:0019887 protein kinase regulator activity;	PF01111	Cyclin-dependent kinase regulatory subunit	D	KOG3484	Cyclin-dependent protein kinase CDC28, regulatory subunit CKS1, and related proteins
Q5MNZ9	WD repeat domain phosphoinositide-interacting protein 1 OS=Homo sapiens OX=9606 GN=WIPI1 PE=1 SV=3	WIPI1	1.742	0.01055473	Up	K17908	WIPI1_2, ATG18; autophagy-related protein 18	map04136 Autophagy - other; map04140 Autophagy - animal; map05131 Shigellosis	GO:0000045 autophagosome assembly; GO:0000422 mitophagy; GO:0006464 cellular protein modification process; GO:0006497 protein lipidation; GO:0006807 nitrogen compound metabolic process; GO:0006810 transport; GO:0006892 post-Golgi vesicle-mediated transport; GO:00068	GO:0000139 Golgi membrane; GO:0000407 pre-autophagosomal structure; GO:0000421 autophagosome membrane; GO:0005622 intracellular; GO:0005623 cell; GO:0005737 cytoplasm; GO:0005768 endosome; GO:0005773 vacuole; GO:0005774 vacuolar membrane;	GO:0005102 receptor binding; GO:0005488 binding; GO:0005515 protein binding; GO:0005543 phospholipid binding; GO:0008134 transcription factor binding; GO:0008289 lipid binding; GO:0030331 estrogen receptor binding; GO:0032266 phosphatidylinositol-3-phosphate binding;			S	KOG2110	Uncharacterized conserved protein, contains WD40 repeats
Q66LE6	Serine/threonine-protein phosphatase 2A 55 kDa regulatory subunit B delta isoform OS=Homo sapiens OX=9606 GN=PPP2R2D PE=1 SV=1	PPP2R2D	1.739	0.047025238	Up	K04354	PPP2R2; serine/threonine-protein phosphatase 2A regulatory subunit B	map03015 mRNA surveillance pathway; map04071 Sphingolipid signaling pathway; map04151 PI3K-Akt signaling pathway; map04152 AMPK signaling pathway; map04261 Adrenergic signaling in cardiomyocytes; map04390 Hippo signaling pathway; map04391 Hippo signaling pathway - fly; map04530 Tight junction; map04728 Dopaminergic synapse; map05142 Chagas disease (American trypanosomiasis); map05160 Hepatitis C; map05165 Human papillomavirus infection	GO:0000278 mitotic cell cycle; GO:0000280 nuclear division; GO:0006464 cellular protein modification process; GO:0006470 protein dephosphorylation; GO:0006793 phosphorus metabolic process; GO:0006796 phosphate-containing compound metabolic process; GO:0006807 nitrogen compound metabolic	GO:0000159 protein phosphatase type 2A complex; GO:0005622 intracellular; GO:0005623 cell; GO:0005634 nucleus; GO:0005654 nucleoplasm; GO:0005737 cytoplasm; GO:0005829 cytosol; GO:0008287 protein serine/threonine phosphatase complex; GO:0031974 membrane-	GO:0003824 catalytic activity; GO:0008195 phosphatidate phosphatase activity; GO:0016787 hydrolase activity; GO:0016788 hydrolase activity, acting on ester bonds; GO:0016791 phosphatase activity; GO:0019208 phosphatase regulator activity; GO:0019888 protein			T	KOG1354	Serine/threonine protein phosphatase 2A, regulatory subunit
P23297	Protein S100-A1 OS=Homo sapiens OX=9606 GN=S100A1 PE=1 SV=2	S100A1	1.735	0.01270885	Up	K23758			GO:0000122 negative regulation of transcription from RNA polymerase II promoter; GO:0002218 activation of innate immune response; GO:0002221 pattern recognition receptor signaling pathway; GO:0002224 toll-like receptor signaling pathway; GO:0002253 activation of immune	GO:0005576 extracellular region; GO:0005622 intracellular; GO:0005623 cell; GO:0005634 nucleus; GO:0005737 cytoplasm; GO:0005783 endoplasmic reticulum; GO:0005829 cytosol; GO:0012505 endomembrane system; GO:0016528 sarcoplasm;	GO:0005488 binding; GO:0005509 calcium ion binding; GO:0005515 protein binding; GO:0019899 enzyme binding; GO:0042802 identical protein binding; GO:0042803 protein homodimerization activity; GO:0043167 ion binding; GO:0043169 cation binding; GO:0044548 S100 protein	PF01023; PF00036	S-100/ICaBP type calcium binding domain; EF hand			

Q96FJ0	AMSH-like protease OS=Homo sapiens OX=9606 GN=STAMBPL1 PE=1 SV=2	STAMBPL1	1.718	0.013466303	Up	K11867	STAMBPL1; AMSH-like protease [EC:3.4.19.12]		GO:0006464 cellular protein modification process; GO:0006508 proteolysis; GO:0006807 nitrogen compound metabolic process; GO:0008152 metabolic process; GO:0009987 cellular process; GO:0016579 protein deubiquitination; GO:0019538 protein metabolic process; GO:0036211 protein modificatio	GO:0005622 intracellular; GO:0005623 cell; GO:0005737 cytoplasm; GO:0005829 cytosol; GO:0044424 intracellular part; GO:0044444 cytoplasmic part; GO:0044464 cell part;	GO:0003824 catalytic activity; GO:0016787 hydrolase activity; GO:0061578 Lys63-specific deubiquitinase activity; GO:0101005 ubiquitinyl hydrolase activity;	PF08969	Domain of unknown function (DUF1873)	O	KOG2880	SMAD6 interacting protein AMSH, contains JAB/MPN/Mov34 domain
Q96PV6	Leukocyte receptor cluster member 8 OS=Homo sapiens OX=9606 GN=LENG8 PE=1 SV=3	LENG8	1.704	0.046929752	Up	K23802	THP3, LENG8; SAC3 family protein THP3/LENG8							K	KOG1861	Leucine permease transcriptional regulator
P53999	Activated RNA polymerase II transcriptional coactivator p15 OS=Homo sapiens OX=9606 GN=SUB1 PE=1 SV=3	SUB1	1.698	0.031681549	Up				GO:0006139 nucleobase-containing compound metabolic process; GO:0006351 transcription, DNA-templated; GO:0006355 regulation of transcription, DNA-templated; GO:0006357 regulation of transcription from RNA polymerase II promoter; GO:0006366	GO:0005622 intracellular; GO:0005623 cell; GO:0005634 nucleus; GO:0005667 transcription factor complex; GO:0005730 nucleolus; GO:0031974 membrane-enclosed lumen; GO:0031981 nuclear lumen; GO:0032991 macromolecular complex; GO:0043226	GO:0000981 RNA polymerase II transcription factor activity, sequence-specific DNA binding; GO:0001205 transcriptional activator activity, RNA polymerase II distal enhancer sequence-specific binding; GO:0001228 transcriptional activator activity, RNA	PF02229	Transcriptional Coactivator p15 (PC4)	K	KOG2712	Transcriptional coactivator
Q8IYT2	Cap-specific mRNA (nucleoside-2'-O-)-methyltransferase 2 OS=Homo sapiens OX=9606 GN=CMTR2 PE=1 SV=2	CMTR2	1.694	0.000920524	Up	K14590	CMTR2, FTSJD1, AFT; cap2 methyltransferase [EC:2.1.1.296]		GO:0001510 RNA methylation; GO:0006139 nucleobase-containing compound metabolic process; GO:0006370 7-methylguanosine mRNA capping; GO:0006396 RNA processing; GO:0006397 mRNA processing; GO:0006725 cellular aromatic compound metabolic process; GO:0006807 nitrogen	GO:0005622 intracellular; GO:0005623 cell; GO:0005634 nucleus; GO:0005737 cytoplasm; GO:0043226 organelle; GO:0043227 membrane-bounded organelle; GO:0043229 intracellular organelle; GO:0043231 intracellular membrane-bounded organelle; GO:00444	GO:0003824 catalytic activity; GO:0004483 mRNA (nucleoside-2'-O-)-methyltransferase activity; GO:0008168 methyltransferase activity; GO:0008171 O-methyltransferase activity; GO:0008173 RNA methyltransferase activity; GO:0008174 mRNA methyltransferase activity; GO:00087			A	KOG3674	FtsJ-like RNA methyltransferase
P04156	Major prion protein OS=Homo sapiens OX=9606 GN=PRNP PE=1 SV=1	PRNP	1.679	0.005412396	Up	K05634	PRNP, PrP, CD230; prion protein	map04216 Ferroptosis; map05020 Prion diseases	GO:0001817 regulation of cytokine production; GO:0001818 negative regulation of cytokine production; GO:0001932 regulation of protein phosphorylation; GO:0001933 negative regulation of protein phosphorylation; GO:0001934 positive regulation of protein phosphorylation; GO:0002682 regulation	GO:0005622 intracellular; GO:0005623 cell; GO:0005634 nucleus; GO:0005635 nuclear envelope; GO:0005737 cytoplasm; GO:0005739 mitochondrion; GO:0005740 mitochondrial envelope; GO:0005741 mitochondrial outer membrane; GO:0005783 endoplasmi	GO:0001540 beta-amyloid binding; GO:0001664 G-protein coupled receptor binding; GO:0002020 protease binding; GO:0005102 receptor binding; GO:0005488 binding; GO:0005507 copper ion binding; GO:0005515 protein binding; GO:0005521 lamin binding; GO:0005539 glycosamin	PF00377	Prion/Doppel alpha-helical domain			

Q9Y4P1	Cysteine protease ATG4B OS=Homo sapiens OX=9606 GN=ATG4B PE=1 SV=2	ATG4B	1.675	0.009155604	Up	K08342	ATG4; cysteine protease ATG4 [EC:3.4.22.-]	map04136 Autophagy - other; map04140 Autophagy - animal	GO:0000045 autophagosome assembly; GO:0000422 mitophagy; GO:0006464 cellular protein modification process; GO:0006497 protein lipidation; GO:0006501 C-terminal protein lipidation; GO:0006508 proteolysis; GO:0006605 protein targeting; GO:0006612 protein targeting to membrane;	GO:0005622 intracellular; GO:0005623 cell; GO:0005737 cytoplasm; GO:0005829 cytosol; GO:0044424 intracellular part; GO:0044444 cytoplasmic part; GO:0044464 cell part;	GO:0003824 catalytic activity; GO:0004175 endopeptidase activity; GO:0004197 cysteine-type endopeptidase activity; GO:0008233 peptidase activity; GO:0008234 cysteine-type peptidase activity; GO:0016787 hydrolase activity; GO:0070011			UZ	KOG2674	Cysteine protease required for autophagy - Apg4p/Aut2p
O75865	Trafficking protein particle complex subunit 6A OS=Homo sapiens OX=9606 GN=TRAPPC6A PE=1 SV=2	TRAPPC6A	1.672	0.021786671	Up	K20304	TRAPPC6, TRS33; trafficking protein particle complex subunit 6		GO:0006810 transport; GO:0006888 ER to Golgi vesicle-mediated transport; GO:0006900 membrane budding; GO:0006901 vesicle coating; GO:0006903 vesicle targeting; GO:0006996 organelle organization; GO:0009987 cellular process; GO:0016043 cellular component organizatio	GO:0005622 intracellular; GO:0005623 cell; GO:0005737 cytoplasm; GO:0005794 Golgi apparatus; GO:0005801 cis-Golgi network; GO:0005802 trans-Golgi network; GO:0005829 cytosol; GO:0012505 endomembrane system; GO:0030008 TRAPP complex; GO:00319	GO:0005085 guanyl-nucleotide exchange factor activity; GO:0005088 Ras guanyl-nucleotide exchange factor activity; GO:0005488 binding; GO:0005515 protein binding; GO:0017016 Ras GTPase binding; GO:0017112 Rab guanyl-nucleotide exchange factor activity; GO:0017137 Rab			U	KOG3316	Transport protein particle (TRAPP) complex subunit
Q9GZY0	Nuclear RNA export factor 2 OS=Homo sapiens OX=9606 GN=NXF2 PE=1 SV=1	NXF2	1.671	0.036638563	Up	K14284	NXF, TAP, MEX67; nuclear RNA export factor	map03008 Ribosome biogenesis in eukaryotes; map03013 RNA transport; map03015 mRNA surveillance pathway; map05164 Influenza A; map05168 Herpes simplex virus 1 infection	GO:0006403 RNA localization; GO:0006405 RNA export from nucleus; GO:0006406 mRNA export from nucleus; GO:0006611 protein export from nucleus; GO:0006810 transport; GO:0006886 intracellular protein transport; GO:0006913 nucleocytoplasmic	GO:0005622 intracellular; GO:0005623 cell; GO:0005634 nucleus; GO:0005654 nucleoplasm; GO:0005737 cytoplasm; GO:0005829 cytosol; GO:0031974 membrane-enclosed lumen; GO:0031981 nuclear lumen; GO:0032991 macromolecular complex; GO:00422	GO:0003676 nucleic acid binding; GO:0003723 RNA binding; GO:0005488 binding; GO:0097159 organic cyclic compound binding; GO:1901363 heterocyclic compound binding;	PF09162; PF02136; PF03943	Tap, RNA-binding; Nuclear transport factor 2 (NTF2) domain; TAP C-terminal domain	A	KOG3763	mRNA export factor TAP/MEX67
P21266	Glutathione S-transferase Mu 3 OS=Homo sapiens OX=9606 GN=GSTM3 PE=1 SV=3	GSTM3	1.669	1.15818E-05	Up	K00799	GST, gst; glutathione S-transferase [EC:2.5.1.18]	map00480 Glutathione metabolism; map00980 Metabolism of xenobiotics by cytochrome P450; map00982 Drug metabolism - cytochrome P450; map00983 Drug metabolism - other enzymes; map01100 Metabolic pathways; map01524 Platinum drug resistance; map04212 Longevity regulating pathway - worm; map05200 Pathways in cancer; map05204 Chemical carcinogenesis; map05225 Hepatocellular carcinoma; map05418 Fluid shear stress and atherosclerosis	GO:0001885 endothelial cell development; GO:0002064 epithelial cell development; GO:0003158 endothelium development; GO:0006518 peptide metabolic process; GO:0006575 cellular modified amino acid metabolic process; GO:0006725 cellular aromatic	GO:0005576 extracellular region; GO:0005622 intracellular; GO:0005623 cell; GO:0005737 cytoplasm; GO:0005829 cytosol; GO:0005929 cilium; GO:0031514 motile cilium; GO:0035686 sperm fibrous sheath; GO:0036126 sperm flagellum; GO:0042995 cell projection; GO:00432	GO:0003824 catalytic activity; GO:0004364 glutathione transferase activity; GO:0005488 binding; GO:0005515 protein binding; GO:0016740 transferase activity; GO:0016765 transferase activity, transferring alkyl or aryl (other than methyl) groups; GO:0019899 enzyme binding; GO:00332	PF02798; PF00043	Glutathione S-transferase, N-terminal domain; Glutathione S-transferase, C-terminal domain	H	KOG1695	Glutathione S-transferase
P04004	Vitronectin OS=Homo sapiens OX=9606 GN=VTN PE=1 SV=1	VTN	1.667	1.2211E-05	Up	K06251	VTN; vitronectin	map04151 PI3K-Akt signaling pathway; map04510 Focal adhesion; map04512 ECM-receptor interaction; map04610 Complement and coagulation cascades; map05165 Human papillomavirus infection; map05205 Proteoglycans in cancer	GO:0001704 formation of primary germ layer; GO:0001706 endoderm formation; GO:0001889 liver development; GO:0001932 regulation of protein phosphorylation; GO:0001934 positive regulation of protein phosphorylation; GO:0002376 immune system process; GO:0002673	GO:0005576 extracellular region; GO:0005604 basement membrane; GO:0005615 extracellular space; GO:0005622 intracellular; GO:0005623 cell; GO:0005737 cytoplasm; GO:0005783 endoplasmic reticulum; GO:0005788 endoplasmic reticulum lumen;	GO:0005102 receptor binding; GO:0005178 integrin binding; GO:0005488 binding; GO:0005515 protein binding; GO:0005518 collagen binding; GO:0005539 glycosaminoglycan binding; GO:0008201 heparin binding; GO:0042802 identical protein binding; GO:0043167 ion binding; GO:00431			OW	KOG1565	Gelatinase A and related matrix metalloproteases

Q9Y2Z2	Protein MTO1 homolog, mitochondrial OS=Homo sapiens OX=9606 GN=MTO1 PE=1 SV=2	MTO1	1.664	0.006165773	Up	K03495	gidA, mnmG, MTO1; tRNA uridine 5-carboxymethylaminomethyl modification enzyme		GO:0000959 mitochondrial RNA metabolic process; GO:0000963 mitochondrial RNA processing; GO:0001510 RNA methylation; GO:0002097 tRNA wobble base modification; GO:0002098 tRNA wobble uridine modification; GO:0006139 nucleobase-containing	GO:0005622 intracellular; GO:0005623 cell; GO:0005737 cytoplasm; GO:0005739 mitochondrion; GO:0043226 organelle; GO:0043227 membrane-bounded organelle; GO:0043229 intracellular organelle; GO:0043231 intracellular membrane-bounded	GO:0000166 nucleotide binding; GO:0005488 binding; GO:0036094 small molecule binding; GO:0043167 ion binding; GO:0043168 anion binding; GO:0048037 cofactor binding; GO:0050660 flavin adenine dinucleotide binding; GO:0050662 coenzyme binding; GO:0097159 organic cyclic	PF13932	GidA associated domain 3	J	KOG2311	NAD/FAD-utilizing protein possibly involved in translation
Q9Y343	Sorting nexin-24 OS=Homo sapiens OX=9606 GN=SNX24 PE=1 SV=1	SNX24	1.657	0.000521427	Up	K17941	SNX22_24; sorting nexin-22/24				GO:0005488 binding; GO:0005543 phospholipid binding; GO:0008289 lipid binding; GO:0010314 phosphatidylinositol-5-phosphate binding; GO:0032266 phosphatidylinositol-3-phosphate binding; GO:0035091 phosphatidylinositol binding; GO:0043167 ion binding;	PF00787	PX domain	DUZ	KOG2101	Intermediate filament-like protein, sorting nexins, and related proteins containing PX (PhoX) domain(s)
A6NCS6	Uncharacterized protein C2orf72 OS=Homo sapiens OX=9606 GN=C2orf72 PE=1 SV=2	C2orf72	1.654	0.034827493	Up											
Q9BQB6	Vitamin K epoxide reductase complex subunit 1 OS=Homo sapiens OX=9606 GN=VKORC1 PE=1 SV=1	VKORC1	1.65	0.018372779	Up	K05357	VKORC1; vitamin-K-epoxide reductase (warfarin-sensitive) [EC:1.17.4.4]	map00130 Ubiquinone and other terpenoid-quinone biosynthesis; map01100 Metabolic pathways	GO:0001501 skeletal system development; GO:0006464 cellular protein modification process; GO:0006766 vitamin metabolic process; GO:0006775 fat-soluble vitamin metabolic process; GO:0006807 nitrogen compound metabolic process; GO:0006950 response to stress; GO:0007275	GO:0005622 intracellular; GO:0005623 cell; GO:0005737 cytoplasm; GO:0005783 endoplasmic reticulum; GO:0005789 endoplasmic reticulum membrane; GO:0012505 endomembrane system; GO:0016020 membrane; GO:0031984 organelle subcompart	GO:0003824 catalytic activity; GO:0016491 oxidoreductase activity; GO:0016614 oxidoreductase activity, acting on CH-OH group of donors; GO:0016900 oxidoreductase activity, acting on the CH-OH group of donors, disulfide as acceptor; GO:0047057 vitamin-K-epoxide	PF07884	Vitamin K epoxide reductase family			
P57721	Poly(rC)-binding protein 3 OS=Homo sapiens OX=9606 GN=PCBP3 PE=1 SV=2	PCBP3	1.648	0.002108945	Up	K21444	PCBP3_4; poly(rC)-binding protein 3/4		GO:0000122 negative regulation of transcription from RNA polymerase II promoter; GO:0006139 nucleobase-containing compound metabolic process; GO:0006355 regulation of transcription, DNA-templated; GO:0006357 regulation of transcription from RNA		GO:0000981 RNA polymerase II transcription factor activity, sequence-specific DNA binding; GO:0001227 transcriptional repressor activity, RNA polymerase II transcription regulatory region sequence-specific binding; GO:0003676 nucleic acid binding;	PF00013	KH domain	A	KOG2190	PolyC-binding proteins alphaCP-1 and related KH domain proteins
P0DI82	Trafficking protein particle complex subunit 2B OS=Homo sapiens OX=9606 GN=TRAPPC2B PE=1 SV=1	TRAPPC2B	1.641	0.049757323	Up	K20301	TRAPPC2, TRS20; trafficking protein particle complex subunit 2			GO:0005622 intracellular; GO:0005623 cell; GO:0005634 nucleus; GO:0005654 nucleoplasm; GO:0005737 cytoplasm; GO:0005783 endoplasmic reticulum; GO:0012505 endomembrane system; GO:0031974 membrane-enclosed lumen; GO:00319				U	KOG3487	TRAPP 20 K subunit

Q8NC26	Zinc finger protein 114 OS=Homo sapiens OX=9606 GN=ZNF114 PE=1 SV=1	ZNF114	1.635	0.002397735	Up	K09228	KRAB; KRAB domain-containing zinc finger protein	map05168 Herpes simplex virus 1 infection	GO:0006355 regulation of transcription, DNA-templated; GO:0006357 regulation of transcription from RNA polymerase II promoter; GO:0009889 regulation of biosynthetic process; GO:0010468 regulation of gene expression; GO:0010556 regulation		GO:0000981 RNA polymerase II transcription factor activity, sequence-specific DNA binding; GO:0003700 transcription factor activity, sequence-specific DNA binding; GO:0005488 binding; GO:0005515 protein binding; GO:0042802 identical protein binding;	PF00096	Zinc finger, C2H2 type	K	KOG1721	FOG: Zn-finger
P51808	Dynein light chain Tctex-type 3 OS=Homo sapiens OX=9606 GN=DYNLT3 PE=1 SV=1	DYNLT3	1.618	0.04316413	Up	K10420	DYNLT; dynein light chain Tctex-type 1		GO:0007346 regulation of mitotic cell cycle; GO:0045787 positive regulation of cell cycle; GO:0045931 positive regulation of mitotic cell cycle; GO:0048518 positive regulation of biological process; GO:0048522 positive regulation of cellular process; GO:0050789 regulation of biological	GO:0000235 astral microtubule; GO:0000775 chromosome, centromeric region; GO:0000776 kinetochore; GO:0000777 condensed chromosome kinetochore; GO:0000779 condensed chromosome, centromeric region; GO:0000793 condensed	GO:0005488 binding; GO:0005515 protein binding; GO:0042802 identical protein binding;			N	KOG4081	Dynein light chain
Q9UPZ6	Thrombospondin type-1 domain-containing protein 7A OS=Homo sapiens OX=9606 GN=THSD7A PE=1 SV=4	THSD7A	1.609	0.001045558	Up					GO:0005623 cell; GO:0005886 plasma membrane; GO:0016020 membrane; GO:0044464 cell part; GO:0071944 cell periphery;		PF00090	Thrombospondin type 1 domain	O	KOG3538	Disintegrin metalloproteinases with thrombospondin repeats
Q13637	Ras-related protein Rab-32 OS=Homo sapiens OX=9606 GN=RAB32 PE=1 SV=3	RAB32	1.604	0.033621517	Up	K07918	RAB32; Ras-related protein Rab-32		GO:0002376 immune system process; GO:0006810 transport; GO:0006996 organelle organization; GO:0007005 mitochondrion organization; GO:0008104 protein localization; GO:0009605 response to external stimulus; GO:0009987 cellular process; GO:00160	GO:0005622 intracellular; GO:0005623 cell; GO:0005737 cytoplasm; GO:0005739 mitochondrion; GO:0005740 mitochondrial envelope; GO:0005741 mitochondrial outer membrane; GO:0005768 endosome; GO:0005769 early endosome; GO:0005783	GO:0000166 nucleotide binding; GO:0001882 nucleoside binding; GO:0001883 purine nucleoside binding; GO:0003824 catalytic activity; GO:0003924 GTPase activity; GO:0005488 binding; GO:0005515 protein binding; GO:0005525 GTP binding; GO:0016462 pyrophosphatase activity;	PF00071	Ras family	U	KOG4423	GTP-binding protein-like, RAS superfamily
Q9BQ48	39S ribosomal protein L34, mitochondrial OS=Homo sapiens OX=9606 GN=MRPL34 PE=1 SV=1	MRPL34	1.59	0.038731945	Up	K02914	RP-L34, MRPL34, rpmH; large subunit ribosomal protein L34	map03010 Ribosome	GO:0006412 translation; GO:0006414 translational elongation; GO:0006415 translational termination; GO:0006518 peptide metabolic process; GO:0006807 nitrogen compound metabolic process; GO:0008152 metabolic process; GO:0009058 biosynthetic process;	GO:0000313 organellar ribosome; GO:0000315 organellar large ribosomal subunit; GO:0005622 intracellular; GO:0005623 cell; GO:0005737 cytoplasm; GO:0005739 mitochondrion; GO:0005740 mitochondrial envelope; GO:0005743 mitochondr	GO:0003735 structural constituent of ribosome; GO:0005198 structural molecule activity;			J	KOG4612	Mitochondrial ribosomal protein L34

P17900	Ganglioside GM2 activator OS=Homo sapiens OX=9606 GN=GM2A PE=1 SV=4	GM2A	1.589	1.17781E-05	Up	K12383	GM2A; ganglioside GM2 activator	map04142 Lysosome	GO:0001573 ganglioside metabolic process; GO:0001775 cell activation; GO:0002252 immune effector process; GO:0002263 cell activation involved in immune response; GO:0002274 myeloid leukocyte activation; GO:0002275 myeloid cell activation involved in immune response; GO:000283	GO:0000323 lytic vacuole; GO:0005576 extracellular region; GO:0005622 intracellular; GO:0005623 cell; GO:0005737 cytoplasm; GO:0005764 lysosome; GO:0005766 primary lysosome; GO:0005773 vacuole; GO:0005775 vacuolar lumen; GO:0012505 endomemb	GO:0003824 catalytic activity; GO:0004553 hydrolase activity, hydrolyzing O-glycosyl compounds; GO:0004563 beta-N-acetylhexosaminidase activity; GO:0008047 enzyme activator activity; GO:0015929 hexosaminidase activity; GO:0016787 hydrolase activity; GO:00167	PF02221	ML domain			
O43913	Origin recognition complex subunit 5 OS=Homo sapiens OX=9606 GN=ORC5 PE=1 SV=1	ORC5	1.585	0.038829366	Up	K02607	ORC5; origin recognition complex subunit 5	map04110 Cell cycle	GO:0000082 G1/S transition of mitotic cell cycle; GO:0000278 mitotic cell cycle; GO:0006139 nucleobase-containing compound metabolic process; GO:0006259 DNA metabolic process; GO:0006260 DNA replication; GO:0006261 DNA-dependent DNA replication; GO:0006270 DNA replication initiation;	GO:0000228 nuclear chromosome; GO:0000808 origin recognition complex; GO:0005622 intracellular; GO:0005623 cell; GO:0005634 nucleus; GO:0005654 nucleoplasm; GO:0005664 nuclear origin of replication recognition complex; GO:0005694 chromosome; GO:00057	GO:0000166 nucleotide binding; GO:0003676 nucleic acid binding; GO:0003677 DNA binding; GO:0003688 DNA replication origin binding; GO:0003690 double-stranded DNA binding; GO:0005488 binding; GO:0036094 small molecule binding; GO:0043565 sequence-specific	PF13191	AAA ATPase domain	L	KOG2543	Origin recognition complex, subunit 5
P40126	L-dopachrome tautomerase OS=Homo sapiens OX=9606 GN=DCT PE=1 SV=1	DCT	1.574	0.012956128	Up	K01827	DCT; dopachrome tautomerase [EC:5.3.3.12]	map00350 Tyrosine metabolism; map01100 Metabolic pathways; map04916 Melanogenesis	GO:0002052 positive regulation of neuroblast proliferation; GO:0006082 organic acid metabolic process; GO:0006520 cellular amino acid metabolic process; GO:0006570 tyrosine metabolic process; GO:0006582 melanin metabolic process; GO:0006583 melanin biosynthetic process from tyrosine;	GO:0005622 intracellular; GO:0005623 cell; GO:0005737 cytoplasm; GO:0005829 cytosol; GO:0012506 vesicle membrane; GO:0016020 membrane; GO:0016021 integral component of membrane; GO:0030659 cytoplasmic vesicle membrane; GO:0031090 organelle membrane;	GO:0003824 catalytic activity; GO:0004167 dopachrome isomerase activity; GO:0005488 binding; GO:0005507 copper ion binding; GO:0016491 oxidoreductase activity; GO:0016853 isomerase activity; GO:0016860 intramolecular oxidoreductase activity; GO:00168	PF00264	Common central domain of tyrosinase			
Q9H7B4	Histone-lysine N-methyltransferase SMYD3 OS=Homo sapiens OX=9606 GN=SMYD3 PE=1 SV=4	SMYD3	1.572	0.002732915	Up	K11426	SMYD; [histone H3]-lysine4/36 N-trimethyltransferase SMYD [EC:2.1.1.354 2.1.1.357]	map00310 Lysine degradation; map01100 Metabolic pathways	GO:0006325 chromatin organization; GO:0006464 cellular protein modification process; GO:0006479 protein methylation; GO:0006807 nitrogen compound metabolic process; GO:0006996 organelle organization; GO:0008152 metabolic process; GO:0008213 protein alkylation;	GO:0005622 intracellular; GO:0005623 cell; GO:0005634 nucleus; GO:0005654 nucleoplasm; GO:0005737 cytoplasm; GO:0005829 cytosol; GO:0031974 membrane-enclosed lumen; GO:0031981 nuclear lumen; GO:0043226 organelle; GO:0043227 membrane-	GO:0003824 catalytic activity; GO:0008168 methyltransferase activity; GO:0008170 N-methyltransferase activity; GO:0008276 protein methyltransferase activity; GO:0008757 S-adenosylmethionine-dependent methyltransferase activity; GO:0016278 lysine N-methyltransferase	PF01753	MYND finger	B	KOG2084	Predicted histone tail methylase containing SET domain
Q5T8I3	Protein FAM102B OS=Homo sapiens OX=9606 GN=FAM102B PE=1 SV=2	FAM102B	1.563	0.004598074	Up											
Q86UY6	N-alpha-acetyltransferase 40 OS=Homo sapiens OX=9606 GN=NAA40 PE=1 SV=1	NAA40	1.554	0.008563664	Up	K20794	NAA40, NAT4; N-alpha-acetyltransferase 40 [EC:2.3.1.257]		GO:0006325 chromatin organization; GO:0006464 cellular protein modification process; GO:0006473 protein acetylation; GO:0006474 N-terminal protein amino acid acetylation; GO:0006475 internal protein amino acid acetylation; GO:0006629 lipid metabolic process; GO:0006807 nitrogen compound		GO:0003824 catalytic activity; GO:0004402 histone acetyltransferase activity; GO:0004596 peptide alpha-N-acetyltransferase activity; GO:0008080 N-acetyltransferase activity; GO:0010485 H4 histone acetyltransferase activity; GO:0016407 acetyltransferase activity; GO:00164			S	KOG2488	Acetyltransferase (GNAT) domain-containing protein

Q4L180	Filamin A-interacting protein 1-like OS=Homo sapiens OX=9606 GN=FILIP1L PE=1 SV=2	FILIP1L	1.554	0.020416358	Up									S	KOG1103	Predicted coiled-coil protein
Q9H5I1	Histone-lysine N-methyltransferase SUV39H2 OS=Homo sapiens OX=9606 GN=SUV39H2 PE=1 SV=2	SUV39H2	1.547	0.007550847	Up	K11419	SUV39H, CLR4; [histone H3]-lysine9 N-trimethyltransferase SUV39H [EC:2.1.1.355]	map00310 Lysine degradation; map01100 Metabolic pathways	GO:0000003 reproduction; GO:0000122 negative regulation of transcription from RNA polymerase II promoter; GO:0000280 nuclear division; GO:0001666 response to hypoxia; GO:0006325 chromatin organization; GO:0006333 chromatin assembly or	GO:0000228 nuclear chromosome; GO:0000775 chromosome, centromeric region; GO:0000785 chromatin; GO:0000790 nuclear chromatin; GO:0000792 heterochromatin; GO:0005622 intracellular; GO:0005623 cell; GO:0005634 nucleus; GO:0005654 nucleoplas	GO:0000976 transcription regulatory region sequence-specific DNA binding; GO:0000977 RNA polymerase II regulatory region sequence-specific DNA binding; GO:0001012 RNA polymerase II regulatory region DNA binding; GO:0001067 regulatory	PF00385	Chromo (CHRromatin Organisation MOdifier) domain	B	KOG1082	Histone H3 (Lys9) methyltransferase SUV39H1/Clr4, required for transcriptional silencing
Q9P2X0	Dolichol-phosphate mannosyltransferase subunit 3 OS=Homo sapiens OX=9606 GN=DPM3 PE=1 SV=2	DPM3	1.54	7.50355E-05	Up	K09659	DPM3; dolichol-phosphate mannosyltransferase subunit 3	map00510 N-Glycan biosynthesis; map01100 Metabolic pathways	GO:0005975 carbohydrate metabolic process; GO:0006464 cellular protein modification process; GO:0006486 protein glycosylation; GO:0006493 protein O-linked glycosylation; GO:0006497 protein lipidation; GO:0006505 GPI anchor metabolic process; GO:0006506 GPI anchor	GO:0005622 intracellular; GO:0005623 cell; GO:0005737 cytoplasm; GO:0005783 endoplasmic reticulum; GO:0005789 endoplasmic reticulum membrane; GO:0012505 endomembrane system; GO:0016020 membrane; GO:0016021 integral component of				OT	KOG4841	Dolichol-phosphate mannosyltransferase, subunit 3
Q9HCE9	Anoctamin-8 OS=Homo sapiens OX=9606 GN=ANO8 PE=1 SV=3	ANO8	1.535	0.028863369	Up	K19502	ANO8, TMEM16H; anoctamin-8		GO:0006464 cellular protein modification process; GO:0006807 nitrogen compound metabolic process; GO:0006810 transport; GO:0006811 ion transport; GO:0006820 anion transport; GO:0006821 chloride transport; GO:0008152 metabolic process; GO:0009987 cellular process; GO:0015698	GO:0005622 intracellular; GO:0005623 cell; GO:0005737 cytoplasm; GO:0005783 endoplasmic reticulum; GO:0005788 endoplasmic reticulum lumen; GO:0005886 plasma membrane; GO:0012505 endomembrane system; GO:0016020 membrane; GO:00319	GO:0005215 transporter activity; GO:0005216 ion channel activity; GO:0005229 intracellular calcium activated chloride channel activity; GO:0005253 anion channel activity; GO:0005254 chloride channel activity; GO:0008509 anion transmembrane transporter activity; GO:00150			D	KOG2513	Protein required for meiotic chromosome segregation
O14867	Transcription regulator protein BACH1 OS=Homo sapiens OX=9606 GN=BACH1 PE=1 SV=2	BACH1	1.533	0.031300795	Up	K09042	BACH; transcription regulator BACH		GO:0000082 G1/S transition of mitotic cell cycle; GO:0000083 regulation of transcription involved in G1/S transition of mitotic cell cycle; GO:0000086 G2/M transition of mitotic cell cycle; GO:0000117 regulation of transcription involved in G2/M transition of mitotic cell cycle; GO:00001	GO:0000151 ubiquitin ligase complex; GO:0005622 intracellular; GO:0005623 cell; GO:0005634 nucleus; GO:0031461 cullin-RING ubiquitin ligase complex; GO:0031463 Cul3-RING ubiquitin ligase complex; GO:0032991 macromolecular complex; GO:00432	GO:0000976 transcription regulatory region sequence-specific DNA binding; GO:0000977 RNA polymerase II regulatory region sequence-specific DNA binding; GO:0000980 RNA polymerase II distal enhancer sequence-specific DNA binding; GO:0000981 RNA	PF00651	BTB/POZ domain	K	KOG3863	bZIP transcription factor NRF1
Q92570	Nuclear receptor subfamily 4 group A member 3 OS=Homo sapiens OX=9606 GN=NR4A3 PE=1 SV=3	NR4A3	1.527	0.03365906	Up	K08559	NR4A3, NOR1; nuclear receptor subfamily 4 group A member 3	map05202 Transcriptional misregulation in cancer	GO:0000122 negative regulation of transcription from RNA polymerase II promoter; GO:0000302 response to reactive oxygen species; GO:0000902 cell morphogenesis; GO:0000904 cell morphogenesis involved in differentiation; GO:0001704 formation of primary	GO:0005622 intracellular; GO:0005623 cell; GO:0005634 nucleus; GO:0005654 nucleoplasm; GO:0005667 transcription factor complex; GO:0031974 membrane-enclosed lumen; GO:0031981 nuclear lumen; GO:0032991 macromolecular complex; GO:00432	GO:0000976 transcription regulatory region sequence-specific DNA binding; GO:0000977 RNA polymerase II regulatory region sequence-specific DNA binding; GO:0000978 RNA polymerase II core promoter proximal region sequence-specific DNA binding;	PF00105; PF00104	Zinc finger, C4 type (two domains); Ligand-binding domain of nuclear hormone receptor	K	KOG4217	Nuclear receptors of the nerve growth factor-induced protein B type

P30825	High affinity cationic amino acid transporter 1 OS=Homo sapiens OX=9606 GN=SLC7A1 PE=1 SV=1	SLC7A1	1.521	0.039255935	Up	K13863	SLC7A1, ATRC1; solute carrier family 7 (cationic amino acid transporter), member 1	map05206 MicroRNAs in cancer	GO:0003333 amino acid transmembrane transport; GO:0006810 transport; GO:0006811 ion transport; GO:0006812 cation transport; GO:0006820 anion transport; GO:0006865 amino acid transport; GO:0009966 regulation of signal transduction; GO:0010646 regulation	GO:0005623 cell; GO:0005886 plasma membrane; GO:0005887 integral component of plasma membrane; GO:0016020 membrane; GO:0016021 integral component of membrane; GO:0031224 intrinsic component of membrane; GO:0031226 intrinsic component of plasma membrane; GO:0044425 membrane	GO:0005215 transporter activity; GO:0005342 organic acid transmembrane transporter activity; GO:0008324 cation transmembrane transporter activity; GO:0008509 anion transmembrane transporter activity; GO:0008514 organic anion transmembrane transporter activity; GO:00150	PF13906	C-terminus of AA_permease	P	KOG1286	Amino acid transporters
P04350	Tubulin beta-4A chain OS=Homo sapiens OX=9606 GN=TUBB4A PE=1 SV=2	TUBB4A	1.518	0.011939219	Up	K07375	TUBB; tubulin beta	map04145 Phagosome; map04540 Gap junction; map05130 Pathogenic Escherichia coli infection	GO:0000086 G2/M transition of mitotic cell cycle; GO:0000278 mitotic cell cycle; GO:0006996 organelle organization; GO:0007049 cell cycle; GO:0007346 regulation of mitotic cell cycle; GO:0009987 cellular process; GO:0010389 regulation of G2/M transition of mitotic cell cycle;	GO:0005622 intracellular; GO:0005623 cell; GO:0005737 cytoplasm; GO:0005829 cytosol; GO:0005856 cytoskeleton; GO:0005874 microtubule; GO:0005929 cilium; GO:0005930 axoneme; GO:0015630 microtubule cytoskeleton; GO:00304	GO:0005488 binding; GO:0005509 calcium ion binding; GO:0043167 ion binding; GO:0043169 cation binding; GO:0046872 metal ion binding;	PF00091; PF03953	Tubulin/FtsZ family, GTPase domain; Tubulin C-terminal domain	Z	KOG1375	Beta tubulin
P00749	Urokinase-type plasminogen activator OS=Homo sapiens OX=9606 GN=PLAU PE=1 SV=2	PLAU	1.517	0.00543974	Up	K01348	PLAU; urokinase plasminogen activator [EC:3.4.21.73]	map04064 NF-kappa B signaling pathway; map04610 Complement and coagulation cascades; map05202 Transcriptional misregulation in cancer; map05205 Proteoglycans in cancer; map05206 MicroRNAs in cancer; map05215 Prostate cancer	GO:0000003 reproduction; GO:0001525 angiogenesis; GO:0001568 blood vessel development; GO:0001666 response to hypoxia; GO:0001678 cellular glucose homeostasis; GO:0001775 cell activation; GO:0001944 vasculature development; GO:00019	GO:0005576 extracellular region; GO:0005615 extracellular space; GO:0005622 intracellular; GO:0005623 cell; GO:0005737 cytoplasm; GO:0005886 plasma membrane; GO:0009986 cell surface; GO:0012505 endomembrane system; GO:0012506 vesicle membrane;	GO:0003824 catalytic activity; GO:0004175 endopeptidase activity; GO:0004252 serine-type endopeptidase activity; GO:0008233 peptidase activity; GO:0008236 serine-type peptidase activity; GO:0016787 hydrolase activity; GO:0017171 serine hydrolase activity;	PF00051; PF00089	Kringle domain; Trypsin			
Q92994	Transcription factor IIIB 90 kDa subunit OS=Homo sapiens OX=9606 GN=BRF1 PE=1 SV=1	BRF1	1.516	0.037824117	Up	K15196	BRF1, GTF3B; transcription factor IIIB 90 kDa subunit		GO:0006139 nucleobase-containing compound metabolic process; GO:0006351 transcription, DNA-templated; GO:0006352 DNA-templated transcription, initiation; GO:0006383 transcription from RNA polymerase III promoter; GO:0006384 transcription initiation from RNA	GO:0000126 transcription factor TFIIIB complex; GO:0005622 intracellular; GO:0005623 cell; GO:0005634 nucleus; GO:0005654 nucleoplasm; GO:0005667 transcription factor complex; GO:0031974 membrane-enclosed lumen; GO:0031981 nuclear lumen;		PF08271; PF00382	TFIIB zinc-binding; Transcription factor TFIIB repeat	K	KOG1598	Transcription initiation factor TFIIIB, Brf1 subunit
Q9BUB4	tRNA-specific adenosine deaminase 1 OS=Homo sapiens OX=9606 GN=ADAT1 PE=1 SV=1	ADAT1	1.515	0.00246342	Up	K15440	TAD1, ADAT1; tRNA-specific adenosine deaminase 1 [EC:3.5.4.34]		GO:0006139 nucleobase-containing compound metabolic process; GO:0006396 RNA processing; GO:0006399 tRNA metabolic process; GO:0006725 cellular aromatic compound metabolic process; GO:0006807 nitrogen compound metabolic process; GO:0008033 tRNA processing; GO:0008152 metabolic		GO:0003676 nucleic acid binding; GO:0003723 RNA binding; GO:0003824 catalytic activity; GO:0004000 adenosine deaminase activity; GO:0005488 binding; GO:0008251 tRNA-specific adenosine deaminase activity; GO:0016787 hydrolase activity; GO:0016810 hydrolase activity,			A	KOG2777	tRNA-specific adenosine deaminase 1

P60891	Ribose-phosphate pyrophosphokinase 1 OS=Homo sapiens OX=9606 GN=PRPS1 PE=1 SV=2	PRPS1	1.514	0.003576064	Up	K00948	PRPS, prsA; ribose-phosphate pyrophosphokinase [EC:2.7.6.1]	map00030 Pentose phosphate pathway; map00230 Purine metabolism; map01100 Metabolic pathways; map01200 Carbon metabolism; map01230 Biosynthesis of amino acids	GO:0006015 5-phosphoribose 1-diphosphate biosynthetic process; GO:0006082 organic acid metabolic process; GO:0006139 nucleobase-containing compound metabolic process; GO:0006144 purine nucleobase metabolic process; GO:0006163 purine nucleotide metabolic process; GO:00061	GO:0002189 ribose phosphate diphosphokinase complex; GO:0005622 intracellular; GO:0005623 cell; GO:0005737 cytoplasm; GO:0005829 cytosol; GO:0032991 macromolecular complex; GO:0044424 intracellular part; GO:0044444 cytoplasmic part; GO:00444	GO:0000166 nucleotide binding; GO:0000287 magnesium ion binding; GO:0001882 nucleoside binding; GO:0001883 purine nucleoside binding; GO:0003824 catalytic activity; GO:0004749 ribose phosphate diphosphokinase activity; GO:0005488 binding; GO:0005515 protein binding;	PF13793	N-terminal domain of ribose phosphate pyrophosphokinase	F	KOG1448	Ribose-phosphate pyrophosphokinase
Q8NBQ5	Estradiol 17-beta-dehydrogenase 11 OS=Homo sapiens OX=9606 GN=HSD17B11 PE=1 SV=3	HSD17B11	1.513	0.04248384	Up				GO:0006629 lipid metabolic process; GO:0006694 steroid biosynthetic process; GO:0006703 estrogen biosynthetic process; GO:0006706 steroid catabolic process; GO:0006710 androgen catabolic process; GO:0008152 metabolic process; GO:0008202 steroid metabolic process; GO:0008209	GO:0005622 intracellular; GO:0005623 cell; GO:0005737 cytoplasm; GO:0005811 lipid particle; GO:0005829 cytosol; GO:0043226 organelle; GO:0043228 non-membrane-bounded organelle; GO:0043229 intracellular organelle; GO:0043232 intracellular non-membrane-	GO:0003824 catalytic activity; GO:0004303 estradiol 17-beta-dehydrogenase activity; GO:0016229 steroid dehydrogenase activity; GO:0016491 oxidoreductase activity; GO:0016614 oxidoreductase activity, acting on CH-OH group of donors; GO:0016616	PF00106	short chain dehydrogenase	Q	KOG1201	Hydroxysteroid 17-beta dehydrogenase 11
Q93096	Protein tyrosine phosphatase type IVA 1 OS=Homo sapiens OX=9606 GN=PTP4A1 PE=1 SV=2	PTP4A1	1.509	0.003653568	Up	K18041	PTP4A; protein tyrosine phosphatase type IVA [EC:3.1.3.48]		GO:0006464 cellular protein modification process; GO:0006470 protein dephosphorylation; GO:0006793 phosphorus metabolic process; GO:0006796 phosphate-containing compound metabolic process; GO:0006807 nitrogen compound metabolic process; GO:0008152 metabolic process; GO:00099	GO:0005622 intracellular; GO:0005623 cell; GO:0005634 nucleus; GO:0005737 cytoplasm; GO:0005768 endosome; GO:0005769 early endosome; GO:0005783 endoplasmic reticulum; GO:0005819 spindle; GO:0005856 cytoskeleton; GO:0005886 plasma membrane;	GO:0003824 catalytic activity; GO:0004721 phosphoprotein phosphatase activity; GO:0004725 protein tyrosine phosphatase activity; GO:0004727 prenylated protein tyrosine phosphatase activity; GO:0016787 hydrolase activity; GO:0016788 hydrolase activity, acting on ester	PF00782	Dual specificity phosphatase, catalytic domain	T	KOG2836	Protein tyrosine phosphatase IVA1
Q9BTT0	Acidic leucine-rich nuclear phosphoprotein 32 family member E OS=Homo sapiens OX=9606 GN=ANP32E PE=1 SV=1	ANP32E	1.507	0.002793367	Up	K18648	ANP32E; acidic leucine-rich nuclear phosphoprotein 32 family member E		GO:0006323 DNA packaging; GO:0006325 chromatin organization; GO:0006333 chromatin assembly or disassembly; GO:0006334 nucleosome assembly; GO:0006338 chromatin remodeling; GO:0006810 transport; GO:0006913 nucleocytoplasmic	GO:0000228 nuclear chromosome; GO:0000785 chromatin; GO:0000790 nuclear chromatin; GO:0000812 Swr1 complex; GO:0005622 intracellular; GO:0005623 cell; GO:0005634 nucleus; GO:0005694 chromosome; GO:0005737 cytoplasm; GO:0031410 cytoplasmi	GO:0004857 enzyme inhibitor activity; GO:0005488 binding; GO:0005515 protein binding; GO:0019208 phosphatase regulator activity; GO:0019212 phosphatase inhibitor activity; GO:0030234 enzyme regulator activity; GO:0042393 histone binding; GO:0098772 molecular function regulator;			D	KOG2739	Leucine-rich acidic nuclear protein
Q9H7F0	Probable cation-transporting ATPase 13A3 OS=Homo sapiens OX=9606 GN=ATP13A3 PE=1 SV=4	ATP13A3	1.506	0.017192996	Up	K14951	ATP13A3_4_5; cation-transporting P-type ATPase 13A3/4/5 [EC:7.2.2.-]		GO:0006810 transport; GO:0006811 ion transport; GO:0006812 cation transport; GO:0006816 calcium ion transport; GO:0006873 cellular ion homeostasis; GO:0006874 cellular calcium ion homeostasis; GO:0006875 cellular metal ion homeostasis; GO:0009987 cellular process;	GO:0005622 intracellular; GO:0005623 cell; GO:0005886 plasma membrane; GO:0005887 integral component of plasma membrane; GO:0016020 membrane; GO:0016021 integral component of membrane; GO:0031224 intrinsic component of membrane; GO:0031226 intrinsic component of plasma	GO:0003824 catalytic activity; GO:0005215 transporter activity; GO:0005388 calcium-transporting ATPase activity; GO:0008324 cation transmembrane transporter activity; GO:0015075 ion transmembrane transporter activity; GO:0015085 calcium ion transmembrane transporter	PF00690; PF00702	Cation transporter/ATPase, N-terminus; haloacid dehalogenase-like hydrolase	P	KOG0208	Cation transport ATPase

Q17RY0	Cytoplasmic polyadenylation element-binding protein 4 OS=Homo sapiens OX=9606 GN=CPEB4 PE=1 SV=1	CPEB4	1.505	0.003683037	Up	K02602	CPEB, ORB; cytoplasmic polyadenylation element-binding protein	map04114 Oocyte meiosis; map04320 Dorso-ventral axis formation; map04914 Progesterone-mediated oocyte maturation	GO:0001101 response to acid chemical; GO:0002931 response to ischemia; GO:0006412 translation; GO:0006417 regulation of translation; GO:0006518 peptide metabolic process; GO:0006807 nitrogen compound metabolic process; GO:0006950 response to stress; GO:00071	GO:0005622 intracellular; GO:0005623 cell; GO:0005634 nucleus; GO:0005737 cytoplasm; GO:0005783 endoplasmic reticulum; GO:0012505 endomembrane system; GO:0014069 postsynaptic density; GO:0030425 dendrite; GO:0032279 asymmetric	GO:0000900 translation repressor activity, nucleic acid binding; GO:0003676 nucleic acid binding; GO:0003723 RNA binding; GO:0003729 mRNA binding; GO:0003730 mRNA 3'-UTR binding; GO:0005488 binding; GO:0008135 translation factor activity, RNA binding;			J	KOG0129	Predicted RNA-binding protein (RRM superfamily)
Q5K4L6	Solute carrier family 27 member 3 OS=Homo sapiens OX=9606 GN=SLC27A3 PE=1 SV=4	SLC27A3	1.504	0.000644631	Up	K08772	SLC27A3, FATP3; solute carrier family 27 (fatty acid transporter), member 3 [EC:6.2.1.-]	map04931 Insulin resistance	GO:0001676 long-chain fatty acid metabolic process; GO:0006082 organic acid metabolic process; GO:0006139 nucleobase-containing compound metabolic process; GO:0006163 purine nucleotide metabolic process; GO:0006629 lipid metabolic process; GO:0006631 fatty acid metabolic	GO:0005622 intracellular; GO:0005623 cell; GO:0005737 cytoplasm; GO:0005739 mitochondrion; GO:0005759 mitochondrial matrix; GO:0005783 endoplasmic reticulum; GO:0012505 endomembrane system; GO:0031974 membrane-enclosed	GO:0003824 catalytic activity; GO:0004321 fatty-acyl-CoA synthase activity; GO:0004467 long-chain fatty acid-CoA ligase activity; GO:0015645 fatty acid ligase activity; GO:0016405 CoA-ligase activity; GO:0016408 C-acyltransferase activity; GO:0016740 transferase activity;	PF13193	Domain of unknown function (DUF4009)	I	KOG1179	Very long-chain acyl-CoA synthetase/fatty acid transporter
Q9H857	5'-nucleotidase domain-containing protein 2 OS=Homo sapiens OX=9606 GN=NT5DC2 PE=1 SV=1	NT5DC2	1.504	0.015840874	Up				GO:0006793 phosphorus metabolic process; GO:0006796 phosphate-containing compound metabolic process; GO:0008152 metabolic process; GO:0009987 cellular process; GO:0016311 dephosphorylation; GO:0044237 cellular metabolic process;		GO:0003824 catalytic activity; GO:0008252 nucleotidase activity; GO:0008253 5'-nucleotidase activity; GO:0016787 hydrolase activity; GO:0016788 hydrolase activity, acting on ester bonds; GO:0016791 phosphatase activity; GO:0042578 phosphoric ester hydrolase			F	KOG2470	Similar to IMP-GMP specific 5'-nucleotidase
Q96DX4	RING finger and SPRY domain-containing protein 1 OS=Homo sapiens OX=9606 GN=RSPRY1 PE=1 SV=1	RSPRY1	1.503	0.003841033	Up	K23332	RSPRY1; RING finger and SPRY domain-containing protein 1					PF13920	Zinc finger, C3HC4 type (RING finger)	O	KOG2242	Scaffold/matrix specific factor hnRNP-U/SAF-A, contains SPRY domain
A6NHX0	Cytosolic arginine sensor for mTORC1 subunit 2 OS=Homo sapiens OX=9606 GN=CASTOR2 PE=1 SV=3	CASTOR2	1.497	0.038270039	Up	K23081	CASTOR2; cytosolic arginine sensor for mTORC1 subunit 2	map04150 mTOR signaling pathway	GO:0009966 regulation of signal transduction; GO:0009968 negative regulation of signal transduction; GO:0010646 regulation of cell communication; GO:0010648 negative regulation of cell communication; GO:0023051 regulation of signaling; GO:0023057 negative	GO:0005622 intracellular; GO:0005623 cell; GO:0005737 cytoplasm; GO:0005829 cytosol; GO:0044424 intracellular part; GO:0044444 cytoplasmic part; GO:0044464 cell part;	GO:0005488 binding; GO:0005515 protein binding; GO:0042802 identical protein binding;	PF13840	Family description			

Q9H9A5	CCR4-NOT transcription complex subunit 10 OS=Homo sapiens OX=9606 GN=CNOT10 PE=1 SV=1	CNOT10	1.494	0.044307025	Up	K12607	CNOT10; CCR4-NOT transcription complex subunit 10	map03018 RNA degradation	GO:0000075 cell cycle checkpoint; GO:0000077 DNA damage checkpoint; GO:0000278 mitotic cell cycle; GO:0000288 nuclear-transcribed mRNA catabolic process, deadenylation-dependent decay; GO:0000289 nuclear-transcribed mRNA poly(A) tail shortening; GO:0000956 nuclear-transcribed mRNA	GO:0005622 intracellular; GO:0005623 cell; GO:0005737 cytoplasm; GO:0005829 cytosol; GO:0030014 CCR4-NOT complex; GO:0032991 macromolecular complex; GO:0044424 intracellular part; GO:0044444 cytoplasmic part; GO:0044464 cell part;				K	KOG2471	TPR repeat-containing protein
Q9BXW9	Fanconi anemia group D2 protein OS=Homo sapiens OX=9606 GN=FANCD2 PE=1 SV=2	FANCD2	1.491	0.041383642	Up	K10891	FANCD2; fanconi anemia group D2 protein	map03460 Fanconi anemia pathway	GO:0000003 reproduction; GO:0000280 nuclear division; GO:0002682 regulation of immune system process; GO:0002694 regulation of leukocyte activation; GO:0006139 nucleobase-containing compound metabolic process; GO:0006259 DNA metabolic process; GO:00062	GO:0000793 condensed chromosome; GO:0005622 intracellular; GO:0005623 cell; GO:0005634 nucleus; GO:0005654 nucleoplasm; GO:0005694 chromosome; GO:0005730 nucleolus; GO:0005737 cytoplasm; GO:0005829 cytosol; GO:0016604 nuclear	GO:0005488 binding; GO:0005515 protein binding; GO:0019899 enzyme binding; GO:0070182 DNA polymerase binding;			S	KOG4712	Uncharacterized conserved protein
Q9H8H3	Methyltransferase-like protein 7A OS=Homo sapiens OX=9606 GN=METTL7A PE=1 SV=1	METTL7A	1.49	0.012091771	Up				GO:0001775 cell activation; GO:0002252 immune effector process; GO:0002263 cell activation involved in immune response; GO:0002274 myeloid leukocyte activation; GO:0002275 myeloid cell activation involved in immune response; GO:0002283 neutrophil activation involved in immune response;	GO:0005576 extracellular region; GO:0005622 intracellular; GO:0005623 cell; GO:0005737 cytoplasm; GO:0005811 lipid particle; GO:0012505 endomembrane system; GO:0030141 secretory granule; GO:0031410 cytoplasmic vesicle; GO:0031974		PF08241	Methyltransferase domain	Q	KOG4300	Predicted methyltransferase
Q5MIZ7	Serine/threonine-protein phosphatase 4 regulatory subunit 3B OS=Homo sapiens OX=9606 GN=PPP4R3B PE=1 SV=2	PPP4R3B	1.488	0.036812237	Up	K17491	SMEK, PPP4R3; protein phosphatase 4 regulatory subunit 3	map04212 Longevity regulating pathway - worm; map04922 Glucagon signaling pathway		GO:0005622 intracellular; GO:0005623 cell; GO:0005634 nucleus; GO:0005654 nucleoplasm; GO:0005813 centrosome; GO:0005815 microtubule organizing center; GO:0005856 cytoskeleton; GO:0015630 microtubule cytoskeleto				G	KOG2175	Protein predicted to be involved in carbohydrate metabolism
Q96BI1	Solute carrier family 22 member 18 OS=Homo sapiens OX=9606 GN=SLC22A18 PE=1 SV=3	SLC22A18	1.484	0.048828519	Up	K08214	SLC22A18; MFS transporter, OCT family, solute carrier family 22 (organic cation transporter), member 18		GO:0003008 system process; GO:0006810 transport; GO:0006811 ion transport; GO:0006812 cation transport; GO:0006820 anion transport; GO:0006855 drug transmembrane transport; GO:0007588 excretion; GO:0015672 monovalent inorganic cation transport; GO:0015695 organic	GO:0005622 intracellular; GO:0005623 cell; GO:0005634 nucleus; GO:0005635 nuclear envelope; GO:0005737 cytoplasm; GO:0005886 plasma membrane; GO:0005887 integral component of plasma membrane; GO:0012505 endomembrane system; GO:0016020 membrane; GO:00160	GO:0005215 transporter activity; GO:0005488 binding; GO:0005515 protein binding; GO:0008324 cation transmembrane transporter activity; GO:0008509 anion transmembrane transporter activity; GO:0008514 organic anion transmembrane transporter activity; GO:0015075 ion transmemb					

P25815	Protein S100-P OS=Homo sapiens OX=9606 GN=S100P PE=1 SV=2	S100P	1.484	0.026662821	Up	K23771			GO:0001667 ameboidal-type cell migration; GO:0001775 cell activation; GO:0002252 immune effector process; GO:0002263 cell activation involved in immune response; GO:0002274 myeloid leukocyte activation; GO:0002275 myeloid cell activation involved in immune response; GO:000228 3	GO:0005576 extracellular region; GO:0005622 intracellular; GO:0005623 cell; GO:0005634 nucleus; GO:0005654 nucleoplasm; GO:0005737 cytoplasm; GO:0005886 plasma membrane; GO:0005902 microvillus; GO:0012505 endomembrane system;	GO:0000287 magnesium ion binding; GO:0005488 binding; GO:0005509 calcium ion binding; GO:0005515 protein binding; GO:0042802 identical protein binding; GO:0042803 protein homodimerization activity; GO:0043167 ion binding; GO:0043169 cation binding; GO:00468	PF01023	S-100/ICaBP type calcium binding domain			
P07919	Cytochrome b-c1 complex subunit 6, mitochondrial OS=Homo sapiens OX=9606 GN=UQCRH PE=1 SV=2	UQCRH	1.48	0.015357019	Up	K00416	QCR6, UQCRH; ubiquinol-cytochrome c reductase subunit 6	map00190 Oxidative phosphorylation; map01100 Metabolic pathways; map04260 Cardiac muscle contraction; map04714 Thermogenesis; map04932 Non-alcoholic fatty liver disease (NAFLD); map05010 Alzheimer disease; map05012 Parkinson disease; map05016 Huntington disease	GO:0006091 generation of precursor metabolites and energy; GO:0006119 oxidative phosphorylation; GO:0006122 mitochondrial electron transport, ubiquinol to cytochrome c; GO:0006139 nucleobase-containing compound metabolic process; GO:0006163 purine nucleotide	GO:0005622 intracellular; GO:0005623 cell; GO:0005737 cytoplasm; GO:0005739 mitochondrion; GO:0005740 mitochondrial envelope; GO:0005743 mitochondrial inner membrane; GO:0005746 mitochondrial respiratory chain; GO:000575 0	GO:0003824 catalytic activity; GO:0005488 binding; GO:0008121 ubiquinol-cytochrome-c reductase activity; GO:0009055 electron carrier activity; GO:0016491 oxidoreductase activity; GO:0016679 oxidoreductase activity, acting on diphenols and related substances as donors;			C	KOG4763	Ubiquinol-cytochrome c reductase hinge protein
Q16270	Insulin-like growth factor-binding protein 7 OS=Homo sapiens OX=9606 GN=IGFBP7 PE=1 SV=1	IGFBP7	1.478	0.031282051	Up	K23580	IGFBP7; insulin-like growth factor-binding protein 7		GO:0006464 cellular protein modification process; GO:0006807 nitrogen compound metabolic process; GO:0007155 cell adhesion; GO:0007275 multicellular organism development; GO:0007423 sensory organ development; GO:0008152 metabolic process; GO:0008285 negative	GO:0005576 extracellular region; GO:0005622 intracellular; GO:0005623 cell; GO:0005737 cytoplasm; GO:0005783 endoplasmic reticulum; GO:0005788 endoplasmic reticulum lumen; GO:0012505 endomembrane system; GO:0031974 membrane-		PF00219; PF07648; PF07679	Insulin-like growth factor binding protein; Kazal-type serine protease inhibitor domain; Immunoglobulin I-set domain			
O15018	PDZ domain-containing protein 2 OS=Homo sapiens OX=9606 GN=PDZD2 PE=1 SV=4	PDZD2	1.478	0.011086849	Up				GO:0007154 cell communication; GO:0007165 signal transduction; GO:0009987 cellular process; GO:0023052 signaling; GO:0035556 intracellular signal transduction; GO:0050789 regulation of biological process; GO:0050794 regulation of cellular process;	GO:0005576 extracellular region; GO:0005622 intracellular; GO:0005623 cell; GO:0005634 nucleus; GO:0005737 cytoplasm; GO:0005783 endoplasmic reticulum; GO:0005911 cell-cell junction; GO:0012505 endomembrane system; GO:0014069 postsynapti		PF00595	PDZ domain (Also known as DHR or GLGF)	F	KOG3528	FOG: PDZ domain
Q92845	Kinesin-associated protein 3 OS=Homo sapiens OX=9606 GN=KIFAP3 PE=1 SV=2	KIFAP3	1.476	0.017088442	Up				GO:0002376 immune system process; GO:0002478 antigen processing and presentation of exogenous peptide antigen; GO:0002495 antigen processing and presentation of peptide antigen via MHC class II; GO:0002504 antigen processing and presentation of peptide or polysaccha	GO:0000228 nuclear chromosome; GO:0000793 condensed chromosome; GO:0000794 condensed nuclear chromosome; GO:0005622 intracellular; GO:0005623 cell; GO:0005634 nucleus; GO:0005694 chromosome; GO:0005737 cytoplasm; GO:00057	GO:0005488 binding; GO:0005515 protein binding; GO:0008092 cytoskeletal protein binding; GO:0019894 kinesin binding;			U	KOG1222	Kinesin associated protein KAP

Q6I9Y2	THO complex subunit 7 homolog OS=Homo sapiens OX=9606 GN=THOC7 PE=1 SV=3	THOC7	1.471	0.043400935	Up	K13176	THOC7; THO complex subunit 7	map03013 RNA transport	GO:0006139 nucleobase-containing compound metabolic process; GO:0006396 RNA processing; GO:0006397 mRNA processing; GO:0006403 RNA localization; GO:0006405 RNA export from nucleus; GO:0006406 mRNA export from nucleus; GO:0006611 protein export from	GO:0000346 transcription export complex; GO:0000347 THO complex; GO:0000445 THO complex part of transcription export complex; GO:0000781 chromosome, telomeric region; GO:0005622 intracellular; GO:0005623 cell; GO:0005634 nucleus; GO:0005654				S	KOG3215	Uncharacterized conserved protein
Q9H871	E3 ubiquitin-protein transferase RMND5A OS=Homo sapiens OX=9606 GN=RMND5A PE=1 SV=1	RMND5A	1.467	0.035000611	Up	K23333	RMND5; E3 ubiquitin-protein transferase RMND5 [EC:2.3.2.27]		GO:0006508 proteolysis; GO:0006511 ubiquitin-dependent protein catabolic process; GO:0006807 nitrogen compound metabolic process; GO:0008152 metabolic process; GO:0009056 catabolic process; GO:0009057 macromolecule catabolic process; GO:0009987 cellular	GO:0005622 intracellular; GO:0005623 cell; GO:0005634 nucleus; GO:0005737 cytoplasm; GO:0032991 macromolecular complex; GO:0034657 GID complex; GO:0043226 organelle; GO:0043227 membrane-bounded organelle; GO:0043229 intracellular organelle;		PF10607; PF13445	CTLH/CRA C-terminal to LisH motif domain; RING-type zinc-finger, LisH dimerisation motif	O	KOG2817	Predicted E3 ubiquitin ligase
P78549	Endonuclease III-like protein 1 OS=Homo sapiens OX=9606 GN=NTHL1 PE=1 SV=2	NTHL1	1.465	0.019380458	Up	K10773	NTH; endonuclease III [EC:4.2.99.18]	map03410 Base excision repair	GO:0006139 nucleobase-containing compound metabolic process; GO:0006220 pyrimidine nucleotide metabolic process; GO:0006244 pyrimidine nucleotide catabolic process; GO:0006259 DNA metabolic process; GO:0006281 DNA repair; GO:0006284 base-excision repair; GO:00062	GO:0005622 intracellular; GO:0005623 cell; GO:0005634 nucleus; GO:0005654 nucleoplasm; GO:0005737 cytoplasm; GO:0005739 mitochondrion; GO:0031974 membrane-enclosed lumen; GO:0031981 nuclear lumen; GO:0043226 organelle; GO:00432	GO:0000702 oxidized base lesion DNA N-glycosylase activity; GO:0000703 oxidized pyrimidine nucleobase lesion DNA N-glycosylase activity; GO:0003676 nucleic acid binding; GO:0003677 DNA binding; GO:0003690 double-stranded DNA binding; GO:0003824 catalytic activity;	PF00730	HhH-GPD superfamily base excision DNA repair protein	L	KOG1921	Endonuclease III
Q9Y2E5	Epididymis-specific alpha-mannosidase OS=Homo sapiens OX=9606 GN=MAN2B2 PE=1 SV=4	MAN2B2	1.465	0.041151173	Up	K12312	MAN2B2; epididymis-specific alpha-mannosidase [EC:3.2.1.24]	map00511 Other glycan degradation	GO:0005975 carbohydrate metabolic process; GO:0005996 monosaccharide metabolic process; GO:0006013 mannose metabolic process; GO:0006464 cellular protein modification process; GO:0006517 protein deglycosylation; GO:0006807 nitrogen compound metabolic process;	GO:0000323 lytic vacuole; GO:0005576 extracellular region; GO:0005622 intracellular; GO:0005623 cell; GO:0005737 cytoplasm; GO:0005764 lysosome; GO:0005773 vacuole; GO:0005775 vacuolar lumen; GO:0031974 membrane-enclosed lumen; GO:00432	GO:0003824 catalytic activity; GO:0004553 hydrolase activity, hydrolyzing O-glycosyl compounds; GO:0004559 alpha-mannosidase activity; GO:0008496 mannan endo-1,6-alpha-mannosidase activity; GO:0015923 mannosidase activity; GO:0016787 hydrolase activity; GO:00167	PF01074; PF09261; PF07748	Glycosyl hydrolases family 38 N-terminal domain; Alpha mannosidase, middle domain; Glycosyl hydrolases family 38 C-terminal domain	G	KOG1959	Glycosyl hydrolase, family 38 - alpha-mannosidase
Q92973	Transportin-1 OS=Homo sapiens OX=9606 GN=TNPO1 PE=1 SV=2	TNPO1	1.464	0.037096271	Up	K18752	TNPO1, IPO2, KPNB2; transportin-1		GO:0000060 protein import into nucleus, translocation; GO:0006606 protein import into nucleus; GO:0006607 NLS-bearing protein import into nucleus; GO:0006610 ribosomal protein import into nucleus; GO:0006810 transport; GO:0006886 intracellular protein transport; GO:00069	GO:0005622 intracellular; GO:0005623 cell; GO:0005634 nucleus; GO:0005635 nuclear envelope; GO:0005737 cytoplasm; GO:0005829 cytosol; GO:0005929 cilium; GO:0012505 endomembrane system; GO:0016020 membrane; GO:0031090 organelle membrane; GO:00319	GO:0005048 signal sequence binding; GO:0005215 transporter activity; GO:0005488 binding; GO:0008139 nuclear localization sequence binding; GO:0008565 protein transporter activity; GO:0033218 amide binding; GO:0042277 peptide binding;			UY	KOG2023	Nuclear transport receptor Karyopherin-beta2/Transportin (importin beta superfamily)
Q6P1M9	Armadillo repeat-containing X-linked protein 5 OS=Homo sapiens OX=9606 GN=ARMCX5 PE=1 SV=1	ARMCX5	1.461	0.021621318	Up											
Q96C57	Protein CUSTOS OS=Homo sapiens OX=9606 GN=CUSTOS PE=1 SV=2	CUSTOS	1.457	0.00052401	Up											

Q15771	Ras-related protein Rab-30 OS=Homo sapiens OX=9606 GN=RAB30 PE=1 SV=2	RAB30	1.454	0.001713269	Up	K07917	RAB30; Ras-related protein Rab-30		GO:0006996 organelle organization; GO:0007030 Golgi organization; GO:0009987 cellular process; GO:0010256 endomembrane system organization; GO:0016043 cellular component organization; GO:0071840 cellular component organization or biogenesis;	GO:0000139 Golgi membrane; GO:0005622 intracellular; GO:0005623 cell; GO:0005737 cytoplasm; GO:0005794 Golgi apparatus; GO:0005795 Golgi stack; GO:0005801 cis-Golgi network; GO:0005802 trans-Golgi network; GO:0012505 endomembrane system;	GO:0003824 catalytic activity; GO:0003924 GTPase activity; GO:0016462 pyrophosphatase activity; GO:0016787 hydrolase activity; GO:0016817 hydrolase activity, acting on acid anhydrides; GO:0016818 hydrolase activity, acting on acid anhydrides, in phosphorus	PF00071	Ras family	U	KOG0095	GTPase Rab30, small G protein superfamily
Q15048	Leucine-rich repeat-containing protein 14 OS=Homo sapiens OX=9606 GN=LRRC14 PE=1 SV=1	LRRC14	1.453	0.036790128	Up				GO:0002682 regulation of immune system process; GO:0002683 negative regulation of immune system process; GO:0006355 regulation of	GO:0005622 intracellular; GO:0005623 cell; GO:0005737 cytoplasm; GO:0044424 intracellular part; GO:0044464 cell part;	GO:0005488 binding; GO:0005515 protein binding; GO:0019899 enzyme binding; GO:0019900 kinase binding;					
Q96FX7	tRNA (adenine(58)-N(1))-methyltransferase catalytic subunit TRMT61A OS=Homo sapiens OX=9606 GN=TRMT61A PE=1 SV=1	TRMT61A	1.452	0.048437658	Up	K07442	TRM61, GCD14; tRNA (adenine57-N1/adenine58-N1)-methyltransferase catalytic subunit [EC:2.1.1.219 2.1.1.220]		GO:0001510 RNA methylation; GO:0006139 nucleobase-containing compound metabolic process; GO:0006725 cellular aromatic compound metabolic process; GO:0006807 nitrogen compound metabolic process; GO:0008152 metabolic process; GO:0009451 RNA modification; GO:00099	GO:0005622 intracellular; GO:0005623 cell; GO:0005634 nucleus; GO:0005654 nucleoplasm; GO:0005737 cytoplasm; GO:0031515 tRNA (m1A) methyltransferase complex; GO:0031974 membrane-enclosed lumen; GO:0031981 nuclear lumen; GO:0032991	GO:0003824 catalytic activity; GO:0008168 methyltransferase activity; GO:0008173 RNA methyltransferase activity; GO:0008174 mRNA methyltransferase activity; GO:0008757 S-adenosylmethionine-dependent methyltransferase activity; GO:0016740 transferase activity; GO:00167			J	KOG2915	tRNA(1-methyladenosine) methyltransferase, subunit GCD14
Q6P1X6	UPF0598 protein C8orf82 OS=Homo sapiens OX=9606 GN=C8orf82 PE=1 SV=2	C8orf82	1.445	0.001145104	Up											
Q9GZQ3	COMM domain-containing protein 5 OS=Homo sapiens OX=9606 GN=COMMD5 PE=1 SV=1	COMMD5	1.443	0.015544625	Up	K22561	COMMD5, HCARG; COMM domain containing 5		GO:0001558 regulation of cell growth; GO:0001655 urogenital system development; GO:0001822 kidney development; GO:0002009 morphogenesis of an epithelium; GO:0007049 cell cycle; GO:0007050 cell cycle arrest; GO:0007275 multicellular	GO:0005622 intracellular; GO:0005623 cell; GO:0005634 nucleus; GO:0005654 nucleoplasm; GO:0005737 cytoplasm; GO:0005829 cytosol; GO:0031974 membrane-enclosed lumen; GO:0031981 nuclear lumen; GO:0043226 organelle; GO:0043227 membrane-		PF07258	HCaRG protein			
Q9NQ89	Protein C12orf4 OS=Homo sapiens OX=9606 GN=C12orf4 PE=1 SV=1	C12orf4	1.443	0.000643537	Up				GO:0002682 regulation of immune system process; GO:0002684 positive regulation of immune system process; GO:0002694 regulation of leukocyte activation; GO:0002697 regulation of immune effector process; GO:0002699 positive regulation of immune effector process; GO:00027	GO:0005622 intracellular; GO:0005623 cell; GO:0005737 cytoplasm; GO:0044424 intracellular part; GO:0044464 cell part;				S	KOG4506	Uncharacterized conserved protein

P12271	Retinaldehyde-binding protein 1 OS=Homo sapiens OX=9606 GN=RLBP1 PE=1 SV=2	RLBP1	1.442	0.022773173	Up	K19625	RLBP1; retinaldehyde-binding protein 1		GO:0001523 retinoid metabolic process; GO:0003008 system process; GO:0006629 lipid metabolic process; GO:0006720 isoprenoid metabolic process; GO:0006721 terpenoid metabolic process; GO:0006766 vitamin metabolic process; GO:0006775 fat-soluble vitamin metabolic process;	GO:0005622 intracellular; GO:0005623 cell; GO:0005737 cytoplasm; GO:0005829 cytosol; GO:0044297 cell body; GO:0044424 intracellular part; GO:0044444 cytoplasmic part; GO:0044464 cell part;	GO:0005488 binding; GO:0005501 retinoid binding; GO:0005502 11-cis retinal binding; GO:0008289 lipid binding; GO:0016918 retinal binding; GO:0019840 isoprenoid binding; GO:0019842 vitamin binding; GO:0036094 small molecule binding;	PF03765; PF00650	CRAL/TRIO, N-terminal domain; CRAL/TRIO domain	I	KOG1471	Phosphatidylinositol transfer protein SEC14 and related proteins
P07108	Acyl-CoA-binding protein OS=Homo sapiens OX=9606 GN=DBI PE=1 SV=2	DBI	1.439	0.01581405	Up	K08762	DBI, ACBP; diazepam-binding inhibitor (GABA receptor modulator, acyl-CoA-binding protein)	map03320 PPAR signaling pathway	GO:0001662 behavioral fear response; GO:0001942 hair follicle development; GO:0002209 behavioral defense response; GO:0003008 system process; GO:0006139 nucleobase-containing compound metabolic process; GO:0006163 purine nucleotide metabolic process; GO:00066	GO:0005576 extracellular region; GO:0005615 extracellular space; GO:0005622 intracellular; GO:0005623 cell; GO:0005634 nucleus; GO:0005737 cytoplasm; GO:0005783 endoplasmic reticulum; GO:0005794 Golgi apparatus; GO:0005886 plasma membrane; GO:00080	GO:0000062 fatty-acyl-CoA binding; GO:0000166 nucleotide binding; GO:0005102 receptor binding; GO:0005488 binding; GO:0005515 protein binding; GO:0017076 purine nucleotide binding; GO:0030156 benzodiazepine receptor binding; GO:0030554 adenyl nucleotide binding; GO:00325	PF00887	Acyl CoA binding protein	I	KOG0817	Acyl-CoA-binding protein
Q9Y508	E3 ubiquitin-protein ligase RNF114 OS=Homo sapiens OX=9606 GN=RNF114 PE=1 SV=1	RNF114	1.438	0.004817742	Up	K15697	RNF114; RING finger protein 114 [EC:2.3.2.27]		GO:0000209 protein polyubiquitination; GO:0006464 cellular protein modification process; GO:0006807 nitrogen compound metabolic process; GO:0008152 metabolic process; GO:0009893 positive regulation of metabolic process; GO:0009894 regulation of catabolic process; GO:00098	GO:0005622 intracellular; GO:0005623 cell; GO:0005737 cytoplasm; GO:0005829 cytosol; GO:0005886 plasma membrane; GO:0016020 membrane; GO:0044424 intracellular part; GO:0044444 cytoplasmic part; GO:0044464 cell part; GO:0071944 cell periphery;	GO:0003824 catalytic activity; GO:0004842 ubiquitin-protein transferase activity; GO:0005488 binding; GO:0005515 protein binding; GO:0016740 transferase activity; GO:0019787 ubiquitin-like protein transferase activity; GO:0019899 enzyme binding; GO:0031624 ubiquitin conjugatin	PF13445; PF05605	RING-type zinc-finger, LisH dimerisation motif; Drought induced 19 protein (Di19)			
Q9H3Z4	DnaJ homolog subfamily C member 5 OS=Homo sapiens OX=9606 GN=DNAJC5 PE=1 SV=1	DNAJC5	1.435	0.043346628	Up	K09525	DNAJC5; DnaJ homolog subfamily C member 5	map04141 Protein processing in endoplasmic reticulum	GO:0001505 regulation of neurotransmitter levels; GO:0001775 cell activation; GO:0002252 immune effector process; GO:0002263 cell activation involved in immune response; GO:0002274 myeloid leukocyte activation; GO:0002275 myeloid cell activation involved in immune response;	GO:0000323 lytic vacuole; GO:0005622 intracellular; GO:0005623 cell; GO:0005737 cytoplasm; GO:0005764 lysosome; GO:0005765 lysosomal membrane; GO:0005766 primary lysosome; GO:0005773 vacuole; GO:0005774 vacuolar membrane; GO:0005886 plasma membrane;	GO:0005488 binding; GO:0005515 protein binding; GO:0043008 ATP-dependent protein binding;	PF00226	DnaJ domain	O	KOG0716	Molecular chaperone (DnaJ superfamily)
Q6PJG6	BRCA1-associated ATM activator 1 OS=Homo sapiens OX=9606 GN=BRAT1 PE=1 SV=2	BRAT1	1.433	0.045789778	Up	K23112	BRAT1; BRCA1-associated ATM activator 1		GO:0001558 regulation of cell growth; GO:0001932 regulation of protein phosphorylation; GO:0001934 positive regulation of protein phosphorylation; GO:0005975 carbohydrate metabolic process; GO:0005996 monosaccharide metabolic process; GO:0006006 glucose	GO:0005622 intracellular; GO:0005623 cell; GO:0005634 nucleus; GO:0005654 nucleoplasm; GO:0005737 cytoplasm; GO:0031974 membrane-enclosed lumen; GO:0031981 nuclear lumen; GO:0043226 organelle; GO:0043227 membrane-bounded organelle;						

Q99571	P2X purinoceptor 4 OS=Homo sapiens OX=9606 GN=P2RX4 PE=1 SV=2	P2RX4	1.432	0.018963371	Up	K05218	P2RX4; P2X purinoceptor 4	map04020 Calcium signaling pathway; map04080 Neuroactive ligand-receptor interaction	GO:0001505 regulation of neurotransmitter levels; GO:0001508 action potential; GO:0001775 cell activation; GO:0001894 tissue homeostasis; GO:0002028 regulation of sodium ion transport; GO:0002682 regulation of immune system process; GO:0002684 positive	GO:0005622 intracellular; GO:0005623 cell; GO:0005634 nucleus; GO:0005635 nuclear envelope; GO:0005637 nuclear inner membrane; GO:0005639 integral component of nuclear inner membrane; GO:0005737 cytoplasm; GO:0005886 plasma membrane; GO:0005887 integral component of plasma	GO:0000166 nucleotide binding; GO:0001614 purinergic nucleotide receptor activity; GO:0004888 transmembrane signaling receptor activity; GO:0004931 extracellular ATP-gated cation channel activity; GO:0005102 receptor binding; GO:0005215 transporter					
P50749	Ras association domain-containing protein 2 OS=Homo sapiens OX=9606 GN=RASSF2 PE=1 SV=1	RASSF2	1.431	0.022568283	Up	K09851	RASSF2_4; Ras association domain-containing protein 2/4	map04391 Hippo signaling pathway - fly; map04392 Hippo signaling pathway - multiple species	GO:0001501 skeletal system development; GO:0001503 ossification; GO:0001932 regulation of protein phosphorylation; GO:0001933 negative regulation of protein phosphorylation; GO:0001934 positive regulation of protein phosphorylation; GO:0002682 regulation of immune	GO:0000775 chromosome, centromeric region; GO:0000776 kinetochore; GO:0005622 intracellular; GO:0005623 cell; GO:0005634 nucleus; GO:0005654 nucleoplasm; GO:0005694 chromosome; GO:0005737 cytoplasm; GO:0005794 Golgi	GO:0003824 catalytic activity; GO:0004672 protein kinase activity; GO:0016301 kinase activity; GO:0016740 transferase activity; GO:0016772 transferase activity, transferring phosphorus-containing groups; GO:0016773 phosphotransferase activity, alcohol group as acceptor;	PF00788	Ras association (RalGDS/AF-6) domain	T	KOG4239	Ras GTPase effector RASSF2
Q96GD4	Aurora kinase B OS=Homo sapiens OX=9606 GN=AURKB PE=1 SV=3	AURKB	1.428	0.040001057	Up	K11479	AURKB; aurora kinase B [EC:2.7.11.1]		GO:0000022 mitotic spindle elongation; GO:0000070 mitotic sister chromatid segregation; GO:0000075 cell cycle checkpoint; GO:0000122 negative regulation of transcription from RNA polymerase II promoter; GO:0000226 microtubule cytoskeleton organizatio	GO:0000228 nuclear chromosome; GO:0000775 chromosome, centromeric region; GO:0000776 kinetochore; GO:0000779 condensed chromosome, centromeric region; GO:0000780 condensed nuclear chromosome, centromeric region; GO:0000793	GO:0003824 catalytic activity; GO:0004672 protein kinase activity; GO:0004674 protein serine/threonine kinase activity; GO:0004712 protein serine/threonine/tyrosine kinase activity; GO:0005488 binding; GO:0005515 protein binding; GO:0016301 kinase activity; GO:0016740 transferase activity;	PF00069	Protein kinase domain	D	KOG0580	Serine/threonine protein kinase
Q8TEA1	tRNA (cytosine(72)-C(5))-methyltransferase NSUN6 OS=Homo sapiens OX=9606 GN=NSUN6 PE=1 SV=1	NSUN6	1.425	0.036757836	Up	K21971	NSUN6; methyltransferase NSUN6 [EC:2.1.1.-]		GO:0001510 RNA methylation; GO:0006139 nucleobase-containing compound metabolic process; GO:0006396 RNA processing; GO:0006399 tRNA metabolic process; GO:0006400 tRNA modification; GO:0006725 cellular aromatic compound metabolic process; GO:0006807 nitrogen compound	GO:0005622 intracellular; GO:0005623 cell; GO:0005737 cytoplasm; GO:0005829 cytosol; GO:0044424 intracellular part; GO:0044444 cytoplasmic part; GO:0044464 cell part;	GO:0000049 tRNA binding; GO:0003676 nucleic acid binding; GO:0003723 RNA binding; GO:0003824 catalytic activity; GO:0005488 binding; GO:0008168 methyltransferase activity; GO:0008173 RNA methyltransferase activity; GO:0008175 tRNA methyltransferase activity; GO:00087			A	KOG1122	tRNA and rRNA cytosine-C5-methylase (nucleolar protein NOL1/NOP2)
O60830	Mitochondrial import inner membrane translocase subunit Tim17-B OS=Homo sapiens OX=9606 GN=TIMM17B PE=1 SV=1	TIMM17B	1.424	0.028799994	Up	K17795	TIM17; mitochondrial import inner membrane translocase subunit TIM17		GO:0006605 protein targeting; GO:0006626 protein targeting to mitochondrion; GO:0006810 transport; GO:0006839 mitochondrial transport; GO:0006886 intracellular protein transport; GO:0006996 organelle organization; GO:0007005 mitochondrion organizatio	GO:0005622 intracellular; GO:0005623 cell; GO:0005737 cytoplasm; GO:0005739 mitochondrion; GO:0005740 mitochondrial envelope; GO:0005743 mitochondrial inner membrane; GO:0005744 mitochondrial inner membrane presequence translocase				U	KOG1652	Mitochondrial import inner membrane translocase, subunit TIM17

O00244	Copper transport protein ATOX1 OS=Homo sapiens OX=9606 GN=ATOX1 PE=1 SV=1	ATOX1	1.423	0.000811966	Up	K07213	ATOX1, ATX1, copZ, golB; copper chaperone	map04978 Mineral absorption	GO:0000041 transition metal ion transport; GO:0006810 transport; GO:0006811 ion transport; GO:0006812 cation transport; GO:0006825 copper ion transport; GO:0006873 cellular ion homeostasis; GO:0006875 cellular metal ion homeostasis; GO:0006878 cellular copper ion	GO:0005622 intracellular; GO:0005623 cell; GO:0005737 cytoplasm; GO:0005829 cytosol; GO:0044424 intracellular part; GO:0044444 cytoplasmic part; GO:0044464 cell part;	GO:0005488 binding; GO:0005507 copper ion binding; GO:0005515 protein binding; GO:0016530 metallochaperone activity; GO:0016531 copper chaperone activity; GO:0032767 copper-dependent protein binding; GO:0043167 ion binding; GO:0043169 cation binding; GO:0046872 metal	PF00403	Heavy-metal-associated domain	P	KOG1603	Copper chaperone
P01024	Complement C3 OS=Homo sapiens OX=9606 GN=C3 PE=1 SV=2	C3	1.419	0.008980706	Up	K03990	C3; complement component 3	map04080 Neuroactive ligand-receptor interaction; map04145 Phagosome; map04610 Complement and coagulation cascades; map05131 Shigellosis; map05133 Pertussis; map05134 Legionellosis; map05140 Leishmaniasis; map05142 Chagas disease (American trypanosomiasis); map05150 Staphylococcus aureus infection; map05152 Tuberculosis; map05167 Kaposi sarcoma-associated herpesvirus infection; map05168 Herpes simplex virus 1 infection; map05203 Viral carcinogenesis; map05322 Systemic lupus erythematosus	GO:0001775 cell activation; GO:0001796 regulation of type IIa hypersensitivity; GO:0001798 positive regulation of type IIa hypersensitivity; GO:0001817 regulation of cytokine production; GO:0001819 positive regulation of cytokine production; GO:0001932 regulation of protein phosphorylation;	GO:0000323 lytic vacuole; GO:0005576 extracellular region; GO:0005615 extracellular space; GO:0005622 intracellular; GO:0005623 cell; GO:0005737 cytoplasm; GO:0005764 lysosome; GO:0005766 primary lysosome; GO:0005773 vacuole; GO:0005775 vacuolar	GO:0001664 G-protein coupled receptor binding; GO:0003824 catalytic activity; GO:0004175 endopeptidase activity; GO:0004252 serine-type endopeptidase activity; GO:0005102 receptor binding; GO:0005488 binding; GO:0005515 protein binding; GO:0008233 peptidase	PF01835; PF07703; PF01821; PF07678; PF07677; PF01759	MG2 domain; Alpha-2-macroglobulin family N-terminal region; Anaphylotoxin-like domain; A-macroglobulin complement component; A-macroglobulin receptor; UNC-6/NTR/C345C module	O	KOG1366	Alpha-macroglobulin
Q15041	ADP-ribosylation factor-like protein 6-interacting protein 1 OS=Homo sapiens OX=9606 GN=ARL6IP1 PE=1 SV=2	ARL6IP1	1.417	0.002638877	Up	K02957	RP-S15Ae, RPS15A; small subunit ribosomal protein S15Ae	map03010 Ribosome	GO:0001505 regulation of neurotransmitter levels; GO:0002036 regulation of L-glutamate transport; GO:0002038 positive regulation of L-glutamate transport; GO:0006605 protein targeting; GO:0006612 protein targeting to membrane; GO:0006613 cotranslational protein targeting to	GO:0005622 intracellular; GO:0005623 cell; GO:0005737 cytoplasm; GO:0005783 endoplasmic reticulum; GO:0005784 Sec61 translocon complex; GO:0005789 endoplasmic reticulum membrane; GO:0005791 rough endoplasmic reticulum; GO:0005829 cytosol; GO:00125	GO:0005488 binding; GO:0005515 protein binding; GO:0042802 identical protein binding;					
Q9BXR0	Queuine tRNA-ribosyltransferase catalytic subunit 1 OS=Homo sapiens OX=9606 GN=QTRT1 PE=1 SV=3	QTRT1	1.416	0.025169636	Up	K00773	tgt, QTRT1; queuine tRNA-ribosyltransferase [EC:2.4.2.29]		GO:0006139 nucleobase-containing compound metabolic process; GO:0006396 RNA processing; GO:0006399 tRNA metabolic process; GO:0006400 tRNA modification; GO:0006725 cellular aromatic compound metabolic process; GO:0006807 nitrogen compound metabolic process; GO:0008033 tRNA	GO:0005622 intracellular; GO:0005623 cell; GO:0005634 nucleus; GO:0005737 cytoplasm; GO:0005739 mitochondrion; GO:0005740 mitochondrial envelope; GO:0005741 mitochondrial outer membrane; GO:0016020 membrane; GO:0019867 outer membrane;	GO:0003824 catalytic activity; GO:0005488 binding; GO:0005515 protein binding; GO:0008479 queuine tRNA-ribosyltransferase activity; GO:0016740 transferase activity; GO:0016757 transferase activity, transferring glycosyl groups; GO:0016763 transferase activity, transferring pentosyl			A	KOG3908	Queuine-tRNA ribosyltransferase
Q13907	Isopentenyl-diphosphate Delta-isomerase 1 OS=Homo sapiens OX=9606 GN=IDI1 PE=1 SV=2	IDI1	1.415	0.018022576	Up	K01823	idi, IDI; isopentenyl-diphosphate Delta-isomerase [EC:5.3.3.2]	map00900 Terpenoid backbone biosynthesis; map01100 Metabolic pathways	GO:0006066 alcohol metabolic process; GO:0006629 lipid metabolic process; GO:0006644 phospholipid metabolic process; GO:0006694 steroid biosynthetic process; GO:0006695 cholesterol biosynthetic process; GO:0006720 isoprenoid metabolic process; GO:0006793 phosphorus	GO:0005622 intracellular; GO:0005623 cell; GO:0005737 cytoplasm; GO:0005777 peroxisome; GO:0005829 cytosol; GO:0042579 microbody; GO:0043226 organelle; GO:0043227 membrane-bounded organelle; GO:0043229 intracellular organelle; GO:00432	GO:0000287 magnesium ion binding; GO:0003824 catalytic activity; GO:0004452 isopentenyl-diphosphate delta-isomerase activity; GO:0005488 binding; GO:0016853 isomerase activity; GO:0016860 intramolecular oxidoreductase activity; GO:0016863	PF00293	NUDIX domain	Q	KOG0142	Isopentenyl pyrophosphate:dimethylallyl pyrophosphate isomerase

Q15653	NF-kappa-B inhibitor beta OS=Homo sapiens OX=9606 GN=NFKBIB PE=1 SV=2	NFKBIB	1.414	0.005984354	Up	K02581	NFKBIB; NF-kappa-B inhibitor beta	map04062 Chemokine signaling pathway; map04621 NOD-like receptor signaling pathway; map04622 RIG-I-like receptor signaling pathway; map04623 Cytosolic DNA-sensing pathway; map04658 Th1 and Th2 cell differentiation; map04659 Th17 cell differentiation; map04660 T cell receptor signaling pathway; map04662 B cell receptor signaling pathway; map04722 Neurotrophin signaling pathway; map04920 Adipocytokine signaling pathway; map05130 Pathogenic Escherichia coli infection; map05131 Shigellosis; map05140 Leishmaniasis; map05145 Toxoplasmosis; map05162 Measles; map05164 Influenza A; map05169 Epstein-Barr virus infection; map05235 PD-L1 expression and PD-1 checkpoint pathway in cancer	GO:0002237 response to molecule of bacterial origin; GO:0006139 nucleobase-containing compound metabolic process; GO:0006351 transcription, DNA-templated; GO:0006355 regulation of transcription, DNA-templated; GO:0006725 cellular aromatic compound metabolic process;	GO:0005622 intracellular; GO:0005623 cell; GO:0005634 nucleus; GO:0005737 cytoplasm; GO:0005829 cytosol; GO:0032991 macromolecular complex; GO:0033256 I-kappaB/NF-kappaB complex; GO:0043226 organelle; GO:0043227 membrane-bounded organelle;	GO:0003712 transcription cofactor activity; GO:0003713 transcription coactivator activity; GO:0005488 binding; GO:0005515 protein binding; GO:0008134 transcription factor binding; GO:0051059 NF-kappaB binding;			K	KOG0504	FOG: Ankyrin repeat
Q93045	Stathmin-2 OS=Homo sapiens OX=9606 GN=STMN2 PE=1 SV=3	STMN2	1.414	0.012827972	Up				GO:0000226 microtubule cytoskeleton organization; GO:0006996 organelle organization; GO:0007010 cytoskeleton organization; GO:0007017 microtubule-based process; GO:0007019 microtubule depolymerization; GO:00070	GO:0005622 intracellular; GO:0005623 cell; GO:0005737 cytoplasm; GO:0005794 Golgi apparatus; GO:0012505 endomembrane system; GO:0030027 lamellipodium; GO:0030424 axon; GO:0030426 growth cone; GO:0030427 site of polarized growth; GO:00312	GO:0005488 binding; GO:0005515 protein binding; GO:0008092 cytoskeletal protein binding; GO:0015631 tubulin binding; GO:0048306 calcium-dependent protein binding;			Z	KOG1280	Uncharacterized conserved protein containing ZZ-type Zn-finger
P13987	CD59 glycoprotein OS=Homo sapiens OX=9606 GN=CD59 PE=1 SV=1	CD59	1.411	0.025409972	Up	K04008	CD59; CD59 antigen	map04610 Complement and coagulation cascades; map04640 Hematopoietic cell lineage	GO:0001775 cell activation; GO:0001969 regulation of activation of membrane attack complex; GO:0001971 negative regulation of activation of membrane attack complex; GO:0002252 immune effector process; GO:0002263 cell activation involved in immune response;	GO:0000139 Golgi membrane; GO:0005576 extracellular region; GO:0005615 extracellular space; GO:0005622 intracellular; GO:0005623 cell; GO:0005737 cytoplasm; GO:0005783 endoplasmic reticulum; GO:0005789 endoplasmic reticulum membrane; GO:00057	GO:0001848 complement binding; GO:0005488 binding; GO:0005515 protein binding;	PF00021	u-PAR/Ly-6 domain			
P00414	Cytochrome c oxidase subunit 3 OS=Homo sapiens OX=9606 GN=MT-CO3 PE=1 SV=2	MT-CO3	1.409	0.037478571	Up	K02262	COX3; cytochrome c oxidase subunit 3	map00190 Oxidative phosphorylation; map01100 Metabolic pathways; map04260 Cardiac muscle contraction; map04714 Thermogenesis; map04932 Non-alcoholic fatty liver disease (NAFLD); map05010 Alzheimer disease; map05012 Parkinson disease; map05016 Huntington disease	GO:0008535 respiratory chain complex IV assembly; GO:0009987 cellular process; GO:0016043 cellular component organization; GO:0017004 cytochrome complex assembly; GO:0022607 cellular component assembly; GO:0034622 cellular macromolecular complex assembly; GO:00439	GO:0005622 intracellular; GO:0005623 cell; GO:0005737 cytoplasm; GO:0005739 mitochondrion; GO:0005740 mitochondrial envelope; GO:0005743 mitochondrial inner membrane; GO:0016020 membrane; GO:0019866 organelle inner membrane;				C	KOG4664	Cytochrome oxidase subunit III and related proteins
Q9UMS0	NFU1 iron-sulfur cluster scaffold homolog, mitochondrial OS=Homo sapiens OX=9606 GN=NFU1 PE=1 SV=2	NFU1	1.409	0.040836877	Up	K22074	NFU1, HIRIP5; NFU1 iron-sulfur cluster scaffold homolog, mitochondrial		GO:0006790 sulfur compound metabolic process; GO:0008152 metabolic process; GO:0009987 cellular process; GO:0016043 cellular component organization; GO:0016226 iron-sulfur cluster assembly; GO:0022607 cellular component assembly; GO:0031163 metallo-sulfur cluster assembly;	GO:0005622 intracellular; GO:0005623 cell; GO:0005634 nucleus; GO:0005654 nucleoplasm; GO:0005737 cytoplasm; GO:0005739 mitochondrion; GO:0005829 cytosol; GO:0031974 membrane-enclosed lumen; GO:0031981 nuclear lumen; GO:0043226	GO:0005488 binding; GO:0005506 iron ion binding; GO:0043167 ion binding; GO:0043169 cation binding; GO:0046872 metal ion binding; GO:0046914 transition metal ion binding; GO:0048037 cofactor binding; GO:0051536 iron-sulfur cluster binding; GO:0051539 4 iron, 4 sulfur	PF08712	Scaffold protein Nfu/NifU N terminal	O	KOG2358	NifU-like domain-containing proteins

O76071	Probable cytosolic iron-sulfur protein assembly protein CIAO1 OS=Homo sapiens OX=9606 GN=CIAO1 PE=1 SV=1	CIAO1	1.409	0.046088905	Up				GO:0006355 regulation of transcription, DNA-templated; GO:0006357 regulation of transcription from RNA polymerase II promoter; GO:0006790 sulfur compound metabolic process; GO:0008152 metabolic process; GO:0008284 positive regulation of cell proliferatio	GO:0005622 intracellular; GO:0005623 cell; GO:0005737 cytoplasm; GO:0005819 spindle; GO:0005829 cytosol; GO:0005856 cytoskeleton; GO:0015630 microtubule cytoskeleton; GO:0032991 macromolecular complex; GO:0043226 organelle;	GO:0003700 transcription factor activity, sequence-specific DNA binding;			S	KOG0645	WD40 repeat protein
Q14956	Transmembrane glycoprotein NMB OS=Homo sapiens OX=9606 GN=GPNMB PE=1 SV=2	GPNMB	1.407	0.028264239	Up	K20732	GPNMB; transmembrane glycoprotein NMB		GO:0001503 ossification; GO:0001649 osteoblast differentiation; GO:0001817 regulation of cytokine production; GO:0001818 negative regulation of cytokine production; GO:0001932 regulation of protein phosphorylation; GO:0001934 positive regulation of protein phosphorylation;	GO:0005622 intracellular; GO:0005623 cell; GO:0005737 cytoplasm; GO:0005886 plasma membrane; GO:0005887 integral component of plasma membrane; GO:0016020 membrane; GO:0016021 integral component of membrane; GO:0031224 intrinsic component of membrane; GO:00312	GO:0005102 receptor binding; GO:0005178 integrin binding; GO:0005488 binding; GO:0005515 protein binding; GO:0005539 glycosaminoglycan binding; GO:0008201 heparin binding; GO:0030545 receptor regulator activity; GO:0042056 chemoattractant activity; GO:0043167 ion binding;	PF00801	PKD domain			
Q99933	BAG family molecular chaperone regulator 1 OS=Homo sapiens OX=9606 GN=BAG1 PE=1 SV=4	BAG1	1.406	0.041416496	Up	K09555	BAG1; BCL2-associated athanogene 1	map04141 Protein processing in endoplasmic reticulum	GO:0001932 regulation of protein phosphorylation; GO:0001933 negative regulation of protein phosphorylation; GO:0006355 regulation of transcription, DNA-templated; GO:0006357 regulation of transcription from RNA polymerase II promoter; GO:0006457 protein	GO:0005622 intracellular; GO:0005623 cell; GO:0005634 nucleus; GO:0005737 cytoplasm; GO:0005739 mitochondrion; GO:0005829 cytosol; GO:0043226 organelle; GO:0043227 membrane-bounded organelle; GO:0043229 intracellular organelle; GO:0043231	GO:0000166 nucleotide binding; GO:0000774 adenyl-nucleotide exchange factor activity; GO:0005488 binding; GO:0005515 protein binding; GO:0017076 purine nucleotide binding; GO:0019899 enzyme binding; GO:0030234 enzyme regulator activity; GO:0030554 adenyl nucleotide binding; GO:00316	PF00240	Ubiquitin family			
P0CG32	Zinc finger CCHC domain-containing protein 18 OS=Homo sapiens OX=9606 GN=ZCCHC18 PE=3 SV=1	ZCCHC18	1.406	0.038435266	Up				GO:0006355 regulation of transcription, DNA-templated; GO:0006357 regulation of transcription from RNA polymerase II promoter; GO:0007154 cell communication; GO:0007165 signal transduction; GO:0007166 cell surface receptor signaling pathway;	GO:0005622 intracellular; GO:0005623 cell; GO:0005634 nucleus; GO:0005654 nucleoplasm; GO:0016604 nuclear body; GO:0016607 nuclear speck; GO:0031974 membrane-enclosed lumen; GO:0031981 nuclear lumen; GO:0043226 organelle; GO:0043227	GO:0003712 transcription cofactor activity; GO:0003713 transcription coactivator activity; GO:0030374 ligand-dependent nuclear receptor transcription coactivator activity;					
Q9NXE4	Sphingomyelin phosphodiesterase 4 OS=Homo sapiens OX=9606 GN=SMPD4 PE=1 SV=3	SMPD4	1.398	0.014945834	Up	K12353	SMPD4; sphingomyelin phosphodiesterase 4 [EC:3.1.4.12]	map00600 Sphingolipid metabolism; map01100 Metabolic pathways	GO:0006629 lipid metabolic process; GO:0006643 membrane lipid metabolic process; GO:0006644 phospholipid metabolic process; GO:0006650 glycerophospholipid metabolic process; GO:0006664 glycolipid metabolic process; GO:0006665 sphingolipid	GO:0005622 intracellular; GO:0005623 cell; GO:0005737 cytoplasm; GO:0005783 endoplasmic reticulum; GO:0005789 endoplasmic reticulum membrane; GO:0005794 Golgi apparatus; GO:0005802 trans-Golgi network; GO:0012505 endomembrane system;	GO:0003824 catalytic activity; GO:0004620 phospholipase activity; GO:0004767 sphingomyelin phosphodiesterase activity; GO:0008081 phosphoric diester hydrolase activity; GO:0016298 lipase activity; GO:0016787 hydrolase activity; GO:0016788 hydrolase			S	KOG4396	Predicted membrane-anchored protein

O75506	Heat shock factor-binding protein 1 OS=Homo sapiens OX=9606 GN=HSBP1 PE=1 SV=1	HSBP1	1.398	0.023291892	Up	K19765	HSBP1; heat shock factor-binding protein 1	map04212 Longevity regulating pathway - worm	GO:0000122 negative regulation of transcription from RNA polymerase II promoter; GO:0006355 regulation of transcription, DNA-templated; GO:0006357 regulation of transcription from RNA polymerase II promoter; GO:0009889 regulation of	GO:0005622 intracellular; GO:0005623 cell; GO:0005634 nucleus; GO:0005654 nucleoplasm; GO:0031974 membrane-enclosed lumen; GO:0031981 nuclear lumen; GO:0043226 organelle; GO:0043227 membrane-bounded organelle; GO:0043229 intracellula	GO:0003712 transcription cofactor activity; GO:0003714 transcription corepressor activity; GO:0005488 binding; GO:0005515 protein binding; GO:0042802 identical protein binding;			K	KOG4117	Heat shock factor binding protein
Q9HAV0	Guanine nucleotide-binding protein subunit beta-4 OS=Homo sapiens OX=9606 GN=GNB4 PE=1 SV=3	GNB4	1.397	0.007484641	Up	K04538	GNB4; guanine nucleotide-binding protein subunit beta-4	map04014 Ras signaling pathway; map04062 Chemokine signaling pathway; map04151 PI3K-Akt signaling pathway; map04371 Apelin signaling pathway; map04713 Circadian entrainment; map04723 Retrograde endocannabinoid signaling; map04724 Glutamatergic synapse; map04725 Cholinergic synapse; map04726 Serotonergic synapse; map04727 GABAergic synapse; map04728 Dopaminergic synapse; map04926 Relaxin signaling pathway; map05032 Morphine addiction; map05034 Alcoholism; map05163 Human cytomegalovirus infection; map05167 Kaposi sarcoma-associated herpesvirus infection; map05170 Human immunodeficiency virus 1 infection; map05200 Pathways in cancer	GO:0006457 protein folding; GO:0007154 cell communication; GO:0007165 signal transduction; GO:0007186 G-protein coupled receptor signaling pathway; GO:0009987 cellular process; GO:0023052 signaling; GO:0050789 regulation of biological process; GO:00507	GO:0005622 intracellular; GO:0005623 cell; GO:0005737 cytoplasm; GO:0005829 cytosol; GO:0005834 heterotrimeric G-protein complex; GO:0005886 plasma membrane; GO:0009898 cytoplasmic side of plasma membrane; GO:0016020 membrane; GO:0019897	GO:0003824 catalytic activity; GO:0003924 GTPase activity; GO:0005488 binding; GO:0016462 pyrophosphatase activity; GO:0016787 hydrolase activity; GO:0016817 hydrolase activity, acting on acid anhydrides; GO:0016818 hydrolase activity, acting on acid anhydrides,			S	KOG0286	G-protein beta subunit
Q99538	Legumain OS=Homo sapiens OX=9606 GN=LGMN PE=1 SV=1	LGMN	1.395	0.00821652	Up	K01369	LGMN; legumain [EC:3.4.22.34]	map04142 Lysosome; map04612 Antigen processing and presentation	GO:0002376 immune system process; GO:0002478 antigen processing and presentation of exogenous peptide antigen; GO:0002495 antigen processing and presentation of peptide antigen via MHC class II; GO:0002504 antigen processing and presentation of peptide or polysaccha	GO:0000323 lytic vacuole; GO:0005576 extracellular region; GO:0005622 intracellular; GO:0005623 cell; GO:0005737 cytoplasm; GO:0005764 lysosome; GO:0005768 endosome; GO:0005770 late endosome; GO:0005773 vacuole; GO:0005775 vacuolar lumen;	GO:0003824 catalytic activity; GO:0004175 endopeptidase activity; GO:0004197 cysteine-type endopeptidase activity; GO:0008233 peptidase activity; GO:0008234 cysteine-type peptidase activity; GO:0016787 hydrolase activity; GO:0070011			O	KOG1348	Asparaginyl peptidases
A0A096LP01	Small integral membrane protein 26 OS=Homo sapiens OX=9606 GN=SMIM26 PE=3 SV=1	SMIM26	1.394	0.045197095	Up											
Q9Y6V0	Protein piccolo OS=Homo sapiens OX=9606 GN=PCLO PE=1 SV=5	PCLO	1.393	0.000481463	Up	K16882	PCLO; protein piccolo	map04911 Insulin secretion	GO:0001505 regulation of neurotransmitter levels; GO:0006810 transport; GO:0006836 neurotransmitter transport; GO:0006887 exocytosis; GO:0006996 organelle organization; GO:0007010 cytoskeleton organization; GO:0007154 cell	GO:0005622 intracellular; GO:0005623 cell; GO:0005737 cytoplasm; GO:0005856 cytoskeleton; GO:0005938 cell cortex; GO:0030863 cortical cytoskeleton; GO:0043226 organelle; GO:0043228 non-membrane-bounded organelle; GO:0043229 intracellula	GO:0005488 binding; GO:0005509 calcium ion binding; GO:0005515 protein binding; GO:0005522 profilin binding; GO:0005543 phospholipid binding; GO:0005544 calcium-dependent phospholipid binding; GO:0008289 lipid binding; GO:0043167 ion binding; GO:0043168 anion binding;	PF05715; PF00595; PF00168	Piccolo Zn-finger; PDZ domain (Also known as DHR or GLGF); C2 domain	U	KOG2060	Rab3 effector RIM1 and related proteins, contain PDZ and C2 domains
Q8IU85	Calcium/calmodulin-dependent protein kinase type 1D OS=Homo sapiens OX=9606 GN=CAMK1D PE=1 SV=1	CAMK1D	1.39	0.005077264	Up	K08794	CAMK1; calcium/calmodulin-dependent protein kinase I [EC:2.7.11.17]	map04020 Calcium signaling pathway; map04921 Oxytocin signaling pathway; map04925 Aldosterone synthesis and secretion; map05214 Glioma	GO:0002682 regulation of immune system process; GO:0002684 positive regulation of immune system process; GO:0002685 regulation of leukocyte migration; GO:0002687 positive regulation of leukocyte migration; GO:0002688 regulation of leukocyte chemotaxis;	GO:0005622 intracellular; GO:0005623 cell; GO:0005634 nucleus; GO:0005737 cytoplasm; GO:0043226 organelle; GO:0043227 membrane-bounded organelle; GO:0043229 intracellular organelle; GO:0043231 intracellular membrane-bounded organelle; GO:00444	GO:0003824 catalytic activity; GO:0004672 protein kinase activity; GO:0004674 protein serine/threonine kinase activity; GO:0004683 calmodulin-dependent protein kinase activity; GO:0005488 binding; GO:0005515 protein binding; GO:0005516 calmodulin binding; GO:0016301 kinase	PF00069	Protein kinase domain	T	KOG0032	Ca2+/calmodulin-dependent protein kinase, EF-Hand protein superfamily

Q9BQG2	Peroxisomal NADH pyrophosphatase NUDT12 OS=Homo sapiens OX=9606 GN=NUDT12 PE=1 SV=1	NUDT12	1.389	0.020439709	Up	K03426	E3.6.1.22, NUDT12, nudC; NAD+ diphosphatase [EC:3.6.1.22]	map00760 Nicotinate and nicotinamide metabolism; map01100 Metabolic pathways; map04146 Peroxisome	GO:0006139 nucleobase-containing compound metabolic process; GO:0006725 cellular aromatic compound metabolic process; GO:0006732 coenzyme metabolic process; GO:0006733 oxidoreduction coenzyme metabolic process; GO:0006739 NADP metabolic process; GO:0006742 NADP	GO:0005622 intracellular; GO:0005623 cell; GO:0005634 nucleus; GO:0005737 cytoplasm; GO:0005777 peroxisome; GO:0005782 peroxisomal matrix; GO:0031907 microbody lumen; GO:0031974 membrane-enclosed lumen; GO:0042579 microbody;	GO:0000210 NAD+ diphosphatase activity; GO:0003824 catalytic activity; GO:0004551 nucleotide diphosphatase activity; GO:0016462 pyrophosphatase activity; GO:0016787 hydrolase activity; GO:0016817 hydrolase activity, acting on acid anhydrides; GO:00168	PF09296; PF09297; PF00293	NADH pyrophosphatase-like rudimentary NUDIX domain; NADH pyrophosphatase zinc ribbon domain; NUDIX domain	L	KOG3084	NADH pyrophosphatase I of the Nudix family of hydrolases
Q96AE7	Tetratricopeptide repeat protein 17 OS=Homo sapiens OX=9606 GN=TTC17 PE=1 SV=1	TTC17	1.389	0.023281632	Up				GO:0006996 organelle organization; GO:0007010 cytoskeleton organization; GO:0007015 actin filament organization; GO:0008154 actin polymerization or depolymerization; GO:0009987 cellular process; GO:0016043 cellular component organization; GO:00226	GO:0005622 intracellular; GO:0005623 cell; GO:0005737 cytoplasm; GO:0005829 cytosol; GO:0005856 cytoskeleton; GO:0005886 plasma membrane; GO:0015629 actin cytoskeleton; GO:0016020 membrane; GO:0043226 organelle; GO:0043228 non-membrane-				Z	KOG4507	Uncharacterized conserved protein, contains TPR repeats
Q12983	BCL2/adenovirus E1B 19 kDa protein-interacting protein 3 OS=Homo sapiens OX=9606 GN=BNIP3 PE=1 SV=3	BNIP3	1.388	0.049331727	Up	K15464	BNIP3; BCL2/adenovirus E1B 19 kDa protein-interacting protein 3	map04068 FoxO signaling pathway; map04137 Mitophagy - animal; map04140 Autophagy - animal; map05131 Shigellosis; map05134 Legionellosis	GO:0000302 response to reactive oxygen species; GO:0000422 mitophagy; GO:0001666 response to hypoxia; GO:0002252 immune effector process; GO:0002376 immune system process; GO:0006807 nitrogen compound metabolic process; GO:0006810 transport; GO:0006839	GO:0005622 intracellular; GO:0005623 cell; GO:0005634 nucleus; GO:0005635 nuclear envelope; GO:0005654 nucleoplasm; GO:0005737 cytoplasm; GO:0005739 mitochondrion; GO:0005740 mitochondrial envelope; GO:0005741 mitochondrial outer	GO:0005488 binding; GO:0005515 protein binding; GO:0019899 enzyme binding; GO:0042802 identical protein binding; GO:0042803 protein homodimerization activity; GO:0046982 protein heterodimerization activity; GO:0046983 protein dimerization activity; GO:0051020 GTPase binding;					
Q9H1A3	Methyltransferase-like protein 9 OS=Homo sapiens OX=9606 GN=METTL9 PE=1 SV=1	METTL9	1.386	0.017861498	Up									S	KOG3987	Uncharacterized conserved protein DREV/CGI-81
O43463	Histone-lysine N-methyltransferase SUV39H1 OS=Homo sapiens OX=9606 GN=SUV39H1 PE=1 SV=1	SUV39H1	1.383	0.000229582	Up	K11419	SUV39H, CLR4; [histone H3]-lysine9 N-trimethyltransferase SUV39H [EC:2.1.1.355]	map00310 Lysine degradation; map01100 Metabolic pathways	GO:0000122 negative regulation of transcription from RNA polymerase II promoter; GO:0000183 chromatin silencing at rDNA; GO:0001666 response to hypoxia; GO:0006323 DNA packaging; GO:0006325 chromatin organization; GO:0006342 chromatin silencing;	GO:0000228 nuclear chromosome; GO:0000775 chromosome, centromeric region; GO:0000785 chromatin; GO:0000790 nuclear chromatin; GO:0000792 heterochromatin; GO:0000793 condensed chromosome; GO:0000794 condensed nuclear chromosome;	GO:0000976 transcription regulatory region sequence-specific DNA binding; GO:0000977 RNA polymerase II regulatory region sequence-specific DNA binding; GO:0001012 RNA polymerase II regulatory region DNA binding; GO:0001067 regulatory	PF00385	Chromo (CHRromatin Organisation MOdifier) domain	B	KOG1082	Histone H3 (Lys9) methyltransferase SUV39H1/Clr4, required for transcriptional silencing

Q96GC9	Vacuole membrane protein 1 OS=Homo sapiens OX=9606 GN=VMP1 PE=1 SV=1	VMP1	1.382	0.046869663	Up	K21248	VMP1; vacuole membrane protein 1	map04140 Autophagy - animal	GO:0000003 reproduction; GO:0006810 transport; GO:0006887 exocytosis; GO:0006914 autophagy; GO:0006996 organelle organization; GO:0007029 endoplasmic reticulum organization; GO:0007030 Golgi organization; GO:0007155 cell adhesion;	GO:0000407 pre-autophagosomal structure; GO:0000421 autophagosome membrane; GO:0005622 intracellular; GO:0005623 cell; GO:0005634 nucleus; GO:0005730 nucleolus; GO:0005737 cytoplasm; GO:0005773 vacuole; GO:0005774 vacuolar membrane; GO:00057				U	KOG1109	Vacuole membrane protein VMP1
Q2M296	Methenyltetrahydrofolate synthase domain-containing protein OS=Homo sapiens OX=9606 GN=MTHFSD PE=1 SV=2	MTHFSD	1.382	0.001778815	Up				GO:0006403 RNA localization; GO:0008298 intracellular mRNA localization; GO:0033036 macromolecule localization; GO:0051179 localization; GO:0051641 cellular localization; GO:0070727 cellular macromolecule localization.	GO:0005622 intracellular; GO:0005623 cell; GO:0005737 cytoplasm; GO:0044424 intracellular part; GO:0044464 cell part;		PF00076	RNA recognition motif. (a.k.a. RRM, RBD, or RNP domain)	H	KOG4410	5-formyltetrahydrofolate cyclo-ligase
P78346	Ribonuclease P protein subunit p30 OS=Homo sapiens OX=9606 GN=RPP30 PE=1 SV=1	RPP30	1.381	0.015000295	Up	K03539	RPP1, RPP30; ribonuclease P/MRP protein subunit RPP1 [EC:3.1.26.5]	map03008 Ribosome biogenesis in eukaryotes; map03013 RNA transport	GO:0000966 RNA 5'-end processing; GO:0001682 tRNA 5'-leader removal; GO:0006139 nucleobase-containing compound metabolic process; GO:0006396 RNA processing; GO:0006399 tRNA metabolic process; GO:0006725 cellular aromatic compound metabolic process; GO:0006807 nitrogen compound	GO:0000172 ribonuclease MRP complex; GO:0005622 intracellular; GO:0005623 cell; GO:0005634 nucleus; GO:0005654 nucleoplasm; GO:0005655 nucleolar ribonuclease P complex; GO:0005730 nucleolus; GO:0005732 small nucleolar ribonucleoprotein	GO:0003676 nucleic acid binding; GO:0003723 RNA binding; GO:0003824 catalytic activity; GO:0004518 nuclease activity; GO:0004519 endonuclease activity; GO:0004521 endoribonuclease activity; GO:0004526 ribonuclease P activity; GO:0004540 ribonucleas	PF01876	RNase P subunit p30	J	KOG2363	Protein subunit of nuclear ribonuclease P (RNase P)
Q9UNL4	Inhibitor of growth protein 4 OS=Homo sapiens OX=9606 GN=ING4 PE=1 SV=1	ING4	1.381	0.047863539	Up	K11346	ING4; inhibitor of growth protein 4		GO:0006139 nucleobase-containing compound metabolic process; GO:0006259 DNA metabolic process; GO:0006260 DNA replication; GO:0006325 chromatin organization; GO:0006355 regulation of transcription, DNA-templated; GO:0006464 cellular protein modification process;	GO:0000123 histone acetyltransferase complex; GO:0005622 intracellular; GO:0005623 cell; GO:0005634 nucleus; GO:0005654 nucleoplasm; GO:0005737 cytoplasm; GO:0005829 cytosol; GO:0005856 cytoskeleton; GO:0031248 protein acetyltransferase complex;	GO:0003712 transcription cofactor activity; GO:0003713 transcription coactivator activity; GO:0005488 binding; GO:0005515 protein binding; GO:0035064 methylated histone binding; GO:0042393 histone binding;	PF12998	Inhibitor of growth proteins N-terminal histone-binding	B	KOG1973	Chromatin remodeling protein, contains PHD Zn-finger
P02679	Fibrinogen gamma chain OS=Homo sapiens OX=9606 GN=FGG PE=1 SV=3	FGG	1.377	8.63666E-05	Up	K03905	FGG; fibrinogen gamma chain	map04610 Complement and coagulation cascades; map04611 Platelet activation; map05150 Staphylococcus aureus infection	GO:0001775 cell activation; GO:0001932 regulation of protein phosphorylation; GO:0001934 positive regulation of protein phosphorylation; GO:0002218 activation of innate immune response; GO:0002221 pattern recognition receptor signaling pathway; GO:0002224 toll-like receptor signaling	GO:0005576 extracellular region; GO:0005577 fibrinogen complex; GO:0005615 extracellular space; GO:0005622 intracellular; GO:0005623 cell; GO:0005737 cytoplasm; GO:0005783 endoplasmic reticulum; GO:0005788 endoplasmic reticulum lumen;	GO:0005102 receptor binding; GO:0005198 structural molecule activity; GO:0005488 binding; GO:0005515 protein binding; GO:0042802 identical protein binding; GO:0042803 protein homodimerization activity; GO:0046983 protein dimerization activity; GO:0050839 cell adhesion molecule	PF00147	Fibrinogen beta and gamma chains, C-terminal globular domain	S	KOG2579	Ficolin and related extracellular proteins

P00450	Ceruloplasmin OS=Homo sapiens OX=9606 GN=CP PE=1 SV=1	CP	1.374	0.014277946	Up	K13624	CP; ceruloplasmin [EC:1.16.3.1]	map00860 Porphyrin and chlorophyll metabolism; map04216 Ferroptosis	GO:0006464 cellular protein modification process; GO:0006807 nitrogen compound metabolic process; GO:0006873 cellular ion homeostasis; GO:0006875 cellular metal ion homeostasis; GO:0006879 cellular iron ion homeostasis; GO:0008152 metabolic process; GO:0009987 cellular	GO:0005576 extracellular region; GO:0005615 extracellular space; GO:0005622 intracellular; GO:0005623 cell; GO:0005737 cytoplasm; GO:0005783 endoplasmic reticulum; GO:0005788 endoplasmic reticulum lumen; GO:0012505 endomembrane	GO:0003824 catalytic activity; GO:0004322 ferroxidase activity; GO:0005488 binding; GO:0005515 protein binding; GO:0016491 oxidoreductase activity; GO:0016722 oxidoreductase activity, oxidizing metal ions; GO:0016724 oxidoreductase activity, oxidizing metal ions,	PF07732; PF00394; PF07731	Multicopper oxidase; Multicopper oxidase; Multicopper oxidase	Q	KOG1263	Multicopper oxidases
Q96GW9	Methionine--tRNA ligase, mitochondrial OS=Homo sapiens OX=9606 GN=MARS2 PE=1 SV=2	MARS2	1.372	0.023323932	Up	K01874	MARS, metG; methionyl-tRNA synthetase [EC:6.1.1.10]	map00450 Selenocompound metabolism; map00970 Aminoacyl-tRNA biosynthesis; map01100 Metabolic pathways	GO:0006082 organic acid metabolic process; GO:0006139 nucleobase-containing compound metabolic process; GO:0006399 tRNA metabolic process; GO:0006412 translation; GO:0006418 tRNA aminoacylation for protein translation; GO:0006431 methionyl-tRNA aminoacylation;	GO:0005622 intracellular; GO:0005623 cell; GO:0005737 cytoplasm; GO:0005739 mitochondrion; GO:0005759 mitochondrial matrix; GO:0031974 membrane-enclosed lumen; GO:0043226 organelle; GO:0043227 membrane-bounded organelle; GO:00432	GO:0003824 catalytic activity; GO:0004812 aminoacyl-tRNA ligase activity; GO:0004825 methionine-tRNA ligase activity; GO:0016874 ligase activity; GO:0016875 ligase activity, forming carbon-oxygen bonds;			J	KOG0436	Methionyl-tRNA synthetase
Q92597	Protein NDRG1 OS=Homo sapiens OX=9606 GN=NDRG1 PE=1 SV=1	NDRG1	1.371	0.034758047	Up	K18266	NDRG1; protein NDRG1		GO:0001666 response to hypoxia; GO:0001775 cell activation; GO:0002274 myeloid leukocyte activation; GO:0002376 immune system process; GO:0006950 response to stress; GO:0006974 cellular response to DNA damage stimulus; GO:0007009 plasma membrane organization; GO:00071	GO:0005622 intracellular; GO:0005623 cell; GO:0005634 nucleus; GO:0005737 cytoplasm; GO:0005768 endosome; GO:0005813 centrosome; GO:0005815 microtubule organizing center; GO:0005829 cytosol; GO:0005856 cytoskeleton; GO:00058	GO:0005488 binding; GO:0005515 protein binding; GO:0008017 microtubule binding; GO:0008092 cytoskeletal protein binding; GO:0015631 tubulin binding; GO:0017016 Ras GTPase binding; GO:0017137 Rab GTPase binding; GO:0019899 enzyme binding; GO:0031267 small GTPase	PF03096	Ndr family	S	KOG2931	Differentiation-related gene 1 protein (NDR1 protein), related proteins
Q9BQ69	ADP-ribose glycohydrolase MACROD1 OS=Homo sapiens OX=9606 GN=MACROD1 PE=1 SV=2	MACROD1	1.367	0.029200676	Up	K23518	MACROD, ymdB; O-acetyl-ADP-ribose deacetylase [EC:3.1.1.106]		GO:0006139 nucleobase-containing compound metabolic process; GO:0006464 cellular protein modification process; GO:0006725 cellular aromatic compound metabolic process; GO:0006807 nitrogen compound metabolic process; GO:0006950 response to stress; GO:0006974 cellular response to DNA	GO:0005622 intracellular; GO:0005623 cell; GO:0005634 nucleus; GO:0005654 nucleoplasm; GO:0031974 membrane-enclosed lumen; GO:0031981 nuclear lumen; GO:0043226 organelle; GO:0043227 membrane-bounded organelle; GO:0043229 intracellula	GO:0003824 catalytic activity; GO:0016787 hydrolase activity; GO:0016798 hydrolase activity, acting on glycosyl bonds; GO:0019213 deacetylase activity;	PF01661	Macro domain	BK	KOG2633	Hismacro and SEC14 domain-containing proteins
Q9Y3D8	Adenylate kinase isoenzyme 6 OS=Homo sapiens OX=9606 GN=AK6 PE=1 SV=1	AK6	1.366	0.039969312	Up	K18532	AK6, FAP7; adenylate kinase [EC:2.7.4.3]	map00230 Purine metabolism; map01100 Metabolic pathways; map03008 Ribosome biogenesis in eukaryotes	GO:0000491 small nucleolar ribonucleoprotein complex assembly; GO:0000492 box C/D snoRNP assembly; GO:0001558 regulation of cell growth; GO:0006139 nucleobase-containing compound metabolic process; GO:0006325 chromatin organization; GO:0006351 transcriptio	GO:0000123 histone acetyltransferase complex; GO:0000124 SAGA complex; GO:0000125 PCAF complex; GO:0000428 DNA-directed RNA polymerase complex; GO:0005622 intracellular; GO:0005623 cell; GO:0005634 nucleus; GO:0005654 nucleoplasm; GO:0005667	GO:0000166 nucleotide binding; GO:0001067 regulatory region nucleic acid binding; GO:0002039 p53 binding; GO:0003676 nucleic acid binding; GO:0003677 DNA binding; GO:0003712 transcription cofactor activity; GO:0003713 transcription coactivator	PF13238	AAA domain	F	KOG3347	Predicted nucleotide kinase/nuclear protein involved oxidative stress response

P04062	Lysosomal acid glucosylceramidase OS=Homo sapiens OX=9606 GN=GBA PE=1 SV=3	GBA	1.365	0.031302208	Up	K01201	GBA, srfJ; glucosylceramidase [EC:3.2.1.45]	map00511 Other glycan degradation; map00600 Sphingolipid metabolism; map01100 Metabolic pathways; map04142 Lysosome	GO:0001817 regulation of cytokine production; GO:0001818 negative regulation of cytokine production; GO:0001932 regulation of protein phosphorylation; GO:0001933 negative regulation of protein phosphorylation; GO:0006066 alcohol metabolic process; GO:0006469 negative regulation of protein kinase	GO:0000323 lytic vacuole; GO:0005576 extracellular region; GO:0005615 extracellular space; GO:0005622 intracellular; GO:0005623 cell; GO:0005737 cytoplasm; GO:0005764 lysosome; GO:0005765 lysosomal membrane; GO:0005773 vacuole; GO:0005774	GO:0003824 catalytic activity; GO:0004348 glucosylceramidase activity; GO:0004553 hydrolase activity, hydrolyzing O-glycosyl compounds; GO:0005102 receptor binding; GO:0005124 scavenger receptor binding; GO:0005488 binding; GO:0005515 protein binding; GO:00167	PF02055	O-Glycosyl hydrolase family 30	G	KOG2566	Beta-glucocerebrosidase
O14733	Dual specificity mitogen-activated protein kinase kinase 7 OS=Homo sapiens OX=9606 GN=MAP2K7 PE=1 SV=2	MAP2K7	1.365	0.044857407	Up	K04431	MAP2K7, MKK7; mitogen-activated protein kinase kinase 7 [EC:2.7.12.2]	map04010 MAPK signaling pathway; map04012 ErbB signaling pathway; map04013 MAPK signaling pathway - fly; map04141 Protein processing in endoplasmic reticulum; map04214 Apoptosis - fly; map04361 Axon regeneration; map04380 Osteoclast differentiation; map04530 Tight junction; map04620 Toll-like receptor signaling pathway; map04624 Toll and Imd signaling pathway; map04660 T cell receptor signaling pathway; map04664 Fc epsilon RI signaling pathway; map04668 TNF signaling pathway; map04722 Neurotrophin signaling pathway; map04912 GnRH signaling pathway; map04926 Relaxin signaling pathway; map05135 Yersinia infection; map05161 Hepatitis B; map05167 Kaposi sarcoma-associated herpesvirus infection; map05169 Epstein-Barr virus infection; map05170 Human immunodeficiency virus 1 infection; map05418 Fluid shear stress and atherosclerosis	GO:0000165 MAPK cascade; GO:0000187 activation of MAPK activity; GO:0001932 regulation of protein phosphorylation; GO:0001934 positive regulation of protein phosphorylation; GO:0006464 cellular protein modification process; GO:0006468 protein phosphorylation; GO:0006793	GO:0005622 intracellular; GO:0005623 cell; GO:0005634 nucleus; GO:0005737 cytoplasm; GO:0005829 cytosol; GO:0043226 organelle; GO:0043227 membrane-bounded organelle; GO:0043229 intracellular organelle; GO:0043231 intracellular membrane-bounded	GO:0000166 nucleotide binding; GO:0000287 magnesium ion binding; GO:0003824 catalytic activity; GO:0004672 protein kinase activity; GO:0004674 protein serine/threonine kinase activity; GO:0004708 MAP kinase kinase activity; GO:0004712 protein serine/threonine/tyros	PF00069	Protein kinase domain	T	KOG0983	Mitogen-activated protein kinase (MAPK) kinase MKK7/JNKK2
Q8WXI4	Acyl-coenzyme A thioesterase 11 OS=Homo sapiens OX=9606 GN=ACOT11 PE=1 SV=1	ACOT11	1.365	0.034953441	Up	K12417	ACOT11; acyl-CoA thioesterase 11 [EC:3.1.2.-]		GO:0006082 organic acid metabolic process; GO:0006139 nucleobase-containing compound metabolic process; GO:0006163 purine nucleotide metabolic process; GO:0006629 lipid metabolic process; GO:0006631 fatty acid metabolic process; GO:0006637 acyl-CoA metabolic	GO:0005622 intracellular; GO:0005623 cell; GO:0005737 cytoplasm; GO:0005829 cytosol; GO:0044424 intracellular part; GO:0044444 cytoplasmic part; GO:0044464 cell part;	GO:0003824 catalytic activity; GO:0016289 CoA hydrolase activity; GO:0016787 hydrolase activity; GO:0016788 hydrolase activity, acting on ester bonds; GO:0016790 thiolester hydrolase activity; GO:0047617 acyl-CoA hydrolase activity;	PF03061; PF01852	Thioesterase superfamily; START domain	I	KOG2763	Acyl-CoA thioesterase
Q9Y3R5	Protein dopey-2 OS=Homo sapiens OX=9606 GN=DOP1B PE=1 SV=5	DOP1B	1.364	0.040974965	Up				GO:0003008 system process; GO:0006810 transport; GO:0006892 post-Golgi vesicle-mediated transport; GO:0006895 Golgi to endosome transport; GO:0006996 organelle organization; GO:0007029 endoplasmic reticulum organization; GO:0007275 multicellular	GO:0000139 Golgi membrane; GO:0005622 intracellular; GO:0005623 cell; GO:0005737 cytoplasm; GO:0005768 endosome; GO:0005794 Golgi apparatus; GO:0005802 trans-Golgi network; GO:0012505 endomembrane system; GO:0016020 membrane; GO:00310				U	KOG3613	Dopey and related predicted leucine zipper transcription factors
Q8N4L2	Type 2 phosphatidylinositol 4,5-bisphosphate 4-phosphatase OS=Homo sapiens OX=9606 GN=PIP4P2 PE=1 SV=1	PIP4P2	1.358	0.001222512	Up	K13084	TMEM55; phosphatidylinositol-4,5-bisphosphate 4-phosphatase [EC:3.1.3.78]	map04070 Phosphatidylinositol signaling system	GO:0006629 lipid metabolic process; GO:0006644 phospholipid metabolic process; GO:0006650 glycerophospholipid metabolic process; GO:0006793 phosphorus metabolic process; GO:0006796 phosphate-containing compound metabolic process; GO:0008152 metabolic	GO:0000323 lytic vacuole; GO:0005622 intracellular; GO:0005623 cell; GO:0005737 cytoplasm; GO:0005764 lysosome; GO:0005765 lysosomal membrane; GO:0005768 endosome; GO:0005770 late endosome; GO:0005773 vacuole; GO:0005774 vacuolar membrane;	GO:0003824 catalytic activity; GO:0016787 hydrolase activity; GO:0016788 hydrolase activity, acting on ester bonds; GO:0016791 phosphatase activity; GO:0034593 phosphatidylinositol bisphosphate phosphatase activity; GO:0034596 phosphatidylinositol phosphate			S	KOG4684	Uncharacterized conserved protein, contains C4-type Zn-finger

Q9Y6U3	Adseverin OS=Homo sapiens OX=9606 GN=SCIN PE=1 SV=4	SCIN	1.356	0.003543466	Up	K05768	GSN; gelsolin	map04666 Fc gamma R-mediated phagocytosis; map04810 Regulation of actin cytoskeleton; map05203 Viral carcinogenesis	GO:0002682 regulation of immune system process; GO:0002684 positive regulation of immune system process; GO:0006810 transport; GO:0006887 exocytosis; GO:0006996 organelle organization; GO:0007010 cytoskeleton organization; GO:0007015 actin	GO:0005622 intracellular; GO:0005623 cell; GO:0005737 cytoplasm; GO:0005886 plasma membrane; GO:0005903 brush border; GO:0005938 cell cortex; GO:0016020 membrane; GO:0032991 macromolecular complex; GO:0044424 intracellular part; GO:00444	GO:0001786 phosphatidylserine binding; GO:0003779 actin binding; GO:0005488 binding; GO:0005509 calcium ion binding; GO:0005515 protein binding; GO:0005543 phospholipid binding; GO:0005545 1-phosphatidylinositol binding; GO:0005546 phosphatidylinositol-4,5-	PF00626	Gelsolin repeat	Z	KOG0443	Actin regulatory proteins (gelsolin/villin family)
Q15118	[Pyruvate dehydrogenase (acetyl-transferring)] kinase isozyme 1, mitochondrial OS=Homo sapiens OX=9606 GN=PDK1 PE=1 SV=1	PDK1	1.356	0.018835363	Up	K12077	PDK1; pyruvate dehydrogenase kinase isoform 1 [EC:2.7.11.2]	map04066 HIF-1 signaling pathway; map04360 Axon guidance; map05230 Central carbon metabolism in cancer	GO:0001666 response to hypoxia; GO:0005975 carbohydrate metabolic process; GO:0005996 monosaccharide metabolic process; GO:0006006 glucose metabolic process; GO:0006109 regulation of carbohydrate metabolic process; GO:0006140 regulation	GO:0005622 intracellular; GO:0005623 cell; GO:0005634 nucleus; GO:0005730 nucleolus; GO:0005737 cytoplasm; GO:0005739 mitochondrion; GO:0005759 mitochondrial matrix; GO:0005886 plasma membrane; GO:0005967 mitochondrial pyruvate dehydroge	GO:0000166 nucleotide binding; GO:0003824 catalytic activity; GO:0004672 protein kinase activity; GO:0004674 protein serine/threonine kinase activity; GO:0004740 pyruvate dehydrogenase (acetyl-transferring) kinase activity; GO:0005488 binding; GO:0005515 protein binding;	PF02518	Histidine kinase-, DNA gyrase B-, and HSP90-like ATPase	T	KOG0787	Dehydrogenase kinase
P33897	ATP-binding cassette sub-family D member 1 OS=Homo sapiens OX=9606 GN=ABCD1 PE=1 SV=2	ABCD1	1.355	0.024304423	Up	K05675	ABCD1, ALD; ATP-binding cassette, subfamily D (ALD), member 1	map02010 ABC transporters; map04146 Peroxisome	GO:0000038 very long-chain fatty acid metabolic process; GO:0001676 long-chain fatty acid metabolic process; GO:0006082 organic acid metabolic process; GO:0006629 lipid metabolic process; GO:0006631 fatty acid metabolic process; GO:0006635 fatty acid beta-oxidation; GO:00068	GO:0005622 intracellular; GO:0005623 cell; GO:0005737 cytoplasm; GO:0005777 peroxisome; GO:0005778 peroxisomal membrane; GO:0005779 integral component of peroxisomal membrane; GO:0005829 cytosol; GO:0016020 membrane; GO:00160	GO:0000166 nucleotide binding; GO:0003824 catalytic activity; GO:0005215 transporter activity; GO:0005319 lipid transporter activity; GO:0005324 long-chain fatty acid transporter activity; GO:0005488 binding; GO:0005515 protein binding; GO:0005524 ATP binding; GO:0008144 drug	PF00005	ABC transporter	I	KOG0064	Peroxisomal long-chain acyl-CoA transporter, ABC superfamily
Q58F21	Bromodomain testis-specific protein OS=Homo sapiens OX=9606 GN=BRDT PE=1 SV=4	BRDT	1.355	0.032796467	Up	K11724	BRDT; bromodomain testis-specific protein		GO:0000003 reproduction; GO:0000280 nuclear division; GO:0001207 histone displacement; GO:0006325 chromatin organization; GO:0006338 chromatin remodeling; GO:0006355 regulation of transcription, DNA-templated; GO:0006996 organelle	GO:0005622 intracellular; GO:0005623 cell; GO:0005634 nucleus; GO:0043226 organelle; GO:0043227 membrane-bounded organelle; GO:0043229 intracellular organelle; GO:0043231 intracellular membrane-bounded organelle; GO:0044424 intracellular part;	GO:0003712 transcription cofactor activity; GO:0003713 transcription coactivator activity; GO:0005488 binding; GO:0005515 protein binding; GO:0042393 histone binding; GO:0070577 lysine-acetylated histone binding;	PF00439	Bromodomain	K	KOG1474	Transcription initiation factor TFIID, subunit BDF1 and related bromodomain proteins
P61009	Signal peptidase complex subunit 3 OS=Homo sapiens OX=9606 GN=SPCS3 PE=1 SV=1	SPCS3	1.355	0.040684731	Up	K12948	SPCS3, SPC3; signal peptidase complex subunit 3 [EC:3.4.-.-]	map03060 Protein export	GO:0006465 signal peptide processing; GO:0006508 proteolysis; GO:0006518 peptide metabolic process; GO:0006605 protein targeting; GO:0006807 nitrogen compound metabolic process; GO:0006810 transport; GO:0006886 intracellular protein transport; GO:0008104 protein localization;	GO:0005622 intracellular; GO:0005623 cell; GO:0005737 cytoplasm; GO:0005783 endoplasmic reticulum; GO:0005787 signal peptidase complex; GO:0005789 endoplasmic reticulum membrane; GO:0012505 endomembrane system; GO:0016020 membrane;	GO:0003824 catalytic activity; GO:0008233 peptidase activity; GO:0016787 hydrolase activity;			U	KOG3372	Signal peptidase complex subunit

Q86Y79	Probable peptidyl-tRNA hydrolase OS=Homo sapiens OX=9606 GN=PTRH1 PE=1 SV=1	PTRH1	1.354	0.003692582	Up	K01056	PTH1, pth, spoVC; peptidyl-tRNA hydrolase, PTH1 family [EC:3.1.1.29]				GO:0003824 catalytic activity; GO:0004045 aminoacyl-tRNA hydrolase activity; GO:0016787 hydrolase activity; GO:0016788 hydrolase activity, acting on ester bonds; GO:0052689 carboxylic ester hydrolase activity;	PF01195	Peptidyl-tRNA hydrolase	J	KOG2255	Peptidyl-tRNA hydrolase
P49788	Retinoic acid receptor responder protein 1 OS=Homo sapiens OX=9606 GN=RARRES1 PE=1 SV=2	RARRES1	1.354	0.001326884	Up	K23595	RARRES1; retinoic acid receptor responder protein 1		GO:0008285 negative regulation of cell proliferation; GO:0042127 regulation of cell proliferation; GO:0048519 negative regulation of biological process; GO:0048523 negative regulation of cellular process; GO:0050789 regulation of biological process; GO:0050794	GO:0016020 membrane; GO:0016021 integral component of membrane; GO:0031224 intrinsic component of membrane; GO:0044425 membrane part;						
O43683	Mitotic checkpoint serine/threonine-protein kinase BUB1 OS=Homo sapiens OX=9606 GN=BUB1 PE=1 SV=1	BUB1	1.353	0.041338651	Up	K02178	BUB1; checkpoint serine/threonine-protein kinase [EC:2.7.11.1]	map04110 Cell cycle; map04114 Oocyte meiosis; map04914 Progesterone-mediated oocyte maturation	GO:0000003 reproduction; GO:0000075 cell cycle checkpoint; GO:0000278 mitotic cell cycle; GO:0000280 nuclear division; GO:0000819 sister chromatid segregation; GO:0006464 cellular protein modification process; GO:0006468 protein phosphorylation; GO:0006793 phosphorus	GO:0000228 nuclear chromosome; GO:0000775 chromosome, centromeric region; GO:0000776 kinetochore; GO:0000777 condensed chromosome kinetochore; GO:0000778 condensed nuclear chromosome kinetochore; GO:0000779	GO:0003824 catalytic activity; GO:0004672 protein kinase activity; GO:0016301 kinase activity; GO:0016740 transferase activity; GO:0016772 transferase activity, transferring phosphorus-containing groups; GO:0016773 phosphotransferase activity, alcohol group as acceptor;	PF08311; PF00069	Mad3/BUB1 homology region 1; Protein kinase domain	D	KOG1166	Mitotic checkpoint serine/threonine protein kinase
Q4ZG55	Protein GREB1 OS=Homo sapiens OX=9606 GN=GREB1 PE=2 SV=1	GREB1	1.352	0.045216133	Up					GO:0005622 intracellular; GO:0005623 cell; GO:0005634 nucleus; GO:0005654 nucleoplasm; GO:0031974 membrane-enclosed lumen; GO:0031981 nuclear lumen; GO:0043226 organelle; GO:0043227 membrane-bounded organelle; GO:0043229 intracellula						
Q7Z3U7	Protein MON2 homolog OS=Homo sapiens OX=9606 GN=MON2 PE=1 SV=3	MON2	1.352	0.040541302	Up				GO:0006810 transport; GO:0006892 post-Golgi vesicle-mediated transport; GO:0006895 Golgi to endosome transport; GO:0016192 vesicle-mediated transport; GO:0016482 cytosolic transport; GO:0046907 intracellular transport; GO:0048193 Golgi vesicle transport; GO:0051179			PF12783	Guanine nucleotide exchange factor in Golgi transport N-terminal	U	KOG1848	Uncharacterized conserved protein

Q08722	Leukocyte surface antigen CD47 OS=Homo sapiens OX=9606 GN=CD47 PE=1 SV=1	CD47	1.348	0.022949558	Up	K06266	CD47; CD47 antigen (Rh-related antigen, integrin-associated signal transducer)	map04512 ECM-receptor interaction	GO:0001775 cell activation; GO:0002252 immune effector process; GO:0002263 cell activation involved in immune response; GO:0002274 myeloid leukocyte activation; GO:0002275 myeloid cell activation involved in immune response; GO:0002283 neutrophil activation involved in immune response;	GO:0005576 extracellular region; GO:0005615 extracellular space; GO:0005622 intracellular; GO:0005623 cell; GO:0005737 cytoplasm; GO:0005886 plasma membrane; GO:0005887 integral component of plasma membrane; GO:0012505 endomembrane system; GO:00125	GO:0038023 signaling receptor activity; GO:0060089 molecular transducer activity; GO:0070053 thrombospondin receptor activity;	PF08204	CD47 immunoglobulin-like domain			
P07602	Prosaposin OS=Homo sapiens OX=9606 GN=PSAP PE=1 SV=2	PSAP	1.346	0.000275799	Up	K12382	PSAP, SGP1; saposin	map04142 Lysosome	GO:0000003 reproduction; GO:0001655 urogenital system development; GO:0001775 cell activation; GO:0001932 regulation of protein phosphorylation; GO:0001934 positive regulation of protein phosphorylation; GO:0002252 immune effector process; GO:0002263 cell	GO:0000323 lytic vacuole; GO:0005576 extracellular region; GO:0005615 extracellular space; GO:0005622 intracellular; GO:0005623 cell; GO:0005737 cytoplasm; GO:0005764 lysosome; GO:0005765 lysosomal membrane; GO:0005766 primary lysosome; GO:00057	GO:0001664 G-protein coupled receptor binding; GO:0003824 catalytic activity; GO:0004553 hydrolase activity, hydrolyzing O-glycosyl compounds; GO:0004565 beta-galactosidase activity; GO:0005102 receptor binding; GO:0005488 binding; GO:0005515 protein binding; GO:00055	PF05184	Saposin-like type B, region 1	GI	KOG1340	Prosaposin
Q14165	Malectin OS=Homo sapiens OX=9606 GN=MLEC PE=1 SV=1	MLEC	1.342	0.030307633	Up				GO:0001775 cell activation; GO:0002252 immune effector process; GO:0002263 cell activation involved in immune response; GO:0002274 myeloid leukocyte activation; GO:0002275 myeloid cell activation involved in immune response; GO:0002283 neutrophil activation involved in immune response;	GO:0005622 intracellular; GO:0005623 cell; GO:0005737 cytoplasm; GO:0005783 endoplasmic reticulum; GO:0005789 endoplasmic reticulum membrane; GO:0005886 plasma membrane; GO:0012505 endomembrane system; GO:0012506 vesicle membrane; GO:00160	GO:0005488 binding; GO:0005515 protein binding; GO:0019899 enzyme binding; GO:0030246 carbohydrate binding;	PF11721	Di-glucose binding within endoplasmic reticulum	U	KOG3593	Predicted receptor-like serine/threonine kinase
O00483	Cytochrome c oxidase subunit NDUFA4 OS=Homo sapiens OX=9606 GN=NDUFA4 PE=1 SV=1	NDUFA4	1.341	0.01990211	Up	K03948	NDUFA4; NADH dehydrogenase (ubiquinone) 1 alpha subcomplex subunit 4	map00190 Oxidative phosphorylation; map01100 Metabolic pathways; map04714 Thermogenesis; map04723 Retrograde endocannabinoid signaling; map04932 Non-alcoholic fatty liver disease (NAFLD); map05010 Alzheimer disease; map05012 Parkinson disease; map05016 Huntington disease	GO:0006091 generation of precursor metabolites and energy; GO:0006119 oxidative phosphorylation; GO:0006120 mitochondrial electron transport, NADH to ubiquinone; GO:0006139 nucleobase-containing compound metabolic process; GO:0006163 purine nucleotide metabolic	GO:0005622 intracellular; GO:0005623 cell; GO:0005737 cytoplasm; GO:0005739 mitochondrion; GO:0005740 mitochondrial envelope; GO:0005743 mitochondrial inner membrane; GO:0005746 mitochondrial respiratory chain; GO:0005747	GO:0003824 catalytic activity; GO:0003954 NADH dehydrogenase activity; GO:0005488 binding; GO:0008137 NADH dehydrogenase (ubiquinone) activity; GO:0016491 oxidoreductase activity; GO:0016651 oxidoreductase activity, acting on NAD(P)H; GO:0016655 oxidoreduc					
Q15714	TSC22 domain family protein 1 OS=Homo sapiens OX=9606 GN=TSC22D1 PE=1 SV=3	TSC22D1	1.34	0.043786969	Up				GO:0006139 nucleobase-containing compound metabolic process; GO:0006351 transcription, DNA-templated; GO:0006355 regulation of transcription, DNA-templated; GO:0006357 regulation of transcription from RNA polymerase II promoter; GO:0006366	GO:0005622 intracellular; GO:0005623 cell; GO:0005634 nucleus; GO:0005737 cytoplasm; GO:0043226 organelle; GO:0043227 membrane-bounded organelle; GO:0043229 intracellular organelle; GO:0043231 intracellular membrane-bounded organelle; GO:00444	GO:0000976 transcription regulatory region sequence-specific DNA binding; GO:0000977 RNA polymerase II regulatory region sequence-specific DNA binding; GO:0000978 RNA polymerase II core promoter proximal region sequence-specific DNA binding;			K	KOG4797	Transcriptional regulator

Q7KZN9	Cytochrome c oxidase assembly protein COX15 homolog OS=Homo sapiens OX=9606 GN=COX15 PE=1 SV=1	COX15	1.338	0.037267685	Up	K02259	COX15, ctaA; cytochrome c oxidase assembly protein subunit 15	map00190 Oxidative phosphorylation; map00860 Porphyrin and chlorophyll metabolism; map01100 Metabolic pathways; map04714 Thermogenesis	GO:0006091 generation of precursor metabolites and energy; GO:0006119 oxidative phosphorylation; GO:0006123 mitochondrial electron transport, cytochrome c to oxygen; GO:0006139 nucleobase-containing compound metabolic process; GO:0006163 purine nucleotide metabolic	GO:0005622 intracellular; GO:0005623 cell; GO:0005634 nucleus; GO:0005737 cytoplasm; GO:0005739 mitochondrion; GO:0005740 mitochondrial envelope; GO:0005743 mitochondrial inner membrane; GO:0005746 mitochondrial respiratory chain;	GO:0003824 catalytic activity; GO:0004129 cytochrome-c oxidase activity; GO:0005215 transporter activity; GO:0008324 cation transmembrane transporter activity; GO:0009055 electron carrier activity; GO:0015002 heme-copper terminal oxidase activity; GO:0015075 ion transmemb			C	KOG2725	Cytochrome oxidase assembly factor COX15
Q92552	28S ribosomal protein S27, mitochondrial OS=Homo sapiens OX=9606 GN=MRPS27 PE=1 SV=3	MRPS27	1.338	0.037478895	Up	K17406	MRPS27; small subunit ribosomal protein S27		GO:0006412 translation; GO:0006414 translational elongation; GO:0006415 translational termination; GO:0006417 regulation of translation; GO:0006518 peptide metabolic process; GO:0006807 nitrogen compound metabolic process; GO:0008152 metabolic	GO:0000313 organellar ribosome; GO:0000314 organellar small ribosomal subunit; GO:0005622 intracellular; GO:0005623 cell; GO:0005737 cytoplasm; GO:0005739 mitochondrion; GO:0005740 mitochondrial envelope; GO:0005743 mitochondr	GO:0000049 tRNA binding; GO:0003676 nucleic acid binding; GO:0003723 RNA binding; GO:0005488 binding; GO:0019843 rRNA binding; GO:0043021 ribonucleoprotein complex binding; GO:0043022 ribosome binding; GO:0044877 macromolecular complex binding;			J	KOG4570	Uncharacterized conserved protein
Q8N2Z9	Centromere protein S OS=Homo sapiens OX=9606 GN=CENPS PE=1 SV=1	CENPS	1.335	0.029835987	Up	K11511	APITD1, CENPS, MHF1; centromere protein S	map03460 Fanconi anemia pathway	GO:0000003 reproduction; GO:0000280 nuclear division; GO:0000712 resolution of meiotic recombination intermediates; GO:0006139 nucleobase-containing compound metabolic process; GO:0006259 DNA metabolic process; GO:0006260 DNA replication; GO:0006261 DNA-	GO:0005622 intracellular; GO:0005623 cell; GO:0005634 nucleus; GO:0005654 nucleoplasm; GO:0005737 cytoplasm; GO:0005829 cytosol; GO:0031974 membrane-enclosed lumen; GO:0031981 nuclear lumen; GO:0032991 macromolecular complex; GO:00432	GO:0003676 nucleic acid binding; GO:0003677 DNA binding; GO:0003682 chromatin binding; GO:0003690 double-stranded DNA binding; GO:0005488 binding; GO:0097159 organic cyclic compound binding; GO:1901363 heterocyclic compound binding;					
Q70CQ3	Ubiquitin carboxyl-terminal hydrolase 30 OS=Homo sapiens OX=9606 GN=USP30 PE=1 SV=1	USP30	1.335	0.027028641	Up	K11851	USP30; ubiquitin carboxyl-terminal hydrolase 30 [EC:3.4.19.12]	map04137 Mitophagy - animal	GO:0000422 mitophagy; GO:0006464 cellular protein modification process; GO:0006508 proteolysis; GO:0006807 nitrogen compound metabolic process; GO:0006914 autophagy; GO:0006996 organelle organization; GO:0007005 mitochondrion organization; GO:00080	GO:0005622 intracellular; GO:0005623 cell; GO:0005737 cytoplasm; GO:0005739 mitochondrion; GO:0005740 mitochondrial envelope; GO:0005741 mitochondrial outer membrane; GO:0016020 membrane; GO:0019867 outer membrane; GO:0031090	GO:0003824 catalytic activity; GO:0004175 endopeptidase activity; GO:0004197 cysteine-type endopeptidase activity; GO:0004843 thiol-dependent ubiquitin-specific protease activity; GO:0008233 peptidase activity; GO:0008234 cysteine-type peptidase			O	KOG1867	Ubiquitin-specific protease
O14773	Tripeptidyl-peptidase 1 OS=Homo sapiens OX=9606 GN=TPP1 PE=1 SV=2	TPP1	1.334	0.012965987	Up	K01279	TPP1, CLN2; tripeptidyl-peptidase I [EC:3.4.14.9]	map04142 Lysosome	GO:0001894 tissue homeostasis; GO:0003008 system process; GO:0006508 proteolysis; GO:0006518 peptide metabolic process; GO:0006629 lipid metabolic process; GO:0006807 nitrogen compound metabolic process; GO:0006950 response to stress; GO:0006986 response to unfolded	GO:0000323 lytic vacuole; GO:0005622 intracellular; GO:0005623 cell; GO:0005737 cytoplasm; GO:0005764 lysosome; GO:0005773 vacuole; GO:0005775 vacuolar lumen; GO:0031974 membrane-enclosed lumen; GO:0043202 lysosomal lumen; GO:00432	GO:0003824 catalytic activity; GO:0004175 endopeptidase activity; GO:0005488 binding; GO:0008233 peptidase activity; GO:0008236 serine-type peptidase activity; GO:0008238 exopeptidase activity; GO:0008240 tripeptidyl-peptidase activity; GO:0016787 hydrolase	PF09286	Pro-kumamolisin, activation domain			

P24941	Cyclin-dependent kinase 2 OS=Homo sapiens OX=9606 GN=CDK2 PE=1 SV=2	CDK2	1.332	0.005819535	Up	K02206	CDK2; cyclin-dependent kinase 2 [EC:2.7.11.22]	map04068 FoxO signaling pathway; map04110 Cell cycle; map04114 Oocyte meiosis; map04115 p53 signaling pathway; map04151 PI3K-Akt signaling pathway; map04218 Cellular senescence; map04914 Progesterone-mediated oocyte maturation; map04934 Cushing syndrome; map05160 Hepatitis C; map05161 Hepatitis B; map05162 Measles; map05165 Human papillomavirus infection; map05166 Human T-cell leukemia virus 1 infection; map05169 Epstein-Barr virus infection; map05200 Pathways in cancer; map05203 Viral carcinogenesis; map05215 Prostate cancer; map05222 Small cell lung cancer; map05226 Gastric cancer	GO:0000003 reproduction; GO:0000075 cell cycle checkpoint; GO:0000077 DNA damage checkpoint; GO:0000082 G1/S transition of mitotic cell cycle; GO:0000086 G2/M transition of mitotic cell cycle; GO:0000226 microtubule cytoskeleton organization; GO:00002	GO:0000307 cyclin-dependent protein kinase holoenzyme complex; GO:0005622 intracellular; GO:0005623 cell; GO:0005634 nucleus; GO:0005654 nucleoplasm; GO:0005737 cytoplasm; GO:0005768 endosome; GO:0005813 centrosome; GO:0005815	GO:0003824 catalytic activity; GO:0004672 protein kinase activity; GO:0004674 protein serine/threonine kinase activity; GO:0004693 cyclin-dependent protein serine/threonine kinase activity; GO:0005488 binding; GO:0005515 protein binding; GO:0016301 kinase activity; GO:0016740	PF00069	Protein kinase domain	T	KOG0594	Protein kinase PCTAIRE and related kinases
Q9BWJ5	Splicing factor 3B subunit 5 OS=Homo sapiens OX=9606 GN=SF3B5 PE=1 SV=1	SF3B5	1.332	0.033220593	Up	K12832	SF3B5, SF3B10; splicing factor 3B subunit 5	map03040 Spliceosome	GO:0000375 RNA splicing, via transesterification reactions; GO:0000377 RNA splicing, via transesterification reactions with bulged adenosine as nucleophile; GO:0000398 mRNA splicing, via spliceosome; GO:0006139 nucleobase-containing compound	GO:0005622 intracellular; GO:0005623 cell; GO:0005634 nucleus; GO:0005654 nucleoplasm; GO:0005681 spliceosomal complex; GO:0005684 U2-type spliceosomal complex; GO:0005686 U2 snRNP; GO:0005689 U12-type spliceosomal complex; GO:0030532 small nuclear				A	KOG3485	Uncharacterized conserved protein
Q9BWW4	Single-stranded DNA-binding protein 3 OS=Homo sapiens OX=9606 GN=SSBP3 PE=1 SV=1	SSBP3	1.332	0.003143262	Up				GO:0002244 hematopoietic progenitor cell differentiation; GO:0002376 immune system process; GO:0002520 immune system development; GO:0003002 regionalization; GO:0006139 nucleobase-containing compound metabolic process; GO:0006351 transcriptio	GO:0005622 intracellular; GO:0005623 cell; GO:0005634 nucleus; GO:0032991 macromolecular complex; GO:0043226 organelle; GO:0043227 membrane-bounded organelle; GO:0043229 intracellular organelle; GO:0043231 intracellular membrane-bounded	GO:0000976 transcription regulatory region sequence-specific DNA binding; GO:0000977 RNA polymerase II regulatory region sequence-specific DNA binding; GO:0000978 RNA polymerase II core promoter proximal region sequence-specific DNA binding;			K	KOG4594	Sequence-specific single-stranded-DNA-binding protein
Q9BYC8	39S ribosomal protein L32, mitochondrial OS=Homo sapiens OX=9606 GN=MRPL32 PE=1 SV=1	MRPL32	1.331	0.00425515	Up	K02911	RP-L32, MRPL32, rpmF; large subunit ribosomal protein L32	map03010 Ribosome	GO:0006412 translation; GO:0006414 translational elongation; GO:0006415 translational termination; GO:0006518 peptide metabolic process; GO:0006807 nitrogen compound metabolic process; GO:0008152 metabolic process; GO:0009058 biosynthetic process;	GO:0000313 organellar ribosome; GO:0000315 organellar large ribosomal subunit; GO:0005622 intracellular; GO:0005623 cell; GO:0005737 cytoplasm; GO:0005739 mitochondrion; GO:0005740 mitochondrial envelope; GO:0005743 mitochondr	GO:0003735 structural constituent of ribosome; GO:0005198 structural molecule activity;			J	KOG4080	Mitochondrial ribosomal protein L32
P55199	RNA polymerase II elongation factor ELL OS=Homo sapiens OX=9606 GN=ELL PE=1 SV=1	ELL	1.331	0.046297769	Up	K15183	ELL; RNA polymerase II elongation factor ELL		GO:0001701 in utero embryonic development; GO:0006139 nucleobase-containing compound metabolic process; GO:0006351 transcription, DNA-templated; GO:0006354 DNA-templated transcription, elongation; GO:0006355 regulation of transcription, DNA-templated; GO:00063	GO:0000785 chromatin; GO:0005622 intracellular; GO:0005623 cell; GO:0005634 nucleus; GO:0005654 nucleoplasm; GO:0005694 chromosome; GO:0008023 transcription elongation factor complex; GO:0015030 Cajal body; GO:0016604 nuclear	GO:0005488 binding; GO:0005515 protein binding; GO:0019899 enzyme binding; GO:0019902 phosphatase binding;	PF07303	Occludin homology domain	K	KOG4796	RNA polymerase II elongation factor

P67775	Serine/threonine-protein phosphatase 2A catalytic subunit alpha isoform OS=Homo sapiens OX=9606 GN=PPP2CA PE=1 SV=1	PPP2CA	1.329	0.04588896	Up	K04382	PPP2C; serine/threonine-protein phosphatase 2A catalytic subunit [EC:3.1.3.16]	map03015 mRNA surveillance pathway; map04013 MAPK signaling pathway - fly; map04071 Sphingolipid signaling pathway; map04114 Oocyte meiosis; map04136 Autophagy - other; map04140 Autophagy - animal; map04151 PI3K-Akt signaling pathway; map04152 AMPK signaling pathway; map04261 Adrenergic signaling in cardiomyocytes; map04350 TGF-beta signaling pathway; map04390 Hippo signaling pathway; map04391 Hippo signaling pathway - fly; map04530 Tight junction; map04728 Dopaminergic synapse; map04730 Long-term depression; map05142 Chagas disease (American trypanosomiasis); map05160 Hepatitis C; map05165 Human papillomavirus infection	GO:0000122 negative regulation of transcription from RNA polymerase II promoter; GO:0000184 nuclear-transcribed mRNA catabolic process, nonsense-mediated decay; GO:0000188 inactivation of MAPK activity; GO:0000956 nuclear-transcribed mRNA catabolic process;	GO:0000159 protein phosphatase type 2A complex; GO:0000775 chromosome, centromeric region; GO:0005622 intracellular; GO:0005623 cell; GO:0005634 nucleus; GO:0005694 chromosome; GO:0005737 cytoplasm; GO:0005739 mitochondrion; GO:00058	GO:0001664 G-protein coupled receptor binding; GO:0003823 antigen binding; GO:0003824 catalytic activity; GO:0004721 phosphoprotein phosphatase activity; GO:0004722 protein serine/threonine phosphatase activity; GO:0005102 receptor binding; GO:0005488 binding; GO:0005515 protein	PF00149	Calcineurin-like phosphoesterase	T	KOG0371	Serine/threonine protein phosphatase 2A, catalytic subunit
Q7L211	Protein ABHD13 OS=Homo sapiens OX=9606 GN=ABHD13 PE=2 SV=1	ABHD13	1.328	0.020185744	Up	K06889	K06889; uncharacterized protein		GO:0002084 protein depalmitoylation; GO:0006464 cellular protein modification process; GO:0006807 nitrogen compound metabolic process; GO:0008152 metabolic process; GO:0009056 catabolic process; GO:0009057 macromolecule catabolic process; GO:0009987 cellular process;	GO:0005622 intracellular; GO:0005623 cell; GO:0005737 cytoplasm; GO:0030425 dendrite; GO:0032838 cell projection cytoplasm; GO:0032839 dendrite cytoplasm; GO:0036477 somatodendritic compartment; GO:0042995 cell projection; GO:0043005 neuron projection;	GO:0003824 catalytic activity; GO:0008474 palmitoyl-(protein) hydrolase activity; GO:0016787 hydrolase activity; GO:0016788 hydrolase activity, acting on ester bonds; GO:0016790 thiolester hydrolase activity; GO:0098599 palmitoyl hydrolase activity;			S	KOG4391	Predicted alpha/beta hydrolase BEM46
P63092	Guanine nucleotide-binding protein G(s) subunit alpha isoforms short OS=Homo sapiens OX=9606 GN=GNAS PE=1 SV=1	GNAS	1.327	0.031926833	Up	K04632	GNAS; guanine nucleotide-binding protein G(s) subunit alpha	map01522 Endocrine resistance; map04015 Rap1 signaling pathway; map04020 Calcium signaling pathway; map04024 cAMP signaling pathway; map04072 Phospholipase D signaling pathway; map04261 Adrenergic signaling in cardiomyocytes; map04270 Vascular smooth muscle contraction; map04361 Axon regeneration; map04540 Gap junction; map04611 Platelet activation; map04713 Circadian entrainment; map04714 Thermogenesis; map04724 Glutamatergic synapse; map04726 Serotonergic synapse; map04728 Dopaminergic synapse; map04730 Long-term depression; map04750 Inflammatory mediator regulation of TRP channels; map04911 Insulin secretion; map04912 GnRH signaling pathway; map04913 Ovarian steroidogenesis; map04915 Estrogen signaling pathway; map04916 Melanogenesis; map04918 Thyroid hormone synthesis; map04921 Oxytocin signaling pathway; map04922 Glucagon signaling pathway; map04923 Regulation of lipolysis in adipocytes; map04924 Renin secretion; map04925 Aldosterone synthesis and secretion; map04926 Relaxin signaling pathway; map04927 Cortisol synthesis and secretion; map04928 Parathyroid hormone synthesis, secretion and action; map04934 Cushing syndrome; map04935 Growth hormone synthesis, secretion and action; map04961 Endocrine and other factor-regulated calcium reabsorption; map04962 Vasopressin-regulated water reabsorption; map04970 Salivary secretion; map04971 Gastric acid secretion; map04972 Pancreatic secretion; map04976 Bile secretion; map05030 Cocaine addiction; map05031 Amphetamine addiction; map05032 Morphine addiction; map05034 Alcoholism; map05110 Vibrio cholerae infection; map05142 Chagas disease (American trypanosomiasis); map05146 Amoebiasis; map05163 Human cytomegalovirus infection; map05165 Human papillomavirus infection; map05200 Pathways in cancer; map05414 Dilated cardiomyopathy (DCM)	GO:0000003 reproduction; GO:0001101 response to acid chemical; GO:0001501 skeletal system development; GO:0001503 ossification; GO:0001775 cell activation; GO:0001894 tissue homeostasis; GO:0001932 regulation of protein phosphorylation;	GO:0000139 Golgi membrane; GO:0001726 ruffle; GO:0005622 intracellular; GO:0005623 cell; GO:0005634 nucleus; GO:0005737 cytoplasm; GO:0005768 endosome; GO:0005794 Golgi apparatus; GO:0005802 trans-Golgi network; GO:0005829 cytosol; GO:0005834 heterotrime	GO:0000166 nucleotide binding; GO:0001664 G-protein coupled receptor binding; GO:0001882 nucleoside binding; GO:0001883 purine nucleoside binding; GO:0001965 G-protein alpha-subunit binding; GO:0003824 catalytic activity; GO:0003924 GTPase activity; GO:00051	PF00503	G-protein alpha subunit	T	KOG0099	G protein subunit Galphas, small G protein superfamily
Q9Y250	Leucine zipper putative tumor suppressor 1 OS=Homo sapiens OX=9606 GN=LZTS1 PE=1 SV=3	LZTS1	1.326	0.022461075	Up				GO:0006139 nucleobase-containing compound metabolic process; GO:0006351 transcription, DNA-templated; GO:0006355 regulation of transcription, DNA-templated; GO:0006725 cellular aromatic compound metabolic process; GO:0006807 nitrogen compound metabolic process; GO:00072	GO:0005623 cell; GO:0005886 plasma membrane; GO:0014069 postsynaptic density; GO:0016020 membrane; GO:0016324 apical plasma membrane; GO:0030425 dendrite; GO:0032279 asymmetric synapse; GO:0036477 somatodendritic compartment; GO:0042995 cell	GO:0003676 nucleic acid binding; GO:0003677 DNA binding; GO:0003700 transcription factor activity, sequence-specific DNA binding; GO:0005488 binding; GO:0097159 organic cyclic compound binding; GO:1901363 heterocyclic compound binding;					
O95235	Kinesin-like protein KIF20A OS=Homo sapiens OX=9606 GN=KIF20A PE=1 SV=1	KIF20A	1.324	0.008245002	Up	K10402	KIF20; kinesin family member 20		GO:0000226 microtubule cytoskeleton organization; GO:0000278 mitotic cell cycle; GO:0000281 mitotic cytokinesis; GO:0000910 cytokinesis; GO:0000920 cell separation after cytokinesis; GO:0001578 microtubule bundle formation; GO:00068	GO:0005576 extracellular region; GO:0005622 intracellular; GO:0005623 cell; GO:0005634 nucleus; GO:0005654 nucleoplasm; GO:0005737 cytoplasm; GO:0005794 Golgi apparatus; GO:0005819 spindle; GO:0005856 cytoskeleton; GO:0005871 kinesin complex;	GO:0003774 motor activity; GO:0003777 microtubule motor activity; GO:0003824 catalytic activity; GO:0005215 transporter activity; GO:0005488 binding; GO:0005515 protein binding; GO:0016462 pyrophosphatase activity; GO:0016787 hydrolase activity; GO:0016817	PF00225	Kinesin motor domain	Z	KOG0247	Kinesin-like protein

P32929	Cystathionine gamma-lyase OS=Homo sapiens OX=9606 GN=CTH PE=1 SV=3	CTH	1.323	0.011498199	Up	K01758	CTH; cystathionine gamma-lyase [EC:4.4.1.1]	map00260 Glycine, serine and threonine metabolism; map00270 Cysteine and methionine metabolism; map00450 Selenocompound metabolism; map01100 Metabolic pathways; map01230 Biosynthesis of amino acids	GO:0000096 sulfur amino acid metabolic process; GO:0000097 sulfur amino acid biosynthetic process; GO:0000098 sulfur amino acid catabolic process; GO:0001558 regulation of cell growth; GO:0006082 organic acid metabolic process; GO:0006355 regulation of transcription, DNA-	GO:0005622 intracellular; GO:0005623 cell; GO:0005737 cytoplasm; GO:0005829 cytosol; GO:0044424 intracellular part; GO:0044444 cytoplasmic part; GO:0044464 cell part;	GO:0003824 catalytic activity; GO:0003962 cystathionine gamma-synthase activity; GO:0004121 cystathionine beta-lyase activity; GO:0004123 cystathionine gamma-lyase activity; GO:0005488 binding; GO:0005515 protein binding; GO:0008144 drug binding; GO:0016740	PF01053	Cys/Met metabolism PLP-dependent enzyme	E	KOG0053	Cystathionine beta-lyases/cystathionine gamma-synthases
Q8NBI5	Solute carrier family 43 member 3 OS=Homo sapiens OX=9606 GN=SLC43A3 PE=1 SV=2	SLC43A3	1.323	0.013872206	Up	K08230	SLC43A3; MFS transporter, LAT3 family, solute carrier family 43, member 3									
Q15800	Methylsterol monooxygenase 1 OS=Homo sapiens OX=9606 GN=MSMO1 PE=1 SV=1	MSMO1	1.322	0.015317069	Up	K07750	MESO1, ERG25; methylsterol monooxygenase [EC:1.14.18.9]	map00100 Steroid biosynthesis; map01100 Metabolic pathways	GO:0006066 alcohol metabolic process; GO:0006082 organic acid metabolic process; GO:0006629 lipid metabolic process; GO:0006631 fatty acid metabolic process; GO:0006694 steroid biosynthetic process; GO:0006695 cholesterol biosynthetic process; GO:0008152 metabolic process;	GO:0005622 intracellular; GO:0005623 cell; GO:0005737 cytoplasm; GO:0005783 endoplasmic reticulum; GO:0005789 endoplasmic reticulum membrane; GO:0005886 plasma membrane; GO:0012505 endomembrane system; GO:0016020 membrane; GO:00160	GO:0000254 C-4 methylsterol oxidase activity; GO:0003824 catalytic activity; GO:0004497 monooxygenase activity; GO:0016491 oxidoreductase activity; GO:0016705 oxidoreductase activity, acting on paired donors, with incorporation or reduction of			I	KOG0873	C-4 sterol methyl oxidase
Q96LJ7	Dehydrogenase/reductase SDR family member 1 OS=Homo sapiens OX=9606 GN=DHRS1 PE=1 SV=1	DHRS1	1.322	0.010832729	Up	K11163	DHRS1; dehydrogenase/reductase SDR family member 1 [EC:1.1.-.-]			GO:0005622 intracellular; GO:0005623 cell; GO:0005737 cytoplasm; GO:0005783 endoplasmic reticulum; GO:0012505 endomembrane system; GO:0043226 organelle; GO:0043227 membrane-bounded organelle; GO:0043229 intracellular organelle;		PF00106	short chain dehydrogenase	Q	KOG0725	Reductases with broad range of substrate specificities
O95994	Anterior gradient protein 2 homolog OS=Homo sapiens OX=9606 GN=AGR2 PE=1 SV=1	AGR2	1.322	0.004813788	Up	K20356	AGR2; anterior gradient protein 2		GO:0002065 columnar/cuboidal epithelial cell differentiation; GO:0002067 glandular epithelial cell differentiation; GO:0006810 transport; GO:0007275 multicellular organism development; GO:0007589 body fluid secretion; GO:0009653	GO:0005576 extracellular region; GO:0005615 extracellular space; GO:0005622 intracellular; GO:0005623 cell; GO:0005737 cytoplasm; GO:0005783 endoplasmic reticulum; GO:0012505 endomembrane system; GO:0043226 organelle; GO:00432	GO:0002162 dystroglycan binding; GO:0005102 receptor binding; GO:0005154 epidermal growth factor receptor binding; GO:0005488 binding; GO:0005515 protein binding; GO:0042802 identical protein binding; GO:0042803 protein homodimerization activity; GO:0046983 protein	PF13899	Thioredoxin-like			

O43167	Zinc finger and BTB domain-containing protein 24 OS=Homo sapiens OX=9606 GN=ZBTB24 PE=1 SV=2	ZBTB24	1.321	0.006022885	Up	K10503	ZBTB24, BIF1; zinc finger and BTB domain-containing protein 24		GO:0002244 hematopoietic progenitor cell differentiation; GO:0002376 immune system process; GO:0002520 immune system development; GO:0006355 regulation of transcription, DNA-templated; GO:0006357 regulation of transcription from RNA		GO:0000981 RNA polymerase II transcription factor activity, sequence-specific DNA binding; GO:0003700 transcription factor activity, sequence-specific DNA binding;	PF00651; PF00096	BTB/POZ domain; Zinc finger, C2H2 type	K	KOG1721	FOG: Zn-finger
Q99523	Sortilin OS=Homo sapiens OX=9606 GN=SORT1 PE=1 SV=3	SORT1	1.32	0.006697463	Up	K12388	SORT1; sortilin	map04142 Lysosome; map04722 Neurotrophin signaling pathway; map04979 Cholesterol metabolism	GO:0006810 transport; GO:0006886 intracellular protein transport; GO:0006892 post-Golgi vesicle-mediated transport; GO:0006895 Golgi to endosome transport; GO:0006897 endocytosis; GO:0006915 apoptotic process; GO:0006996 organelle organization;	GO:0005622 intracellular; GO:0005623 cell; GO:0005737 cytoplasm; GO:0005768 endosome; GO:0005769 early endosome; GO:0005794 Golgi apparatus; GO:0005798 Golgi-associated vesicle; GO:0005829 cytosol; GO:0005886 plasma membrane; GO:0005905 clathrin-coated pit; GO:00099	GO:0001653 peptide receptor activity; GO:0004888 transmembrane signaling receptor activity; GO:0004930 G-protein coupled receptor activity; GO:0005030 neurotrophin receptor activity; GO:0005488 binding; GO:0005515 protein binding; GO:0008188 neuropeptide receptor			U	KOG3511	Sortilin and related receptors
Q9UMY1	Nucleolar protein 7 OS=Homo sapiens OX=9606 GN=NOL7 PE=1 SV=2	NOL7	1.32	0.035510111	Up					GO:0005622 intracellular; GO:0005623 cell; GO:0005634 nucleus; GO:0005730 nucleolus; GO:0005737 cytoplasm; GO:0005739 mitochondrion; GO:0031974 membrane-enclosed lumen; GO:0031981 nuclear lumen; GO:0043226 organelle; GO:0043227		PF08157	NUC129 domain			
P10619	Lysosomal protective protein OS=Homo sapiens OX=9606 GN=CTSA PE=1 SV=2	CTSA	1.318	0.000842427	Up	K13289	CTSA, CPY; cathepsin A (carboxypeptidase C) [EC:3.4.16.5]	map04142 Lysosome; map04614 Renin-angiotensin system	GO:0001775 cell activation; GO:0002252 immune effector process; GO:0002263 cell activation involved in immune response; GO:0002274 myeloid leukocyte activation; GO:0002275 myeloid cell activation involved in immune response; GO:0002283 neutrophil activation involved in immune response;	GO:0000323 lytic vacuole; GO:0005576 extracellular region; GO:0005622 intracellular; GO:0005623 cell; GO:0005634 nucleus; GO:0005654 nucleoplasm; GO:0005737 cytoplasm; GO:0005764 lysosome; GO:0005766 primary lysosome; GO:0005773 vacuole; GO:00057	GO:0003824 catalytic activity; GO:0004180 carboxypeptidase activity; GO:0004185 serine-type carboxypeptidase activity; GO:0004308 exo-alpha-sialidase activity; GO:0004553 hydrolase activity, hydrolyzing O-glycosyl compounds; GO:0008047 enzyme activator	PF00450	Serine carboxypeptidase	O	KOG1282	Serine carboxypeptidases (lysosomal cathepsin A)
Q9P0N9	TBC1 domain family member 7 OS=Homo sapiens OX=9606 GN=TBC1D7 PE=1 SV=1	TBC1D7	1.317	0.029959645	Up	K20396	TBC1D7; TBC1 domain family member 7	map04150 mTOR signaling pathway	GO:0009893 positive regulation of metabolic process; GO:0009966 regulation of signal transduction; GO:0009968 negative regulation of signal transduction; GO:0010033 response to organic substance; GO:0010604 positive regulation of macromolecule metabolic process;	GO:0005622 intracellular; GO:0005623 cell; GO:0005737 cytoplasm; GO:0005815 microtubule organizing center; GO:0005829 cytosol; GO:0005856 cytoskeleton; GO:0005929 cilium; GO:0015630 microtubule cytoskeleton; GO:0031410	GO:0005096 GTPase activator activity; GO:0005488 binding; GO:0005515 protein binding; GO:0008047 enzyme activator activity; GO:0017016 Ras GTPase binding; GO:0017137 Rab GTPase binding; GO:0019899 enzyme binding; GO:0030234 enzyme regulator activity; GO:0030695 GTPase regulator					

P02671	Fibrinogen alpha chain OS=Homo sapiens OX=9606 GN=FGA PE=1 SV=2	FGA	1.315	0.002801658	Up	K03903	FGA; fibrinogen alpha chain	map04610 Complement and coagulation cascades; map04611 Platelet activation	GO:0001775 cell activation; GO:0001889 liver development; GO:0001932 regulation of protein phosphorylation; GO:0001934 positive regulation of protein phosphorylation; GO:0002218 activation of innate immune response; GO:0002221 pattern recognition receptor signaling pathway;	GO:0005576 extracellular region; GO:0005577 fibrinogen complex; GO:0005615 extracellular space; GO:0005622 intracellular; GO:0005623 cell; GO:0005737 cytoplasm; GO:0005783 endoplasmic reticulum; GO:0005788 endoplasmic reticulum lumen;	GO:0005198 structural molecule activity;	PF12160; PF00147	Fibrinogen alpha C domain; Fibrinogen beta and gamma chains, C-terminal globular domain	S	KOG2579	Ficolin and related extracellular proteins
Q9UK99	F-box only protein 3 OS=Homo sapiens OX=9606 GN=FBXO3 PE=1 SV=3	FBXO3	1.315	0.00876469	Up	K10290	FBXO3; F-box protein 3		GO:0006464 cellular protein modification process; GO:0006508 proteolysis; GO:0006807 nitrogen compound metabolic process; GO:0008152 metabolic process; GO:0009987 cellular process; GO:0016567 protein ubiquitination; GO:0019538 protein metabolic process; GO:0032446 protein modificatio	GO:0005622 intracellular; GO:0005623 cell; GO:0005634 nucleus; GO:0005654 nucleoplasm; GO:0005737 cytoplasm; GO:0005829 cytosol; GO:0031974 membrane-enclosed lumen; GO:0031981 nuclear lumen; GO:0043226 organelle; GO:0043227 membrane-	GO:0003824 catalytic activity; GO:0004842 ubiquitin-protein transferase activity; GO:0016740 transferase activity; GO:0019787 ubiquitin-like protein transferase activity;	PF12937; PF09346	F-box-like; SMI1 / KNR4 family (SUKH-1)	P	KOG4408	Putative Mg2+ and Co2+ transporter CorD
Q8IXH7	Negative elongation factor C/D OS=Homo sapiens OX=9606 GN=NELFCD PE=1 SV=2	NELFCD	1.315	0.000203857	Up	K15181	TH1L, NELFD; negative elongation factor C/D		GO:0006139 nucleobase-containing compound metabolic process; GO:0006351 transcription, DNA-templated; GO:0006354 DNA-templated transcription, elongation; GO:0006366 transcription from RNA polymerase II promoter; GO:0006368 transcription elongation	GO:0005622 intracellular; GO:0005623 cell; GO:0005634 nucleus; GO:0005654 nucleoplasm; GO:0008023 transcription elongation factor complex; GO:0031974 membrane-enclosed lumen; GO:0031981 nuclear lumen; GO:0032021 NELF complex; GO:00329						
P04040	Catalase OS=Homo sapiens OX=9606 GN=CAT PE=1 SV=3	CAT	1.313	0.003456077	Up	K03781	katE, CAT, catB, srpA; catalase [EC:1.11.1.6]	map00380 Tryptophan metabolism; map00630 Glyoxylate and dicarboxylate metabolism; map01100 Metabolic pathways; map01200 Carbon metabolism; map04068 FoxO signaling pathway; map04146 Peroxisome; map04211 Longevity regulating pathway; map04212 Longevity regulating pathway - worm; map04213 Longevity regulating pathway - multiple species; map05014 Amyotrophic lateral sclerosis (ALS)	GO:0000302 response to reactive oxygen species; GO:0001101 response to acid chemical; GO:0001655 urogenital system development; GO:0001657 ureteric bud development; GO:0001666 response to hypoxia; GO:0001775 cell activation; GO:0001822 kidney	GO:0000323 lytic vacuole; GO:0005576 extracellular region; GO:0005615 extracellular space; GO:0005622 intracellular; GO:0005623 cell; GO:0005737 cytoplasm; GO:0005739 mitochondrion; GO:0005740 mitochondrial envelope; GO:0005758	GO:0000166 nucleotide binding; GO:0003824 catalytic activity; GO:0004046 aminoacylase activity; GO:0004096 catalase activity; GO:0004601 peroxidase activity; GO:0005102 receptor binding; GO:0005488 binding; GO:0005515 protein binding; GO:0016209 antioxidant activity; GO:00164	PF00199	Catalase	P	KOG0047	Catalase
P31323	cAMP-dependent protein kinase type II-beta regulatory subunit OS=Homo sapiens OX=9606 GN=PRKAR2B PE=1 SV=3	PRKAR2B	1.313	0.002255708	Up	K04739	PRKAR; cAMP-dependent protein kinase regulator	map04910 Insulin signaling pathway	GO:0000086 G2/M transition of mitotic cell cycle; GO:0000278 mitotic cell cycle; GO:0001932 regulation of protein phosphorylation; GO:0001933 negative regulation of protein phosphorylation; GO:0001934 positive regulation of protein phosphorylation; GO:0003008 system process; GO:0003014 renal	GO:0005622 intracellular; GO:0005623 cell; GO:0005737 cytoplasm; GO:0005739 mitochondrion; GO:0005740 mitochondrial envelope; GO:0005743 mitochondrial inner membrane; GO:0005813 centrosome; GO:0005815 microtubule	GO:0000166 nucleotide binding; GO:0004857 enzyme inhibitor activity; GO:0004860 protein kinase inhibitor activity; GO:0004862 cAMP-dependent protein kinase inhibitor activity; GO:0005488 binding; GO:0005515 protein binding; GO:0008603 cAMP-dependent protein kinase regulator	PF02197; PF00027	Regulatory subunit of type II PKA R-subunit; Cyclic nucleotide-binding domain	T	KOG1113	cAMP-dependent protein kinase types I and II, regulatory subunit

Q9H5V9	UPF0428 protein CXorf56 OS=Homo sapiens OX=9606 GN=CXorf56 PE=1 SV=1	CXorf56	1.313	0.017997126	Up					GO:0005622 intracellular; GO:0005623 cell; GO:0005634 nucleus; GO:0043226 organelle; GO:0043227 membrane-bounded organelle; GO:0043229 intracellular organelle; GO:0043231 intracellular membrane-bounded organelle; GO:0044297 cell body; GO:00444				S	KOG4397	Uncharacterized conserved protein
P98088	Mucin-5AC OS=Homo sapiens OX=9606 GN=MUC5AC PE=1 SV=4	MUC5AC	1.312	0.003601291	Up	K21125	MUC5AC; mucin-5AC	map04657 IL-17 signaling pathway	GO:0002218 activation of innate immune response; GO:0002220 innate immune response activating cell surface receptor signaling pathway; GO:0002223 stimulatory C-type lectin receptor signaling pathway; GO:0002253 activation of immune response; GO:0002376 immune system	GO:0005576 extracellular region; GO:0005615 extracellular space; GO:0005622 intracellular; GO:0005623 cell; GO:0005737 cytoplasm; GO:0005794 Golgi apparatus; GO:0005796 Golgi lumen; GO:0005886 plasma membrane; GO:0012505 endomembrane system;	GO:0005198 structural molecule activity; GO:0005201 extracellular matrix structural constituent;	PF08742; PF01826	C8 domain; Trypsin Inhibitor like cysteine rich domain	W	KOG1216	von Willebrand factor and related coagulation proteins
Q9UHL4	Dipeptidyl peptidase 2 OS=Homo sapiens OX=9606 GN=DPP7 PE=1 SV=3	DPP7	1.311	0.005983799	Up	K01276	DPP7; dipeptidyl-peptidase II [EC:3.4.14.2]		GO:0001775 cell activation; GO:0002252 immune effector process; GO:0002263 cell activation involved in immune response; GO:0002274 myeloid leukocyte activation; GO:0002275 myeloid cell activation involved in immune response; GO:0002283 neutrophil activation involved in immune response;	GO:0000323 lytic vacuole; GO:0005576 extracellular region; GO:0005622 intracellular; GO:0005623 cell; GO:0005737 cytoplasm; GO:0005764 lysosome; GO:0005766 primary lysosome; GO:0005773 vacuole; GO:0005775 vacuolar lumen; GO:0005794 Golgi apparatus;	GO:0003824 catalytic activity; GO:0004177 aminopeptidase activity; GO:0008233 peptidase activity; GO:0008236 serine-type peptidase activity; GO:0008238 exopeptidase activity; GO:0008239 dipeptidyl-peptidase activity; GO:0016787 hydrolase activity; GO:00171	PF05577	Serine carboxypeptidase S28	O	KOG2183	Prolylcarboxypeptidase (angiotensinase C)
Q9HAD4	WD repeat-containing protein 41 OS=Homo sapiens OX=9606 GN=WDR41 PE=1 SV=3	WDR41	1.311	0.030693737	Up	K23610	WDR41; WD repeat-containing protein 41	map04140 Autophagy - animal	GO:0009894 regulation of catabolic process; GO:0010506 regulation of autophagy; GO:0019222 regulation of metabolic process; GO:0031323 regulation of cellular metabolic process; GO:0031329 regulation of cellular catabolic process; GO:0050789	GO:0005622 intracellular; GO:0005623 cell; GO:0005737 cytoplasm; GO:0032045 guanyl-nucleotide exchange factor complex; GO:0032991 macromolecular complex; GO:0044424 intracellular part; GO:0044464 cell part;						
P58557	Endoribonuclease YbeY OS=Homo sapiens OX=9606 GN=YBEY PE=1 SV=2	YBEY	1.311	0.024104221	Up					GO:0005622 intracellular; GO:0005623 cell; GO:0005634 nucleus; GO:0043226 organelle; GO:0043227 membrane-bounded organelle; GO:0043229 intracellular organelle; GO:0043231 intracellular membrane-bounded organelle; GO:0044424 intracellular part;						

P78417	Glutathione S-transferase omega-1 OS=Homo sapiens OX=9606 GN=GSTO1 PE=1 SV=2	GSTO1	1.31	0.012967071	Up	K00799	GST, gst; glutathione S-transferase [EC:2.5.1.18]	map00480 Glutathione metabolism; map00980 Metabolism of xenobiotics by cytochrome P450; map00982 Drug metabolism - cytochrome P450; map00983 Drug metabolism - other enzymes; map01100 Metabolic pathways; map01524 Platinum drug resistance; map04212 Longevity regulating pathway - worm; map05200 Pathways in cancer; map05204 Chemical carcinogenesis; map05225 Hepatocellular carcinoma; map05418 Fluid shear stress and atherosclerosis	GO:0005975 carbohydrate metabolic process; GO:0005996 monosaccharide metabolic process; GO:0006082 organic acid metabolic process; GO:0006518 peptide metabolic process; GO:0006575 cellular modified amino acid metabolic process; GO:0006732 coenzyme metabolic	GO:0005576 extracellular region; GO:0005604 basement membrane; GO:0005622 intracellular; GO:0005623 cell; GO:0005634 nucleus; GO:0005635 nuclear envelope; GO:0005737 cytoplasm; GO:0005829 cytosol; GO:0012505 endomembrane system; GO:0016020	GO:0003824 catalytic activity; GO:0004364 glutathione transferase activity; GO:0015036 disulfide oxidoreductase activity; GO:0015037 peptide disulfide oxidoreductase activity; GO:0015038 glutathione disulfide oxidoreductase activity; GO:0016209 antioxidant activity;	PF13417	Glutathione S-transferase, N-terminal domain	O	KOG0406	Glutathione S-transferase
Q14011	Cold-inducible RNA-binding protein OS=Homo sapiens OX=9606 GN=CIRBP PE=1 SV=1	CIRBP	1.31	0.036476245	Up	K13195	CIRBP; cold-inducible RNA-binding protein					PF00076	RNA recognition motif. (a.k.a. RRM, RBD, or RNP domain)	A	KOG0118	FOG: RRM domain
P15586	N-acetylglucosamine-6-sulfatase OS=Homo sapiens OX=9606 GN=GNS PE=1 SV=3	GNS	1.309	0.033224284	Up	K01137	GNS; N-acetylglucosamine-6-sulfatase [EC:3.1.6.14]	map00531 Glycosaminoglycan degradation; map01100 Metabolic pathways; map04142 Lysosome	GO:0001775 cell activation; GO:0002252 immune effector process; GO:0002263 cell activation involved in immune response; GO:0002274 myeloid leukocyte activation; GO:0002275 myeloid cell activation involved in immune response; GO:0002283 neutrophil activation involved in immune response;	GO:0000323 lytic vacuole; GO:0005576 extracellular region; GO:0005622 intracellular; GO:0005623 cell; GO:0005737 cytoplasm; GO:0005764 lysosome; GO:0005766 primary lysosome; GO:0005773 vacuole; GO:0005775 vacuolar lumen; GO:0012505 endomemb	GO:0003824 catalytic activity; GO:0005488 binding; GO:0005539 glycosaminoglycan binding; GO:0008449 N-acetylglucosamine-6-sulfatase activity; GO:0008484 sulfuric ester hydrolase activity; GO:0016787 hydrolase activity; GO:0016788 hydrolase activity, acting on ester			G	KOG3731	Sulfatases
O75127	Pentatricopeptide repeat-containing protein 1, mitochondrial OS=Homo sapiens OX=9606 GN=PTCD1 PE=1 SV=2	PTCD1	1.309	0.005806636	Up	K17710	PTCD1; pentatricopeptide repeat domain-containing protein 1		GO:0006139 nucleobase-containing compound metabolic process; GO:0006396 RNA processing; GO:0006399 tRNA metabolic process; GO:0006725 cellular aromatic compound metabolic process; GO:0006807 nitrogen compound metabolic process; GO:0008033 tRNA processing; GO:0008152 metabolic	GO:0005622 intracellular; GO:0005623 cell; GO:0005737 cytoplasm; GO:0005739 mitochondrion; GO:0005759 mitochondrial matrix; GO:0031974 membrane-enclosed lumen; GO:0043226 organelle; GO:0043227 membrane-bounded organelle; GO:00432	GO:0000049 tRNA binding; GO:0003676 nucleic acid binding; GO:0003723 RNA binding; GO:0005488 binding; GO:0097159 organic cyclic compound binding; GO:1901363 heterocyclic compound binding;			S	KOG4197	FOG: PPR repeat
P18858	DNA ligase 1 OS=Homo sapiens OX=9606 GN=LIG1 PE=1 SV=1	LIG1	1.308	0.001548214	Up	K10747	LIG1; DNA ligase 1 [EC:6.5.1.1 6.5.1.6 6.5.1.7]	map03030 DNA replication; map03410 Base excision repair; map03420 Nucleotide excision repair; map03430 Mismatch repair	GO:0000278 mitotic cell cycle; GO:0000302 response to reactive oxygen species; GO:0000726 non-recombinational repair; GO:0006139 nucleobase-containing compound metabolic process; GO:0006259 DNA metabolic process; GO:0006260 DNA replication; GO:0006261 DNA-dependent	GO:0005622 intracellular; GO:0005623 cell; GO:0005634 nucleus; GO:0005654 nucleoplasm; GO:0005737 cytoplasm; GO:0005739 mitochondrion; GO:0031974 membrane-enclosed lumen; GO:0031981 nuclear lumen; GO:0043226 organelle; GO:00432	GO:0003676 nucleic acid binding; GO:0003677 DNA binding; GO:0003824 catalytic activity; GO:0003909 DNA ligase activity; GO:0003910 DNA ligase (ATP) activity; GO:0005488 binding; GO:0016874 ligase activity; GO:0016886 ligase activity, forming phosphoric ester bonds;	PF01068	ATP dependent DNA ligase domain	L	KOG0967	ATP-dependent DNA ligase I

Q14674	Separin OS=Homo sapiens OX=9606 GN=ESPL1 PE=1 SV=3	ESPL1	1.306	0.002809028	Up	K02365	ESP1; separase [EC:3.4.22.49]	map04110 Cell cycle; map04114 Oocyte meiosis; map05166 Human T-cell leukemia virus 1 infection	GO:0000003 reproduction; GO:0000070 mitotic sister chromatid segregation; GO:0000212 meiotic spindle organization; GO:0000226 microtubule cytoskeleton organization; GO:0000278 mitotic cell cycle; GO:0000280 nuclear division; GO:0000281 mitotic	GO:0005622 intracellular; GO:0005623 cell; GO:0005634 nucleus; GO:0005737 cytoplasm; GO:0005813 centrosome; GO:0005815 microtubule organizing center; GO:0005819 spindle; GO:0005829 cytosol; GO:0005856 cytoskeleton; GO:0015630	GO:0003824 catalytic activity; GO:0004175 endopeptidase activity; GO:0004197 cysteine-type endopeptidase activity; GO:0008233 peptidase activity; GO:0008234 cysteine-type peptidase activity; GO:0016787 hydrolase activity; GO:0070011			D	KOG1849	Regulator of spindle pole body duplication
O75531	Barrier-to-autointegration factor OS=Homo sapiens OX=9606 GN=BANF1 PE=1 SV=1	BANF1	1.306	0.019776309	Up	K21870	BANF1; barrier-to-autointegration factor		GO:0000278 mitotic cell cycle; GO:0006810 transport; GO:0006996 organelle organization; GO:0006997 nucleus organization; GO:0006998 nuclear envelope organization; GO:0007049 cell cycle; GO:0007084 mitotic nuclear envelope reassembly; GO:0009605	GO:0005622 intracellular; GO:0005623 cell; GO:0005634 nucleus; GO:0005654 nucleoplasm; GO:0005737 cytoplasm; GO:0005829 cytosol; GO:0031974 membrane-enclosed lumen; GO:0031981 nuclear lumen; GO:0043226 organelle; GO:0043227 membrane-	GO:0005488 binding; GO:0005515 protein binding; GO:0008022 protein C-terminus binding; GO:0019899 enzyme binding; GO:0019904 protein domain specific binding; GO:0042802 identical protein binding; GO:0042803 protein homodimerization activity; GO:0046983 protein dimerization activity;	PF02961	Barrier to autointegration factor	BL	KOG4233	DNA-bridging protein BAF
Q15645	Pachytene checkpoint protein 2 homolog OS=Homo sapiens OX=9606 GN=TRIP13 PE=1 SV=2	TRIP13	1.306	0.04227029	Up	K22399	TRIP13; pachytene checkpoint protein 2		GO:0000003 reproduction; GO:0000075 cell cycle checkpoint; GO:0000278 mitotic cell cycle; GO:0000280 nuclear division; GO:0001556 oocyte maturation; GO:0003006 developmental process involved in reproduction; GO:0006139 nucleobase-containing compound metabolic	GO:0001673 male germ cell nucleus; GO:0005622 intracellular; GO:0005623 cell; GO:0005634 nucleus; GO:0043073 germ cell nucleus; GO:0043226 organelle; GO:0043227 membrane-bounded organelle; GO:0043229 intracellular organelle; GO:0043231 intracellula	GO:0000166 nucleotide binding; GO:0003712 transcription cofactor activity; GO:0005488 binding; GO:0005515 protein binding; GO:0005524 ATP binding; GO:0008144 drug binding; GO:0017076 purine nucleotide binding; GO:0030554 adenyl nucleotide binding; GO:0032553 ribonucleot	PF00004	ATPase family associated with various cellular activities (AAA)	L	KOG0744	AAA+-type ATPase
Q53R41	FAST kinase domain-containing protein 1, mitochondrial OS=Homo sapiens OX=9606 GN=FASTKD1 PE=1 SV=1	FASTKD1	1.305	0.02166398	Up				GO:0000959 mitochondrial RNA metabolic process; GO:0006091 generation of precursor metabolites and energy; GO:0006139 nucleobase-containing compound metabolic process; GO:0006725 cellular aromatic compound metabolic process; GO:0006807 nitrogen compound metabolic process;	GO:0005622 intracellular; GO:0005623 cell; GO:0005737 cytoplasm; GO:0005739 mitochondrion; GO:0043226 organelle; GO:0043227 membrane-bounded organelle; GO:0043229 intracellular organelle; GO:0043231 intracellular membrane-bounded		PF08373	RAP domain			
O75928	E3 SUMO-protein ligase PIAS2 OS=Homo sapiens OX=9606 GN=PIAS2 PE=1 SV=3	PIAS2	1.304	0.003113897	Up	K16063	PIAS2; E3 SUMO-protein ligase PIAS2 [EC:2.3.2.-]	map04120 Ubiquitin mediated proteolysis; map04630 JAK-STAT signaling pathway	GO:0006139 nucleobase-containing compound metabolic process; GO:0006351 transcription, DNA-templated; GO:0006355 regulation of transcription, DNA-templated; GO:0006357 regulation of transcription from RNA polymerase II promoter; GO:0006464 cellular	GO:0005622 intracellular; GO:0005623 cell; GO:0005634 nucleus; GO:0005654 nucleoplasm; GO:0016604 nuclear body; GO:0031974 membrane-enclosed lumen; GO:0031981 nuclear lumen; GO:0043226 organelle; GO:0043227 membrane-bounded organelle;	GO:0003676 nucleic acid binding; GO:0003677 DNA binding; GO:0003712 transcription cofactor activity; GO:0003713 transcription coactivator activity; GO:0003824 catalytic activity; GO:0005102 receptor binding; GO:0005488 binding; GO:0005515 protein binding; GO:0008134	PF14324; PF02891	PINIT domain; MIZ/SP-RING zinc finger	K	KOG2169	Zn-finger transcription factor

Q96ED9	Protein Hook homolog 2 OS=Homo sapiens OX=9606 GN=HOOK2 PE=1 SV=3	HOOK2	1.303	0.005177575	Up	K16611	HOOK2; protein HOOK2		GO:0000226 microtubule cytoskeleton organization; GO:0006810 transport; GO:0006897 endocytosis; GO:0006996 organelle organization; GO:0007010 cytoskeleton organization; GO:0007017 microtubule-based process;	GO:0005622 intracellular; GO:0005623 cell; GO:0005737 cytoplasm; GO:0005813 centrosome; GO:0005815 microtubule organizing center; GO:0005829 cytosol; GO:0005856 cytoskeleton; GO:0015630 microtubule cytoskeleton;	GO:0005488 binding; GO:0005515 protein binding; GO:0008017 microtubule binding; GO:0008092 cytoskeletal protein binding; GO:0015631 tubulin binding; GO:0042802 identical protein binding; GO:0051959 dynein light intermediate chain binding;					
P81877	Single-stranded DNA-binding protein 2 OS=Homo sapiens OX=9606 GN=SSBP2 PE=1 SV=2	SSBP2	1.303	0.039570003	Up				GO:0006139 nucleobase-containing compound metabolic process; GO:0006351 transcription, DNA-templated; GO:0006355 regulation of transcription, DNA-templated; GO:0006357 regulation of transcription from RNA polymerase II promoter; GO:0006366	GO:0005622 intracellular; GO:0005623 cell; GO:0005634 nucleus; GO:0005737 cytoplasm; GO:0043226 organelle; GO:0043227 membrane-bounded organelle; GO:0043229 intracellular organelle; GO:0043231 intracellular membrane-bounded organelle; GO:00444	GO:0000976 transcription regulatory region sequence-specific DNA binding; GO:0000977 RNA polymerase II regulatory region sequence-specific DNA binding; GO:0000978 RNA polymerase II core promoter proximal region sequence-specific DNA binding;			L	KOG4594	Sequence-specific single-stranded-DNA-binding protein
O60488	Long-chain-fatty-acid--CoA ligase 4 OS=Homo sapiens OX=9606 GN=ACSL4 PE=1 SV=2	ACSL4	1.302	0.024982643	Up	K01897	ACSL, fadD; long-chain acyl-CoA synthetase [EC:6.2.1.3]	map00061 Fatty acid biosynthesis; map00071 Fatty acid degradation; map01100 Metabolic pathways; map01212 Fatty acid metabolism; map03320 PPAR signaling pathway; map04146 Peroxisome; map04216 Ferroptosis; map04714 Thermogenesis; map04920 Adipocytokine signaling pathway	GO:0000003 reproduction; GO:0001558 regulation of cell growth; GO:0001676 long-chain fatty acid metabolic process; GO:0006082 organic acid metabolic process; GO:0006139 nucleobase-containing compound metabolic process; GO:0006163 purine nucleotide metabolic	GO:0005622 intracellular; GO:0005623 cell; GO:0005737 cytoplasm; GO:0005739 mitochondrion; GO:0005740 mitochondrial envelope; GO:0005777 peroxisome; GO:0005783 endoplasmic reticulum; GO:0005789 endoplasmic reticulum	GO:0003824 catalytic activity; GO:0004467 long-chain fatty acid-CoA ligase activity; GO:0015645 fatty acid ligase activity; GO:0016405 CoA-ligase activity; GO:0016874 ligase activity; GO:0016877 ligase activity, forming carbon-sulfur bonds; GO:0016878 acid-thiol ligase activity;			I	KOG1180	Acyl-CoA synthetase
O43657	Tetraspanin-6 OS=Homo sapiens OX=9606 GN=TSPAN6 PE=1 SV=1	TSPAN6	1.302	0.003271509	Up	K17295	TSPAN6; tetraspanin-6		GO:0001817 regulation of cytokine production; GO:0002682 regulation of immune system process; GO:0002683 negative regulation of immune system process; GO:0002697 regulation of immune effector process; GO:0002698 negative regulation of immune effector process; GO:0002831	GO:0005623 cell; GO:0005886 plasma membrane; GO:0005887 integral component of plasma membrane; GO:0016020 membrane; GO:0016021 integral component of membrane; GO:0031224 intrinsic component of membrane; GO:0031226 intrinsic component of plasma membrane; GO:0044425 membrane				S	KOG3882	Tetraspanin family integral membrane protein
Q7Z4W1	L-xylulose reductase OS=Homo sapiens OX=9606 GN=DCXR PE=1 SV=2	DCXR	1.301	0.038395432	Up	K03331	DCXR; L-xylulose reductase [EC:1.1.1.10]	map00040 Pentose and glucuronate interconversions; map01100 Metabolic pathways	GO:0005975 carbohydrate metabolic process; GO:0005996 monosaccharide metabolic process; GO:0005997 xylulose metabolic process; GO:0006006 glucose metabolic process; GO:0006063 uronic acid metabolic process; GO:0006064 glucuronate catabolic process; GO:00060	GO:0005622 intracellular; GO:0005623 cell; GO:0005737 cytoplasm; GO:0005856 cytoskeleton; GO:0005874 microtubule; GO:0005881 cytoplasmic microtubule; GO:0005886 plasma membrane; GO:0005902 microvillus; GO:00059	GO:0003824 catalytic activity; GO:0005488 binding; GO:0005515 protein binding; GO:0016491 oxidoreductase activity; GO:0016614 oxidoreductase activity, acting on CH-OH group of donors; GO:0016616 oxidoreductase activity, acting on the CH-OH group of donors,	PF13561	Enoyl-(Acyl carrier protein) reductase	Q	KOG1207	Diacetyl reductase/L-xylulose reductase

Q8N568	Serine/threonine-protein kinase DCLK2 OS=Homo sapiens OX=9606 GN=DCLK2 PE=1 SV=4	DCLK2	0.769	0.028269763	Down	K08805	DCLK1_2; doublecortin-like kinase 1/2 [EC:2.7.11.1]		GO:0000226 microtubule cytoskeleton organization; GO:0006464 cellular protein modification process; GO:0006468 protein phosphorylation; GO:0006793 phosphorus metabolic process; GO:0006796 phosphate-containing compound metabolic process; GO:0006807 nitrogen	GO:0005622 intracellular; GO:0005623 cell; GO:0005856 cytoskeleton; GO:0015630 microtubule cytoskeleton; GO:0043226 organelle; GO:0043228 non-membrane-bounded organelle; GO:0043229 intracellular organelle; GO:0043232 intracellula	GO:0003824 catalytic activity; GO:0004672 protein kinase activity; GO:0004674 protein serine/threonine kinase activity; GO:0016301 kinase activity; GO:0016740 transferase activity; GO:0016772 transferase activity, transferring phosphorus-containing groups; GO:0016773 phosphotra	PF00069	Protein kinase domain	T	KOG0032	Ca2+/calmodulin-dependent protein kinase, EF-Hand protein superfamily
Q01844	RNA-binding protein EWS OS=Homo sapiens OX=9606 GN=EWSR1 PE=1 SV=1	EWSR1	0.769	0.016464821	Down	K13209	EWSR1; RNA-binding protein EWS	map05202 Transcriptional misregulation in cancer		GO:0005622 intracellular; GO:0005623 cell; GO:0005634 nucleus; GO:0005730 nucleolus; GO:0031974 membrane-enclosed lumen; GO:0031981 nuclear lumen; GO:0043226 organelle; GO:0043227 membrane-bounded organelle; GO:0043228 non-membrane-bounded	GO:0005488 binding; GO:0005515 protein binding; GO:0042802 identical protein binding;	PF00076; PF00641	RNA recognition motif. (a.k.a. RRM, RBD, or RNP domain); Zn-finger in Ran binding protein and others	A	KOG1995	Conserved Zn-finger protein
Q8WV22	Non-structural maintenance of chromosomes element 1 homolog OS=Homo sapiens OX=9606 GN=NSMCE1 PE=1 SV=5	NSMCE1	0.768	0.006666618	Down	K22817	NSMCE1, NSE1; non-structural maintenance of chromosomes element 1 [EC:2.3.2.27]		GO:0000724 double-strand break repair via homologous recombination; GO:0000725 recombinational repair; GO:0006139 nucleobase-containing compound metabolic process; GO:0006259 DNA metabolic process; GO:0006281 DNA repair; GO:0006301 postreplicat	GO:0000793 condensed chromosome; GO:0005622 intracellular; GO:0005623 cell; GO:0005634 nucleus; GO:0005654 nucleoplasm; GO:0005694 chromosome; GO:0030915 Smc5-Smc6 complex; GO:0031974 membrane-enclosed lumen; GO:00319	GO:0003824 catalytic activity; GO:0004842 ubiquitin-protein transferase activity; GO:0005488 binding; GO:0005515 protein binding; GO:0016740 transferase activity; GO:0019787 ubiquitin-like protein transferase activity; GO:0046983 protein dimerization activity;	PF08746	RING-like domain	B	KOG4718	Non-SMC (structural maintenance of chromosomes) element 1 protein (NSE1)
Q7Z417	Nuclear fragile X mental retardation-interacting protein 2 OS=Homo sapiens OX=9606 GN=NUFIP2 PE=1 SV=1	NUFIP2	0.768	0.006217644	Down					GO:0005622 intracellular; GO:0005623 cell; GO:0005634 nucleus; GO:0005654 nucleoplasm; GO:0005737 cytoplasm; GO:0005829 cytosol; GO:0005840 ribosome; GO:0005844 polysome; GO:0010494 cytoplasmic stress granule; GO:0016604 nuclear body;	GO:0003676 nucleic acid binding; GO:0003723 RNA binding; GO:0005488 binding; GO:0097159 organic cyclic compound binding; GO:1901363 heterocyclic compound binding;					
B7ZAP0	Rab GTPase-activating protein 1-like, isoform 10 OS=Homo sapiens OX=9606 GN=RABGAP1L PE=1 SV=1	RABGAP1L	0.767	0.016990278	Down	K20284	RABGAP1, GAPCENA; Rab GTPase-activating protein 1							S	KOG1102	Rab6 GTPase activator GAPCenA and related TBC domain proteins

Q96JK9	Mastermind-like protein 3 OS=Homo sapiens OX=9606 GN=MAML3 PE=1 SV=4	MAML3	0.766	0.025145757	Down	K06061	MAML; mastermind	map04330 Notch signaling pathway; map04658 Th1 and Th2 cell differentiation; map05165 Human papillomavirus infection	GO:0006139 nucleobase-containing compound metabolic process; GO:0006351 transcription, DNA-templated; GO:0006352 DNA-templated transcription, initiation; GO:0006355 regulation of transcription, DNA-templated; GO:0006357 regulation of transcription from	GO:0005622 intracellular; GO:0005623 cell; GO:0005634 nucleus; GO:0005654 nucleoplasm; GO:0016604 nuclear body; GO:0016607 nuclear speck; GO:0031974 membrane-enclosed lumen; GO:0031981 nuclear lumen; GO:0043226 organelle; GO:0043227	GO:0003712 transcription cofactor activity; GO:0003713 transcription coactivator activity;	PF09596	MamL-1 domain			
P42892	Endothelin-converting enzyme 1 OS=Homo sapiens OX=9606 GN=ECE1 PE=1 SV=2	ECE1	0.765	0.004502502	Down	K01415	ECE; endothelin-converting enzyme [EC:3.4.24.71]		GO:0001666 response to hypoxia; GO:0001919 regulation of receptor recycling; GO:0001921 positive regulation of receptor recycling; GO:0001932 regulation of protein phosphorylation; GO:0001990 regulation of systemic arterial blood pressure by hormone; GO:0003008 system process;	GO:0005622 intracellular; GO:0005623 cell; GO:0005737 cytoplasm; GO:0005768 endosome; GO:0005769 early endosome; GO:0005794 Golgi apparatus; GO:0005886 plasma membrane; GO:0009897 external side of plasma membrane; GO:0009986 cell surface; GO:0010008	GO:0003824 catalytic activity; GO:0004175 endopeptidase activity; GO:0004222 metalloendopeptidase activity; GO:0005488 binding; GO:0005515 protein binding; GO:0008233 peptidase activity; GO:0008237 metallopeptidase activity; GO:0008270 zinc ion binding; GO:00167			E	KOG3624	M13 family peptidase
P19022	Cadherin-2 OS=Homo sapiens OX=9606 GN=CDH2 PE=1 SV=4	CDH2	0.765	0.046042116	Down	K06736	CDH2, CDHN, CD325; cadherin 2, type 1, N-cadherin	map04514 Cell adhesion molecules (CAMs); map05412 Arrhythmogenic right ventricular cardiomyopathy (ARVC)	GO:0001568 blood vessel development; GO:0001932 regulation of protein phosphorylation; GO:0001934 positive regulation of protein phosphorylation; GO:0001941 postsynaptic membrane organization; GO:0001944 vasculature development; GO:0002065	GO:0005622 intracellular; GO:0005623 cell; GO:0005737 cytoplasm; GO:0005783 endoplasmic reticulum; GO:0005788 endoplasmic reticulum lumen; GO:0005886 plasma membrane; GO:0005887 integral component of plasma membrane; GO:0005911 cell-cell junction; GO:00059	GO:0005488 binding; GO:0005515 protein binding; GO:0008013 beta-catenin binding; GO:0019899 enzyme binding; GO:0019900 kinase binding; GO:0019901 protein kinase binding; GO:0019902 phosphatase binding; GO:0019903 protein phosphatase binding; GO:0042802 identical protein	PF08758; PF00028	Cadherin prodomain like; Cadherin domain	T	KOG3594	FOG: Cadherin repeats
Q8N137	Centrobin OS=Homo sapiens OX=9606 GN=CNTROB PE=1 SV=1	CNTROB	0.762	0.02131696	Down	K16468	CNTROB; centrobin		GO:0000226 microtubule cytoskeleton organization; GO:0000278 mitotic cell cycle; GO:0000281 mitotic cytokinesis; GO:0000910 cytokinesis; GO:0006996 organelle organization; GO:0007010 cytoskeleton organization; GO:00070	GO:0005622 intracellular; GO:0005623 cell; GO:0005737 cytoplasm; GO:0005813 centrosome; GO:0005814 centriole; GO:0005815 microtubule organizing center; GO:0005829 cytosol; GO:0005856 cytoskeleton; GO:0015630 microtubul	GO:0005488 binding; GO:0005515 protein binding; GO:0019904 protein domain specific binding;					
Q9H069	Dynein regulatory complex subunit 3 OS=Homo sapiens OX=9606 GN=DRC3 PE=1 SV=2	DRC3	0.762	0.035220019	Down					GO:0005622 intracellular; GO:0005623 cell; GO:0005737 cytoplasm; GO:0005856 cytoskeleton; GO:0005929 cilium; GO:0005930 axoneme; GO:0032838 cell projection cytoplasm; GO:0042995 cell projection; GO:0043226 organelle; GO:0043228 non-membrane-				T	KOG0531	Protein phosphatase 1, regulatory subunit, and related proteins

Q8NEC7	Glutathione S-transferase C-terminal domain-containing protein OS=Homo sapiens OX=9606 GN=GSTCD PE=1 SV=2	GSTCD	0.762	0.021735076	Down					GO:0005622 intracellular; GO:0005623 cell; GO:0005634 nucleus; GO:0005654 nucleoplasm; GO:0005737 cytoplasm; GO:0031974 membrane-enclosed lumen; GO:0031981 nuclear lumen; GO:0043226 organelle; GO:0043227 membrane-bounded organelle;		PF13679	Methyltransferase domain			
Q07157	Tight junction protein ZO-1 OS=Homo sapiens OX=9606 GN=TJP1 PE=1 SV=3	TJP1	0.761	0.018971947	Down	K05701	TJP1, ZO1; tight junction protein 1	map04520 Adherens junction; map04530 Tight junction; map04540 Gap junction; map05110 Vibrio cholerae infection; map05120 Epithelial cell signaling in Helicobacter pylori infection; map05130 Pathogenic Escherichia coli infection; map05132 Salmonella infection	GO:0001701 in utero embryonic development; GO:0001824 blastocyst development; GO:0001825 blastocyst formation; GO:0001885 endothelial cell development; GO:0002064 epithelial cell development; GO:0003008 system process; GO:0003158	GO:0005622 intracellular; GO:0005623 cell; GO:0005634 nucleus; GO:0005737 cytoplasm; GO:0005829 cytosol; GO:0005886 plasma membrane; GO:0005911 cell-cell junction; GO:0005912 adherens junction; GO:0005913 cell-cell adherens junction; GO:0005923 bicellular tight	GO:0005488 binding; GO:0005515 protein binding; GO:0008022 protein C-terminus binding; GO:0019904 protein domain specific binding; GO:0071253 connexin binding;	PF00595; PF07653; PF00625	PDZ domain (Also known as DHR or GLGF); Variant SH3 domain; Guanylate kinase	T	KOG3580	Tight junction proteins
O00488	Zinc finger protein 593 OS=Homo sapiens OX=9606 GN=ZNF593 PE=1 SV=2	ZNF593	0.76	0.04660635	Down	K14821	BUD20; bud site selection protein 20		GO:0000054 ribosomal subunit export from nucleus; GO:0000055 ribosomal large subunit export from nucleus; GO:0006355 regulation of transcription, DNA-templated; GO:0006357 regulation of transcription from RNA polymerase II	GO:0005622 intracellular; GO:0005623 cell; GO:0005634 nucleus; GO:0005730 nucleolus; GO:0030684 preribosome; GO:0030687 preribosome, large subunit precursor; GO:0031974 membrane-enclosed lumen; GO:0031981 nuclear lumen; GO:0032991	GO:0000981 RNA polymerase II transcription factor activity, sequence-specific DNA binding; GO:0003700 transcription factor activity, sequence-specific DNA binding; GO:0005488 binding; GO:0008270 zinc ion binding; GO:0043021 ribonucleoprotein complex binding;			K	KOG3408	U1-like Zn-finger-containing protein, probabl erole in RNA processing/splicing
C9JI98	Transmembrane protein 238 OS=Homo sapiens OX=9606 GN=TMEM238 PE=1 SV=1	TMEM238	0.759	2.4681E-05	Down											
Q96I23	Protein preY, mitochondrial OS=Homo sapiens OX=9606 GN=PYURF PE=1 SV=1	PYURF	0.757	0.033013992	Down	K11001	PIGY; phosphatidylinositol N-acetylglucosaminyltransferase subunit Y	map00563 Glycosylphosphatidylinositol (GPI)-anchor biosynthesis; map01100 Metabolic pathways	GO:0006464 cellular protein modification process; GO:0006497 protein lipidation; GO:0006505 GPI anchor metabolic process; GO:0006506 GPI anchor biosynthetic process; GO:0006629 lipid metabolic process; GO:0006643 membrane lipid metabolic process; GO:0006644 phospholip	GO:0000506 glycosylphosphatidylinositol-N-acetylglucosaminyltransferase (GPI-GnT) complex; GO:0005622 intracellular; GO:0005623 cell; GO:0005737 cytoplasm; GO:0005783 endoplasmic reticulum; GO:0005789 endoplasmic reticulum membrane; GO:0005886 plasma		PF03966	Trm112p-like protein			
Q8N8A2	Serine/threonine-protein phosphatase 6 regulatory ankyrin repeat subunit B OS=Homo sapiens OX=9606 GN=ANKRD44 PE=1 SV=3	ANKRD44	0.757	0.034240416	Down	K15503	ANKRD44; serine/threonine-protein phosphatase 6 regulatory ankyrin repeat subunit B							M	KOG4177	Ankyrin

Q9BTT6	Leucine-rich repeat-containing protein 1 OS=Homo sapiens OX=9606 GN=LRRC1 PE=1 SV=1	LRRC1	0.757	0.008158115	Down				GO:0007154 cell communication; GO:0007165 signal transduction; GO:0009987 cellular process; GO:0023052 signaling; GO:0050789 regulation of biological process; GO:0050794 regulation of cellular process; GO:0050896 response to stimulus; GO:0051716 cellular	GO:0005622 intracellular; GO:0005623 cell; GO:0005737 cytoplasm; GO:0005829 cytosol; GO:0005886 plasma membrane; GO:0016020 membrane; GO:0044424 intracellular part; GO:0044444 cytoplasmic part; GO:0044464 cell part; GO:0071944 cell periphery;				S	KOG0619	FOG: Leucine rich repeat
Q9Y3D2	Methionine-R-sulfoxide reductase B2, mitochondrial OS=Homo sapiens OX=9606 GN=MSRB2 PE=1 SV=2	MSRB2	0.756	0.049481355	Down	K07305	msrB; peptide-methionine (R)-S-oxide reductase [EC:1.8.4.12]		GO:0006355 regulation of transcription, DNA-templated; GO:0006807 nitrogen compound metabolic process; GO:0006996 organelle organization; GO:0007010 cytoskeleton organization; GO:0007015 actin filament organization; GO:0008152 metabolic	GO:0005622 intracellular; GO:0005623 cell; GO:0005737 cytoplasm; GO:0005739 mitochondrion; GO:0005829 cytosol; GO:0043226 organelle; GO:0043227 membrane-bounded organelle; GO:0043229 intracellular organelle; GO:0043231 intracellular	GO:0003700 transcription factor activity, sequence-specific DNA binding; GO:0003779 actin binding; GO:0003824 catalytic activity; GO:0005488 binding; GO:0005515 protein binding; GO:0008092 cytoskeletal protein binding; GO:0008270 zinc ion binding; GO:0016491 oxidoreduc			O	KOG0856	Predicted pilin-like transcription factor
P49593	Protein phosphatase 1F OS=Homo sapiens OX=9606 GN=PPM1F PE=1 SV=3	PPM1F	0.755	0.044480894	Down	K17502	PPM1F, POPX2; protein phosphatase 1F [EC:3.1.3.16]		GO:0001932 regulation of protein phosphorylation; GO:0001933 negative regulation of protein phosphorylation; GO:0001952 regulation of cell-matrix adhesion; GO:0001954 positive regulation of cell-matrix adhesion; GO:0006325 chromatin organization; GO:0006355	GO:0005622 intracellular; GO:0005623 cell; GO:0005737 cytoplasm; GO:0005829 cytosol; GO:0032991 macromolecular complex; GO:0044424 intracellular part; GO:0044444 cytoplasmic part; GO:0044464 cell part; GO:0048471 perinuclear region of	GO:0003824 catalytic activity; GO:0004721 phosphoprotein phosphatase activity; GO:0004722 protein serine/threonine phosphatase activity; GO:0004723 calcium-dependent protein serine/threonine phosphatase activity; GO:0016787 hydrolase activity; GO:0016788 hydrolase			T	KOG0698	Serine/threonine protein phosphatase
Q9C0C2	182 kDa tankyrase-1-binding protein OS=Homo sapiens OX=9606 GN=TNKS1BP1 PE=1 SV=4	TNKS1BP1	0.755	0.01374415	Down				GO:0000075 cell cycle checkpoint; GO:0000077 DNA damage checkpoint; GO:0000278 mitotic cell cycle; GO:0000288 nuclear-transcribed mRNA catabolic process, deadenylation-dependent decay; GO:0000289 nuclear-transcribed mRNA poly(A) tail shortening; GO:0000723 telomere maintenanc	GO:0000228 nuclear chromosome; GO:0000785 chromatin; GO:0000790 nuclear chromatin; GO:0000792 heterochromatin; GO:0005622 intracellular; GO:0005623 cell; GO:0005634 nucleus; GO:0005694 chromosome; GO:0005720 nuclear heterochromatin; GO:00057	GO:0005488 binding; GO:0005515 protein binding; GO:0019899 enzyme binding; GO:0019904 protein domain specific binding; GO:0044877 macromolecular complex binding; GO:0071532 ankyrin repeat binding;					
Q96NA2	Rab-interacting lysosomal protein OS=Homo sapiens OX=9606 GN=RILP PE=1 SV=1	RILP	0.753	0.02552364	Down	K13883	RILP; Rab-interacting lysosomal protein	map04145 Phagosome; map05132 Salmonella infection	GO:0002376 immune system process; GO:0002478 antigen processing and presentation of exogenous peptide antigen; GO:0002495 antigen processing and presentation of peptide antigen via MHC class II; GO:0002504 antigen processing and presentation of peptide or polysaccha	GO:0000323 lytic vacuole; GO:0005622 intracellular; GO:0005623 cell; GO:0005737 cytoplasm; GO:0005764 lysosome; GO:0005765 lysosomal membrane; GO:0005768 endosome; GO:0005770 late endosome; GO:0005773 vacuole; GO:0005774 vacuolar membrane;	GO:0005488 binding; GO:0005515 protein binding; GO:0017016 Ras GTPase binding; GO:0017137 Rab GTPase binding; GO:0019899 enzyme binding; GO:0031267 small GTPase binding; GO:0051020 GTPase binding; GO:0051959 dynein light intermediate chain binding;					

Q8WW12	PEST proteolytic signal-containing nuclear protein OS=Homo sapiens OX=9606 GN=PCNP PE=1 SV=2	PCNP	0.753	0.019966901	Down				GO:0006464 cellular protein modification process; GO:0006508 proteolysis; GO:0006511 ubiquitin-dependent protein catabolic process; GO:0006807 nitrogen compound metabolic process; GO:0008152 metabolic process; GO:0009056 catabolic process; GO:0009057 macromole	GO:0005622 intracellular; GO:0005623 cell; GO:0005634 nucleus; GO:0005654 nucleoplasm; GO:0016604 nuclear body; GO:0031974 membrane-enclosed lumen; GO:0031981 nuclear lumen; GO:0043226 organelle; GO:0043227 membrane-bounded organelle;						
Q8IVF2	Protein AHNAK2 OS=Homo sapiens OX=9606 GN=AHNAK2 PE=1 SV=2	AHNAK2	0.753	0.00414078	Down					GO:0005622 intracellular; GO:0005623 cell; GO:0005737 cytoplasm; GO:0005829 cytosol; GO:0005886 plasma membrane; GO:0012506 vesicle membrane; GO:0016020 membrane; GO:0030016 myofibril; GO:0030017 sarcomere; GO:0030018 Z disc; GO:0030315 T-tubule;						
P26373	60S ribosomal protein L13 OS=Homo sapiens OX=9606 GN=RPL13 PE=1 SV=4	RPL13	0.751	0.041146451	Down	K02873	RP-L13e, RPL13; large subunit ribosomal protein L13e	map03010 Ribosome	GO:0000184 nuclear-transcribed mRNA catabolic process, nonsense-mediated decay; GO:0000956 nuclear-transcribed mRNA catabolic process; GO:0001889 liver development; GO:0006139 nucleobase-containing compound metabolic process; GO:0006401 RNA catabolic process; GO:00064	GO:0005622 intracellular; GO:0005623 cell; GO:0005634 nucleus; GO:0005730 nucleolus; GO:0005737 cytoplasm; GO:0005783 endoplasmic reticulum; GO:0005829 cytosol; GO:0005840 ribosome; GO:0012505 endomembrane system; GO:0015934 large	GO:0003676 nucleic acid binding; GO:0003723 RNA binding; GO:0003735 structural constituent of ribosome; GO:0005198 structural molecule activity; GO:0005488 binding; GO:0097159 organic cyclic compound binding; GO:1901363 heterocyclic compound binding;			J	KOG3295	60S Ribosomal protein L13
Q16186	Proteasomal ubiquitin receptor ADRM1 OS=Homo sapiens OX=9606 GN=ADRM1 PE=1 SV=2	ADRM1	0.751	0.046869881	Down	K06691	RPN13; 26S proteasome regulatory subunit N13	map03050 Proteasome; map05169 Epstein-Barr virus infection	GO:0000003 reproduction; GO:0001541 ovarian follicle development; GO:0002064 epithelial cell development; GO:0002376 immune system process; GO:0002520 immune system development; GO:0002682 regulation of immune system process; GO:00026	GO:0000502 proteasome complex; GO:0005622 intracellular; GO:0005623 cell; GO:0005634 nucleus; GO:0005654 nucleoplasm; GO:0005737 cytoplasm; GO:0005829 cytosol; GO:0005838 proteasome regulatory particle; GO:0005886 plasma membrane; GO:0008541	GO:0002020 protease binding; GO:0005488 binding; GO:0005515 protein binding; GO:0008047 enzyme activator activity; GO:0016504 peptidase activator activity; GO:0019899 enzyme binding; GO:0030234 enzyme regulator activity; GO:0032182 ubiquitin-like protein binding; GO:0043130			K	KOG3037	Cell membrane glycoprotein
Q8N394	Protein O-mannosyl-transferase TMTC2 OS=Homo sapiens OX=9606 GN=TMTC2 PE=1 SV=1	TMTC2	0.751	0.003926591	Down	K23424	TMTC; protein O-mannosyl-transferase [EC:2.4.1.-]		GO:0042592 homeostatic process; GO:0048878 chemical homeostasis; GO:0050801 ion homeostasis; GO:0055065 metal ion homeostasis; GO:0055074 calcium ion homeostasis; GO:0055080 cation homeostasis; GO:0065007 biological regulation;	GO:0005622 intracellular; GO:0005623 cell; GO:0005737 cytoplasm; GO:0005783 endoplasmic reticulum; GO:0005789 endoplasmic reticulum membrane; GO:0012505 endomembrane system; GO:0016020 membrane; GO:0031984 organelle subcompart				S	KOG1124	FOG: TPR repeat

Q01650	Large neutral amino acids transporter small subunit 1 OS=Homo sapiens OX=9606 GN=SLC7A5 PE=1 SV=2	SLC7A5	0.75	0.000669296	Down	K13780	SLC7A5, LAT1; solute carrier family 7 (L-type amino acid transporter), member 5	map04150 mTOR signaling pathway; map05230 Central carbon metabolism in cancer	GO:0002376 immune system process; GO:0003333 amino acid transmembrane transport; GO:0006082 organic acid metabolic process; GO:0006520 cellular amino acid metabolic process; GO:0006807 nitrogen compound metabolic process; GO:0006810 transport; GO:0006811 ion transport;	GO:0005622 intracellular; GO:0005623 cell; GO:0005737 cytoplasm; GO:0005829 cytosol; GO:0005886 plasma membrane; GO:0005887 integral component of plasma membrane; GO:0016020 membrane; GO:0016021 integral component of membrane; GO:0016324 apical plasma membrane;	GO:0003823 antigen binding; GO:0005215 transporter activity; GO:0005342 organic acid transmembrane transporter activity; GO:0005488 binding; GO:0008509 anion transmembrane transporter activity; GO:0008514 organic anion transmembrane transporter activity; GO:0015075 ion			P	KOG1287	Amino acid transporters
Q96NU1	Sterile alpha motif domain-containing protein 11 OS=Homo sapiens OX=9606 GN=SAMD11 PE=1 SV=3	SAMD11	0.75	0.004615232	Down							PF07647	SAM domain (Sterile alpha motif)	S	KOG4333	Nuclear DEAF-1 related transcriptional regulator (suppressin) and related SAND domain proteins
Q8TEK3	Histone-lysine N-methyltransferase, H3 lysine-79 specific OS=Homo sapiens OX=9606 GN=DOT1L PE=1 SV=3	DOT1L	0.749	0.001058601	Down	K11427	DOT1L, DOT1; [histone H3]-lysine79 N-trimethyltransferase [EC:2.1.1.360]	map00310 Lysine degradation; map01100 Metabolic pathways; map05202 Transcriptional misregulation in cancer	GO:0000075 cell cycle checkpoint; GO:0000077 DNA damage checkpoint; GO:0006139 nucleobase-containing compound metabolic process; GO:0006259 DNA metabolic process; GO:0006281 DNA repair; GO:0006325 chromatin organization; GO:0006342 chromatin silencing;	GO:0005622 intracellular; GO:0005623 cell; GO:0005634 nucleus; GO:0005654 nucleoplasm; GO:0031974 membrane-enclosed lumen; GO:0031981 nuclear lumen; GO:0032991 macromolecular complex; GO:0043226 organelle; GO:0043227 membrane-	GO:0003824 catalytic activity; GO:0005488 binding; GO:0005515 protein binding; GO:0008134 transcription factor binding; GO:0008168 methyltransferase activity; GO:0008170 N-methyltransferase activity; GO:0008276 protein methyltransferase activity; GO:0008757 S-adenosylm	PF08123	Histone methylation protein DOT1	B	KOG3924	Putative protein methyltransferase involved in meiosis and transcriptional silencing (Dot1)
O15234	Protein CASC3 OS=Homo sapiens OX=9606 GN=CASC3 PE=1 SV=2	CASC3	0.749	0.001625446	Down	K14323	CASC3, MLN51; protein CASC3	map03013 RNA transport; map03015 mRNA surveillance pathway	GO:0000184 nuclear-transcribed mRNA catabolic process, nonsense-mediated decay; GO:0000375 RNA splicing, via transesterification reactions; GO:0000377 RNA splicing, via transesterification reactions with bulged adenosine as nucleophile; GO:0000398 mRNA	GO:0005622 intracellular; GO:0005623 cell; GO:0005634 nucleus; GO:0005635 nuclear envelope; GO:0005654 nucleoplasm; GO:0005737 cytoplasm; GO:0005829 cytosol; GO:0012505 endomembrane system; GO:0016020 membrane; GO:0031090 organelle	GO:0005488 binding; GO:0005515 protein binding; GO:0019899 enzyme binding; GO:0031625 ubiquitin protein ligase binding; GO:0042802 identical protein binding; GO:0044389 ubiquitin-like protein ligase binding;	PF09405	CASC3/Barentsz eIF4AIII binding	A	KOG4264	Nucleo-cytoplasmic protein MLN51
O60551	Glycylpeptide N-tetradecanoyltransferase 2 OS=Homo sapiens OX=9606 GN=NMT2 PE=1 SV=1	NMT2	0.747	0.015150376	Down	K00671	NMT; glycylpeptide N-tetradecanoyltransferase [EC:2.3.1.97]		GO:0006464 cellular protein modification process; GO:0006497 protein lipidation; GO:0006498 N-terminal protein lipidation; GO:0006499 N-terminal protein myristoylation; GO:0006807 nitrogen compound metabolic process; GO:0006810 transport; GO:0008152 metabolic process;	GO:0005622 intracellular; GO:0005623 cell; GO:0005737 cytoplasm; GO:0005794 Golgi apparatus; GO:0005829 cytosol; GO:0005886 plasma membrane; GO:0012505 endomembrane system; GO:0016020 membrane; GO:0019898 extrinsic component of membrane;	GO:0003824 catalytic activity; GO:0004379 glycylpeptide N-tetradecanoyltransferase activity; GO:0016410 N-acyltransferase activity; GO:0016740 transferase activity; GO:0016746 transferase activity, transferring acyl groups; GO:0016747 transferase activity, transferring	PF01233; PF02799	Myristoyl-CoA:protein N-myristoyltransferase, N-terminal domain; Myristoyl-CoA:protein N-myristoyltransferase, C-terminal domain	I	KOG2779	N-myristoyl transferase

P48681	Nestin OS=Homo sapiens OX=9606 GN=NES PE=1 SV=2	NES	0.746	0.02548016	Down	K07609	NES; nestin		GO:0000086 G2/M transition of mitotic cell cycle; GO:0000278 mitotic cell cycle; GO:0000902 cell morphogenesis; GO:0001654 eye development; GO:0007049 cell cycle; GO:0007275 multicellular organism development; GO:0007399 nervous system development;	GO:0005622 intracellular; GO:0005623 cell; GO:0005737 cytoplasm; GO:0005856 cytoskeleton; GO:0005882 intermediate filament; GO:0043226 organelle; GO:0043228 non-membrane-bounded organelle; GO:0043229 intracellular organelle; GO:0043232	GO:0001664 G-protein coupled receptor binding; GO:0005102 receptor binding; GO:0005126 cytokine receptor binding; GO:0005488 binding; GO:0005515 protein binding; GO:0019215 intermediate filament binding; GO:0031730 CCR5 chemokine receptor binding; GO:0042379 chemokine					
Q6IE81	Protein Jade-1 OS=Homo sapiens OX=9606 GN=JADE1 PE=1 SV=1	JADE1	0.746	0.005220857	Down	K11347	JADE1, PHF17; protein Jade-1		GO:0001558 regulation of cell growth; GO:0006325 chromatin organization; GO:0006464 cellular protein modification process; GO:0006473 protein acetylation; GO:0006475 internal protein amino acid acetylation; GO:0006807 nitrogen compound metabolic process; GO:0006915 apoptotic	GO:0000123 histone acetyltransferase complex; GO:0005622 intracellular; GO:0005623 cell; GO:0005634 nucleus; GO:0005654 nucleoplasm; GO:0005737 cytoplasm; GO:0005815 microtubule organizing center; GO:0005829 cytosol; GO:0005856 cytoskeleto	GO:0003712 transcription cofactor activity; GO:0003713 transcription coactivator activity;	PF13832	PHD-zinc-finger like domain	K	KOG0954	PHD finger protein
P08195	4F2 cell-surface antigen heavy chain OS=Homo sapiens OX=9606 GN=SLC3A2 PE=1 SV=3	SLC3A2	0.745	0.007222384	Down	K06519	SLC3A2, MDU1, CD98; solute carrier family 3, member 2	map04150 mTOR signaling pathway; map04216 Ferroptosis; map04974 Protein digestion and absorption	GO:0002376 immune system process; GO:0003333 amino acid transmembrane transport; GO:0006810 transport; GO:0006811 ion transport; GO:0006812 cation transport; GO:0006814 sodium ion transport; GO:0006816 calcium ion transport; GO:0006820 anion transport; GO:00068	GO:0005622 intracellular; GO:0005623 cell; GO:0005634 nucleus; GO:0005737 cytoplasm; GO:0005829 cytosol; GO:0005886 plasma membrane; GO:0009986 cell surface; GO:0016020 membrane; GO:0016021 integral component of membrane; GO:0016324 apical plasma membrane;	GO:0003676 nucleic acid binding; GO:0003723 RNA binding; GO:0003725 double-stranded RNA binding; GO:0005215 transporter activity; GO:0005342 organic acid transmembrane transporter activity; GO:0005432 calcium:sodium antiporter activity; GO:0005488 binding;	PF00128	Alpha amylase, catalytic domain	P	KOG0471	Alpha-amylase
Q99650	Oncostatin-M-specific receptor subunit beta OS=Homo sapiens OX=9606 GN=OSMR PE=1 SV=1	OSMR	0.741	0.010984443	Down	K05057	OSMR; oncostatin M receptor	map04060 Cytokine-cytokine receptor interaction; map04151 PI3K-Akt signaling pathway; map04630 JAK-STAT signaling pathway	GO:0002673 regulation of acute inflammatory response; GO:0002675 positive regulation of acute inflammatory response; GO:0007154 cell communication; GO:0007165 signal transduction; GO:0007166 cell surface receptor signaling pathway; GO:0008284 positive regulation	GO:0005623 cell; GO:0005886 plasma membrane; GO:0005887 integral component of plasma membrane; GO:0005900 oncostatin-M receptor complex; GO:0009897 external side of plasma membrane; GO:0009986 cell surface; GO:0016020 membrane; GO:0016021 integral component of membrane;	GO:0004888 transmembrane signaling receptor activity; GO:0004896 cytokine receptor activity; GO:0004924 oncostatin-M receptor activity; GO:0005488 binding; GO:0005515 protein binding; GO:0019838 growth factor binding; GO:0019955 cytokine binding; GO:0038023 signaling	PF00041	Fibronectin type III domain			
P28715	DNA repair protein complementing XP-G cells OS=Homo sapiens OX=9606 GN=ERCC5 PE=1 SV=3	ERCC5	0.741	0.022875289	Down	K10846	ERCC5, XPG, RAD2; DNA excision repair protein ERCC-5	map03420 Nucleotide excision repair	GO:0006139 nucleobase-containing compound metabolic process; GO:0006259 DNA metabolic process; GO:0006281 DNA repair; GO:0006283 transcription-coupled nucleotide-excision repair; GO:0006289 nucleotide-excision repair; GO:0006293 nucleotide-excision repair,	GO:0005622 intracellular; GO:0005623 cell; GO:0005634 nucleus; GO:0005654 nucleoplasm; GO:0031974 membrane-enclosed lumen; GO:0031981 nuclear lumen; GO:0043226 organelle; GO:0043227 membrane-bounded organelle; GO:0043229 intracellula	GO:0000217 DNA secondary structure binding; GO:0000405 bubble DNA binding; GO:0003676 nucleic acid binding; GO:0003677 DNA binding; GO:0003690 double-stranded DNA binding; GO:0003697 single-stranded DNA binding; GO:0003824 catalytic activity; GO:0004518			L	KOG2520	5'-3' exonuclease

Q9Y5B8	Nucleoside diphosphate kinase 7 OS=Homo sapiens OX=9606 GN=NME7 PE=1 SV=1	NME7	0.738	0.014811715	Down	K00940	ndk, NME; nucleoside-diphosphate kinase [EC:2.7.4.6]	map00230 Purine metabolism; map00240 Pyrimidine metabolism; map00983 Drug metabolism - other enzymes; map01100 Metabolic pathways	GO:0003002 regionalization; GO:0003341 cilium movement; GO:0003351 epithelial cilium movement; GO:0006139 nucleobase-containing compound metabolic process; GO:0006165 nucleoside diphosphate phosphorylation; GO:0006725 cellular aromatic compound metabolic	GO:0005622 intracellular; GO:0005623 cell; GO:0005737 cytoplasm; GO:0005813 centrosome; GO:0005815 microtubule organizing center; GO:0005829 cytosol; GO:0005856 cytoskeleton; GO:0005929 cilium; GO:0015630 microtubule	GO:0003824 catalytic activity; GO:0004550 nucleoside diphosphate kinase activity; GO:0016301 kinase activity; GO:0016740 transferase activity; GO:0016772 transferase activity, transferring phosphorus-containing groups; GO:0016776 phosphotransferase activity, phosphate group as	PF00334	Nucleoside diphosphate kinase	F	KOG0888	Nucleoside diphosphate kinase
O60869	Endothelial differentiation-related factor 1 OS=Homo sapiens OX=9606 GN=EDF1 PE=1 SV=1	EDF1	0.738	0.047431613	Down	K03627	MBF1; putative transcription factor		GO:0003158 endothelium development; GO:0006355 regulation of transcription, DNA-templated; GO:0007275 multicellular organism development; GO:0009888 tissue development; GO:0009889 regulation of biosynthetic process; GO:00098	GO:0005622 intracellular; GO:0005623 cell; GO:0005634 nucleus; GO:0005730 nucleolus; GO:0005737 cytoplasm; GO:0005829 cytosol; GO:0031974 membrane-enclosed lumen; GO:0031981 nuclear lumen; GO:0043226 organelle; GO:0043227 membrane-bounded	GO:0003712 transcription cofactor activity; GO:0003713 transcription coactivator activity;	PF01381	Helix-turn-helix	K	KOG3398	Transcription factor MBF1
Q9Y2D5	A-kinase anchor protein 2 OS=Homo sapiens OX=9606 GN=AKAP2 PE=1 SV=3	AKAP2	0.738	0.005737448	Down	K16519	AKAP2; A-kinase anchor protein 2		GO:0006996 organelle organization; GO:0007010 cytoskeleton organization; GO:0007015 actin filament organization; GO:0007154 cell communication; GO:0007165 signal transduction; GO:0007166 cell surface receptor signaling pathway; GO:00071	GO:0032991 macromolecular complex;	GO:0005488 binding; GO:0005515 protein binding; GO:0019904 protein domain specific binding; GO:0051018 protein kinase A binding;					
Q8IY18	Structural maintenance of chromosomes protein 5 OS=Homo sapiens OX=9606 GN=SMC5 PE=1 SV=2	SMC5	0.738	0.047637392	Down	K22803	SMC5; structural maintenance of chromosomes protein 5		GO:0000722 telomere maintenance via recombination; GO:0000723 telomere maintenance; GO:0000724 double-strand break repair via homologous recombination; GO:0000725 recombinational repair; GO:0006139 nucleobase-containing compound	GO:0000781 chromosome, telomeric region; GO:0000793 condensed chromosome; GO:0000803 sex chromosome; GO:0005622 intracellular; GO:0005623 cell; GO:0005634 nucleus; GO:0005654 nucleoplasm; GO:0005694 chromosome;		PF02463	RecF/RecN/SMC N terminal domain	D	KOG0979	Structural maintenance of chromosome protein SMC5/Spr18, SMC superfamily
Q9H6F5	Coiled-coil domain-containing protein 86 OS=Homo sapiens OX=9606 GN=CCDC86 PE=1 SV=1	CCDC86	0.737	0.006752561	Down	K14822	CGR1; rRNA-processing protein CGR1			GO:0005622 intracellular; GO:0005623 cell; GO:0005634 nucleus; GO:0005730 nucleolus; GO:0031974 membrane-enclosed lumen; GO:0031981 nuclear lumen; GO:0043226 organelle; GO:0043227 membrane-bounded organelle; GO:0043228 non-membrane-bounded				S	KOG4538	Predicted coiled-coil protein

Q96B23	Uncharacterized protein C18orf25 OS=Homo sapiens OX=9606 GN=C18orf25 PE=1 SV=3	C18orf25	0.737	0.015326506	Down				GO:0000209 protein polyubiquitination; GO:0006464 cellular protein modification process; GO:0006508 proteolysis; GO:0006511 ubiquitin-dependent protein catabolic process; GO:0006807 nitrogen compound metabolic process; GO:0008152 metabolic process; GO:0009056 catabolic		GO:0003824 catalytic activity; GO:0004842 ubiquitin-protein transferase activity; GO:0016740 transferase activity; GO:0019787 ubiquitin-like protein transferase activity; GO:0061630 ubiquitin protein ligase activity; GO:0061659 ubiquitin-like protein ligase activity;					
P15509	Granulocyte-macrophage colony-stimulating factor receptor subunit alpha OS=Homo sapiens OX=9606 GN=CSF2RA PE=1 SV=1	CSF2RA	0.736	0.04150129	Down	K05066	CSF2RA, GMCSFR, CD116; granulocyte-macrophage colony-stimulating factor receptor alpha	map04060 Cytokine-cytokine receptor interaction; map04630 JAK-STAT signaling pathway; map04640 Hematopoietic cell lineage; map05200 Pathways in cancer	GO:0000165 MAPK cascade; GO:0006464 cellular protein modification process; GO:0006468 protein phosphorylation; GO:0006793 phosphorus metabolic process; GO:0006796 phosphate-containing compound metabolic process; GO:0006807 nitrogen compound metabolic process; GO:0007154 cell	GO:0005623 cell; GO:0005886 plasma membrane; GO:0005887 integral component of plasma membrane; GO:0016020 membrane; GO:0016021 integral component of membrane; GO:0031224 intrinsic component of membrane; GO:0031226 intrinsic component of plasma membrane; GO:0044425 membrane	GO:0003824 catalytic activity; GO:0004672 protein kinase activity; GO:0004713 protein tyrosine kinase activity; GO:0004888 transmembrane signaling receptor activity; GO:0004896 cytokine receptor activity; GO:0004902 granulocyte colony-stimulating factor receptor activity;	PF09240	Interleukin-6 receptor alpha chain, binding			
P09038	Fibroblast growth factor 2 OS=Homo sapiens OX=9606 GN=FGF2 PE=1 SV=3	FGF2	0.736	0.016379953	Down	K18497	FGF2; fibroblast growth factor 2	map01521 EGFR tyrosine kinase inhibitor resistance; map04010 MAPK signaling pathway; map04014 Ras signaling pathway; map04015 Rap1 signaling pathway; map04151 PI3K-Akt signaling pathway; map04550 Signaling pathways regulating pluripotency of stem cells; map04810 Regulation of actin cytoskeleton; map05167 Kaposi sarcoma-associated herpesvirus infection; map05200 Pathways in cancer; map05205 Proteoglycans in cancer; map05218 Melanoma; map05224 Breast cancer; map05226 Gastric cancer	GO:0000165 MAPK cascade; GO:0000186 activation of MAPKK activity; GO:0000187 activation of MAPK activity; GO:0001501 skeletal system development; GO:0001525 angiogenesis; GO:0001568 blood vessel development; GO:0001655 urogenital	GO:0005576 extracellular region; GO:0005615 extracellular space; GO:0005622 intracellular; GO:0005623 cell; GO:0005634 nucleus; GO:0005737 cytoplasm; GO:0043226 organelle; GO:0043227 membrane-bounded organelle; GO:0043229 intracellular organelle;	GO:0003712 transcription cofactor activity; GO:0003713 transcription coactivator activity; GO:0003824 catalytic activity; GO:0004672 protein kinase activity; GO:0004713 protein tyrosine kinase activity; GO:0005085 guanyl-nucleotide exchange factor activity; GO:0005088 Ras	PF00167	Fibroblast growth factor	K	KOG3885	Fibroblast growth factor
Q3B7T1	Erythroid differentiation-related factor 1 OS=Homo sapiens OX=9606 GN=EDRF1 PE=1 SV=1	EDRF1	0.735	0.031804229	Down				GO:0006355 regulation of transcription, DNA-templated; GO:0009889 regulation of biosynthetic process; GO:0009891 positive regulation of biosynthetic process; GO:0009893 positive regulation of metabolic process; GO:0010468 regulation of gene expression; GO:00105							
P46821	Microtubule-associated protein 1B OS=Homo sapiens OX=9606 GN=MAP1B PE=1 SV=2	MAP1B	0.733	0.001275953	Down	K10429	MAP1; microtubule-associated protein 1		GO:0000226 microtubule cytoskeleton organization; GO:0000902 cell morphogenesis; GO:0000904 cell morphogenesis involved in differentiation; GO:0001558 regulation of cell growth; GO:0001578 microtubule bundle formation; GO:0001764 neuron	GO:0001750 photoreceptor outer segment; GO:0005622 intracellular; GO:0005623 cell; GO:0005737 cytoplasm; GO:0005829 cytosol; GO:0005856 cytoskeleton; GO:0005874 microtubule; GO:0005875 microtubule associated complex; GO:00058	GO:0003779 actin binding; GO:0005198 structural molecule activity; GO:0005488 binding; GO:0005515 protein binding; GO:0005519 cytoskeletal regulatory protein binding; GO:0005543 phospholipid binding; GO:0008017 microtubule binding; GO:0008092 cytoskeletal protein			Z	KOG3592	Microtubule-associated proteins

Q86YV5	Inactive tyrosine-protein kinase PRAG1 OS=Homo sapiens OX=9606 GN=PRAG1 PE=1 SV=4	PRAG1	0.733	0.013385214	Down	K17537	SGK223; tyrosine-protein kinase SgK223 [EC:2.7.10.2]		GO:0006464 cellular protein modification process; GO:0006468 protein phosphorylation; GO:0006793 phosphorus metabolic process; GO:0006796 phosphate-containing compound metabolic process; GO:0006807 nitrogen compound metabolic process; GO:0006928 movement of cell or subcellular	GO:0005622 intracellular; GO:0005623 cell; GO:0005634 nucleus; GO:0005737 cytoplasm; GO:0005912 adherens junction; GO:0005924 cell-substrate adherens junction; GO:0005925 focal adhesion; GO:0030054 cell junction; GO:0030055 cell-substrate junction; GO:00432	GO:0003824 catalytic activity; GO:0004672 protein kinase activity; GO:0005488 binding; GO:0005515 protein binding; GO:0016301 kinase activity; GO:0016740 transferase activity; GO:0016772 transferase activity, transferring phosphorus-containing groups; GO:0016773 phosphotransferase	PF00069	Protein kinase domain			
Q7Z2Y8	Interferon-induced very large GTPase 1 OS=Homo sapiens OX=9606 GN=GVINP1 PE=2 SV=2	GVINP1	0.732	0.000636488	Down											
Q9H8X2	Inositol-pentakisphosphate 2-kinase OS=Homo sapiens OX=9606 GN=IPPK PE=1 SV=1	IPPK	0.731	0.020600823	Down	K10572	IPPK; inositol-pentakisphosphate 2-kinase [EC:2.7.1.158]	map00562 Inositol phosphate metabolism; map01100 Metabolic pathways; map04070 Phosphatidylinositol signaling system	GO:0005975 carbohydrate metabolic process; GO:0006020 inositol metabolic process; GO:0006066 alcohol metabolic process; GO:0006355 regulation of transcription, DNA-templated; GO:0006356 regulation of transcription from RNA polymerase I promoter; GO:00067	GO:0005622 intracellular; GO:0005623 cell; GO:0005634 nucleus; GO:0005654 nucleoplasm; GO:0005730 nucleolus; GO:0005737 cytoplasm; GO:0005829 cytosol; GO:0031974 membrane-enclosed lumen; GO:0031981 nuclear lumen; GO:0043226 organelle;	GO:0003824 catalytic activity; GO:0005198 structural molecule activity; GO:0005488 binding; GO:0005515 protein binding; GO:0016301 kinase activity; GO:0016740 transferase activity; GO:0016772 transferase activity, transferring phosphorus-containing groups; GO:0016773 phosphotra			T	KOG4749	Inositol polyphosphate kinase
Q96RK0	Protein capicua homolog OS=Homo sapiens OX=9606 GN=CIC PE=1 SV=2	CIC	0.731	0.005290422	Down	K20225	CIC; capicua transcriptional repressor	map04013 MAPK signaling pathway - fly	GO:0000122 negative regulation of transcription from RNA polymerase II promoter; GO:0003008 system process; GO:0006355 regulation of transcription, DNA-templated; GO:0006357 regulation of transcription from RNA polymerase II promoter; GO:00072	GO:0005622 intracellular; GO:0005623 cell; GO:0005634 nucleus; GO:0005654 nucleoplasm; GO:0031974 membrane-enclosed lumen; GO:0031981 nuclear lumen; GO:0032991 macromolecular complex; GO:0043226 organelle; GO:0043227 membrane-	GO:0000981 RNA polymerase II transcription factor activity, sequence-specific DNA binding; GO:0003682 chromatin binding; GO:0003700 transcription factor activity, sequence-specific DNA binding; GO:0005488 binding;	PF00505	HMG (high mobility group) box	K	KOG2746	HMG-box transcription factor Capicua and related proteins
O75113	NEDD4-binding protein 1 OS=Homo sapiens OX=9606 GN=N4BP1 PE=1 SV=4	N4BP1	0.729	0.045671355	Down				GO:0009314 response to radiation; GO:0009411 response to UV; GO:0009416 response to light stimulus; GO:0009628 response to abiotic stimulus; GO:0009892 negative regulation of metabolic process; GO:0009894 regulation of catabolic process; GO:00098	GO:0005622 intracellular; GO:0005623 cell; GO:0005634 nucleus; GO:0005654 nucleoplasm; GO:0005730 nucleolus; GO:0016604 nuclear body; GO:0016605 PML body; GO:0031974 membrane-enclosed lumen; GO:0031981 nuclear lumen; GO:0043226		PF11977	Zc3h12a-like Ribonuclease NYN domain	S	KOG3777	Uncharacterized conserved protein
Q9Y376	Calcium-binding protein 39 OS=Homo sapiens OX=9606 GN=CAB39 PE=1 SV=1	CAB39	0.727	0.048303117	Down	K08272	CAB39, MO25; calcium binding protein 39	map04150 mTOR signaling pathway; map04152 AMPK signaling pathway	GO:0001775 cell activation; GO:0001932 regulation of protein phosphorylation; GO:0001934 positive regulation of protein phosphorylation; GO:0002252 immune effector process; GO:0002263 cell activation involved in immune response; GO:0002274 myeloid leukocyte activation; GO:0002275 myeloid	GO:0005576 extracellular region; GO:0005622 intracellular; GO:0005623 cell; GO:0005737 cytoplasm; GO:0005829 cytosol; GO:0012505 endomembrane system; GO:0030141 secretory granule; GO:0031410 cytoplasmic vesicle; GO:0031974 membrane-	GO:0005488 binding; GO:0005509 calcium ion binding; GO:0005515 protein binding; GO:0008047 enzyme activator activity; GO:0019207 kinase regulator activity; GO:0019209 kinase activator activity; GO:0019887 protein kinase regulator activity; GO:0019899 enzyme binding; GO:0019900 kinase			S	KOG1566	Conserved protein Mo25

Q9Y3E7	Charged multivesicular body protein 3 OS=Homo sapiens OX=9606 GN=CHMP3 PE=1 SV=3	CHMP3	0.727	0.049842683	Down	K12193	VPS24, CHMP3; charged multivesicular body protein 3	map04144 Endocytosis; map04217 Necroptosis	GO:0000920 cell separation after cytokinesis; GO:0006810 transport; GO:0006996 organelle organization; GO:0007034 vacuolar transport; GO:0007041 lysosomal transport; GO:0008333 endosome to lysosome transport; GO:0009987 cellular process;	GO:0000815 ESCRT III complex; GO:0005622 intracellular; GO:0005623 cell; GO:0005737 cytoplasm; GO:0005768 endosome; GO:0005769 early endosome; GO:0005770 late endosome; GO:0005886 plasma membrane; GO:0010008 endosome membrane; GO:0012505	GO:0002020 protease binding; GO:0005488 binding; GO:0005515 protein binding; GO:0005543 phospholipid binding; GO:0005546 phosphatidylinositol-4,5-bisphosphate binding; GO:0008289 lipid binding; GO:0019899 enzyme binding; GO:0031210 phosphatidylcholine binding; GO:00350			U	KOG3229	Vacuolar sorting protein VPS24
Q9HAY6	Beta,beta-carotene 15,15'-dioxygenase OS=Homo sapiens OX=9606 GN=BCO1 PE=1 SV=1	BCO1	0.726	0.039917226	Down	K00515	BCMO1, BCDO1; beta-carotene 15,15'-dioxygenase [EC:1.13.11.63]	map00830 Retinol metabolism; map01100 Metabolic pathways	GO:0001523 retinoid metabolic process; GO:0006081 cellular aldehyde metabolic process; GO:0006629 lipid metabolic process; GO:0006720 isoprenoid metabolic process; GO:0006721 terpenoid metabolic process; GO:0006766 vitamin metabolic process; GO:0006775 fat-soluble vitamin	GO:0005622 intracellular; GO:0005623 cell; GO:0005737 cytoplasm; GO:0005829 cytosol; GO:0044424 intracellular part; GO:0044444 cytoplasmic part; GO:0044464 cell part;	GO:0003824 catalytic activity; GO:0003834 beta-carotene 15,15'-monooxygenase activity; GO:0016491 oxidoreductase activity; GO:0016701 oxidoreductase activity, acting on single donors with incorporation of molecular oxygen; GO:0016702 oxidoreduc			Q	KOG1285	Beta, beta-carotene 15,15'-dioxygenase and related enzymes
Q8NFT8	Delta and Notch-like epidermal growth factor-related receptor OS=Homo sapiens OX=9606 GN=DNER PE=1 SV=1	DNER	0.726	0.001583845	Down				GO:0001764 neuron migration; GO:0006807 nitrogen compound metabolic process; GO:0006810 transport; GO:0006897 endocytosis; GO:0006928 movement of cell or subcellular component; GO:0007154 cell communication; GO:0007165 signal transduction; GO:00072	GO:0005622 intracellular; GO:0005623 cell; GO:0005737 cytoplasm; GO:0005768 endosome; GO:0005769 early endosome; GO:0005886 plasma membrane; GO:0012505 endomembrane system; GO:0016020 membrane; GO:0030425 dendrite; GO:0031410	GO:0004888 transmembrane signaling receptor activity; GO:0005102 receptor binding; GO:0005112 Notch binding; GO:0005488 binding; GO:0005509 calcium ion binding; GO:0005515 protein binding; GO:0030276 clathrin binding; GO:0038023 signaling receptor activity; GO:00431	PF00008; PF12661	EGF-like domain; Human growth factor-like EGF	T	KOG1217	Fibrillins and related proteins containing Ca2+-binding EGF-like domains
Q15648	Mediator of RNA polymerase II transcription subunit 1 OS=Homo sapiens OX=9606 GN=MED1 PE=1 SV=4	MED1	0.725	0.020548549	Down	K15144	MED1; mediator of RNA polymerase II transcription subunit 1	map01522 Endocrine resistance; map04919 Thyroid hormone signaling pathway	GO:0000003 reproduction; GO:0000122 negative regulation of transcription from RNA	GO:0000151 ubiquitin ligase complex; GO:0000785 chromatin; GO:0005622 intracellula	GO:0000976 transcription regulatory region sequence-specific DNA binding; GO:00009	PF10744	Mediator of RNA polymerase II transcription subunit 1			
O43854	EGF-like repeat and discoidin I-like domain-containing protein 3 OS=Homo sapiens OX=9606 GN=EDIL3 PE=1 SV=1	EDIL3	0.725	0.017226913	Down				GO:0010810 regulation of cell-substrate adhesion; GO:0010811 positive regulation of cell-substrate adhesion; GO:0030155 regulation of cell adhesion; GO:0045785 positive regulation of cell adhesion; GO:0048518 positive regulation of biological process; GO:0050789 regulation	GO:0005576 extracellular region;	GO:0005102 receptor binding; GO:0005178 integrin binding; GO:0005488 binding; GO:0005515 protein binding; GO:0044877 macromolecular complex binding; GO:0050839 cell adhesion molecule binding;	PF00008; PF12661; PF00754	EGF-like domain; Human growth factor-like EGF; F5/8 type C domain			

Q9HCD6	Protein TANC2 OS=Homo sapiens OX=9606 GN=TANC2 PE=1 SV=3	TANC2	0.725	0.002981467	Down				GO:0001701 in utero embryonic development; GO:0007275 multicellular organism development; GO:0009790 embryo development; GO:0009792 embryo development ending in birth or egg hatching; GO:0032501 multicellular organismal process; GO:0032502					S	KOG0504	FOG: Ankyrin repeat
Q15629	Translocating chain-associated membrane protein 1 OS=Homo sapiens OX=9606 GN=TRAM1 PE=1 SV=3	TRAM1	0.723	0.010950675	Down	K14010	TRAM1; translocating chain-associated membrane protein 1	map04141 Protein processing in endoplasmic reticulum	GO:0006605 protein targeting; GO:0006612 protein targeting to membrane; GO:0006613 cotranslational protein targeting to membrane; GO:0006614 SRP-dependent cotranslational protein targeting to membrane; GO:0006616 SRP-dependent cotranslational protein targeting to membrane, translocation; GO:0006810	GO:0005622 intracellular; GO:0005623 cell; GO:0005737 cytoplasm; GO:0005783 endoplasmic reticulum; GO:0005789 endoplasmic reticulum membrane; GO:0012505 endomembrane system; GO:0016020 membrane; GO:0016021 integral component of	GO:0038023 signaling receptor activity; GO:0060089 molecular transducer activity;	PF03798	TLC domain	U	KOG1608	Protein transporter of the TRAM (translocating chain-associating membrane) superfamily
P11166	Solute carrier family 2, facilitated glucose transporter member 1 OS=Homo sapiens OX=9606 GN=SLC2A1 PE=1 SV=2	SLC2A1	0.718	0.020104943	Down	K07299	SLC2A1, GLUT1; MFS transporter, SP family, solute carrier family 2 (facilitated glucose transporter), member 1	map04066 HIF-1 signaling pathway; map04911 Insulin secretion; map04919 Thyroid hormone signaling pathway; map04920 Adipocytokine signaling pathway; map04922 Glucagon signaling pathway; map04931 Insulin resistance; map04976 Bile secretion; map05166 Human T-cell leukemia virus 1 infection; map05200 Pathways in cancer; map05211 Renal cell carcinoma; map05230 Central carbon metabolism in cancer	GO:0000003 reproduction; GO:0002791 regulation of peptide secretion; GO:0005975 carbohydrate metabolic process; GO:0005984 disaccharide metabolic process; GO:0005988 lactose metabolic process; GO:0005989 lactose biosynthetic process; GO:0005996 monosacch	GO:0000139 Golgi membrane; GO:0001939 female pronucleus; GO:0005622 intracellular; GO:0005623 cell; GO:0005634 nucleus; GO:0005737 cytoplasm; GO:0005794 Golgi apparatus; GO:0005829 cytosol; GO:0005856 cytoskeleton; GO:0005886 plasma membrane; GO:0005887 integral	GO:0005215 transporter activity; GO:0005355 glucose transmembrane transporter activity; GO:0005488 binding; GO:0005515 protein binding; GO:0008509 anion transmembrane transporter activity; GO:0008514 organic anion transmembrane transporter activity; GO:0015075 ion transmemb			P	KOG0569	Permease of the major facilitator superfamily
Q9HAW4	Claspin OS=Homo sapiens OX=9606 GN=CLSPN PE=1 SV=3	CLSPN	0.717	0.044406627	Down				GO:0000075 cell cycle checkpoint; GO:0000076 DNA replication checkpoint; GO:0000077 DNA damage checkpoint; GO:0000278 mitotic cell cycle; GO:0001932 regulation of protein phosphorylation; GO:0001934 positive regulation of protein phosphorylation; GO:0006139 nucleobase-containing	GO:0005622 intracellular; GO:0005623 cell; GO:0005634 nucleus; GO:0005654 nucleoplasm; GO:0005737 cytoplasm; GO:0005794 Golgi apparatus; GO:0012505 endomembrane system; GO:0031974 membrane-enclosed lumen; GO:0031981 nuclear lumen;	GO:0000217 DNA secondary structure binding; GO:0003676 nucleic acid binding; GO:0003677 DNA binding; GO:0005488 binding; GO:0010997 anaphase-promoting complex binding; GO:0044877 macromolecular complex binding; GO:0097159 organic cyclic compound binding;			DT	KOG4156	Claspin, protein mediating phosphorylation and activation of Chk1 protein kinase in the DNA replication checkpoint response
O14775	Guanine nucleotide-binding protein subunit beta-5 OS=Homo sapiens OX=9606 GN=GNB5 PE=1 SV=2	GNB5	0.716	0.045411646	Down	K04539	GNB5; guanine nucleotide-binding protein subunit beta-5	map04014 Ras signaling pathway; map04062 Chemokine signaling pathway; map04151 PI3K-Akt signaling pathway; map04371 Apelin signaling pathway; map04713 Circadian entrainment; map04723 Retrograde endocannabinoid signaling; map04724 Glutamatergic synapse; map04725 Cholinergic synapse; map04726 Serotonergic synapse; map04727 GABAergic synapse; map04728 Dopaminergic synapse; map04926 Relaxin signaling pathway; map05032 Morphine addiction; map05034 Alcoholism; map05163 Human cytomegalovirus infection; map05167 Kaposi sarcoma-associated herpesvirus infection; map05170 Human immunodeficiency virus 1 infection; map05200 Pathways in cancer	GO:0006457 protein folding; GO:0007154 cell communication; GO:0007165 signal transduction; GO:0007186 G-protein coupled receptor signaling pathway; GO:0007212 dopamine receptor signaling pathway; GO:0009987 cellular process; GO:0010959 regulation of metal	GO:0001750 photoreceptor outer segment; GO:0001917 photoreceptor inner segment; GO:0005622 intracellular; GO:0005623 cell; GO:0005634 nucleus; GO:0005737 cytoplasm; GO:0005829 cytosol; GO:0005886 plasma membrane; GO:0005929 cilium; GO:0016020 membrane;	GO:0003824 catalytic activity; GO:0003924 GTPase activity; GO:0005096 GTPase activator activity; GO:0005488 binding; GO:0005515 protein binding; GO:0008047 enzyme activator activity; GO:0016462 pyrophosphatase activity; GO:0016787 hydrolase activity; GO:0016817 hydrolase			S	KOG0286	G-protein beta subunit

Q15388	Mitochondrial import receptor subunit TOM20 homolog OS=Homo sapiens OX=9606 GN=TOMM20 PE=1 SV=1	TOMM20	0.716	0.023258657	Down	K17770	TOM20; mitochondrial import receptor subunit TOM20		GO:0006403 RNA localization; GO:0006464 cellular protein modification process; GO:0006508 proteolysis; GO:0006605 protein targeting; GO:0006626 protein targeting to mitochondrion; GO:0006807 nitrogen compound metabolic process; GO:0006810 transport; GO:0006839 mitochondr	GO:0005622 intracellular; GO:0005623 cell; GO:0005737 cytoplasm; GO:0005739 mitochondrion; GO:0005740 mitochondrial envelope; GO:0005741 mitochondrial outer membrane; GO:0005742 mitochondrial outer membrane translocase complex; GO:00160	GO:0005048 signal sequence binding; GO:0005215 transporter activity; GO:0005488 binding; GO:0005515 protein binding; GO:0008320 protein transmembrane transporter activity; GO:0008565 protein transporter activity; GO:0015399 primary active transmembrane transporter activity; GO:00154			U	KOG4056	Translocase of outer mitochondrial membrane complex, subunit TOM20
Q15942	Zyxin OS=Homo sapiens OX=9606 GN=ZYX PE=1 SV=1	ZYX	0.714	0.003372338	Down	K06273	ZYX; zyxin	map04510 Focal adhesion	GO:0002376 immune system process; GO:0006950 response to stress; GO:0006952 defense response; GO:0006955 immune response; GO:0006996 organelle organization; GO:0007010 cytoskeleton organization; GO:0007015 actin filament organization; GO:00071	GO:0001725 stress fiber; GO:0005622 intracellular; GO:0005623 cell; GO:0005634 nucleus; GO:0005737 cytoplasm; GO:0005829 cytosol; GO:0005856 cytoskeleton; GO:0005911 cell-cell junction; GO:0005912 adherens junction; GO:0005913 cell-cell adherens junction;		PF00412	LIM domain	T	KOG1704	FOG: LIM domain
P20810	Calpastatin OS=Homo sapiens OX=9606 GN=CAST PE=1 SV=4	CAST	0.712	0.024344129	Down	K04281	CAST; calpastatin	map05131 Shigellosis	GO:0000003 reproduction; GO:0000768 syncytium formation by plasma membrane fusion; GO:0001775 cell activation; GO:0001889 liver development; GO:0006807 nitrogen compound metabolic process; GO:0006949 syncytium formation; GO:0007275 multicellular	GO:0005622 intracellular; GO:0005623 cell; GO:0005634 nucleus; GO:0005737 cytoplasm; GO:0005783 endoplasmic reticulum; GO:0005829 cytosol; GO:0012505 endomembrane system; GO:0014069 postsynaptic density; GO:0016020 membrane; GO:00322	GO:0002020 protease binding; GO:0004857 enzyme inhibitor activity; GO:0004866 endopeptidase inhibitor activity; GO:0004869 cysteine-type endopeptidase inhibitor activity; GO:0005488 binding; GO:0005515 protein binding; GO:0010859 calcium-dependent cysteine-					
Q9Y2W3	Proton-associated sugar transporter A OS=Homo sapiens OX=9606 GN=SLC45A1 PE=1 SV=5	SLC45A1	0.71	0.008774677	Down	K15378	SLC45A1_2_4; solute carrier family 45, member 1/2/4		GO:0006810 transport; GO:0008643 carbohydrate transport; GO:0008645 hexose transport; GO:0015749 monosaccharide transport; GO:0034219 carbohydrate transmembrane transport; GO:0051179 localization; GO:0051234 establishment of		GO:0005215 transporter activity; GO:0005355 glucose transmembrane transporter activity; GO:0015144 carbohydrate transmembrane transporter activity; GO:0015145 monosaccharide transmembrane transporter activity; GO:0015149 hexose transmembrane transporter			G	KOG0637	Sucrose transporter and related proteins
Q8N960	Centrosomal protein of 120 kDa OS=Homo sapiens OX=9606 GN=CEP120 PE=1 SV=2	CEP120	0.71	0.025826082	Down	K16459	CEP120; centrosomal protein CEP120		GO:0000226 microtubule cytoskeleton organization; GO:0006810 transport; GO:0006996 organelle organization; GO:0007010 cytoskeleton organization; GO:0007017 microtubule-based process; GO:0007049 cell cycle; GO:00070	GO:0005622 intracellular; GO:0005623 cell; GO:0005737 cytoplasm; GO:0005813 centrosome; GO:0005814 centriole; GO:0005815 microtubule organizing center; GO:0005856 cytoskeleton; GO:0015630 microtubule cytoskeleto	GO:0005488 binding; GO:0005515 protein binding; GO:0008022 protein C-terminus binding;	PF00168	C2 domain			
Q68DH5	G-protein coupled receptor-associated protein LMBRD2 OS=Homo sapiens OX=9606 GN=LMBRD2 PE=1 SV=1	LMBRD2	0.709	0.048549265	Down									S	KOG2296	Integral membrane protein

P25054	Adenomatous polyposis coli protein OS=Homo sapiens OX=9606 GN=APC PE=1 SV=2	APC	0.707	0.007281816	Down	K02085	APC; adenomatosis polyposis coli protein	map04310 Wnt signaling pathway; map04390 Hippo signaling pathway; map04550 Signaling pathways regulating pluripotency of stem cells; map04810 Regulation of actin cytoskeleton; map04934 Cushing syndrome; map05165 Human papillomavirus infection; map05200 Pathways in cancer; map05206 MicroRNAs in cancer; map05210 Colorectal cancer; map05213 Endometrial cancer; map05217 Basal cell carcinoma; map05224 Breast cancer; map05225 Hepatocellular carcinoma; map05226 Gastric cancer	GO:0000075 cell cycle checkpoint; GO:0000079 regulation of cyclin-dependent protein serine/threonine kinase activity; GO:0000226 microtubule cytoskeleton organization; GO:0000278 mitotic cell cycle; GO:0000281 mitotic cytokinesis; GO:0000820	GO:0000775 chromosome, centromeric region; GO:0000776 kinetochore; GO:0001726 ruffle; GO:0005622 intracellular; GO:0005623 cell; GO:0005634 nucleus; GO:0005635 nuclear envelope; GO:0005654 nucleoplasm; GO:0005694 chromosome;	GO:0002020 protease binding; GO:0005488 binding; GO:0005515 protein binding; GO:0008013 beta-catenin binding; GO:0008017 microtubule binding; GO:0008092 cytoskeletal protein binding; GO:0015631 tubulin binding; GO:0019207 kinase regulator activity; GO:0019887 protein kinase			Z	KOG2122	Beta-catenin-binding protein APC, contains ARM repeats
P57088	Transmembrane protein 33 OS=Homo sapiens OX=9606 GN=TMEM33 PE=1 SV=2	TMEM33	0.707	0.029635651	Down	K20724	TMEM33; transmembrane protein 33		GO:0006810 transport; GO:0006913 nucleocytoplasmic transport; GO:0006950 response to stress; GO:0006996 organelle organization; GO:0006997 nucleus organization; GO:0006999 nuclear pore organization; GO:0007029 endoplasmic reticulum organizatio	GO:0005622 intracellular; GO:0005623 cell; GO:0005634 nucleus; GO:0005635 nuclear envelope; GO:0005643 nuclear pore; GO:0005737 cytoplasm; GO:0005783 endoplasmic reticulum; GO:0005789 endoplasmic reticulum membrane; GO:0012505 endomembrane	GO:0005198 structural molecule activity; GO:0017056 structural constituent of nuclear pore;			S	KOG4002	Uncharacterized integral membrane protein
Q8IWR1	Tripartite motif-containing protein 59 OS=Homo sapiens OX=9606 GN=TRIM59 PE=1 SV=1	TRIM59	0.706	0.023095927	Down	K12028	TRIM59; tripartite motif-containing protein 59		GO:0002376 immune system process; GO:0006464 cellular protein modification process; GO:0006807 nitrogen compound metabolic process; GO:0006950 response to stress; GO:0006952 defense response; GO:0006955 immune response; GO:0006996 organelle organization; GO:0008152	GO:0005622 intracellular; GO:0005623 cell; GO:0005737 cytoplasm; GO:0005783 endoplasmic reticulum; GO:0005813 centrosome; GO:0005815 microtubule organizing center; GO:0005856 cytoskeleton; GO:0005929 cilium; GO:00125	GO:0003824 catalytic activity; GO:0004842 ubiquitin-protein transferase activity; GO:0016740 transferase activity; GO:0019787 ubiquitin-like protein transferase activity; GO:0061630 ubiquitin protein ligase activity; GO:0061659 ubiquitin-like protein ligase activity;	PF13445; PF00643	RING-type zinc-finger, LisH dimerisation motif; B-box zinc finger	O	KOG2177	Predicted E3 ubiquitin ligase
Q6PK04	Coiled-coil domain-containing protein 137 OS=Homo sapiens OX=9606 GN=CCDC137 PE=1 SV=1	CCDC137	0.703	0.003689553	Down					GO:0001650 fibrillar center; GO:0005622 intracellular; GO:0005623 cell; GO:0005634 nucleus; GO:0005694 chromosome; GO:0005730 nucleolus; GO:0031974 membrane-enclosed lumen; GO:0031981 nuclear lumen; GO:0043226 organelle; GO:0043227						
Q6UX04	Spliceosome-associated protein CWC27 homolog OS=Homo sapiens OX=9606 GN=CWC27 PE=1 SV=1	CWC27	0.702	0.023804816	Down	K12737	SDCCAG10; peptidyl-prolyl cis-trans isomerase SDCCAG10 [EC:5.2.1.8]		GO:0000375 RNA splicing, via transesterification reactions; GO:0000377 RNA splicing, via transesterification reactions with bulged adenosine as nucleophile; GO:0000398 mRNA splicing, via spliceosome; GO:0000413 protein peptidyl-prolyl isomerizati	GO:0005622 intracellular; GO:0005623 cell; GO:0005634 nucleus; GO:0005654 nucleoplasm; GO:0005681 spliceosomal complex; GO:0031974 membrane-enclosed lumen; GO:0031981 nuclear lumen; GO:0032991 macromolecular complex; GO:0043226	GO:0003755 peptidyl-prolyl cis-trans isomerase activity; GO:0003824 catalytic activity; GO:0016853 isomerase activity; GO:0016859 cis-trans isomerase activity;	PF00160	Cyclophilin type peptidyl-prolyl cis-trans isomerase/CLD	A	KOG0885	Peptidyl-prolyl cis-trans isomerase

Q99418	Cytohesin-2 OS=Homo sapiens OX=9606 GN=CYTH2 PE=1 SV=2	CYTH2	0.702	0.016948812	Down	K18441	CYTH; cytohesin	map04072 Phospholipase D signaling pathway; map04144 Endocytosis; map05130 Pathogenic Escherichia coli infection	GO:0007275 multicellular organism development; GO:0007399 nervous system development; GO:0009987 cellular process; GO:0010721 negative regulation of cell development; GO:0010975 regulation of neuron projection development; GO:0010977 negative regulation	GO:0001726 ruffle; GO:0005622 intracellular; GO:0005623 cell; GO:0005737 cytoplasm; GO:0005886 plasma membrane; GO:0005911 cell-cell junction; GO:0005923 bicellular tight junction; GO:0016020 membrane; GO:0030054 cell junction; GO:0031252 cell leading	GO:0005085 guanyl-nucleotide exchange factor activity; GO:0005086 ARF guanyl-nucleotide exchange factor activity; GO:0005488 binding; GO:0005515 protein binding; GO:0019899 enzyme binding; GO:0036094 small molecule binding; GO:0043167 ion binding; GO:0043168 anion binding;	PF01369; PF00169	Sec7 domain; PH domain	L	KOG0930	Guanine nucleotide exchange factor Cytohesin, contains PH and Sec7 domains
Q9HD26	Golgi-associated PDZ and coiled-coil motif-containing protein OS=Homo sapiens OX=9606 GN=GOPC PE=1 SV=1	GOPC	0.702	0.027574767	Down				GO:0000003 reproduction; GO:0003006 developmental process involved in reproduction; GO:0006810 transport; GO:0006888 ER to Golgi vesicle-mediated transport; GO:0006892 post-Golgi vesicle-mediated transport; GO:0006893 Golgi to plasma membrane	GO:0000139 Golgi membrane; GO:0000323 lytic vacuole; GO:0005622 intracellular; GO:0005623 cell; GO:0005737 cytoplasm; GO:0005764 lysosome; GO:0005765 lysosomal membrane; GO:0005773 vacuole; GO:0005774 vacuolar membrane; GO:0005794 Golgi apparatus;	GO:0000149 SNARE binding; GO:0001664 G-protein coupled receptor binding; GO:0005102 receptor binding; GO:0005109 frizzled binding; GO:0005488 binding; GO:0005515 protein binding; GO:0008022 protein C-terminus binding; GO:0019905 syntaxin binding; GO:0030234 enzyme regulator activity;	PF00595	PDZ domain (Also known as DHR or GLGF)	S	KOG3528	FOG: PDZ domain
O15446	DNA-directed RNA polymerase I subunit RPA34 OS=Homo sapiens OX=9606 GN=CD3EAP PE=1 SV=1	CD3EAP	0.7	0.007959651	Down				GO:0006139 nucleobase-containing compound metabolic process; GO:0006351 transcription, DNA-templated; GO:0006352 DNA-templated transcription, initiation; GO:0006353 DNA-templated transcription, termination; GO:0006360 transcription from RNA polymerase	GO:0000120 RNA polymerase I transcription factor complex; GO:0000428 DNA-directed RNA polymerase complex; GO:0001650 fibrillar center; GO:0005622 intracellular; GO:0005623 cell; GO:0005634 nucleus; GO:0005654 nucleoplasm; GO:0005667 transcriptio						
Q14123	Calcium/calmodulin-dependent 3',5'-cyclic nucleotide phosphodiesterase 1C OS=Homo sapiens OX=9606 GN=PDE1C PE=1 SV=1	PDE1C	0.697	0.041775924	Down	K13755	PDE1; calcium/calmodulin-dependent 3',5'-cyclic nucleotide phosphodiesterase [EC:3.1.4.17]	map00230 Purine metabolism; map01100 Metabolic pathways; map04020 Calcium signaling pathway; map04740 Olfactory transduction; map04742 Taste transduction; map04924 Renin secretion; map05032 Morphine addiction	GO:0002791 regulation of peptide secretion; GO:0002792 negative regulation of peptide secretion; GO:0003008 system process; GO:0006139 nucleobase-containing compound metabolic process; GO:0006163 purine nucleotide metabolic process; GO:0006195 purine nucleotide catabolic process; GO:00061	GO:0005623 cell; GO:0005929 cilium; GO:0036477 somatodendritic compartment; GO:0042995 cell projection; GO:0043025 neuronal cell body; GO:0043226 organelle; GO:0044297 cell body; GO:0044464 cell part; GO:0097458 neuron part;	GO:0000166 nucleotide binding; GO:0003824 catalytic activity; GO:0004112 cyclic-nucleotide phosphodiesterase activity; GO:0004114 3',5'-cyclic-nucleotide phosphodiesterase activity; GO:0004115 3',5'-cyclic-AMP phosphodiesterase activity; GO:0004117 calmodulin-dependent	PF00233	3'5'-cyclic nucleotide phosphodiesterase	T	KOG3688	Cyclic GMP phosphodiesterase
Q9NWM0	Spermine oxidase OS=Homo sapiens OX=9606 GN=SMOX PE=1 SV=1	SMOX	0.696	8.28568E-07	Down	K12259	SMOX, PAO5; spermine oxidase [EC:1.5.3.16 1.5.3.-]	map00330 Arginine and proline metabolism; map00410 beta-Alanine metabolism; map01100 Metabolic pathways	GO:0006576 cellular biogenic amine metabolic process; GO:0006595 polyamine metabolic process; GO:0006596 polyamine biosynthetic process; GO:0006598 polyamine catabolic process; GO:0006807 nitrogen compound metabolic process; GO:0008152 metabolic process; GO:00082	GO:0005622 intracellular; GO:0005623 cell; GO:0005634 nucleus; GO:0005635 nuclear envelope; GO:0005654 nucleoplasm; GO:0005737 cytoplasm; GO:0005829 cytosol; GO:0012505 endomembrane system; GO:0016020 membrane; GO:0031090 organelle	GO:0003824 catalytic activity; GO:0016491 oxidoreductase activity; GO:0016645 oxidoreductase activity, acting on the CH-NH group of donors; GO:0016647 oxidoreductase activity, acting on the CH-NH group of donors, oxygen as acceptor; GO:0046592 polyamine	PF01593	Flavin containing amine oxidoreductase	H	KOG0685	Flavin-containing amine oxidase

Q96G28	Cilia- and flagella-associated protein 36 OS=Homo sapiens OX=9606 GN=CFAP36 PE=1 SV=2	CFAP36	0.696	0.004288074	Down					GO:0005623 cell; GO:0005929 cilium; GO:0035869 ciliary transition zone; GO:0042995 cell projection; GO:0043226 organelle; GO:0044422 organelle part; GO:0044441 ciliary part; GO:0044463 cell projection part; GO:0044464 cell part; GO:0097546 ciliary base;	GO:0005488 binding; GO:0005515 protein binding; GO:0047485 protein N-terminus binding;			S	KOG4511	Uncharacterized conserved protein
Q9NVX0	HAUS augmin-like complex subunit 2 OS=Homo sapiens OX=9606 GN=HAUS2 PE=1 SV=1	HAUS2	0.693	0.040761218	Down	K16585	HAUS2; HAUS augmin-like complex subunit 2		GO:0000086 G2/M transition of mitotic cell cycle; GO:0000226 microtubule cytoskeleton organization; GO:0000278 mitotic cell cycle; GO:0006996 organelle organization; GO:0007010 cytoskeleton organization; GO:0007017 microtubule-based	GO:0005622 intracellular; GO:0005623 cell; GO:0005737 cytoplasm; GO:0005813 centrosome; GO:0005815 microtubule organizing center; GO:0005819 spindle; GO:0005829 cytosol; GO:0005856 cytoskeleton; GO:0005874 microtubule;						
Q2TAM9	Tumor suppressor candidate gene 1 protein OS=Homo sapiens OX=9606 GN=TUSC1 PE=1 SV=3	TUSC1	0.691	0.036999848	Down											
Q8WUA7	TBC1 domain family member 22A OS=Homo sapiens OX=9606 GN=TBC1D22A PE=1 SV=2	TBC1D22A	0.688	0.044577886	Down	K20360	TBC1D22, GYP1; TBC1 domain family member 2		GO:0006810 transport; GO:0006886 intracellular protein transport; GO:0008104 protein localization; GO:0015031 protein transport; GO:0015833 peptide transport; GO:0031338 regulation of vesicle fusion; GO:0032879 regulation of localization; GO:0033036	GO:0005622 intracellular; GO:0005623 cell; GO:0012505 endomembrane system; GO:0044464 cell part;	GO:0005096 GTPase activator activity; GO:0005488 binding; GO:0005515 protein binding; GO:0008047 enzyme activator activity; GO:0017016 Ras GTPase binding; GO:0017137 Rab GTPase binding; GO:0019899 enzyme binding; GO:0030234 enzyme regulator activity; GO:0030695 GTPase regulator			U	KOG1092	Ypt/Rab-specific GTPase-activating protein GYP1
P32455	Guanylate-binding protein 1 OS=Homo sapiens OX=9606 GN=GBP1 PE=1 SV=2	GBP1	0.688	0.001469536	Down	K20899	GBP1_3_4_7; guanylate-binding protein 1/3/4/7	map04621 NOD-like receptor signaling pathway	GO:0001817 regulation of cytokine production; GO:0001818 negative regulation of cytokine production; GO:0001932 regulation of protein phosphorylation; GO:0001933 negative regulation of protein phosphorylation; GO:0002376 immune system process; GO:0002682 regulation of immune system	GO:0005576 extracellular region; GO:0005622 intracellular; GO:0005623 cell; GO:0005634 nucleus; GO:0005654 nucleoplasm; GO:0005737 cytoplasm; GO:0005794 Golgi apparatus; GO:0005829 cytosol; GO:0005856 cytoskeleton; GO:0012505 endomemb	GO:0000166 nucleotide binding; GO:0001882 nucleoside binding; GO:0001883 purine nucleoside binding; GO:0003779 actin binding; GO:0003824 catalytic activity; GO:0003924 GTPase activity; GO:0005488 binding; GO:0005515 protein binding; GO:0005525 GTP binding; GO:0008092	PF02263; PF02841	Guanylate-binding protein, N-terminal domain; Guanylate-binding protein, C-terminal domain	S	KOG2037	Guanylate-binding protein
O43520	Phospholipid-transporting ATPase IC OS=Homo sapiens OX=9606 GN=ATP8B1 PE=1 SV=3	ATP8B1	0.685	0.025882539	Down	K01530	E7.6.2.1; phospholipid-translocating ATPase [EC:7.6.2.1]		GO:0003008 system process; GO:0006082 organic acid metabolic process; GO:0006355 regulation of transcription, DNA-templated; GO:0006629 lipid metabolic process; GO:0006810 transport; GO:0006811 ion transport; GO:0006820 anion transport; GO:0006855 drug transmemb	GO:0005622 intracellular; GO:0005623 cell; GO:0005737 cytoplasm; GO:0005783 endoplasmic reticulum; GO:0005794 Golgi apparatus; GO:0005886 plasma membrane; GO:0005887 integral component of plasma membrane; GO:0005903 brush border; GO:0012505 endomemb	GO:0003824 catalytic activity; GO:0004012 phospholipid-translocating ATPase activity; GO:0005215 transporter activity; GO:0005319 lipid transporter activity; GO:0005488 binding; GO:0005543 phospholipid binding; GO:0005548 phospholipid transporter activity; GO:00082			P	KOG0206	P-type ATPase

P23588	Eukaryotic translation initiation factor 4B OS=Homo sapiens OX=9606 GN=EIF4B PE=1 SV=2	EIF4B	0.684	0.041864169	Down	K03258	EIF4B; translation initiation factor 4B	map03013 RNA transport; map04150 mTOR signaling pathway; map04151 PI3K-Akt signaling pathway; map05205 Proteoglycans in cancer	GO:0001731 formation of translation preinitiation complex; GO:0002181 cytoplasmic translation; GO:0002183 cytoplasmic translational initiation; GO:0006412 translation; GO:0006413 translational initiation; GO:0006417 regulation of translation; GO:00064	GO:0005622 intracellular; GO:0005623 cell; GO:0005737 cytoplasm; GO:0005829 cytosol; GO:0005844 polysome; GO:0016281 eukaryotic translation initiation factor 4F complex; GO:0030425 dendrite; GO:0032991 macromolecular complex; GO:0036477	GO:0003676 nucleic acid binding; GO:0003723 RNA binding; GO:0003725 double-stranded RNA binding; GO:0003727 single-stranded RNA binding; GO:0003743 translation initiation factor activity; GO:0003824 catalytic activity; GO:0004386 helicase activity; GO:0005488 binding;	PF00076	RNA recognition motif. (a.k.a. RRM, RBD, or RNP domain)	A	KOG0118	FOG: RRM domain
P36941	Tumor necrosis factor receptor superfamily member 3 OS=Homo sapiens OX=9606 GN=LTBR PE=1 SV=1	LTBR	0.683	0.023242707	Down	K03159	TNFRSF3, LTBR; lymphotoxin beta receptor TNFR superfamily member 3	map04060 Cytokine-cytokine receptor interaction; map04061 Viral protein interaction with cytokine and cytokine receptor; map04064 NF-kappa B signaling pathway; map04066 HIF-1 signaling pathway; map04672 Intestinal immune network for IgA production; map05166 Human T-cell leukemia virus 1 infection; map05203 Viral carcinogenesis	GO:0001773 myeloid dendritic cell activation; GO:0001775 cell activation; GO:0001932 regulation of protein phosphorylation; GO:0001934 positive regulation of protein phosphorylation; GO:0002237 response to molecule of bacterial origin; GO:0002274 myeloid leukocyte activation; GO:00023	GO:0005622 intracellular; GO:0005623 cell; GO:0005737 cytoplasm; GO:0005794 Golgi apparatus; GO:0005886 plasma membrane; GO:0005887 integral component of plasma membrane; GO:0012505 endomembrane system; GO:0016020 membrane; GO:0016021 integral component	GO:0004888 transmembrane signaling receptor activity; GO:0005031 tumor necrosis factor-activated receptor activity; GO:0005035 death receptor activity; GO:0005488 binding; GO:0005515 protein binding; GO:0019899 enzyme binding; GO:0031625 ubiquitin protein ligase	PF00020	TNFR/NGFR cysteine-rich region			
Q7Z6B0	Coiled-coil domain-containing protein 91 OS=Homo sapiens OX=9606 GN=CCDC91 PE=1 SV=2	CCDC91	0.683	0.048978613	Down				GO:0006810 transport; GO:0006892 post-Golgi vesicle-mediated transport; GO:0006896 Golgi to vacuole transport; GO:0007034 vacuolar transport; GO:0007041 lysosomal transport; GO:0016192 vesicle-mediated transport; GO:0016482 cytosolic transport; GO:0046907	GO:0005622 intracellular; GO:0005623 cell; GO:0005634 nucleus; GO:0005654 nucleoplasm; GO:0005737 cytoplasm; GO:0005794 Golgi apparatus; GO:0005802 trans-Golgi network; GO:0012505 endomembrane system; GO:0031974 membrane-enclosed	GO:0005488 binding; GO:0005515 protein binding; GO:0042802 identical protein binding;					
Q8IVT2	Mitotic interactor and substrate of PLK1 OS=Homo sapiens OX=9606 GN=MISP PE=1 SV=1	MISP	0.68	0.012564204	Down					GO:0005622 intracellular; GO:0005623 cell; GO:0005737 cytoplasm; GO:0005856 cytoskeleton; GO:0005886 plasma membrane; GO:0005912 adherens junction; GO:0005924 cell-substrate adherens junction; GO:0005925 focal adhesion; GO:0005938 cell cortex;						
Q7Z494	Nephrocystin-3 OS=Homo sapiens OX=9606 GN=NPHP3 PE=1 SV=1	NPHP3	0.679	0.031034406	Down	K19360	NPHP3; nephrocystin-3		GO:0001655 urogenital system development; GO:0001822 kidney development; GO:0001889 liver development; GO:0001894 tissue homeostasis; GO:0001895 retina homeostasis; GO:0001947 heart looping; GO:0002009 morphogenesis of an epithelium; GO:00030	GO:0005622 intracellular; GO:0005623 cell; GO:0005737 cytoplasm; GO:0005829 cytosol; GO:0005929 cilium; GO:0042995 cell projection; GO:0043226 organelle; GO:0044422 organelle part; GO:0044424 intracellular part; GO:0044441 ciliary part; GO:00444				Z	KOG1840	Kinesin light chain

Q9UBC1	NF-kappa-B inhibitor-like protein 1 OS=Homo sapiens OX=9606 GN=NFKBIL1 PE=1 SV=1	NFKBIL1	0.677	0.049114984	Down	K09256	NFKBIL1; NF-kappa-B inhibitor-like protein 1		GO:0001817 regulation of cytokine production; GO:0001818 negative regulation of cytokine production; GO:0002237 response to molecule of bacterial origin; GO:0002682 regulation of immune system process; GO:0002683 negative regulation of immune system process; GO:0002831 regulation	GO:0005622 intracellular; GO:0005623 cell; GO:0005634 nucleus; GO:0005737 cytoplasm; GO:0005829 cytosol; GO:0043226 organelle; GO:0043227 membrane-bounded organelle; GO:0043229 intracellular organelle; GO:0043231 intracellular membrane-bounded						
O75807	Protein phosphatase 1 regulatory subunit 15A OS=Homo sapiens OX=9606 GN=PPP1R15A PE=1 SV=1	PPP1R15A	0.674	0.005417896	Down	K14019	PPP1R15A, GADD34; protein phosphatase 1 regulatory subunit 15A	map04141 Protein processing in endoplasmic reticulum	GO:0000122 negative regulation of transcription from RNA polymerase II promoter; GO:0001932 regulation of protein phosphorylation; GO:0001933 negative regulation of protein phosphorylation; GO:0001934 positive regulation of protein phosphorylation; GO:0006355 regulation	GO:0000164 protein phosphatase type 1 complex; GO:0005622 intracellular; GO:0005623 cell; GO:0005737 cytoplasm; GO:0005739 mitochondrion; GO:0005783 endoplasmic reticulum; GO:0005789 endoplasmic reticulum membrane; GO:0005794 Golgi apparatus;	GO:0005488 binding; GO:0005515 protein binding; GO:0008047 enzyme activator activity; GO:0008157 protein phosphatase 1 binding; GO:0019208 phosphatase regulator activity; GO:0019211 phosphatase activator activity; GO:0019888 protein phosphatase regulator activity; GO:0019899 enzyme	PF10488	Phosphatase-1 catalytic subunit binding region			
Q9NR12	PDZ and LIM domain protein 7 OS=Homo sapiens OX=9606 GN=PDLIM7 PE=1 SV=1	PDLIM7	0.674	0.003113653	Down	K19867	LDB3; LIM domain-binding protein 3		GO:0000902 cell morphogenesis; GO:0000904 cell morphogenesis involved in differentiation; GO:0006810 transport; GO:0006897 endocytosis; GO:0006898 receptor-mediated endocytosis; GO:0006928 movement of cell or subcellular component;	GO:0001725 stress fiber; GO:0001726 ruffle; GO:0005622 intracellular; GO:0005623 cell; GO:0005634 nucleus; GO:0005737 cytoplasm; GO:0005829 cytosol; GO:0005856 cytoskeleton; GO:0005911 cell-cell junction; GO:0005912 adherens junction; GO:0005913 cell-cell		PF00595; PF00412	PDZ domain (Also known as DHR or GLGF); LIM domain	TZ	KOG1703	Adaptor protein Enigma and related PDZ-LIM proteins
Q7Z422	SUZ domain-containing protein 1 OS=Homo sapiens OX=9606 GN=SZRD1 PE=1 SV=1	SZRD1	0.674	0.010373877	Down							PF12752; PF12901	SUZ domain; SUZ-C motif			
Q8NBF2	NHL repeat-containing protein 2 OS=Homo sapiens OX=9606 GN=NHLRC2 PE=1 SV=1	NHLRC2	0.672	0.033678083	Down				GO:0002576 platelet degranulation; GO:0006810 transport; GO:0006887 exocytosis; GO:0009987 cellular process; GO:0016192 vesicle-mediated transport; GO:0032940 secretion by cell; GO:0045055 regulated exocytosis; GO:0046903 secretion; GO:0051179 localization	GO:0005576 extracellular region; GO:0005622 intracellular; GO:0005623 cell; GO:0005737 cytoplasm; GO:0012505 endomembrane system; GO:0030141 secretory granule; GO:0031091 platelet alpha granule; GO:0031093 platelet alpha granule lumen;		PF13905	Thioredoxin-like	O	KOG2177	Predicted E3 ubiquitin ligase
P15151	Poliovirus receptor OS=Homo sapiens OX=9606 GN=PVR PE=1 SV=2	PVR	0.671	0.010964902	Down	K06539	PVR, NECL5, CD155; poliovirus receptor	map04514 Cell adhesion molecules (CAMs)	GO:0001910 regulation of leukocyte mediated cytotoxicity; GO:0001912 positive regulation of leukocyte mediated cytotoxicity; GO:0001914 regulation of T cell mediated cytotoxicity; GO:0001916 positive regulation of T cell mediated cytotoxicity; GO:00026	GO:0005576 extracellular region; GO:0005615 extracellular space; GO:0005622 intracellular; GO:0005623 cell; GO:0005737 cytoplasm; GO:0005886 plasma membrane; GO:0005887 integral component of plasma membrane; GO:0005911 cell-cell junction; GO:0005912 adherens	GO:0005102 receptor binding; GO:0005488 binding; GO:0005515 protein binding; GO:0038023 signaling receptor activity; GO:0042802 identical protein binding; GO:0042803 protein homodimerization activity; GO:0046983 protein dimerization activity; GO:0050839 cell adhesion molecule	PF07686; PF08205	Immunoglobulin V-set domain; CD80-like C2-set immunoglobulin domain			

Q14517	Protocadherin Fat 1 OS=Homo sapiens OX=9606 GN=FAT1 PE=1 SV=2	FAT1	0.669	0.049181498	Down	K16506	FAT1_2_3; protocadherin Fat 1/2/3		GO:0006928 movement of cell or subcellular component; GO:0006996 organelle organization; GO:0007010 cytoskeleton organization; GO:0007015 actin filament organization; GO:0007154 cell communication; GO:0007155 cell adhesion; GO:00071	GO:0005622 intracellular; GO:0005623 cell; GO:0005634 nucleus; GO:0005737 cytoplasm; GO:0005886 plasma membrane; GO:0005887 integral component of plasma membrane; GO:0005911 cell-cell junction; GO:0016020 membrane; GO:0016021 integral component of membrane; GO:00300		PF00028; PF02210; PF00008	Cadherin domain; Laminin G domain; EGF-like domain	T	KOG3594	FOG: Cadherin repeats
Q9UMX1	Suppressor of fused homolog OS=Homo sapiens OX=9606 GN=SUFU PE=1 SV=2	SUFU	0.665	0.013157854	Down	K06229	SUFU; suppressor of fused	map04340 Hedgehog signaling pathway; map04341 Hedgehog signaling pathway - fly; map05200 Pathways in cancer; map05217 Basal cell carcinoma	GO:0000122 negative regulation of transcription from RNA polymerase II promoter; GO:0001501 skeletal system development; GO:0006355 regulation of transcription, DNA-templated; GO:0006357 regulation of transcription from RNA polymerase II	GO:0005622 intracellular; GO:0005623 cell; GO:0005634 nucleus; GO:0005737 cytoplasm; GO:0005829 cytosol; GO:0005929 cilium; GO:0042995 cell projection; GO:0043226 organelle; GO:0043227 membrane-bounded organelle; GO:0043229 intracellular organelle; GO:00432	GO:0003712 transcription cofactor activity; GO:0003714 transcription corepressor activity; GO:0005488 binding; GO:0005515 protein binding; GO:0008134 transcription factor binding; GO:0019899 enzyme binding; GO:0019900 kinase binding; GO:0019901 protein kinase binding;					
P18621	60S ribosomal protein L17 OS=Homo sapiens OX=9606 GN=RPL17 PE=1 SV=3	RPL17	0.664	0.038174096	Down	K02880	RP-L17e, RPL17; large subunit ribosomal protein L17e	map03010 Ribosome	GO:0000184 nuclear-transcribed mRNA catabolic process, nonsense-mediated decay; GO:0000956 nuclear-transcribed mRNA catabolic process; GO:0006139 nucleobase-containing compound metabolic process; GO:0006401 RNA catabolic process; GO:0006402 mRNA catabolic process; GO:00064	GO:0005622 intracellular; GO:0005623 cell; GO:0005737 cytoplasm; GO:0005829 cytosol; GO:0044424 intracellular part; GO:0044444 cytoplasmic part; GO:0044464 cell part;	GO:0003735 structural constituent of ribosome; GO:0005198 structural molecule activity;	PF00237	Ribosomal protein L22p/L17e	J	KOG3353	60S ribosomal protein L22
O43741	5'-AMP-activated protein kinase subunit beta-2 OS=Homo sapiens OX=9606 GN=PRKAB2 PE=1 SV=1	PRKAB2	0.663	0.002292018	Down	K07199	PRKAB; 5'-AMP-activated protein kinase, regulatory beta subunit	map04068 FoxO signaling pathway; map04152 AMPK signaling pathway; map04211 Longevity regulating pathway; map04213 Longevity regulating pathway - multiple species; map04371 Apelin signaling pathway; map04530 Tight junction; map04710 Circadian rhythm; map04714 Thermogenesis; map04910 Insulin signaling pathway; map04920 Adipocytokine signaling pathway; map04921 Oxytocin signaling pathway; map04922 Glucagon signaling pathway; map04931 Insulin resistance; map04932 Non-alcoholic fatty liver disease (NAFLD); map05410 Hypertrophic cardiomyopathy (HCM)	GO:0006464 cellular protein modification process; GO:0006468 protein phosphorylation; GO:0006793 phosphorus metabolic process; GO:0006796 phosphate-containing compound metabolic process; GO:0006807 nitrogen compound metabolic process; GO:0006914 autophagy; GO:0007049 cell	GO:0005622 intracellular; GO:0005623 cell; GO:0005634 nucleus; GO:0005654 nucleoplasm; GO:0005737 cytoplasm; GO:0005829 cytosol; GO:0005886 plasma membrane; GO:0005952 cAMP-dependent protein kinase complex; GO:0016020 membrane; GO:0016324 apical	GO:0003824 catalytic activity; GO:0004672 protein kinase activity; GO:0004674 protein serine/threonine kinase activity; GO:0004679 AMP-activated protein kinase activity; GO:0005488 binding; GO:0005515 protein binding; GO:0016301 kinase activity; GO:0016740 transferase activity;			G	KOG1616	Protein involved in Snf1 protein kinase complex assembly
P00750	Tissue-type plasminogen activator OS=Homo sapiens OX=9606 GN=PLAT PE=1 SV=1	PLAT	0.659	0.001627749	Down	K01343	PLAT; tissue plasminogen activator [EC:3.4.21.68]	map04371 Apelin signaling pathway; map04610 Complement and coagulation cascades; map05202 Transcriptional misregulation in cancer; map05215 Prostate cancer; map05418 Fluid shear stress and atherosclerosis	GO:0001101 response to acid chemical; GO:0001666 response to hypoxia; GO:0006464 cellular protein modification process; GO:0006508 proteolysis; GO:0006807 nitrogen compound metabolic process; GO:0006928 movement of cell or subcellular component; GO:0006950	GO:0005576 extracellular region; GO:0005615 extracellular space; GO:0005622 intracellular; GO:0005623 cell; GO:0005737 cytoplasm; GO:0009986 cell surface; GO:0012505 endomembrane system; GO:0030141 secretory granule; GO:0031410	GO:0003824 catalytic activity; GO:0004175 endopeptidase activity; GO:0004252 serine-type endopeptidase activity; GO:0005102 receptor binding; GO:0005488 binding; GO:0005515 protein binding; GO:0008233 peptidase activity; GO:0008236 serine-type peptidase activity;	PF00039; PF00008; PF00051; PF00089	Fibronectin type I domain; EGF-like domain; Kringle domain; Trypsin	O	KOG3627	Trypsin

P11831	Serum response factor OS=Homo sapiens OX=9606 GN=SRF PE=1 SV=1	SRF	0.659	0.019815158	Down	K04378	SRF; serum response factor	map04010 MAPK signaling pathway; map04022 cGMP-PKG signaling pathway; map05166 Human T-cell leukemia virus 1 infection; map05203 Viral carcinogenesis	GO:0000122 negative regulation of transcription from RNA polymerase II promoter;	GO:0000228 nuclear chromosome; GO:0000785 chromatin; GO:0000790 nuclear chromatin;	GO:0000976 transcription regulatory region sequence-specific DNA binding;	PF00319	SRF-type transcription factor (DNA-binding and dimerisation domain)	K	KOG0015	Regulator of arginine metabolism and related MADS box-containing transcription factors
Q00973	Beta-1,4 N-acetylgalactosaminyltransferase 1 OS=Homo sapiens OX=9606 GN=B4GALNT1 PE=1 SV=2	B4GALNT1	0.658	0.038302584	Down	K00725	B4GALNT1, GALGT; (N-Acetylneuraminyl)-galactosylglucosylceramide N-acetylgalactosaminyltransferase [EC:2.4.1.92]	map00604 Glycosphingolipid biosynthesis - ganglio series; map01100 Metabolic pathways	GO:0000003 reproduction; GO:0001573 ganglioside metabolic process; GO:0001574 ganglioside biosynthetic process; GO:0005975 carbohydrate metabolic process; GO:0006629 lipid metabolic process; GO:0006643 membrane lipid metabolic process; GO:00066	GO:0000139 Golgi membrane; GO:0005622 intracellular; GO:0005623 cell; GO:0005737 cytoplasm; GO:0005794 Golgi apparatus; GO:0005886 plasma membrane; GO:0012505 endomembrane system; GO:0016020 membrane; GO:0031090 organelle membrane; GO:00319	GO:0003824 catalytic activity; GO:0003947 (N-acetylneuraminyl)-galactosylglucosylceramide N-acetylgalactosaminyltransferase activity; GO:0008194 UDP-glycosyltransferase activity; GO:0008376 acetylgalactosaminyltransferase activity; GO:0016740 transferase activity; GO:0016757					
Q86UB9	Transmembrane protein 135 OS=Homo sapiens OX=9606 GN=TMEM135 PE=2 SV=2	TMEM135	0.654	0.016317835	Down				GO:0006950 response to stress; GO:0006996 organelle organization; GO:0007031 peroxisome organization; GO:0007275 multicellular organism development; GO:0007568 aging; GO:0008340 determination of adult lifespan; GO:0009266 response to	GO:0005622 intracellular; GO:0005623 cell; GO:0005737 cytoplasm; GO:0005739 mitochondrion; GO:0005777 peroxisome; GO:0005811 lipid particle; GO:0042579 microbody; GO:0043226 organelle; GO:0043227 membrane-bounded organelle;				S	KOG1398	Uncharacterized conserved protein
P0CG31	Putative zinc finger protein 286B OS=Homo sapiens OX=9606 GN=ZNF286B PE=5 SV=1	ZNF286B	0.653	0.005364156	Down	K09228	KRAB; KRAB domain-containing zinc finger protein	map05168 Herpes simplex virus 1 infection	GO:0006355 regulation of transcription, DNA-templated; GO:0006357 regulation of transcription from RNA polymerase II promoter; GO:0009889 regulation of biosynthetic process; GO:0010468 regulation of gene expression; GO:0010556 regulation		GO:0000981 RNA polymerase II transcription factor activity, sequence-specific DNA binding; GO:0003700 transcription factor activity, sequence-specific DNA binding;	PF00096; PF13465	Zinc finger, C2H2 type; Zinc-finger double domain	K	KOG1721	FOG: Zn-finger
Q9BPW9	Dehydrogenase/reductase SDR family member 9 OS=Homo sapiens OX=9606 GN=DHRS9 PE=1 SV=1	DHRS9	0.65	0.011683454	Down	K11149	DHRS9; dehydrogenase/reductase SDR family member 9 [EC:1.1.-.-]	map00830 Retinol metabolism; map01100 Metabolic pathways	GO:0001523 retinoid metabolic process; GO:0002138 retinoic acid biosynthetic process; GO:0006066 alcohol metabolic process; GO:0006082 organic acid metabolic process; GO:0006629 lipid metabolic process; GO:0006720 isoprenoid metabolic process; GO:0006721 terpenoid metabolic	GO:0005622 intracellular; GO:0005623 cell; GO:0005737 cytoplasm; GO:0005783 endoplasmic reticulum; GO:0005789 endoplasmic reticulum membrane; GO:0012505 endomembrane system; GO:0016020 membrane; GO:0016021 integral component of	GO:0003824 catalytic activity; GO:0004022 alcohol dehydrogenase (NAD) activity; GO:0004745 retinol dehydrogenase activity; GO:0016229 steroid dehydrogenase activity; GO:0016491 oxidoreductase activity; GO:0016614 oxidoreductase activity, acting on CH-OH	PF00106	short chain dehydrogenase	Q	KOG1610	Corticosteroid 11-beta-dehydrogenase and related short chain-type dehydrogenases

Q15678	Tyrosine-protein phosphatase non-receptor type 14 OS=Homo sapiens OX=9606 GN=PTPN14 PE=1 SV=2	PTPN14	0.65	0.035432661	Down	K18025	PTPN14_21; tyrosine-protein phosphatase non-receptor type 14/21 [EC:3.1.3.48]		GO:0001944 vasculature development; GO:0001945 lymph vessel development; GO:0001946 lymphangiogenesis; GO:0006464 cellular protein modification process; GO:0006470 protein dephosphorylation; GO:0006793 phosphorus metabolic process; GO:0006796 phosphate-	GO:0005622 intracellular; GO:0005623 cell; GO:0005634 nucleus; GO:0005654 nucleoplasm; GO:0005737 cytoplasm; GO:0031974 membrane-enclosed lumen; GO:0031981 nuclear lumen; GO:0043226 organelle; GO:0043227 membrane-bounded organelle;	GO:0003712 transcription cofactor activity; GO:0003824 catalytic activity; GO:0004721 phosphoprotein phosphatase activity; GO:0004725 protein tyrosine phosphatase activity; GO:0005102 receptor binding; GO:0005488 binding; GO:0005515 protein binding; GO:0016787 hydrolase activity;	PF09379; PF00373; PF09380; PF00102	FERM N-terminal domain ; FERM central domain; FERM C-terminal PH-like domain; Protein-tyrosine phosphatase	T	KOG0792	Protein tyrosine phosphatase PTPMEG, contains FERM domain
Q13568	Interferon regulatory factor 5 OS=Homo sapiens OX=9606 GN=IRF5 PE=1 SV=2	IRF5	0.649	0.03887144	Down	K09446	IRF5; interferon regulatory factor 5	map04620 Toll-like receptor signaling pathway	GO:0001817 regulation of cytokine production; GO:0001819 positive regulation of cytokine production; GO:0002237 response to molecule of bacterial origin; GO:0002252 immune effector process; GO:0002376 immune system process; GO:0006139 nucleobase-containing compound metabolic process;	GO:0005622 intracellular; GO:0005623 cell; GO:0005634 nucleus; GO:0005737 cytoplasm; GO:0005829 cytosol; GO:0043226 organelle; GO:0043227 membrane-bounded organelle; GO:0043229 intracellular organelle; GO:0043231 intracellular membrane-bounded	GO:0000981 RNA polymerase II transcription factor activity, sequence-specific DNA binding; GO:0001228 transcriptional activator activity, RNA polymerase II transcription regulatory region sequence-specific binding; GO:0003676 nucleic acid binding;	PF00605	Interferon regulatory factor transcription factor			
Q96IZ0	PRKC apoptosis WT1 regulator protein OS=Homo sapiens OX=9606 GN=PAWR PE=1 SV=1	PAWR	0.643	0.024562722	Down				GO:0000122 negative regulation of transcription from RNA polymerase II promoter; GO:0001816 cytokine production; GO:0002237 response to molecule of bacterial origin; GO:0002682 regulation of immune system process; GO:0002683 negative regulation of immune system process;	GO:0000228 nuclear chromosome; GO:0000785 chromatin; GO:0000790 nuclear chromatin; GO:0005622 intracellular; GO:0005623 cell; GO:0005634 nucleus; GO:0005694 chromosome; GO:0005737 cytoplasm; GO:0005856 cytoskeleton; GO:0005884 actin	GO:0003712 transcription cofactor activity; GO:0003714 transcription corepressor activity; GO:0003779 actin binding; GO:0005080 protein kinase C binding; GO:0005488 binding; GO:0005515 protein binding; GO:0008092 cytoskeletal protein binding; GO:0008157 protein phosphatas					
Q7L945	Zinc finger protein 627 OS=Homo sapiens OX=9606 GN=ZNF627 PE=1 SV=1	ZNF627	0.64	0.024158429	Down	K09228	KRAB; KRAB domain-containing zinc finger protein	map05168 Herpes simplex virus 1 infection	GO:0006355 regulation of transcription, DNA-templated; GO:0006357 regulation of transcription from RNA polymerase II promoter; GO:0009889 regulation of biosynthetic process; GO:0010468 regulation of gene expression; GO:0010556 regulation		GO:0000981 RNA polymerase II transcription factor activity, sequence-specific DNA binding; GO:0003700 transcription factor activity, sequence-specific DNA binding;	PF00096	Zinc finger, C2H2 type	K	KOG1721	FOG: Zn-finger
Q16533	snRNA-activating protein complex subunit 1 OS=Homo sapiens OX=9606 GN=SNAPC1 PE=1 SV=1	SNAPC1	0.64	0.032193498	Down	K15208	SNAPC1; snRNA-activating protein complex subunit 1		GO:0006139 nucleobase-containing compound metabolic process; GO:0006351 transcription, DNA-templated; GO:0006366 transcription from RNA polymerase II promoter; GO:0006383 transcription from RNA polymerase III promoter; GO:0006725 cellular aromatic	GO:0005622 intracellular; GO:0005623 cell; GO:0005634 nucleus; GO:0005654 nucleoplasm; GO:0005667 transcription factor complex; GO:0005730 nucleolus; GO:0019185 snRNA-activating protein complex; GO:0031974 membrane-enclosed lumen; GO:00319	GO:0003676 nucleic acid binding; GO:0003677 DNA binding; GO:0005488 binding; GO:0043565 sequence-specific DNA binding; GO:0097159 organic cyclic compound binding; GO:1901363 heterocyclic compound binding;			K	KOG4746	Small nuclear RNA activating complex (SNAPc), subunit SNAP43

Q96RY7	Intraflagellar transport protein 140 homolog OS=Homo sapiens OX=9606 GN=IFT140 PE=1 SV=1	IFT140	0.639	0.041949058	Down	K19672	IFT140; intraflagellar transport protein 140		GO:0001501 skeletal system development; GO:0001654 eye development; GO:0001655 urogenital system development; GO:0003002 regionalization; GO:0006810 transport; GO:0006928 movement of cell or subcellular component; GO:0006996	GO:0001750 photoreceptor outer segment; GO:0005622 intracellular; GO:0005623 cell; GO:0005737 cytoplasm; GO:0005813 centrosome; GO:0005815 microtubule organizing center; GO:0005856 cytoskeleton; GO:0005929 cilium; GO:00059				S	KOG3617	WD40 and TPR repeat-containing protein
P10412	Histone H1.4 OS=Homo sapiens OX=9606 GN=H1-4 PE=1 SV=2	H1-4	0.636	0.02581203	Down	K11275	H1_5; histone H1/5		GO:0000122 negative regulation of transcription from RNA polymerase II promoter; GO:0006323 DNA packaging; GO:0006325 chromatin organization; GO:0006333 chromatin assembly or disassembly; GO:0006355 regulation of transcription, DNA-	GO:0000228 nuclear chromosome; GO:0000785 chromatin; GO:0000790 nuclear chromatin; GO:0000791 euchromatin; GO:0005622 intracellular; GO:0005623 cell; GO:0005634 nucleus; GO:0005694 chromosome; GO:0005719 nuclear euchromatin; GO:00319	GO:0003676 nucleic acid binding; GO:0003677 DNA binding; GO:0003682 chromatin binding; GO:0005488 binding; GO:0031490 chromatin DNA binding; GO:0097159 organic cyclic compound binding; GO:1901363 heterocyclic compound binding;	PF00538	linker histone H1 and H5 family	B	KOG4012	Histone H1
Q9Y672	Dolichyl pyrophosphate Man9GlcNAc2 alpha-1,3-glucosyltransferase OS=Homo sapiens OX=9606 GN=ALG6 PE=1 SV=2	ALG6	0.635	0.002741727	Down	K03848	ALG6; alpha-1,3-glucosyltransferase [EC:2.4.1.267]	map00510 N-Glycan biosynthesis; map01100 Metabolic pathways	GO:0006464 cellular protein modification process; GO:0006486 protein glycosylation; GO:0006487 protein N-linked glycosylation; GO:0006488 dolichol-linked oligosaccharide biosynthetic process; GO:0006490 oligosaccharide-lipid intermediate biosynthetic process; GO:00066	GO:0005622 intracellular; GO:0005623 cell; GO:0005737 cytoplasm; GO:0005783 endoplasmic reticulum; GO:0005789 endoplasmic reticulum membrane; GO:0012505 endomembrane system; GO:0016020 membrane; GO:0031984 organelle subcompart	GO:0003824 catalytic activity; GO:0004583 dolichyl-phosphate-glucose-glycolipid alpha-glucosyltransferase activity; GO:0016740 transferase activity; GO:0016757 transferase activity, transferring glycosyl groups; GO:0016758 transferase activity, transferring hexosyl groups;			G	KOG2575	Glucosyltransferase - Alg6p
Q2KHR2	DNA-binding protein RFX7 OS=Homo sapiens OX=9606 GN=RFX7 PE=1 SV=1	RFX7	0.633	0.004855891	Down				GO:0006355 regulation of transcription, DNA-templated; GO:0006357 regulation of transcription from RNA polymerase II promoter; GO:0009889 regulation of biosynthetic process; GO:0010468 regulation of gene expression; GO:0010556 regulation	GO:0005622 intracellular; GO:0005623 cell; GO:0005634 nucleus; GO:0043226 organelle; GO:0043227 membrane-bounded organelle; GO:0043229 intracellular organelle; GO:0043231 intracellular membrane-bounded organelle; GO:0044424 intracellular part;	GO:0000976 transcription regulatory region sequence-specific DNA binding; GO:0000977 RNA polymerase II regulatory region sequence-specific DNA binding; GO:0000978 RNA polymerase II core promoter proximal region sequence-specific DNA binding;	PF02257	RFX DNA-binding domain	K	KOG3712	RFX family transcription factor
Q92963	GTP-binding protein Rit1 OS=Homo sapiens OX=9606 GN=RIT1 PE=1 SV=1	RIT1	0.627	0.01288662	Down	K07832	RIT1; Ras-like without CAAX 1		GO:0007154 cell communication; GO:0007165 signal transduction; GO:0007264 small GTPase mediated signal transduction; GO:0007265 Ras protein signal transduction; GO:0009987 cellular process; GO:0023052 signaling; GO:0035556 intracellular signal	GO:0005623 cell; GO:0005886 plasma membrane; GO:0016020 membrane; GO:0044464 cell part; GO:0071944 cell periphery;	GO:0000166 nucleotide binding; GO:0001882 nucleoside binding; GO:0001883 purine nucleoside binding; GO:0003824 catalytic activity; GO:0003924 GTPase activity; GO:0005488 binding; GO:0005515 protein binding; GO:0005516 calmodulin binding; GO:0005525 GTP binding; GO:00164	PF00071	Ras family	S	KOG0395	Ras-related GTPase

O95182	NADH dehydrogenase [ubiquinone] 1 alpha subcomplex subunit 7 OS=Homo sapiens OX=9606 GN=NDUFA7 PE=1 SV=3	NDUFA7	0.621	0.043459947	Down	K03951	NDUFA7; NADH dehydrogenase (ubiquinone) 1 alpha subcomplex subunit 7	map00190 Oxidative phosphorylation; map01100 Metabolic pathways; map04714 Thermogenesis; map04723 Retrograde endocannabinoid signaling; map04932 Non-alcoholic fatty liver disease (NAFLD); map05010 Alzheimer disease; map05012 Parkinson disease; map05016 Huntington disease	GO:0006091 generation of precursor metabolites and energy; GO:0006119 oxidative phosphorylation; GO:0006120 mitochondrial electron transport, NADH to ubiquinone; GO:0006139 nucleobase-containing compound metabolic process; GO:0006163 purine nucleotide metabolic	GO:0000313 organellar ribosome; GO:0005622 intracellular; GO:0005623 cell; GO:0005737 cytoplasm; GO:0005739 mitochondrion; GO:0005740 mitochondrial envelope; GO:0005743 mitochondrial inner membrane; GO:0005746 mitochondrial	GO:0003735 structural constituent of ribosome; GO:0003824 catalytic activity; GO:0003954 NADH dehydrogenase activity; GO:0005198 structural molecule activity; GO:0008137 NADH dehydrogenase (ubiquinone) activity; GO:0016491 oxidoreductase activity; GO:00166			C	KOG4630	NADH:ubiquinone oxidoreductase, NDUFA7/B14.5A subunit
O15145	Actin-related protein 2/3 complex subunit 3 OS=Homo sapiens OX=9606 GN=ARPC3 PE=1 SV=3	ARPC3	0.621	0.016931656	Down	K05756	ARPC3; actin related protein 2/3 complex, subunit 3	map04144 Endocytosis; map04666 Fc gamma R-mediated phagocytosis; map04810 Regulation of actin cytoskeleton; map05100 Bacterial invasion of epithelial cells; map05130 Pathogenic Escherichia coli infection; map05131 Shigellosis; map05132 Salmonella infection	GO:0002252 immune effector process; GO:0002253 activation of immune response; GO:0002376 immune system process; GO:0002429 immune response-activating cell surface receptor signaling pathway; GO:0002431 Fc receptor mediated stimulatory signaling pathway; GO:0002433 immune response-	GO:0005622 intracellular; GO:0005623 cell; GO:0005737 cytoplasm; GO:0005829 cytosol; GO:0005856 cytoskeleton; GO:0005884 actin filament; GO:0005885 Arp2/3 protein complex; GO:0015629 actin cytoskeleton; GO:0030027 lamellipodium; GO:00304	GO:0005198 structural molecule activity; GO:0005200 structural constituent of cytoskeleton;			Z	KOG3155	Actin-related protein Arp2/3 complex, subunit ARPC3
Q86TS9	39S ribosomal protein L52, mitochondrial OS=Homo sapiens OX=9606 GN=MRPL52 PE=1 SV=2	MRPL52	0.621	0.03100891	Down	K17433	MRPL52; large subunit ribosomal protein L52		GO:0006412 translation; GO:0006414 translational elongation; GO:0006415 translational termination; GO:0006518 peptide metabolic process; GO:0006807 nitrogen compound metabolic process; GO:0008152 metabolic process; GO:0009058 biosynthetic process;	GO:0000313 organellar ribosome; GO:0000315 organellar large ribosomal subunit; GO:0005622 intracellular; GO:0005623 cell; GO:0005737 cytoplasm; GO:0005739 mitochondrion; GO:0005740 mitochondrial envelope; GO:0005743 mitochondr	GO:0003735 structural constituent of ribosome; GO:0005198 structural molecule activity;					
P18124	60S ribosomal protein L7 OS=Homo sapiens OX=9606 GN=RPL7 PE=1 SV=1	RPL7	0.614	0.036763313	Down	K02937	RP-L7e, RPL7; large subunit ribosomal protein L7e	map03010 Ribosome	GO:0000184 nuclear-transcribed mRNA catabolic process, nonsense-mediated decay; GO:0000463 maturation of LSU-rRNA from tricistronic rRNA transcript (SSU-rRNA, 5.8S rRNA, LSU-rRNA); GO:0000470 maturation of LSU-rRNA; GO:0000956 nuclear-transcribed	GO:0005622 intracellular; GO:0005623 cell; GO:0005737 cytoplasm; GO:0005829 cytosol; GO:0032991 macromolecular complex; GO:0044424 intracellular part; GO:0044444 cytoplasmic part; GO:0044464 cell part; GO:1990904 ribonucleoprotein	GO:0003676 nucleic acid binding; GO:0003677 DNA binding; GO:0003723 RNA binding; GO:0003729 mRNA binding; GO:0003735 structural constituent of ribosome; GO:0005198 structural molecule activity; GO:0005488 binding; GO:0005515 protein binding; GO:0042802	PF08079; PF00327	Ribosomal L30 N-terminal domain; Ribosomal protein L30p/L7e	J	KOG3184	60S ribosomal protein L7
Q96GA3	Protein LTV1 homolog OS=Homo sapiens OX=9606 GN=LTV1 PE=1 SV=1	LTV1	0.608	0.020118455	Down	K14798	LTV1; protein LTV1		GO:0000054 ribosomal subunit export from nucleus; GO:0000056 ribosomal small subunit export from nucleus; GO:0006403 RNA localization; GO:0006405 RNA export from nucleus; GO:0006611 protein export from nucleus; GO:0006810	GO:0005622 intracellular; GO:0005623 cell; GO:0005634 nucleus; GO:0005654 nucleoplasm; GO:0005737 cytoplasm; GO:0005768 endosome; GO:0005770 late endosome; GO:0005773 vacuole; GO:0005774 vacuolar membrane; GO:0005829 cytosol; GO:0010008				U	KOG2637	Uncharacterized conserved protein
Q96F05	Uncharacterized protein C11orf24 OS=Homo sapiens OX=9606 GN=C11orf24 PE=1 SV=2	C11orf24	0.597	0.042978778	Down											

P07305	Histone H1.0 OS=Homo sapiens OX=9606 GN=H1-0 PE=1 SV=3	H1-0	0.596	0.004074814	Down	K11275	H1_5; histone H1/5		GO:0000737 DNA catabolic process, endonucleolytic; GO:0006139 nucleobase-containing compound metabolic process; GO:0006259 DNA metabolic process; GO:0006308 DNA catabolic process; GO:0006309 apoptotic DNA fragmentation; GO:0006725 cellular aromatic compound	GO:0000228 nuclear chromosome; GO:0000785 chromatin; GO:0000790 nuclear chromatin; GO:0000791 euchromatin; GO:0005622 intracellular; GO:0005623 cell; GO:0005634 nucleus; GO:0005654 nucleoplasm; GO:0005694 chromosome; GO:00057	GO:0003676 nucleic acid binding; GO:0003677 DNA binding; GO:0003680 AT DNA binding; GO:0003682 chromatin binding; GO:0003690 double-stranded DNA binding; GO:0005488 binding; GO:0031490 chromatin DNA binding; GO:0043565 sequence-specific	PF00538	linker histone H1 and H5 family	B	KOG4012	Histone H1
P82909	28S ribosomal protein S36, mitochondrial OS=Homo sapiens OX=9606 GN=MRPS36 PE=1 SV=2	MRPS36	0.593	0.030298615	Down	K17414	MRPS36; small subunit ribosomal protein S36		GO:0006412 translation; GO:0006414 translational elongation; GO:0006415 translational termination; GO:0006518 peptide metabolic process; GO:0006807 nitrogen compound metabolic process; GO:0008152 metabolic process; GO:0009058 biosynthetic process;	GO:0000313 organellar ribosome; GO:0000314 organellar small ribosomal subunit; GO:0005622 intracellular; GO:0005623 cell; GO:0005737 cytoplasm; GO:0005739 mitochondrion; GO:0005740 mitochondrial envelope; GO:0005743 mitochondr	GO:0003735 structural constituent of ribosome; GO:0005198 structural molecule activity;					
Q9C0B7	Transport and Golgi organization protein 6 homolog OS=Homo sapiens OX=9606 GN=TANGO6 PE=1 SV=2	TANGO6	0.591	0.04525669	Down									S	KOG4653	Uncharacterized conserved protein
Q9UHA3	Probable ribosome biogenesis protein RLP24 OS=Homo sapiens OX=9606 GN=RSL24D1 PE=1 SV=1	RSL24D1	0.59	0.044205639	Down	K02896	RP-L24e, RPL24; large subunit ribosomal protein L24e	map03010 Ribosome	GO:0000027 ribosomal large subunit assembly; GO:0006412 translation; GO:0006518 peptide metabolic process; GO:0006807 nitrogen compound metabolic process; GO:0006996 organelle organization; GO:0008152 metabolic process; GO:0009058 biosynthetic process;	GO:0005622 intracellular; GO:0005623 cell; GO:0005634 nucleus; GO:0005730 nucleolus; GO:0005737 cytoplasm; GO:0005829 cytosol; GO:0005840 ribosome; GO:0015934 large ribosomal subunit; GO:0022625 cytosolic large ribosomal subunit; GO:0022626	GO:0003676 nucleic acid binding; GO:0003723 RNA binding; GO:0003735 structural constituent of ribosome; GO:0005198 structural molecule activity; GO:0005488 binding; GO:0097159 organic cyclic compound binding; GO:1901363 heterocyclic compound binding;	PF01246	Ribosomal protein L24e	J	KOG1723	60s ribosomal protein L30 isolog
Q00536	Cyclin-dependent kinase 16 OS=Homo sapiens OX=9606 GN=CDK16 PE=1 SV=1	CDK16	0.59	0.041735306	Down	K08820	CDK16, PCTK1; cyclin-dependent kinase 16 [EC:2.7.11.22]		GO:0000003 reproduction; GO:0002790 peptide secretion; GO:0002791 regulation of peptide secretion; GO:0006464 cellular protein modification process; GO:0006468 protein phosphorylation; GO:0006793 phosphorus metabolic process; GO:0006796 phosphate-containing compound	GO:0005622 intracellular; GO:0005623 cell; GO:0005737 cytoplasm; GO:0005829 cytosol; GO:0005856 cytoskeleton; GO:0005886 plasma membrane; GO:0009898 cytoplasmic side of plasma membrane; GO:0015630 microtubule cytoskeleton; GO:00160	GO:0003824 catalytic activity; GO:0004672 protein kinase activity; GO:0004674 protein serine/threonine kinase activity; GO:0016301 kinase activity; GO:0016740 transferase activity; GO:0016772 transferase activity, transferring phosphorus-containing groups; GO:0016773 phosphotra	PF00069	Protein kinase domain	T	KOG0594	Protein kinase PCTAIRE and related kinases
Q9BW04	Specifically androgen-regulated gene protein OS=Homo sapiens OX=9606 GN=SARG PE=1 SV=2	SARG	0.587	0.022516673	Down	K13719	OTU1, YOD1; ubiquitin thioesterase OTU1 [EC:3.1.2.-]	map04141 Protein processing in endoplasmic reticulum		GO:0005622 intracellular; GO:0005623 cell; GO:0005737 cytoplasm; GO:0005829 cytosol; GO:0005886 plasma membrane; GO:0016020 membrane; GO:0044424 intracellular part; GO:0044444 cytoplasmic part; GO:0044464 cell part; GO:0071944 cell periphery;						

P42696	RNA-binding protein 34 OS=Homo sapiens OX=9606 GN=RBM34 PE=1 SV=2	RBM34	0.587	0.045822556	Down	K14837	NOP12; nucleolar protein 12			GO:0005622 intracellular; GO:0005623 cell; GO:0005634 nucleus; GO:0005730 nucleolus; GO:0031974 membrane-enclosed lumen; GO:0031981 nuclear lumen; GO:0043226 organelle; GO:0043227 membrane-bounded organelle; GO:0043228 non-membrane-bounded		PF00076	RNA recognition motif. (a.k.a. RRM, RBD, or RNP domain)	A	KOG0118	FOG: RRM domain
O60861	Growth arrest-specific protein 7 OS=Homo sapiens OX=9606 GN=GAS7 PE=1 SV=3	GAS7	0.584	0.037590133	Down	K18618	GAS7; growth arrest-specific protein 7		GO:0000902 cell morphogenesis; GO:0006355 regulation of transcription, DNA-templated; GO:0006996 organelle organization; GO:0007010 cytoskeleton organization; GO:0007015 actin filament organization; GO:0007049 cell cycle; GO:00070	GO:0001726 ruffle; GO:0005622 intracellular; GO:0005623 cell; GO:0005737 cytoplasm; GO:0005856 cytoskeleton; GO:0005884 actin filament; GO:0015629 actin cytoskeleton; GO:0031252 cell leading edge; GO:0042995 cell projection; GO:0043226	GO:0003700 transcription factor activity, sequence-specific DNA binding; GO:0003779 actin binding; GO:0005488 binding; GO:0005515 protein binding; GO:0008092 cytoskeletal protein binding; GO:0044877 macromolecular complex binding; GO:0051015 actin filament	PF00397	WW domain	DO	KOG2398	Predicted proline-serine-threonine phosphatase-interacting protein (PSTPIP)
Q86SJ2	Amphoterin-induced protein 2 OS=Homo sapiens OX=9606 GN=AMIGO2 PE=1 SV=1	AMIGO2	0.584	0.010153372	Down	K22529	AMIGO; amphoterin-induced protein		GO:0007155 cell adhesion; GO:0007156 homophilic cell adhesion via plasma membrane adhesion molecules; GO:0007157 heterophilic cell-cell adhesion via plasma membrane cell adhesion molecules; GO:0010941 regulation of cell death; GO:0022610 biological adhesion;	GO:0005623 cell; GO:0005886 plasma membrane; GO:0005887 integral component of plasma membrane; GO:0016020 membrane; GO:0016021 integral component of membrane; GO:0031224 intrinsic component of membrane; GO:0031226 intrinsic component of plasma membrane; GO:0044425 membrane		PF00047	Immunoglobulin domain			
Q8TF74	WAS/WASL-interacting protein family member 2 OS=Homo sapiens OX=9606 GN=WIPF2 PE=1 SV=1	WIPF2	0.581	0.005189587	Down	K19475	WIPF; WAS/WASL-interacting protein	map04144 Endocytosis; map05130 Pathogenic Escherichia coli infection; map05135 Yersinia infection	GO:0002252 immune effector process; GO:0002253 activation of immune response; GO:0002376 immune system process; GO:0002429 immune response-activating cell surface receptor signaling pathway; GO:0002431 Fc receptor mediated stimulatory signaling pathway; GO:0002433 immune response-	GO:0005622 intracellular; GO:0005623 cell; GO:0005634 nucleus; GO:0005654 nucleoplasm; GO:0005737 cytoplasm; GO:0005829 cytosol; GO:0005886 plasma membrane; GO:0016020 membrane; GO:0031974 membrane-enclosed lumen; GO:0031981 nuclear lumen;				Z	KOG4462	WASP-interacting protein VRP1/WIP, contains WH2 domain
P10301	Ras-related protein R-Ras OS=Homo sapiens OX=9606 GN=RRAS PE=1 SV=1	RRAS	0.576	0.045375734	Down	K07829	RRAS; Ras-related protein R-Ras	map04010 MAPK signaling pathway; map04014 Ras signaling pathway; map04015 Rap1 signaling pathway; map04024 cAMP signaling pathway; map04072 Phospholipase D signaling pathway; map04137 Mitophagy - animal; map04140 Autophagy - animal; map04218 Cellular senescence; map04360 Axon guidance; map04371 Apelin signaling pathway; map04625 C-type lectin receptor signaling pathway; map04810 Regulation of actin cytoskeleton; map05205 Proteoglycans in cancer	GO:0001932 regulation of protein phosphorylation; GO:0002376 immune system process; GO:0002520 immune system development; GO:0002521 leukocyte differentiation; GO:0007154 cell communication; GO:0007165 signal transduction; GO:0007264 small GTPase	GO:0005622 intracellular; GO:0005623 cell; GO:0005737 cytoplasm; GO:0005829 cytosol; GO:0005886 plasma membrane; GO:0016020 membrane; GO:0044424 intracellular part; GO:0044444 cytoplasmic part; GO:0044464 cell part; GO:0071944 cell periphery;	GO:0000166 nucleotide binding; GO:0001882 nucleoside binding; GO:0001883 purine nucleoside binding; GO:0003824 catalytic activity; GO:0003924 GTPase activity; GO:0005488 binding; GO:0016462 pyrophosphatase activity; GO:0016787 hydrolase activity; GO:0016817	PF00071	Ras family	S	KOG0395	Ras-related GTPase

Q9GZP9	Derlin-2 OS=Homo sapiens OX=9606 GN=DERL2 PE=1 SV=1	DERL2	0.575	0.034860693	Down	K13989	DERL2_3; Derlin-2/3	map04141 Protein processing in endoplasmic reticulum	GO:0001558 regulation of cell growth; GO:0001967 suckling behavior; GO:0006464 cellular protein modification process; GO:0006508 proteolysis; GO:0006511 ubiquitin-dependent protein catabolic process; GO:0006517 protein deglycosylation; GO:0006807 nitrogen compound metabolic	GO:0005622 intracellular; GO:0005623 cell; GO:0005737 cytoplasm; GO:0005768 endosome; GO:0005769 early endosome; GO:0005770 late endosome; GO:0005783 endoplasmic reticulum; GO:0005789 endoplasmic reticulum membrane; GO:0012505 endomemb				S	KOG0858	Predicted membrane protein
P62837	Ubiquitin-conjugating enzyme E2 D2 OS=Homo sapiens OX=9606 GN=UBE2D2 PE=1 SV=1	UBE2D2	0.57	0.044237935	Down	K06689	UBE2D, UBC4, UBC5; ubiquitin-conjugating enzyme E2 D [EC:2.3.2.23]	map04013 MAPK signaling pathway - fly; map04120 Ubiquitin mediated proteolysis; map04141 Protein processing in endoplasmic reticulum; map04624 Toll and Imd signaling pathway; map05131 Shigellosis	GO:0000209 protein polyubiquitination; GO:0006464 cellular protein modification process; GO:0006807 nitrogen compound metabolic process; GO:0008152 metabolic process; GO:0009987 cellular process; GO:0016567 protein ubiquitination; GO:0019538 protein metabolic process; GO:0032446 protein	GO:0000151 ubiquitin ligase complex; GO:0005622 intracellular; GO:0005623 cell; GO:0032991 macromolecular complex; GO:0044424 intracellular part; GO:0044464 cell part; GO:1902494 catalytic complex; GO:1990234 transferase complex;	GO:0003824 catalytic activity; GO:0004842 ubiquitin-protein transferase activity; GO:0016740 transferase activity; GO:0019787 ubiquitin-like protein transferase activity; GO:0061631 ubiquitin conjugating enzyme activity; GO:0061650 ubiquitin-like protein conjugating enzyme	PF00179	Ubiquitin-conjugating enzyme	O	KOG0417	Ubiquitin-protein ligase
Q9Y3E1	Hepatoma-derived growth factor-related protein 3 OS=Homo sapiens OX=9606 GN=HDGFL3 PE=1 SV=1	HDGFL3	0.568	0.043870316	Down				GO:0000226 microtubule cytoskeleton organization; GO:0006996 organelle organization; GO:0007010 cytoskeleton organization; GO:0007017 microtubule-based process; GO:0007026 negative regulation of microtubule depolymeri	GO:0005622 intracellular; GO:0005623 cell; GO:0005634 nucleus; GO:0005654 nucleoplasm; GO:0005737 cytoplasm; GO:0005829 cytosol; GO:0031974 membrane-enclosed lumen; GO:0031981 nuclear lumen; GO:0043226 organelle; GO:0043227 membrane-	GO:0005488 binding; GO:0005515 protein binding; GO:0008017 microtubule binding; GO:0008092 cytoskeletal protein binding; GO:0015631 tubulin binding;	PF00855	PWWP domain	K	KOG1904	Transcription coactivator
P49918	Cyclin-dependent kinase inhibitor 1C OS=Homo sapiens OX=9606 GN=CDKN1C PE=1 SV=1	CDKN1C	0.566	0.03341927	Down	K09993	CDKN1C, P57, KIP2; cyclin-dependent kinase inhibitor 1C	map04110 Cell cycle	GO:0000003 reproduction; GO:0000122 negative regulation of transcription from RNA polymerase II promoter; GO:0001501 skeletal system development; GO:0001654 eye development; GO:0001655 urogenital system development; GO:0001701 in utero	GO:0005622 intracellular; GO:0005623 cell; GO:0005634 nucleus; GO:0005737 cytoplasm; GO:0043226 organelle; GO:0043227 membrane-bounded organelle; GO:0043229 intracellular organelle; GO:0043231 intracellular membrane-bounded organelle; GO:00444	GO:0004857 enzyme inhibitor activity; GO:0004860 protein kinase inhibitor activity; GO:0005488 binding; GO:0019207 kinase regulator activity; GO:0019210 kinase inhibitor activity; GO:0019887 protein kinase regulator activity; GO:0030234 enzyme regulator activity; GO:0044877 macromole			K	KOG4743	Cyclin-dependent kinase inhibitor
O60828	Polyglutamine-binding protein 1 OS=Homo sapiens OX=9606 GN=PQBP1 PE=1 SV=1	PQBP1	0.566	0.047404368	Down	K12865	PQBP1, NPW38; polyglutamine-binding protein 1	map03040 Spliceosome	GO:0000375 RNA splicing, via transesterification reactions; GO:0000377 RNA splicing, via transesterification reactions with bulged adenosine as nucleophile; GO:0000380 alternative mRNA splicing, via spliceosome; GO:0000398 mRNA splicing,	GO:0005622 intracellular; GO:0005623 cell; GO:0005634 nucleus; GO:0005654 nucleoplasm; GO:0005737 cytoplasm; GO:0005813 centrosome; GO:0005815 microtubule organizing center; GO:0005829 cytosol; GO:0005856 cytoskeleton;	GO:0003676 nucleic acid binding; GO:0003677 DNA binding; GO:0003690 double-stranded DNA binding; GO:0003712 transcription cofactor activity; GO:0003713 transcription coactivator activity; GO:0005488 binding; GO:0005515 protein binding; GO:0008022 protein C-terminus			K	KOG3427	Polyglutamine tract-binding protein PQBP-1

O60675	Transcription factor MafK OS=Homo sapiens OX=9606 GN=MAFK PE=1 SV=1	MAFK	0.565	0.025493662	Down	K09037	MAFF_G_K; transcription factor MAFF/G/K		GO:0000122 negative regulation of transcription from RNA polymerase II promoter; GO:0006355 regulation of transcription, DNA-templated; GO:0006357 regulation of transcription from RNA polymerase II promoter; GO:0006950 response to stress;	GO:0005622 intracellular; GO:0005623 cell; GO:0005634 nucleus; GO:0005654 nucleoplasm; GO:0031974 membrane-enclosed lumen; GO:0031981 nuclear lumen; GO:0043226 organelle; GO:0043227 membrane-bounded organelle; GO:0043229 intracellula	GO:0000976 transcription regulatory region sequence-specific DNA binding; GO:0000981 RNA polymerase II transcription factor activity, sequence-specific DNA binding; GO:0001067 regulatory region nucleic acid binding; GO:0001221 transcriptio			K	KOG4196	bZIP transcription factor MafK
P54687	Branched-chain-amino-acid aminotransferase, cytosolic OS=Homo sapiens OX=9606 GN=BCAT1 PE=1 SV=3	BCAT1	0.554	0.016875549	Down	K00826	E2.6.1.42, ilvE; branched-chain amino acid aminotransferase [EC:2.6.1.42]	map00270 Cysteine and methionine metabolism; map00280 Valine, leucine and isoleucine degradation; map00290 Valine, leucine and isoleucine biosynthesis; map00770 Pantothenate and CoA biosynthesis; map01100 Metabolic pathways; map01210 2-Oxocarboxylic acid metabolism; map01230 Biosynthesis of amino acids	GO:0000082 G1/S transition of mitotic cell cycle; GO:0000278 mitotic cell cycle; GO:0006082 organic acid metabolic process; GO:0006520 cellular amino acid metabolic process; GO:0006551 leucine metabolic process; GO:0006573 valine metabolic process; GO:0006807 nitrogen compound metabolic process;	GO:0005622 intracellular; GO:0005623 cell; GO:0005737 cytoplasm; GO:0005739 mitochondrion; GO:0005829 cytosol; GO:0043226 organelle; GO:0043227 membrane-bounded organelle; GO:0043229 intracellular organelle; GO:0043231 intracellular	GO:0003824 catalytic activity; GO:0004084 branched-chain-amino-acid transaminase activity; GO:0005488 binding; GO:0005515 protein binding; GO:0008483 transaminase activity; GO:0016740 transferase activity; GO:0016769 transferase activity, transferring nitrogenous groups; GO:00428	PF01063	Aminotransferase class IV	E	KOG0975	Branched chain aminotransferase BCAT1, pyridoxal phosphate enzymes type IV superfamily
P17302	Gap junction alpha-1 protein OS=Homo sapiens OX=9606 GN=GJA1 PE=1 SV=2	GJA1	0.553	0.024855564	Down	K07372	GJA1, CX43; gap junction alpha-1 protein	map04540 Gap junction; map05131 Shigellosis; map05412 Arrhythmogenic right ventricular cardiomyopathy (ARVC)	GO:0000003 reproduction; GO:0000902 cell morphogenesis; GO:0001101 response to acid chemical; GO:0001503 ossification; GO:0001508 action potential; GO:0001558 regulation of cell growth; GO:0001568 blood vessel development; GO:00016	GO:0000139 Golgi membrane; GO:0000323 lytic vacuole; GO:0005622 intracellular; GO:0005623 cell; GO:0005634 nucleus; GO:0005654 nucleoplasm; GO:0005737 cytoplasm; GO:0005739 mitochondrion; GO:0005740 mitochondrial envelope; GO:00057	GO:0005102 receptor binding; GO:0005215 transporter activity; GO:0005243 gap junction channel activity; GO:0005488 binding; GO:0005515 protein binding; GO:0008092 cytoskeletal protein binding; GO:0015075 ion transmembrane transporter activity; GO:0015267 channel activity;					
Q92859	Neogenin OS=Homo sapiens OX=9606 GN=NEO1 PE=1 SV=2	NEO1	0.552	0.017120403	Down	K06766	NEO1; neogenin	map04350 TGF-beta signaling pathway; map04360 Axon guidance; map04514 Cell adhesion molecules (CAMs)	GO:0000768 syncytium formation by plasma membrane fusion; GO:0001764 neuron migration; GO:0002791 regulation of peptide secretion; GO:0002792 negative regulation of peptide secretion; GO:0006355 regulation of transcription, DNA-templated; GO:0006357 regulation of	GO:0005622 intracellular; GO:0005623 cell; GO:0005634 nucleus; GO:0005654 nucleoplasm; GO:0005737 cytoplasm; GO:0005794 Golgi apparatus; GO:0005886 plasma membrane; GO:0005887 integral component of plasma membrane; GO:0009986 cell surface; GO:0012505	GO:0005102 receptor binding; GO:0005488 binding; GO:0005515 protein binding; GO:0033612 receptor serine/threonine kinase binding; GO:0038023 signaling receptor activity; GO:0039706 co-receptor binding; GO:0045296 cadherin binding; GO:0050839 cell adhesion molecule	PF13895; PF13927; PF07679; PF00041	Immunoglobulin domain; Immunoglobulin domain; Immunoglobulin I-set domain; Fibronectin type III domain	T	KOG4221	Receptor mediating netrin-dependent axon guidance
Q5VW00	DDB1- and CUL4-associated factor 12-like protein 2 OS=Homo sapiens OX=9606 GN=DCAF12L2 PE=2 SV=1	DCAF12L2	0.538	0.031959801	Down	K11803	DCAF12; DDB1- and CUL4-associated factor 12									

P16403	Histone H1.2 OS=Homo sapiens OX=9606 GN=H1-2 PE=1 SV=2	H1-2	0.533	0.011935636	Down	K11275	H1_5; histone H1/5		GO:0000122 negative regulation of transcription from RNA polymerase II promoter; GO:0006323 DNA packaging; GO:0006325 chromatin organization; GO:0006333 chromatin assembly or disassembly; GO:0006355 regulation of transcription, DNA-	GO:0000228 nuclear chromosome; GO:0000785 chromatin; GO:0000790 nuclear chromatin; GO:0000791 euchromatin; GO:0005622 intracellular; GO:0005623 cell; GO:0005634 nucleus; GO:0005694 chromosome; GO:0005719 nuclear euchromatin; GO:00319	GO:0003676 nucleic acid binding; GO:0003677 DNA binding; GO:0003682 chromatin binding; GO:0005488 binding; GO:0031490 chromatin DNA binding; GO:0097159 organic cyclic compound binding; GO:1901363 heterocyclic compound binding;	PF00538	linker histone H1 and H5 family	B	KOG4012	Histone H1
P47914	60S ribosomal protein L29 OS=Homo sapiens OX=9606 GN=RPL29 PE=1 SV=2	RPL29	0.531	0.040051706	Down	K02905	RP-L29e, RPL29; large subunit ribosomal protein L29e	map03010 Ribosome	GO:0000003 reproduction; GO:0000184 nuclear-transcribed mRNA catabolic process, nonsense-mediated decay; GO:0000956 nuclear-transcribed mRNA catabolic process; GO:0002181 cytoplasmic translation; GO:0006139 nucleobase-containing compound metabolic process;	GO:0005622 intracellular; GO:0005623 cell; GO:0005737 cytoplasm; GO:0005783 endoplasmic reticulum; GO:0005789 endoplasmic reticulum membrane; GO:0005791 rough endoplasmic reticulum; GO:0005829 cytosol; GO:0005840 ribosome; GO:0012505	GO:0003735 structural constituent of ribosome; GO:0005198 structural molecule activity; GO:0005488 binding; GO:0005539 glycosaminoglycan binding; GO:0008201 heparin binding; GO:0043167 ion binding; GO:0043168 anion binding; GO:0097367 carbohydrate			J	KOG3504	60S ribosomal protein L29
P62745	Rho-related GTP-binding protein RhoB OS=Homo sapiens OX=9606 GN=RHOB PE=1 SV=1	RHOB	0.523	0.019215741	Down	K07856	RHOB; Ras homolog gene family, member B		GO:0000278 mitotic cell cycle; GO:0000281 mitotic cytokinesis; GO:0000302 response to reactive oxygen species; GO:0000910 cytokinesis; GO:0002009 morphogenesis of an epithelium; GO:0003158 endothelium development; GO:0003159 morphogen	GO:0005622 intracellular; GO:0005623 cell; GO:0005634 nucleus; GO:0005737 cytoplasm; GO:0005768 endosome; GO:0005769 early endosome; GO:0005770 late endosome; GO:0005829 cytosol; GO:0005886 plasma membrane; GO:0010008 endosome membrane; GO:0012505	GO:0000166 nucleotide binding; GO:0001882 nucleoside binding; GO:0001883 purine nucleoside binding; GO:0003824 catalytic activity; GO:0003924 GTPase activity; GO:0005488 binding; GO:0005525 GTP binding; GO:0016462 pyrophosphatase activity; GO:0016787 hydrolase	PF00071	Ras family	U	KOG0393	Ras-related small GTPase, Rho type
Q96IF1	LIM domain-containing protein ajuba OS=Homo sapiens OX=9606 GN=AJUBA PE=1 SV=1	AJUBA	0.521	0.037020026	Down	K16682	AJUBA, LIMD1, WTIP; LIM domain-containing protein	map04390 Hippo signaling pathway; map04391 Hippo signaling pathway - fly; map04392 Hippo signaling pathway - multiple species	GO:0000086 G2/M transition of mitotic cell cycle; GO:0000122 negative regulation of transcription from RNA polymerase II promoter; GO:0000278 mitotic cell cycle; GO:0001666 response to hypoxia; GO:0001932 regulation of protein phosphorylation; GO:0001934 positive regulation	GO:0000932 P-body; GO:0005622 intracellular; GO:0005623 cell; GO:0005634 nucleus; GO:0005654 nucleoplasm; GO:0005667 transcription factor complex; GO:0005737 cytoplasm; GO:0005794 Golgi apparatus; GO:0005815 microtubule organizing center;	GO:0003682 chromatin binding; GO:0003712 transcription cofactor activity; GO:0003714 transcription corepressor activity; GO:0003779 actin binding; GO:0005488 binding; GO:0005515 protein binding; GO:0008092 cytoskeletal protein binding; GO:0044877 macromole	PF00412	LIM domain	K	KOG1704	FOG: LIM domain
Q6DKJ4	Nucleoredoxin OS=Homo sapiens OX=9606 GN=NXN PE=1 SV=2	NXN	0.518	0.048137977	Down	K17609	NXN; nucleoredoxin [EC:1.8.1.8]		GO:0007275 multicellular organism development; GO:0008152 metabolic process; GO:0009636 response to toxic substance; GO:0009892 negative regulation of metabolic process; GO:0009966 regulation of signal transduction; GO:0009968 negative regulation	GO:0005622 intracellular; GO:0005623 cell; GO:0005634 nucleus; GO:0005737 cytoplasm; GO:0043226 organelle; GO:0043227 membrane-bounded organelle; GO:0043229 intracellular organelle; GO:0043231 intracellular membrane-bounded organelle; GO:00444	GO:0003824 catalytic activity; GO:0004791 thioredoxin-disulfide reductase activity; GO:0015036 disulfide oxidoreductase activity; GO:0016209 antioxidant activity; GO:0016491 oxidoreductase activity; GO:0016651 oxidoreductase activity, acting on NAD(P)H;	PF13905; PF13848	Thioredoxin-like; Thioredoxin-like domain	O	KOG2501	Thioredoxin, nucleoredoxin and related proteins

Q9H668	CST complex subunit STN1 OS=Homo sapiens OX=9606 GN=STN1 PE=1 SV=2	STN1	0.506	0.045355471	Down	K23312	STN1; CST complex subunit STN1		GO:0000723 telomere maintenance; GO:0006139 nucleobase-containing compound metabolic process; GO:0006259 DNA metabolic process; GO:0006275 regulation of DNA replication; GO:0006725 cellular aromatic compound metabolic process; GO:0006807 nitrogen compound metabolic	GO:0000228 nuclear chromosome; GO:0000781 chromosome, telomeric region; GO:0000782 telomere cap complex; GO:0000783 nuclear telomere cap complex; GO:0000784 nuclear chromosome, telomeric region; GO:0001650 fibrillar center; GO:0005622	GO:0003676 nucleic acid binding; GO:0003677 DNA binding; GO:0003697 single-stranded DNA binding; GO:0005488 binding; GO:0042162 telomeric DNA binding; GO:0043047 single-stranded telomeric DNA binding; GO:0043565 sequence-specific DNA binding;	PF01336; PF09170	OB-fold nucleic acid binding domain; Domain of unknown function (DUF1879)	B	KOG3108	Single-stranded DNA-binding replication protein A (RPA), medium (30 kD) subunit
P36969	Phospholipid hydroperoxide glutathione peroxidase OS=Homo sapiens OX=9606 GN=GPX4 PE=1 SV=3	GPX4	0.505	0.036357449	Down	K05361	GPX4; phospholipid-hydroperoxide glutathione peroxidase [EC:1.11.1.12]	map00480 Glutathione metabolism; map01100 Metabolic pathways; map04216 Ferroptosis	GO:0000003 reproduction; GO:0001676 long-chain fatty acid metabolic process; GO:0006082 organic acid metabolic process; GO:0006325 chromatin organization; GO:0006518 peptide metabolic process; GO:0006575 cellular modified amino acid metabolic process; GO:00066	GO:0005622 intracellular; GO:0005623 cell; GO:0005634 nucleus; GO:0005635 nuclear envelope; GO:0005654 nucleoplasm; GO:0005737 cytoplasm; GO:0005739 mitochondrion; GO:0005829 cytosol; GO:0012505 endomembrane system; GO:0031967	GO:0003824 catalytic activity; GO:0004601 peroxidase activity; GO:0004602 glutathione peroxidase activity; GO:0005488 binding; GO:0005515 protein binding; GO:0008430 selenium binding; GO:0016209 antioxidant activity; GO:0016491 oxidoreductase activity; GO:00166			O	KOG1651	Glutathione peroxidase
O00220	Tumor necrosis factor receptor superfamily member 10A OS=Homo sapiens OX=9606 GN=TNFRSF10A PE=1 SV=3	TNFRSF10A	0.5	0.029436907	Down	K04722	TNFRSF10A_B, TRAILR1_2, DR4_5, CD261_2; tumor necrosis factor receptor superfamily member 10A/B	map04060 Cytokine-cytokine receptor interaction; map04061 Viral protein interaction with cytokine and cytokine receptor; map04210 Apoptosis; map04217 Necroptosis; map04650 Natural killer cell mediated cytotoxicity; map05130 Pathogenic Escherichia coli infection; map05164 Influenza A	GO:0001932 regulation of protein phosphorylation; GO:0001934 positive regulation of protein phosphorylation; GO:0002237 response to molecule of bacterial origin; GO:0002376 immune system process; GO:0006915 apoptotic process; GO:0006919 activation of cysteine-type	GO:0005623 cell; GO:0005886 plasma membrane; GO:0005887 integral component of plasma membrane; GO:0009986 cell surface; GO:0016020 membrane; GO:0016021 integral component of membrane; GO:0031224 intrinsic component of membrane; GO:0031226 intrinsic component of plasma membrane;	GO:0002020 protease binding; GO:0004888 transmembrane signaling receptor activity; GO:0005031 tumor necrosis factor-activated receptor activity; GO:0005035 death receptor activity; GO:0005488 binding; GO:0005515 protein binding; GO:0008134 transcription factor binding;	PF00020; PF00531	TNFR/NGFR cysteine-rich region; Death domain			
Q9BRS8	La-related protein 6 OS=Homo sapiens OX=9606 GN=LARP6 PE=1 SV=1	LARP6	0.495	0.014610868	Down	K18733	LARP6; la-related protein 6		GO:0006417 regulation of translation; GO:0009889 regulation of biosynthetic process; GO:0009891 positive regulation of biosynthetic process; GO:0009893 positive regulation of metabolic process; GO:0010468 regulation of gene expression; GO:0010556 regulation	GO:0005622 intracellular; GO:0005623 cell; GO:0005634 nucleus; GO:0005737 cytoplasm; GO:0005844 polysome; GO:0032991 macromolecular complex; GO:0043226 organelle; GO:0043227 membrane-bounded organelle; GO:0043229 intracellular organelle;	GO:0003676 nucleic acid binding; GO:0003723 RNA binding; GO:0003729 mRNA binding; GO:0005488 binding; GO:0005515 protein binding; GO:0008092 cytoskeletal protein binding; GO:0017022 myosin binding; GO:0035613 RNA stem-loop binding; GO:0048027 mRNA 5'-UTR binding;	PF05383; PF12901	La domain; SUZ-C motif	A	KOG1855	Predicted RNA-binding protein
Q8WVN8	Ubiquitin-conjugating enzyme E2 Q2 OS=Homo sapiens OX=9606 GN=UBE2Q2 PE=1 SV=1	UBE2Q2	0.494	0.030662369	Down	K10582	UBE2Q; ubiquitin-conjugating enzyme E2 Q [EC:2.3.2.23]	map04120 Ubiquitin mediated proteolysis	GO:0000209 protein polyubiquitination; GO:0006464 cellular protein modification process; GO:0006807 nitrogen compound metabolic process; GO:0008152 metabolic process; GO:0009987 cellular process; GO:0016567 protein ubiquitination; GO:0019538 protein metabolic process; GO:0032446 protein	GO:0005622 intracellular; GO:0005623 cell; GO:0005737 cytoplasm; GO:0005829 cytosol; GO:0044424 intracellular part; GO:0044444 cytoplasmic part; GO:0044464 cell part;	GO:0003824 catalytic activity; GO:0004842 ubiquitin-protein transferase activity; GO:0016740 transferase activity; GO:0019787 ubiquitin-like protein transferase activity;	PF00179	Ubiquitin-conjugating enzyme	O	KOG0897	Predicted ubiquitin-conjugating enzyme

Q9P021	Cysteine-rich PDZ-binding protein OS=Homo sapiens OX=9606 GN=CRIPT PE=1 SV=1	CRIPT	0.483	0.046845185	Down				GO:0000226 microtubule cytoskeleton organization; GO:0006996 organelle organization; GO:0007010 cytoskeleton organization; GO:0007017 microtubule-based process; GO:0008104 protein localization; GO:0009987 cellular process;	GO:0001650 fibrillar center; GO:0005622 intracellular; GO:0005623 cell; GO:0005634 nucleus; GO:0005730 nucleolus; GO:0014069 postsynaptic density; GO:0030425 dendrite; GO:0031974 membrane-enclosed lumen; GO:0031981 nuclear lumen; GO:0032279	GO:0005488 binding; GO:0005515 protein binding; GO:0008017 microtubule binding; GO:0008092 cytoskeletal protein binding; GO:0015631 tubulin binding; GO:0019904 protein domain specific binding; GO:0030165 PDZ domain binding; GO:0044877 macromolecular complex			Z	KOG3476	Microtubule-associated protein CRIPT

PMB72h *vs* Control

Protein accession	Protein description	Gene name	PMB72h/Control Ratio	PMB72h/Control P value	Regulated Type	KEGG Gene	KEGG pathway	Biological Process	Cellular Component	Molecular Function	Pfam ID	Domain description	KOG category	KOG NO.	KOG description
P57739	Claudin-2 OS=Homo sapiens OX=9606 GN=CLDN2 PE=1 SV=1	CLDN2	1.836	0.000244944	Up	CLDN; claudin	map04514 Cell adhesion molecules (CAMs); map04530 Tight junction; map04670 Leukocyte transendothelial migration; map05130 Pathogenic Escherichia coli infection; map05160 Hepatitis C	GO:0007155 cell adhesion; GO:0016338 calcium-independent cell-cell adhesion via plasma membrane cell-adhesion molecules; GO:0022610 biological adhesion; GO:0098609 cell-cell adhesion; GO:0098742 cell-cell adhesion via plasma-membrane adhesion molecules;	GO:0005911 cell-cell junction; GO:0005923 bicellular tight junction; GO:0016020 membrane; GO:0016021 integral component of membrane; GO:0030054 cell junction; GO:0031224 intrinsic component of membrane; GO:0043296 apical junction complex; GO:0044425 membrane	GO:0005488 binding; GO:0005515 protein binding; GO:0042802 identical protein binding;					
P40126	L-dopachrome tautomerase OS=Homo sapiens OX=9606 GN=DCT PE=1 SV=1	DCT	1.786	6.10174E-05	Up	DCT; dopachrome tautomerase [EC:5.3.3.12]	map00350 Tyrosine metabolism; map01100 Metabolic pathways; map04916 Melanogenesis	GO:0002052 positive regulation of neuroblast proliferation; GO:0006082 organic acid metabolic process; GO:0006520 cellular amino acid metabolic process; GO:0006570 tyrosine metabolic process; GO:0006582 melanin metabolic process; GO:0006583 melanin biosynthetic process from tyrosine;	GO:0005622 intracellular; GO:0005623 cell; GO:0005737 cytoplasm; GO:0005829 cytosol; GO:0012506 vesicle membrane; GO:0016020 membrane; GO:0016021 integral component of membrane; GO:0030659 cytoplasmic vesicle membrane; GO:0031090 organelle membrane;	GO:0003824 catalytic activity; GO:0004167 dopachrome isomerase activity; GO:0005488 binding; GO:0005507 copper ion binding; GO:0016491 oxidoreductase activity; GO:0016853 isomerase activity; GO:0016860 intramolecular oxidoreductase activity; GO:00168	PF00264	Common central domain of tyrosinase			
Q01581	Hydroxymethylglutaryl -CoA synthase, cytoplasmic OS=Homo sapiens OX=9606 GN=HMGCS1 PE=1 SV=2	HMGCS1	1.63	0.001138525	Up	E2.3.3.10; hydroxymethylglutaryl-CoA synthase [EC:2.3.3.10]	map00072 Synthesis and degradation of ketone bodies; map00280 Valine, leucine and isoleucine degradation; map00650 Butanoate metabolism; map00900 Terpenoid backbone biosynthesis; map01100 Metabolic pathways; map03320 PPAR signaling pathway	GO:0000003 reproduction; GO:0001889 liver development; GO:0003006 developmental process involved in reproduction; GO:0006066 alcohol metabolic process; GO:0006629 lipid metabolic process; GO:0006694 steroid biosynthetic process; GO:0006695 cholesterol	GO:0005622 intracellular; GO:0005623 cell; GO:0005737 cytoplasm; GO:0005829 cytosol; GO:0044424 intracellular part; GO:0044444 cytoplasmic part; GO:0044464 cell part;	GO:0003824 catalytic activity; GO:0004421 hydroxymethylglutaryl-CoA synthase activity; GO:0005488 binding; GO:0005515 protein binding; GO:0008144 drug binding; GO:0016740 transferase activity; GO:0016746 transferase activity, transferring acyl groups; GO:0016853	PF01154; PF08540	Hydroxymethylglutaryl-coenzyme A synthase N terminal; Hydroxymethylglutaryl-coenzyme A synthase C terminal	H	KOG1393	Hydroxymethylglutaryl-CoA synthase
Q9Y6V0	Protein piccolo OS=Homo sapiens OX=9606 GN=PCLO PE=1 SV=5	PCLO	1.577	0.000258374	Up	PCLO; protein piccolo	map04911 Insulin secretion	GO:0001505 regulation of neurotransmitter levels; GO:0006810 transport; GO:0006836 neurotransmitter transport; GO:0006887 exocytosis; GO:0006996 organelle organization; GO:0007010 cytoskeleton organization; GO:0007154 cell	GO:0005622 intracellular; GO:0005623 cell; GO:0005737 cytoplasm; GO:0005856 cytoskeleton; GO:0005938 cell cortex; GO:0030863 cortical cytoskeleton; GO:0043226 organelle; GO:0043228 non-membrane-bounded organelle; GO:0043229 intracellula	GO:0005488 binding; GO:0005509 calcium ion binding; GO:0005515 protein binding; GO:0005522 profilin binding; GO:0005543 phospholipid binding; GO:0005544 calcium-dependent phospholipid binding; GO:0008289 lipid binding; GO:0043167 ion binding; GO:0043168 anion binding;	PF05715; PF00595; PF00168	Piccolo Zn-finger; PDZ domain (Also known as DHR or GLGF); C2 domain	U	KOG2060	Rab3 effector RIM1 and related proteins, contain PDZ and C2 domains

Q96GN5	Cell division cycle-associated 7-like protein OS=Homo sapiens OX=9606 GN=CDCA7L PE=1 SV=2	CDCA7L	1.545	0.007436041	Up			GO:0008284 positive regulation of cell proliferation; GO:0042127 regulation of cell proliferation; GO:0048518 positive regulation of biological process; GO:0048522 positive regulation of cellular process; GO:0050789 regulation of biological process; GO:0050794	GO:0001650 fibrillar center; GO:0005622 intracellular; GO:0005623 cell; GO:0005634 nucleus; GO:0005730 nucleolus; GO:0005737 cytoplasm; GO:0005829 cytosol; GO:0031974 membrane-enclosed lumen; GO:0031981 nuclear lumen; GO:0043226 organelle; GO:00432		PF10497	Zinc-finger domain of monoamine-oxidase A repressor R1			
P23508	Colorectal mutant cancer protein OS=Homo sapiens OX=9606 GN=MCC PE=1 SV=2	MCC	1.535	0.002935614	Up			GO:0007154 cell communication; GO:0007165 signal transduction; GO:0008104 protein localization; GO:0008285 negative regulation of cell proliferation; GO:0009966 regulation of signal transduction; GO:0009968 negative regulation of signal transduction; GO:00099	GO:0005622 intracellular; GO:0005623 cell; GO:0005634 nucleus; GO:0005654 nucleoplasm; GO:0005737 cytoplasm; GO:0005829 cytosol; GO:0005886 plasma membrane; GO:0016020 membrane; GO:0031974 membrane-enclosed lumen; GO:0031981 nuclear lumen;	GO:0038023 signaling receptor activity; GO:0060089 molecular transducer activity;	PF10506	PDZ domain of MCC-2 bdg protein for Usher syndrome			
Q9UBF9	Myotilin OS=Homo sapiens OX=9606 GN=MYOT PE=1 SV=2	MYOT	1.482	0.03720008	Up	MYOT; myotilin		GO:0003008 system process; GO:0003012 muscle system process; GO:0006936 muscle contraction; GO:0032501 multicellular organismal process;	GO:0005622 intracellular; GO:0005623 cell; GO:0005737 cytoplasm; GO:0005856 cytoskeleton; GO:0015629 actin cytoskeleton; GO:0030016 myofibril; GO:0030017 sarcomere; GO:0030018 Z disc; GO:0031674 I band; GO:0043226 organelle; GO:00432	GO:0005198 structural molecule activity; GO:0005488 binding; GO:0005515 protein binding; GO:0008092 cytoskeletal protein binding; GO:0008307 structural constituent of muscle; GO:0042805 actinin binding; GO:0051393 alpha-actinin binding;	PF07679	Immunoglobulin I-set domain			
Q8TBP0	TBC1 domain family member 16 OS=Homo sapiens OX=9606 GN=TBC1D16 PE=2 SV=1	TBC1D16	1.477	0.002550409	Up			GO:0001919 regulation of receptor recycling; GO:0006810 transport; GO:0006886 intracellular protein transport; GO:0008104 protein localization; GO:0015031 protein transport; GO:0015833 peptide transport; GO:0019222 regulation of metabolic process; GO:0023051	GO:0005622 intracellular; GO:0005623 cell; GO:0005737 cytoplasm; GO:0005768 endosome; GO:0005769 early endosome; GO:0005829 cytosol; GO:0012505 endomembrane system; GO:0031410 cytoplasmic vesicle; GO:0031982 vesicle; GO:0043226 organelle;	GO:0005096 GTPase activator activity; GO:0005488 binding; GO:0005515 protein binding; GO:0008047 enzyme activator activity; GO:0017016 Ras GTPase binding; GO:0017137 Rab GTPase binding; GO:0019899 enzyme binding; GO:0030234 enzyme regulator activity; GO:0030695 GTPase regulator			U	KOG2224	Uncharacterized conserved protein, contains TBC domain

P27987	Inositol-trisphosphate 3-kinase B OS=Homo sapiens OX=9606 GN=ITPKB PE=1 SV=5	ITPKB	1.472	0.011602995	Up	ITPK; 1D-myo-inositol-triphosphate 3-kinase [EC:2.7.1.127]	map00562 Inositol phosphate metabolism; map01100 Metabolic pathways; map04020 Calcium signaling pathway; map04070 Phosphatidylinositol signaling system	GO:0006066 alcohol metabolic process; GO:0006793 phosphorus metabolic process; GO:0006796 phosphate-containing compound metabolic process; GO:0007154 cell communication; GO:0007165 signal transduction; GO:0008152 metabolic process; GO:0009987 cellular process;	GO:0005622 intracellular; GO:0005623 cell; GO:0005737 cytoplasm; GO:0005829 cytosol; GO:0044424 intracellular part; GO:0044444 cytoplasmic part; GO:0044464 cell part;	GO:0003824 catalytic activity; GO:0008440 inositol-1,4,5-trisphosphate 3-kinase activity; GO:0016301 kinase activity; GO:0016740 transferase activity; GO:0016772 transferase activity, transferring phosphorus-containing groups; GO:0016773 phosphotransferase activity, alcohol group as			I	KOG1621	1D-myo-inositol-triphosphate 3-kinase A
A8MQ03	Cysteine-rich tail protein 1 OS=Homo sapiens OX=9606 GN=CYSRT1 PE=1 SV=1	CYSRT1	1.471	0.024303519	Up										
Q92570	Nuclear receptor subfamily 4 group A member 3 OS=Homo sapiens OX=9606 GN=NR4A3 PE=1 SV=3	NR4A3	1.458	0.000592332	Up	NR4A3, NOR1; nuclear receptor subfamily 4 group A member 3	map05202 Transcriptional misregulation in cancer	GO:0000122 negative regulation of transcription from RNA polymerase II promoter; GO:0000302 response to reactive oxygen species; GO:0000902 cell morphogenesis; GO:0000904 cell morphogenesis involved in differentiation; GO:0001704 formation of primary	GO:0005622 intracellular; GO:0005623 cell; GO:0005634 nucleus; GO:0005654 nucleoplasm; GO:0005667 transcription factor complex; GO:0031974 membrane-enclosed lumen; GO:0031981 nuclear lumen; GO:0032991 macromolecular complex; GO:00432	GO:0000976 transcription regulatory region sequence-specific DNA binding; GO:0000977 RNA polymerase II regulatory region sequence-specific DNA binding; GO:0000978 RNA polymerase II core promoter proximal region sequence-specific DNA binding;	PF00105; PF00104	Zinc finger, C4 type (two domains); Ligand-binding domain of nuclear hormone receptor	K	KOG4217	Nuclear receptors of the nerve growth factor-induced protein B type
Q9ULX9	Transcription factor MafF OS=Homo sapiens OX=9606 GN=MAFF PE=1 SV=2	MAFF	1.452	0.003229509	Up	MAFF_G_K; transcription factor MAFF/G/K		GO:0000003 reproduction; GO:0001701 in utero embryonic development; GO:0006139 nucleobase-containing compound metabolic process; GO:0006351 transcription, DNA-templated; GO:0006355 regulation of transcription, DNA-templated; GO:0006357 regulation	GO:0005622 intracellular; GO:0005623 cell; GO:0005634 nucleus; GO:0005654 nucleoplasm; GO:0005737 cytoplasm; GO:0005739 mitochondrion; GO:0031974 membrane-enclosed lumen; GO:0031981 nuclear lumen; GO:0043226 organelle; GO:00432	GO:0000981 RNA polymerase II transcription factor activity, sequence-specific DNA binding; GO:0001228 transcriptional activator activity, RNA polymerase II transcription regulatory region sequence-specific binding; GO:0003676 nucleic acid binding;			K	KOG4196	bZIP transcription factor MafK
P98088	Mucin-5AC OS=Homo sapiens OX=9606 GN=MUC5AC PE=1 SV=4	MUC5AC	1.448	0.000730795	Up	MUC5AC; mucin-5AC	map04657 IL-17 signaling pathway	GO:0002218 activation of innate immune response; GO:0002220 innate immune response activating cell surface receptor signaling pathway; GO:0002223 stimulatory C-type lectin receptor signaling pathway; GO:0002253 activation of immune response; GO:0002376 immune system	GO:0005576 extracellular region; GO:0005615 extracellular space; GO:0005622 intracellular; GO:0005623 cell; GO:0005737 cytoplasm; GO:0005794 Golgi apparatus; GO:0005796 Golgi lumen; GO:0005886 plasma membrane; GO:0012505 endomembrane system;	GO:0005198 structural molecule activity; GO:0005201 extracellular matrix structural constituent;	PF08742; PF01826	C8 domain; Trypsin Inhibitor like cysteine rich domain	W	KOG1216	von Willebrand factor and related coagulation proteins

Q15800	Methylsterol monooxygenase 1 OS=Homo sapiens OX=9606 GN=MSMO1 PE=1 SV=1	MSMO1	1.436	0.004932119	Up	MESO1, ERG25; methylsterol monooxygenase [EC:1.14.18.9]	map00100 Steroid biosynthesis; map01100 Metabolic pathways	GO:0006066 alcohol metabolic process; GO:0006082 organic acid metabolic process; GO:0006629 lipid metabolic process; GO:0006631 fatty acid metabolic process; GO:0006694 steroid biosynthetic process; GO:0006695 cholesterol biosynthetic process; GO:0008152 metabolic process;	GO:0005622 intracellular; GO:0005623 cell; GO:0005737 cytoplasm; GO:0005783 endoplasmic reticulum; GO:0005789 endoplasmic reticulum membrane; GO:0005886 plasma membrane; GO:0012505 endomembrane system; GO:0016020 membrane; GO:00160	GO:0000254 C-4 methylsterol oxidase activity; GO:0003824 catalytic activity; GO:0004497 monooxygenase activity; GO:0016491 oxidoreductase activity; GO:0016705 oxidoreductase activity, acting on paired donors, with incorporation or reduction of			I	KOG0873	C-4 sterol methyl oxidase
Q9Y274	Type 2 lactosamine alpha-2,3-sialyltransferase OS=Homo sapiens OX=9606 GN=ST3GAL6 PE=1 SV=1	ST3GAL6	1.419	0.000210036	Up	SIAT10, ST3GAL6; neolactotetraosylceramide alpha-2,3-sialyltransferase (sialyltransferase 10) [EC:2.4.99.6]	map00601 Glycosphingolipid biosynthesis - lacto and neolacto series; map01100 Metabolic pathways	GO:0005975 carbohydrate metabolic process; GO:0006022 aminoglycan metabolic process; GO:0006023 aminoglycan biosynthetic process; GO:0006024 glycosaminoglycan biosynthetic process; GO:0006082 organic acid metabolic process; GO:0006464 cellular protein	GO:0000139 Golgi membrane; GO:0005622 intracellular; GO:0005623 cell; GO:0005737 cytoplasm; GO:0005794 Golgi apparatus; GO:0012505 endomembrane system; GO:0016020 membrane; GO:0016021 integral component of membrane; GO:0031090 organelle	GO:0003824 catalytic activity; GO:0003836 beta-galactoside (CMP) alpha-2,3-sialyltransferase activity; GO:0008373 sialyltransferase activity; GO:0016740 transferase activity; GO:0016757 transferase activity, transferring glycosyl groups; GO:0052798 beta-galactoside alpha-2,3-			G	KOG2692	Sialyltransferase
O15018	PDZ domain-containing protein 2 OS=Homo sapiens OX=9606 GN=PDZD2 PE=1 SV=4	PDZD2	1.41	0.002234849	Up			GO:0007154 cell communication; GO:0007165 signal transduction; GO:0009987 cellular process; GO:0023052 signaling; GO:0035556 intracellular signal transduction; GO:0050789 regulation of biological process; GO:0050794 regulation of cellular process;	GO:0005576 extracellular region; GO:0005622 intracellular; GO:0005623 cell; GO:0005634 nucleus; GO:0005737 cytoplasm; GO:0005783 endoplasmic reticulum; GO:0005911 cell-cell junction; GO:0012505 endomembrane system; GO:0014069 postsynapti		PF00595	PDZ domain (Also known as DHR or GLGF)	F	KOG3528	FOG: PDZ domain
Q9UJC3	Protein Hook homolog 1 OS=Homo sapiens OX=9606 GN=HOOK1 PE=1 SV=2	HOOK1	1.401	0.0016908	Up	HOOK1; protein HOOK1		GO:0000003 reproduction; GO:0000226 microtubule cytoskeleton organization; GO:0003006 developmental process involved in reproduction; GO:0006810 transport; GO:0006996 organelle organization; GO:0007010 cytoskeleto	GO:0005622 intracellular; GO:0005623 cell; GO:0005737 cytoplasm; GO:0005813 centrosome; GO:0005815 microtubule organizing center; GO:0005856 cytoskeleton; GO:0015630 microtubule cytoskeleton; GO:0032991	GO:0003779 actin binding; GO:0005488 binding; GO:0005515 protein binding; GO:0008017 microtubule binding; GO:0008092 cytoskeletal protein binding; GO:0015631 tubulin binding; GO:0042802 identical protein binding; GO:0051959 dynein light intermediate chain binding;					

P15056	Serine/threonine-protein kinase B-raf OS=Homo sapiens OX=9606 GN=BRAF PE=1 SV=4	BRAF	1.399	0.044021857	Up	BRAF; B-Raf proto-oncogene serine/threonine-protein kinase [EC:2.7.11.1]	map01521 EGFR tyrosine kinase inhibitor resistance; map01522 Endocrine resistance; map04010 MAPK signaling pathway; map04012 ErbB signaling pathway; map04013 MAPK signaling pathway - fly; map04015 Rap1 signaling pathway; map04024 cAMP signaling pathway; map04062 Chemokine signaling pathway; map04068 FoxO signaling pathway; map04150 mTOR signaling pathway; map04214 Apoptosis - fly; map04270 Vascular smooth muscle contraction; map04320 Dorso-ventral axis formation; map04510 Focal adhesion; map04650 Natural killer cell mediated cytotoxicity; map04720 Long-term potentiation; map04722 Neurotrophin signaling pathway; map04726 Serotonergic synapse; map04730 Long-term depression; map04810 Regulation of actin cytoskeleton; map04910 Insulin signaling pathway; map04914 Progesterone-mediated oocyte maturation; map04928 Parathyroid hormone synthesis, secretion and action; map04934 Cushing syndrome; map05034 Alcoholism; map05160 Hepatitis C; map05161 Hepatitis B; map05200 Pathways in cancer; map05205 Proteoglycans in cancer; map05210 Colorectal cancer; map05211 Renal cell carcinoma; map05212 Pancreatic cancer; map05213 Endometrial cancer; map05214 Glioma; map05215 Prostate cancer; map05216 Thyroid cancer; map05218 Melanoma; map05219 Bladder cancer; map05220 Chronic myeloid leukemia; map05221 Acute myeloid leukemia; map05223 Non-small cell lung cancer; map05224 Breast cancer; map05225 Hepatocellular carcinoma; map05226	GO:0000165 MAPK cascade; GO:0000186 activation of MAPKK activity; GO:0001505 regulation of neurotransmitter levels; GO:0001775 cell activation; GO:0001932 regulation of protein phosphorylation; GO:0001934 positive regulation of protein phosphorylation; GO:00022	GO:0005622 intracellular; GO:0005623 cell; GO:0005634 nucleus; GO:0005737 cytoplasm; GO:0005739 mitochondrion; GO:0005829 cytosol; GO:0005886 plasma membrane; GO:0016020 membrane; GO:0042995 cell projection; GO:0043005 neuron projection; GO:0043226	GO:0003824 catalytic activity; GO:0004672 protein kinase activity; GO:0004674 protein serine/threonine kinase activity; GO:0004709 MAP kinase kinase kinase activity; GO:0005488 binding; GO:0005509 calcium ion binding; GO:0005515 protein binding; GO:0016301 kinase activity;	PF02196; PF00130; PF07714	Raf-like Ras-binding domain; Phorbol esters/diacylglycerol binding domain (C1 domain); Protein tyrosine kinase	T	KOG0193	Serine/threonine protein kinase RAF
P37268	Squalene synthase OS=Homo sapiens OX=9606 GN=FDFT1 PE=1 SV=1	FDFT1	1.384	0.000574063	Up	FDFT1; farnesyl-diphosphate farnesyltransferase [EC:2.5.1.21]	map00100 Steroid biosynthesis; map01100 Metabolic pathways	GO:0006066 alcohol metabolic process; GO:0006629 lipid metabolic process; GO:0006644 phospholipid metabolic process; GO:0006694 steroid biosynthetic process; GO:0006695 cholesterol biosynthetic process; GO:0006720 isoprenoid metabolic process; GO:0006721 terpenoid	GO:0005622 intracellular; GO:0005623 cell; GO:0005737 cytoplasm; GO:0005783 endoplasmic reticulum; GO:0005789 endoplasmic reticulum membrane; GO:0012505 endomembrane system; GO:0016020 membrane; GO:0016021 integral component of	GO:0003824 catalytic activity; GO:0004310 farnesyl-diphosphate farnesyltransferase activity; GO:0004311 farnesyltranstransferase activity; GO:0004659 prenyltransferase activity; GO:0016740 transferase activity; GO:0016765 transferase activity, transferring alkyl or	PF00494	Squalene/phytoene synthase	I	KOG1459	Squalene synthetase
P50219	Motor neuron and pancreas homeobox protein 1 OS=Homo sapiens OX=9606 GN=MNX1 PE=1 SV=3	MNX1	1.376	0.010910009	Up	HLXB9, HB9; homeobox protein HB9	map04950 Maturity onset diabetes of the young	GO:0000902 cell morphogenesis; GO:0000904 cell morphogenesis involved in differentiation; GO:0001708 cell fate specification; GO:0001764 neuron migration; GO:0002376 immune system process; GO:0003002 regionalization; GO:0006355 regulation of transcriptio	GO:0005622 intracellular; GO:0005623 cell; GO:0005634 nucleus; GO:0005730 nucleolus; GO:0005737 cytoplasm; GO:0005829 cytosol; GO:0031974 membrane-enclosed lumen; GO:0031981 nuclear lumen; GO:0043226 organelle; GO:0043227 membrane-bounded	GO:0000981 RNA polymerase II transcription factor activity, sequence-specific DNA binding; GO:0003700 transcription factor activity, sequence-specific DNA binding;	PF00046	Homeobox domain	K	KOG0489	Transcription factor zerknullt and related HOX domain proteins
Q9Y2J2	Band 4.1-like protein 3 OS=Homo sapiens OX=9606 GN=EPB41L3 PE=1 SV=2	EPB41L3	1.374	0.001298924	Up	EPB41, 4.1R; erythrocyte membrane protein band 4.1		GO:0000902 cell morphogenesis; GO:0002175 protein localization to paranode region of axon; GO:0006996 organelle organization; GO:0007009 plasma membrane organization; GO:0007010 cytoskeleton organization; GO:0007016 cytoskeletal anchoring	GO:0005622 intracellular; GO:0005623 cell; GO:0005737 cytoplasm; GO:0005829 cytosol; GO:0005856 cytoskeleton; GO:0005886 plasma membrane; GO:0005911 cell-cell junction; GO:0014069 postsynaptic density; GO:0016020 membrane; GO:0030054 cell junction;	GO:0005198 structural molecule activity; GO:0005200 structural constituent of cytoskeleton;	PF09379; PF00373; PF09380; PF04382; PF05902	FERM N-terminal domain ; FERM central domain; FERM C-terminal PH-like domain; SAB domain; 4.1 protein C-terminal domain (CTD)	Z	KOG3530	FERM domain protein EHM2
P80297	Metallothionein-1X OS=Homo sapiens OX=9606 GN=MT1X PE=1 SV=1	MT1X	1.372	0.010347643	Up	MT1_2; metallothionein 1/2	map04212 Longevity regulating pathway - worm; map04978 Mineral absorption				PF00131	Metallothionein			

P11055	Myosin-3 OS=Homo sapiens OX=9606 GN=MYH3 PE=1 SV=3	MYH3	1.371	0.002705681	Up	MYH; myosin heavy chain	map04530 Tight junction; map05130 Pathogenic Escherichia coli infection	GO:0003008 system process; GO:0003009 skeletal muscle contraction; GO:0003012 muscle system process; GO:0006139 nucleobase-containing compound metabolic process; GO:0006163 purine nucleotide metabolic process; GO:0006464 cellular protein modification process; GO:0006470 protein	GO:0005622 intracellular; GO:0005623 cell; GO:0005737 cytoplasm; GO:0005829 cytosol; GO:0005856 cytoskeleton; GO:0005859 muscle myosin complex; GO:0015629 actin cytoskeleton; GO:0016459 myosin complex; GO:0016460 myosin II complex; GO:0030016	GO:0000146 microfilament motor activity; GO:0003774 motor activity; GO:0003779 actin binding; GO:0003824 catalytic activity; GO:0004721 phosphoprotein phosphatase activity; GO:0004722 protein serine/threonine phosphatase activity; GO:0005488 binding; GO:0005515 protein binding;	PF02736; PF00063	Myosin N-terminal SH3-like domain; Myosin head (motor domain)	Z	KOG0161	Myosin class II heavy chain
P13929	Beta-enolase OS=Homo sapiens OX=9606 GN=ENO3 PE=1 SV=5	ENO3	1.362	0.000782974	Up	ENO, eno; enolase [EC:4.2.1.11]	map00010 Glycolysis / Gluconeogenesis; map01100 Metabolic pathways; map01200 Carbon metabolism; map01230 Biosynthesis of amino acids; map03018 RNA degradation; map04066 HIF-1 signaling pathway	GO:0005975 carbohydrate metabolic process; GO:0005996 monosaccharide metabolic process; GO:0006006 glucose metabolic process; GO:0006007 glucose catabolic process; GO:0006082 organic acid metabolic process; GO:0006090 pyruvate metabolic process; GO:00060	GO:0005622 intracellular; GO:0005623 cell; GO:0005737 cytoplasm; GO:0005829 cytosol; GO:0005886 plasma membrane; GO:0016020 membrane; GO:0044424 intracellular part; GO:0044444 cytoplasmic part; GO:0044464 cell part; GO:0071944 cell periphery;	GO:0003824 catalytic activity; GO:0004634 phosphopyruvate hydratase activity; GO:0005488 binding; GO:0005515 protein binding; GO:0016829 lyase activity; GO:0016835 carbon-oxygen lyase activity; GO:0016836 hydro-lyase activity; GO:0042802 identical protein binding;	PF03952; PF00113	Enolase, N-terminal domain; Enolase, C-terminal TIM barrel domain	G	KOG2670	Enolase
Q5U3C3	Transmembrane protein 164 OS=Homo sapiens OX=9606 GN=TMEM164 PE=2 SV=1	TMEM164	1.357	0.039401893	Up										
Q8NC26	Zinc finger protein 114 OS=Homo sapiens OX=9606 GN=ZNF114 PE=1 SV=1	ZNF114	1.356	0.000527766	Up	KRAB; KRAB domain-containing zinc finger protein	map05168 Herpes simplex virus 1 infection	GO:0006355 regulation of transcription, DNA-templated; GO:0006357 regulation of transcription from RNA polymerase II promoter; GO:0009889 regulation of biosynthetic process; GO:0010468 regulation of gene expression; GO:0010556 regulation		GO:0000981 RNA polymerase II transcription factor activity, sequence-specific DNA binding; GO:0003700 transcription factor activity, sequence-specific DNA binding; GO:0005488 binding; GO:0005515 protein binding; GO:0042802 identical protein binding;	PF00096	Zinc finger, C2H2 type	K	KOG1721	FOG: Zn-finger
Q16655	Melanoma antigen recognized by T-cells 1 OS=Homo sapiens OX=9606 GN=MLANA PE=1 SV=1	MLANA	1.355	0.006586795	Up	MLANA, MART1; melanoma antigen recognized by T-cells 1			GO:0000139 Golgi membrane; GO:0005622 intracellular; GO:0005623 cell; GO:0005737 cytoplasm; GO:0005783 endoplasmic reticulum; GO:0005789 endoplasmic reticulum membrane; GO:0005794 Golgi apparatus; GO:0005802 trans-Golgi network; GO:0005886 plasma						

Q9H0T7	Ras-related protein Rab-17 OS=Homo sapiens OX=9606 GN=RAB17 PE=1 SV=2	RAB17	1.355	0.00012786	Up	RAB17; Ras-related protein Rab-17		GO:0002251 organ or tissue specific immune response; GO:0002376 immune system process; GO:0002385 mucosal immune response; GO:0002386 immune response in mucosal-associated lymphoid tissue; GO:0002414 immunoglobulin transcytosis in epithelial cells; GO:0002415	GO:0005622 intracellular; GO:0005623 cell; GO:0005737 cytoplasm; GO:0005768 endosome; GO:0005769 early endosome; GO:0005886 plasma membrane; GO:0010008 endosome membrane; GO:0012505 endomembrane system; GO:0016020 membrane; GO:00163	GO:0000166 nucleotide binding; GO:0001882 nucleoside binding; GO:0001883 purine nucleoside binding; GO:0003824 catalytic activity; GO:0003924 GTPase activity; GO:0005488 binding; GO:0016462 pyrophosphatase activity; GO:0016787 hydrolase activity; GO:0016817	PF00071	Ras family	U	KOG0092	GTPase Rab5/YPT51 and related small G protein superfamily GTPases
P78317	E3 ubiquitin-protein ligase RNF4 OS=Homo sapiens OX=9606 GN=RNF4 PE=1 SV=1	RNF4	1.349	0.023272786	Up	RNF4; E3 ubiquitin-protein ligase RNF4 [EC:2.3.2.27]		GO:0000209 protein polyubiquitination; GO:0006355 regulation of transcription, DNA-templated; GO:0006357 regulation of transcription from RNA polymerase II promoter; GO:0006464 cellular protein modification process; GO:0006508 proteolysis; GO:0006511	GO:0005622 intracellular; GO:0005623 cell; GO:0005634 nucleus; GO:0005654 nucleoplasm; GO:0005737 cytoplasm; GO:0016604 nuclear body; GO:0016605 PML body; GO:0031974 membrane-enclosed lumen; GO:0031981 nuclear lumen; GO:0043226	GO:0003676 nucleic acid binding; GO:0003677 DNA binding; GO:0003682 chromatin binding; GO:0003700 transcription factor activity, sequence-specific DNA binding; GO:0003712 transcription cofactor activity; GO:0003713 transcription coactivator activity;	PF13639	Ring finger domain	K	KOG0320	Predicted E3 ubiquitin ligase
P54652	Heat shock-related 70 kDa protein 2 OS=Homo sapiens OX=9606 GN=HSPA2 PE=1 SV=1	HSPA2	1.343	0.00029824	Up	HSPA1s; heat shock 70kDa protein 1/2/6/8	map03040 Spliceosome; map04010 MAPK signaling pathway; map04141 Protein processing in endoplasmic reticulum; map04144 Endocytosis; map04213 Longevity regulating pathway - multiple species; map04612 Antigen processing and presentation; map04915 Estrogen signaling pathway; map05134 Legionellosis; map05145 Toxoplasmosis; map05162 Measles	GO:0000003 reproduction; GO:0000280 nuclear division; GO:0001932 regulation of protein phosphorylation; GO:0001934 positive regulation of protein phosphorylation; GO:0003006 developmental process involved in reproduction; GO:0006457 protein folding; GO:00069	GO:0000228 nuclear chromosome; GO:0000793 condensed chromosome; GO:0000794 condensed nuclear chromosome; GO:0000795 synaptonemal complex; GO:0001673 male germ cell nucleus; GO:0005622 intracellular; GO:0005623 cell; GO:00056	GO:0005488 binding; GO:0005515 protein binding; GO:0008092 cytoskeletal protein binding; GO:0008289 lipid binding; GO:0019899 enzyme binding; GO:0019904 protein domain specific binding; GO:0048156 tau protein binding; GO:0051082 unfolded protein binding; GO:00510			O	KOG0101	Molecular chaperones HSP70/HSC70, HSP70 superfamily
P51160	Cone cGMP-specific 3',5'-cyclic phosphodiesterase subunit alpha' OS=Homo sapiens OX=9606 GN=PDE6C PE=1 SV=2	PDE6C	1.339	0.007486473	Up	PDE6C; cone cGMP-specific 3',5'-cyclic phosphodiesterase subunit alpha' [EC:3.1.4.35]	map00230 Purine metabolism; map01100 Metabolic pathways	GO:0001654 eye development; GO:0001754 eye photoreceptor cell differentiation; GO:0003008 system process; GO:0003407 neural retina development; GO:0007154 cell			PF01590; PF00233	GAF domain; 3'5'-cyclic nucleotide phosphodiesterase	T	KOG3689	Cyclic nucleotide phosphodiesterase
Q4ZG55	Protein GREB1 OS=Homo sapiens OX=9606 GN=GREB1 PE=2 SV=1	GREB1	1.338	0.000690177	Up				GO:0005622 intracellular; GO:0005623 cell; GO:0005634 nucleus; GO:0005654 nucleoplasm; GO:0031974 membrane-enclosed lumen; GO:0031981 nuclear lumen; GO:0043226 organelle; GO:0043227 membrane-bounded organelle; GO:0043229 intracellula						

Q9H8H3	Methyltransferase -like protein 7A OS=Homo sapiens OX=9606 GN=METTL7A PE=1 SV=1	METTL7A	1.334	0.006200511	Up			GO:0001775 cell activation; GO:0002252 immune effector process; GO:0002263 cell activation involved in immune response; GO:0002274 myeloid leukocyte activation; GO:0002275 myeloid cell activation involved in immune response; GO:0002283 neutrophil activation involved in immune response;	GO:0005576 extracellular region; GO:0005622 intracellular; GO:0005623 cell; GO:0005737 cytoplasm; GO:0005811 lipid particle; GO:0012505 endomembrane system; GO:0030141 secretory granule; GO:0031410 cytoplasmic vesicle; GO:0031974		PF08241	Methyltransferase domain	Q	KOG4300	Predicted methyltransferase
O94788	Retinal dehydrogenase 2 OS=Homo sapiens OX=9606 GN=ALDH1A2 PE=1 SV=3	ALDH1A2	1.333	0.003099618	Up	ALDH1A; retinal dehydrogenase [EC:1.2.1.36]	map00830 Retinol metabolism; map01100 Metabolic pathways	GO:0001101 response to acid chemical; GO:0001523 retinoid metabolic process; GO:0001568 blood vessel development; GO:0001654 eye development; GO:0001655 urogenital system development; GO:0001756 somitogenesis; GO:0001822 kidney developme	GO:0005622 intracellular; GO:0005623 cell; GO:0005737 cytoplasm; GO:0005829 cytosol; GO:0044424 intracellular part; GO:0044444 cytoplasmic part; GO:0044464 cell part; GO:0048471 perinuclear region of cytoplasm;	GO:0001758 retinal dehydrogenase activity; GO:0003824 catalytic activity; GO:0004028 3-chloroallyl aldehyde dehydrogenase activity; GO:0004029 aldehyde dehydrogenase (NAD) activity; GO:0005488 binding; GO:0005501 retinoid binding; GO:0008289 lipid binding; GO:00164			C	KOG2450	Aldehyde dehydrogenase
Q5K4L6	Solute carrier family 27 member 3 OS=Homo sapiens OX=9606 GN=SLC27A3 PE=1 SV=4	SLC27A3	1.329	0.004056092	Up	SLC27A3, FATP3; solute carrier family 27 (fatty acid transporter), member 3 [EC:6.2.1.-]	map04931 Insulin resistance	GO:0001676 long-chain fatty acid metabolic process; GO:0006082 organic acid metabolic process; GO:0006139 nucleobase-containing compound metabolic process; GO:0006163 purine nucleotide metabolic process; GO:0006629 lipid metabolic process; GO:0006631 fatty acid metabolic	GO:0005622 intracellular; GO:0005623 cell; GO:0005737 cytoplasm; GO:0005739 mitochondrion; GO:0005759 mitochondrial matrix; GO:0005783 endoplasmic reticulum; GO:0012505 endomembrane system; GO:0031974 membrane-enclosed	GO:0003824 catalytic activity; GO:0004321 fatty-acyl-CoA synthase activity; GO:0004467 long-chain fatty acid-CoA ligase activity; GO:0015645 fatty acid ligase activity; GO:0016405 CoA-ligase activity; GO:0016408 C-acyltransferase activity; GO:0016740 transferase activity;	PF13193	Domain of unknown function (DUF4009)	I	KOG1179	Very long-chain acyl-CoA synthetase/fatty acid transporter
P02795	Metallothionein-2 OS=Homo sapiens OX=9606 GN=MT2A PE=1 SV=1	MT2A	1.322	0.005258103	Up						PF00131	Metallothionein			
Q56NI9	N-acetyltransferase ESCO2 OS=Homo sapiens OX=9606 GN=ESCO2 PE=1 SV=1	ESCO2	1.321	6.98026E-05	Up	ESCO, ECO1; N-acetyltransferase [EC:2.3.1.-]		GO:0002244 hematopoietic progenitor cell differentiation; GO:0002376 immune system process; GO:0002520 immune system development; GO:0006139 nucleobase-containing compound metabolic process; GO:0006259 DNA metabolic process; GO:0006275 regulation	GO:0000228 nuclear chromosome; GO:0000775 chromosome, centromeric region; GO:0000785 chromatin; GO:0000790 nuclear chromatin; GO:0000792 heterochromatin; GO:0000803 sex chromosome; GO:0001741 XY body; GO:0005622 intracellular;	GO:0003824 catalytic activity; GO:0004468 lysine N-acetyltransferase activity, acting on acetyl phosphate as donor; GO:0008080 N-acetyltransferase activity; GO:0016407 acetyltransferase activity; GO:0016410 N-acyltransferase activity; GO:0016740 transferase	PF13878; PF13880	zinc-finger of acetyl-transferase ESCO; ESCO1/2 acetyl-transferase	L	KOG3014	Protein involved in establishing cohesion between sister chromatids during DNA replication

P21580	Tumor necrosis factor alpha-induced protein 3 OS=Homo sapiens OX=9606 GN=TNFAIP3 PE=1 SV=1	TNFAIP3	1.318	0.003153434	Up	TNFAIP3, A20, OTUD7C; tumor necrosis factor, alpha-induced protein 3 [EC:3.4.19.12]	map04064 NF-kappa B signaling pathway; map04217 Necroptosis; map04621 NOD-like receptor signaling pathway; map04657 IL-17 signaling pathway; map04668 TNF signaling pathway; map05162 Measles; map05169 Epstein-Barr virus infection	GO:0000079 regulation of cyclin-dependent protein serine/threonine kinase activity; GO:0000209 protein polyubiquitination; GO:0000302 response to reactive oxygen species; GO:0001775 cell activation; GO:0001776 leukocyte homeostasis; GO:0001782 B cell homeostasi	GO:0000323 lytic vacuole; GO:0005622 intracellular; GO:0005623 cell; GO:0005634 nucleus; GO:0005737 cytoplasm; GO:0005764 lysosome; GO:0005773 vacuole; GO:0005829 cytosol; GO:0043226 organelle; GO:0043227 membrane-bounded organelle; GO:0043229	GO:0002020 protease binding; GO:0003824 catalytic activity; GO:0004842 ubiquitin-protein transferase activity; GO:0004843 thiol-dependent ubiquitin-specific protease activity; GO:0005488 binding; GO:0005515 protein binding; GO:0008233 peptidase activity; GO:0008234 cysteine-			K	KOG4345	NF-kappa B regulator AP20/Cezanne
Q66K64	DDB1- and CUL4-associated factor 15 OS=Homo sapiens OX=9606 GN=DCAF15 PE=1 SV=1	DCAF15	1.316	0.0112988	Up	DCAF15; DDB1- and CUL4-associated factor 15		GO:0006464 cellular protein modification process; GO:0006807 nitrogen compound metabolic process; GO:0008152 metabolic process; GO:0009987 cellular process; GO:0016567 protein ubiquitination; GO:0019538 protein metabolic process; GO:0032446 protein modification by small protein conjugatio	GO:0000151 ubiquitin ligase complex; GO:0005622 intracellular; GO:0005623 cell; GO:0031461 cullin-RING ubiquitin ligase complex; GO:0032991 macromolecular complex; GO:0044424 intracellular part; GO:0044464 cell part; GO:0080008 Cul4-						
Q9NRZ9	Lymphoid-specific helicase OS=Homo sapiens OX=9606 GN=HELLS PE=1 SV=1	HELLS	1.316	2.8087E-05	Up	HELLS, DDM1; ATP-dependent DNA helicase		GO:0001655 urogenital system development; GO:0001775 cell activation; GO:0002376 immune system process; GO:0002520 immune system development; GO:0002521 leukocyte differentiation; GO:0006139 nucleobase-containing compound metabolic process; GO:00062	GO:0000775 chromosome, centromeric region; GO:0000785 chromatin; GO:0000792 heterochromatin; GO:0005622 intracellular; GO:0005623 cell; GO:0005634 nucleus; GO:0005694 chromosome; GO:0005721 pericentric heterochromatin; GO:00432	GO:0003682 chromatin binding; GO:0005488 binding;			K	KOG0385	Chromatin remodeling complex WSTF-ISWI, small subunit
Q9Y283	Inversin OS=Homo sapiens OX=9606 GN=INVS PE=1 SV=2	INVS	1.315	0.001762137	Up	INVS; inversin	map04310 Wnt signaling pathway	GO:0001655 urogenital system development; GO:0001822 kidney development; GO:0003002 regionalization; GO:0003341 cilium movement; GO:0003351 epithelial cilium movement; GO:0006928 movement of cell or subcellular component; GO:0007017	GO:0005622 intracellular; GO:0005623 cell; GO:0005634 nucleus; GO:0005737 cytoplasm; GO:0005819 spindle; GO:0005856 cytoskeleton; GO:0005929 cilium; GO:0015630 microtubule cytoskeleton; GO:0016020 membrane; GO:0042995 cell projection;				S	KOG0504	FOG: Ankyrin repeat

P37840	Alpha-synuclein OS=Homo sapiens OX=9606 GN=SNCA PE=1 SV=1	SNCA	1.315	0.000731839	Up	SNCA; alpha-synuclein	map05010 Alzheimer disease; map05012 Parkinson disease	GO:0000122 negative regulation of transcription from RNA polymerase II promoter; GO:0001504 neurotransmitter uptake; GO:0001505 regulation of neurotransmitter levels; GO:0001774 microglial cell activation; GO:0001775 cell activation; GO:00019	GO:0000323 lytic vacuole; GO:0005576 extracellular region; GO:0005615 extracellular space; GO:0005622 intracellular; GO:0005623 cell; GO:0005634 nucleus; GO:0005635 nuclear envelope; GO:0005640 nuclear outer membrane; GO:0005737 cytoplasm; GO:0005739	GO:0000287 magnesium ion binding; GO:0001067 regulatory region nucleic acid binding; GO:0003676 nucleic acid binding; GO:0003677 DNA binding; GO:0003779 actin binding; GO:0003824 catalytic activity; GO:0004857 enzyme inhibitor activity; GO:0004866					
P51587	Breast cancer type 2 susceptibility protein OS=Homo sapiens OX=9606 GN=BRCA2 PE=1 SV=3	BRCA2	1.315	0.000154038	Up	BRCA2, FANCD1; breast cancer 2 susceptibility protein	map03440 Homologous recombination; map03460 Fanconi anemia pathway; map05200 Pathways in cancer; map05212 Pancreatic cancer; map05224 Breast cancer	GO:0000003 reproduction; GO:0000226 microtubule cytoskeleton organization; GO:0000278 mitotic cell cycle; GO:0000280 nuclear division; GO:0000281 mitotic cytokinesis; GO:0000722 telomere maintenance via recombination; GO:0000723	GO:0000228 nuclear chromosome; GO:0000781 chromosome, telomeric region; GO:0000784 nuclear chromosome, telomeric region; GO:0000793 condensed chromosome; GO:0000794 condensed nuclear chromosome; GO:0000795 synaptonemal	GO:0002020 protease binding; GO:0003676 nucleic acid binding; GO:0003677 DNA binding; GO:0003697 single-stranded DNA binding; GO:0003824 catalytic activity; GO:0004402 histone acetyltransferase activity; GO:0005488 binding; GO:0005515 protein binding; GO:0008022 protein C-terminus	PF09169; PF09103; PF09121; PF09104	BRCA2, helical; BRCA2, oligonucleotide/oligosaccharide-binding, domain 1; Tower; BRCA2, oligonucleotide/oligosaccharide-binding, domain 3	L	KOG4751	DNA recombinational repair protein BRCA2
Q86T65	Disheveled-associated activator of morphogenesis 2 OS=Homo sapiens OX=9606 GN=DAAM2 PE=1 SV=3	DAAM2	1.312	0.03939052	Up	DAAM; dishevelled associated activator of morphogenesis	map04310 Wnt signaling pathway	GO:0007275 multicellular organism development; GO:0007399 nervous system development; GO:0007417 central nervous system development; GO:0009966 regulation of signal transduction; GO:0009967 positive regulation of signal transduction; GO:00099					TZ	KOG1922	Rho GTPase effector BNI1 and related formins
P51159	Ras-related protein Rab-27A OS=Homo sapiens OX=9606 GN=RAB27A PE=1 SV=3	RAB27A	1.311	0.010003583	Up	RAB27A; Ras-related protein Rab-27A		GO:0001775 cell activation; GO:0001906 cell killing; GO:0001909 leukocyte mediated cytotoxicity; GO:0001913 T cell mediated cytotoxicity; GO:0002228 natural killer cell mediated immunity; GO:0002250 adaptive immune response; GO:0002252 immune effector process; GO:00022	GO:0000323 lytic vacuole; GO:0001750 photoreceptor outer segment; GO:0005576 extracellular region; GO:0005622 intracellular; GO:0005623 cell; GO:0005737 cytoplasm; GO:0005764 lysosome; GO:0005768 endosome; GO:0005770 late endosome; GO:00057	GO:0000166 nucleotide binding; GO:0001882 nucleoside binding; GO:0001883 purine nucleoside binding; GO:0003824 catalytic activity; GO:0003924 GTPase activity; GO:0005488 binding; GO:0005515 protein binding; GO:0005525 GTP binding; GO:0008092 cytoskeletal protein binding;	PF00071	Ras family	U	KOG0081	GTPase Rab27, small G protein superfamily

P23276	Kell blood group glycoprotein OS=Homo sapiens OX=9606 GN=KEL PE=1 SV=2	KEL	1.305	0.025787539	Up	KEL, CD238; Kell blood group glycoprotein [EC:3.4.24.-]		GO:0003008 system process; GO:0003013 circulatory system process; GO:0003018 vascular process in circulatory system; GO:0006508 proteolysis; GO:0006807 nitrogen compound metabolic process; GO:0006873 cellular ion homeostasis; GO:0006874 cellular calcium ion homeostasis;	GO:0005623 cell; GO:0005886 plasma membrane; GO:0016020 membrane; GO:0016021 integral component of membrane; GO:0031224 intrinsic component of membrane; GO:0044425 membrane part; GO:0044464 cell part; GO:0071944 cell periphery;	GO:0003824 catalytic activity; GO:0004175 endopeptidase activity; GO:0004222 metalloendopeptidase activity; GO:0008233 peptidase activity; GO:0008237 metallopeptidase activity; GO:0016787 hydrolase activity; GO:0070011 peptidase activity, acting on			E	KOG3624	M13 family peptidase
Q6ZN17	Protein lin-28 homolog B OS=Homo sapiens OX=9606 GN=LIN28B PE=1 SV=1	LIN28B	1.303	0.00017157	Up	LIN28; protein lin-28		GO:0006139 nucleobase-containing compound metabolic process; GO:0006396 RNA processing; GO:0006401 RNA catabolic process; GO:0006725 cellular aromatic compound metabolic process; GO:0006807 nitrogen compound metabolic process; GO:0008152 metabolic process; GO:0009056	GO:0005622 intracellular; GO:0005623 cell; GO:0005634 nucleus; GO:0005730 nucleolus; GO:0005737 cytoplasm; GO:0005829 cytosol; GO:0031974 membrane-enclosed lumen; GO:0031981 nuclear lumen; GO:0043226 organelle; GO:0043227 membrane-bounded	GO:0003676 nucleic acid binding; GO:0003723 RNA binding; GO:0005488 binding; GO:0097159 organic cyclic compound binding; GO:1901363 heterocyclic compound binding;	PF00313; PF00098	'Cold-shock' DNA-binding domain; Zinc knuckle	J	KOG3070	Predicted RNA-binding protein containing PIN domain and invovled in translation or RNA processing
P23297	Protein S100-A1 OS=Homo sapiens OX=9606 GN=S100A1 PE=1 SV=2	S100A1	1.302	0.002439233	Up			GO:0000122 negative regulation of transcription from RNA polymerase II promoter; GO:0002218 activation of innate immune response; GO:0002221 pattern recognition receptor signaling pathway; GO:0002224 toll-like receptor signaling pathway; GO:0002253 activation of immune	GO:0005576 extracellular region; GO:0005622 intracellular; GO:0005623 cell; GO:0005634 nucleus; GO:0005737 cytoplasm; GO:0005783 endoplasmic reticulum; GO:0005829 cytosol; GO:0012505 endomembrane system; GO:0016528 sarcoplasm;	GO:0005488 binding; GO:0005509 calcium ion binding; GO:0005515 protein binding; GO:0019899 enzyme binding; GO:0042802 identical protein binding; GO:0042803 protein homodimerization activity; GO:0043167 ion binding; GO:0043169 cation binding; GO:0044548 S100 protein	PF01023; PF00036	S-100/ICaBP type calcium binding domain; EF hand			
Q9Y6U3	Adseverin OS=Homo sapiens OX=9606 GN=SCIN PE=1 SV=4	SCIN	1.301	0.002386513	Up	GSN; gelsolin	map04666 Fc gamma R-mediated phagocytosis; map04810 Regulation of actin cytoskeleton; map05203 Viral carcinogenesis	GO:0002682 regulation of immune system process; GO:0002684 positive regulation of immune system process; GO:0006810 transport; GO:0006887 exocytosis; GO:0006996 organelle organization; GO:0007010 cytoskeleton organization; GO:0007015 actin	GO:0005622 intracellular; GO:0005623 cell; GO:0005737 cytoplasm; GO:0005886 plasma membrane; GO:0005903 brush border; GO:0005938 cell cortex; GO:0016020 membrane; GO:0032991 macromolecular complex; GO:0044424 intracellular part; GO:00444	GO:0001786 phosphatidylserine binding; GO:0003779 actin binding; GO:0005488 binding; GO:0005509 calcium ion binding; GO:0005515 protein binding; GO:0005543 phospholipid binding; GO:0005545 1-phosphatidylinositol binding; GO:0005546 phosphatidylinositol-4,5-	PF00626	Gelsolin repeat	Z	KOG0443	Actin regulatory proteins (gelsolin/villin family)
Q8IXQ4	GPALPP motifs-containing protein 1 OS=Homo sapiens OX=9606 GN=GPALPP1 PE=1 SV=1	GPALPP1	0.768	0.015302938	Down								S	KOG4188	Uncharacterized conserved protein

Q9Y4K0	Lysyl oxidase homolog 2 OS=Homo sapiens OX=9606 GN=LOXL2 PE=1 SV=1	LOXL2	0.767	0.000489862	Down	LOXL2_3_4; lysyl oxidase-like protein 2/3/4 [EC:1.4.3.-]		GO:0000122 negative regulation of transcription from RNA polymerase II promoter; GO:0001525 angiogenesis; GO:0001568 blood vessel development; GO:0001666 response to hypoxia; GO:0001667 ameboidal-type cell migration; GO:0001837 epithelial	GO:0000785 chromatin; GO:0005576 extracellular region; GO:0005604 basement membrane; GO:0005615 extracellular space; GO:0005622 intracellular; GO:0005623 cell; GO:0005634 nucleus; GO:0005654 nucleoplasm; GO:0005694 chromosome;	GO:0003824 catalytic activity; GO:0004720 protein-lysine 6-oxidase activity; GO:0005488 binding; GO:0005507 copper ion binding; GO:0005509 calcium ion binding; GO:0009055 electron carrier activity; GO:0016491 oxidoreductase activity; GO:0016638 oxidoreductase	PF00530	Scavenger receptor cysteine-rich domain			
Q96CS7	Pleckstrin homology domain-containing family B member 2 OS=Homo sapiens OX=9606 GN=PLEKHB2 PE=1 SV=1	PLEKHB2	0.767	0.049299851	Down			GO:0045595 regulation of cell differentiation; GO:0050789 regulation of biological process; GO:0050793 regulation of developmental process; GO:0050794 regulation of cellular process; GO:0065007 biological regulation;	GO:0016020 membrane; GO:0016021 integral component of membrane; GO:0031224 intrinsic component of membrane; GO:0044425 membrane part;	GO:0005488 binding; GO:0005543 phospholipid binding; GO:0005547 phosphatidylinositol-3,4,5-trisphosphate binding; GO:0008289 lipid binding; GO:0035091 phosphatidylinositol binding; GO:0043167 ion binding; GO:0043168 anion binding; GO:1901981 phosphatidylinositol	PF00169	PH domain			
P35568	Insulin receptor substrate 1 OS=Homo sapiens OX=9606 GN=IRS1 PE=1 SV=1	IRS1	0.766	0.001143416	Down	IRS1; insulin receptor substrate 1	map04022 cGMP-PKG signaling pathway; map04068 FoxO signaling pathway; map04140 Autophagy - animal; map04150 mTOR signaling pathway; map04151 PI3K-Akt signaling pathway; map04152 AMPK signaling pathway; map04211 Longevity regulating pathway; map04212 Longevity regulating pathway - worm; map04213 Longevity regulating pathway - multiple species; map04722 Neurotrophin signaling pathway; map04910 Insulin signaling pathway; map04920 Adipocytokine signaling pathway; map04923 Regulation of lipolysis in adipocytes; map04930 Type II diabetes mellitus; map04931 Insulin resistance; map04932 Non-alcoholic fatty liver disease (NAFLD); map04935 Growth hormone synthesis, secretion and action; map04960 Aldosterone-regulated sodium reabsorption; map05206 MicroRNAs in cancer	GO:0000165 MAPK cascade; GO:0002053 positive regulation of mesenchymal cell proliferation; GO:0002791 regulation of peptide secretion; GO:0002792 negative regulation of peptide secretion; GO:0002793 positive regulation of peptide secretion; GO:0005979 regulation of glycogen	GO:0005622 intracellular; GO:0005623 cell; GO:0005634 nucleus; GO:0005737 cytoplasm; GO:0005815 microtubule organizing center; GO:0005829 cytosol; GO:0005856 cytoskeleton; GO:0005886 plasma membrane; GO:0005887 integral component of plasma membrane;	GO:0001784 phosphotyrosine binding; GO:0003824 catalytic activity; GO:0005068 transmembrane receptor protein tyrosine kinase adaptor activity; GO:0005080 protein kinase C binding; GO:0005085 guanyl-nucleotide exchange factor activity; GO:0005088 Ras guanyl-	PF00169; PF02174	PH domain; PTB domain (IRS-1 type)			
Q9NWM0	Spermine oxidase OS=Homo sapiens OX=9606 GN=SMOX PE=1 SV=1	SMOX	0.764	0.000437	Down	SMOX, PAO5; spermine oxidase [EC:1.5.3.16 1.5.3.-]	map00330 Arginine and proline metabolism; map00410 beta-Alanine metabolism; map01100 Metabolic pathways	GO:0006576 cellular biogenic amine metabolic process; GO:0006595 polyamine metabolic process; GO:0006596 polyamine biosynthetic process; GO:0006598 polyamine catabolic process; GO:0006807 nitrogen compound metabolic process; GO:0008152 metabolic process; GO:00082	GO:0005622 intracellular; GO:0005623 cell; GO:0005634 nucleus; GO:0005635 nuclear envelope; GO:0005654 nucleoplasm; GO:0005737 cytoplasm; GO:0005829 cytosol; GO:0012505 endomembrane system; GO:0016020 membrane; GO:0031090 organelle	GO:0003824 catalytic activity; GO:0016491 oxidoreductase activity; GO:0016645 oxidoreductase activity, acting on the CH-NH group of donors; GO:0016647 oxidoreductase activity, acting on the CH-NH group of donors, oxygen as acceptor; GO:0046592 polyamine	PF01593	Flavin containing amine oxidoreductase	H	KOG0685	Flavin-containing amine oxidase

P09038	Fibroblast growth factor 2 OS=Homo sapiens OX=9606 GN=FGF2 PE=1 SV=3	FGF2	0.763	2.92275E-06	Down	FGF2; fibroblast growth factor 2	map01521 EGFR tyrosine kinase inhibitor resistance; map04010 MAPK signaling pathway; map04014 Ras signaling pathway; map04015 Rap1 signaling pathway; map04151 PI3K-Akt signaling pathway; map04550 Signaling pathways regulating pluripotency of stem cells; map04810 Regulation of actin cytoskeleton; map05167 Kaposi sarcoma-associated herpesvirus infection; map05200 Pathways in cancer; map05205 Proteoglycans in cancer; map05218 Melanoma; map05224 Breast cancer; map05226 Gastric cancer	GO:0000165 MAPK cascade; GO:0000186 activation of MAPKK activity; GO:0000187 activation of MAPK activity; GO:0001501 skeletal system development; GO:0001525 angiogenesis; GO:0001568 blood vessel development; GO:0001655 urogenital system	GO:0005576 extracellular region; GO:0005615 extracellular space; GO:0005622 intracellular; GO:0005623 cell; GO:0005634 nucleus; GO:0005737 cytoplasm; GO:0043226 organelle; GO:0043227 membrane-bounded organelle; GO:0043229 intracellular organelle;	GO:0003712 transcription cofactor activity; GO:0003713 transcription coactivator activity; GO:0003824 catalytic activity; GO:0004672 protein kinase activity; GO:0004713 protein tyrosine kinase activity; GO:0005085 guanyl-nucleotide exchange factor activity; GO:0005088 Ras	PF00167	Fibroblast growth factor	K	KOG3885	Fibroblast growth factor
P24385	G1/S-specific cyclin-D1 OS=Homo sapiens OX=9606 GN=CCND1 PE=1 SV=1	CCND1	0.763	0.00441583	Down	CCND1; G1/S-specific cyclin-D1	map01522 Endocrine resistance; map04068 FoxO signaling pathway; map04110 Cell cycle; map04115 p53 signaling pathway; map04151 PI3K-Akt signaling pathway; map04152 AMPK signaling pathway; map04218 Cellular senescence; map04310 Wnt signaling pathway; map04340 Hedgehog signaling pathway; map04371 Apelin signaling pathway; map04390 Hippo signaling pathway; map04510 Focal adhesion; map04530 Tight junction; map04630 JAK-STAT signaling pathway; map04917 Prolactin signaling pathway; map04919 Thyroid hormone signaling pathway; map04921 Oxytocin signaling pathway; map04933 AGE-RAGE signaling pathway in diabetic complications; map04934 Cushing syndrome; map05160 Hepatitis C; map05162 Measles; map05163 Human cytomegalovirus infection; map05165 Human papillomavirus infection; map05166 Human T-cell leukemia virus 1 infection; map05167 Kaposi sarcoma-associated herpesvirus infection; map05169 Epstein-Barr virus infection; map05200 Pathways in cancer; map05203 Viral carcinogenesis; map05205 Proteoglycans in cancer; map05206 MicroRNAs in cancer; map05210 Colorectal cancer; map05212 Pancreatic cancer; map05213 Endometrial cancer; map05214 Glioma; map05215 Prostate cancer; map05216 Thyroid cancer; map05218 Melanoma; map05219 Bladder cancer; map05220 Chronic myeloid leukemia; map05221 Acute myeloid leukemia; map05222 Small cell lung cancer; map05223 Non-small cell lung cancer; map05224	GO:0000003 reproduction; GO:0000075 cell cycle checkpoint; GO:0000077 DNA damage checkpoint; GO:0000079 regulation of cyclin-dependent protein serine/threonine kinase activity; GO:0000082 G1/S transition of mitotic cell cycle; GO:0000122 negative regulation of	GO:0000307 cyclin-dependent protein kinase holoenzyme complex; GO:0005622 intracellular; GO:0005623 cell; GO:0005634 nucleus; GO:0005654 nucleoplasm; GO:0005737 cytoplasm; GO:0005829 cytosol; GO:0017053 transcriptional repressor complex; GO:00319	GO:0003712 transcription cofactor activity; GO:0003714 transcription corepressor activity; GO:0003824 catalytic activity; GO:0004672 protein kinase activity; GO:0004674 protein serine/threonine kinase activity; GO:0004693 cyclin-dependent protein serine/threonine kinase	PF00134; PF02984	Cyclin, N-terminal domain; Cyclin, C-terminal domain	K	KOG0656	G1/S-specific cyclin D
Q9NR12	PDZ and LIM domain protein 7 OS=Homo sapiens OX=9606 GN=PDLIM7 PE=1 SV=1	PDLIM7	0.762	0.000162596	Down	LDB3; LIM domain-binding protein 3		GO:0000902 cell morphogenesis; GO:0000904 cell morphogenesis involved in differentiation; GO:0006810 transport; GO:0006897 endocytosis; GO:0006898 receptor-mediated endocytosis; GO:0006928 movement of cell or subcellular component;	GO:0001725 stress fiber; GO:0001726 ruffle; GO:0005622 intracellular; GO:0005623 cell; GO:0005634 nucleus; GO:0005737 cytoplasm; GO:0005829 cytosol; GO:0005856 cytoskeleton; GO:0005911 cell-cell junction; GO:0005912 adherens junction; GO:0005913 cell-cell		PF00595; PF00412	PDZ domain (Also known as DHR or GLGF); LIM domain	TZ	KOG1703	Adaptor protein Enigma and related PDZ-LIM proteins
P09493	Tropomyosin alpha-1 chain OS=Homo sapiens OX=9606 GN=TPM1 PE=1 SV=2	TPM1	0.761	0.001145264	Down	TPM1; tropomyosin 1	map04260 Cardiac muscle contraction; map04261 Adrenergic signaling in cardiomyocytes; map05206 MicroRNAs in cancer; map05410 Hypertrophic cardiomyopathy (HCM); map05414 Dilated cardiomyopathy (DCM)	GO:0000302 response to reactive oxygen species; GO:0001701 in utero embryonic development; GO:0001993 regulation of systemic arterial blood pressure by norepinephrine-epinephrine; GO:0001996 positive regulation of heart rate by epinephrine-norepinephrine;	GO:0001725 stress fiber; GO:0001726 ruffle; GO:0005622 intracellular; GO:0005623 cell; GO:0005737 cytoplasm; GO:0005829 cytosol; GO:0005856 cytoskeleton; GO:0005862 muscle thin filament tropomyosin; GO:0005865 striated muscle thin filament; GO:00058	GO:0003779 actin binding; GO:0005198 structural molecule activity; GO:0005200 structural constituent of cytoskeleton; GO:0005488 binding; GO:0005515 protein binding; GO:0008092 cytoskeletal protein binding; GO:0008307 structural constituent of muscle; GO:00199			Z	KOG1003	Actin filament-coating protein tropomyosin

P42766	60S ribosomal protein L35 OS=Homo sapiens OX=9606 GN=RPL35 PE=1 SV=2	RPL35	0.758	8.55576E-05	Down	RP-L35e, RPL35; large subunit ribosomal protein L35e	map03010 Ribosome	GO:0000184 nuclear-transcribed mRNA catabolic process, nonsense-mediated decay; GO:0000463 maturation of LSU-rRNA from tricistronic rRNA transcript (SSU-rRNA, 5.8S rRNA, LSU-rRNA); GO:0000470 maturation of LSU-rRNA; GO:0000956 nuclear-transcribed	GO:0005622 intracellular; GO:0005623 cell; GO:0005634 nucleus; GO:0005730 nucleolus; GO:0005737 cytoplasm; GO:0005829 cytosol; GO:0005840 ribosome; GO:0015934 large ribosomal subunit; GO:0022625 cytosolic large ribosomal subunit; GO:0022626	GO:0003676 nucleic acid binding; GO:0003723 RNA binding; GO:0003729 mRNA binding; GO:0003735 structural constituent of ribosome; GO:0005198 structural molecule activity; GO:0005488 binding; GO:0043021 ribonucleoprotein complex binding; GO:0044877			J	KOG3436	60S ribosomal protein L35
P00750	Tissue-type plasminogen activator OS=Homo sapiens OX=9606 GN=PLAT PE=1 SV=1	PLAT	0.757	0.001173643	Down	PLAT; tissue plasminogen activator [EC:3.4.21.68]	map04371 Apelin signaling pathway; map04610 Complement and coagulation cascades; map05202 Transcriptional misregulation in cancer; map05215 Prostate cancer; map05418 Fluid shear stress and atherosclerosis	GO:0001101 response to acid chemical; GO:0001666 response to hypoxia; GO:0006464 cellular protein modification process; GO:0006508 proteolysis; GO:0006807 nitrogen compound metabolic process; GO:0006928 movement of cell or subcellular component; GO:0006950	GO:0005576 extracellular region; GO:0005615 extracellular space; GO:0005622 intracellular; GO:0005623 cell; GO:0005737 cytoplasm; GO:0009986 cell surface; GO:0012505 endomembrane system; GO:0030141 secretory granule; GO:0031410	GO:0003824 catalytic activity; GO:0004175 endopeptidase activity; GO:0004252 serine-type endopeptidase activity; GO:0005102 receptor binding; GO:0005488 binding; GO:0005515 protein binding; GO:0008233 peptidase activity; GO:0008236 serine-type peptidase activity;	PF00039; PF00008; PF00051; PF00089	Fibronectin type I domain; EGF-like domain; Kringle domain; Trypsin	O	KOG3627	Trypsin
Q12791	Calcium-activated potassium channel subunit alpha-1 OS=Homo sapiens OX=9606 GN=KCNMA1 PE=1 SV=2	KCNMA1	0.753	0.002220593	Down	KCNMA1, KCA1.1; potassium large conductance calcium-activated channel subfamily M alpha member 1	map04022 cGMP-PKG signaling pathway; map04270 Vascular smooth muscle contraction; map04911 Insulin secretion; map04924 Renin secretion; map04970 Salivary secretion; map04972 Pancreatic secretion	GO:0001508 action potential; GO:0001666 response to hypoxia; GO:0002065 columnar/cuboidal epithelial cell differentiation; GO:0003008 system process; GO:0003012 muscle system process; GO:0003013 circulatory system process; GO:0003014 renal system process;	GO:0005576 extracellular region; GO:0005615 extracellular space; GO:0005622 intracellular; GO:0005623 cell; GO:0005737 cytoplasm; GO:0005783 endoplasmic reticulum; GO:0005886 plasma membrane; GO:0005887 integral component of plasma membrane; GO:00059	GO:0003779 actin binding; GO:0005215 transporter activity; GO:0005216 ion channel activity; GO:0005227 calcium activated cation channel activity; GO:0005244 voltage-gated ion channel activity; GO:0005249 voltage-gated potassium channel activity; GO:0005261 cation channel			P	KOG1420	Ca2+-activated K+ channel Slowpoke, alpha subunit
Q9BZQ8	Protein Niban 1 OS=Homo sapiens OX=9606 GN=NIBAN1 PE=1 SV=1	NIBAN1	0.753	3.05292E-05	Down			GO:0001932 regulation of protein phosphorylation; GO:0001933 negative regulation of protein phosphorylation; GO:0001934 positive regulation of protein phosphorylation; GO:0006417 regulation of translation; GO:0006950 response to stress; GO:0009889 regulation of	GO:0005622 intracellular; GO:0005623 cell; GO:0005737 cytoplasm; GO:0005829 cytosol; GO:0005886 plasma membrane; GO:0016020 membrane; GO:0044424 intracellular part; GO:0044444 cytoplasmic part; GO:0044464 cell part; GO:0071944 cell periphery;						

O14967	Calmegin OS=Homo sapiens OX=9606 GN=CLGN PE=1 SV=1	CLGN	0.75	0.00012228	Down	CLGN; calmegin		GO:0000003 reproduction; GO:0006457 protein folding; GO:0007338 single fertilization; GO:0007339 binding of sperm to zona pellucida; GO:0008037 cell recognition; GO:0009566 fertilization; GO:0009987 cellular process; GO:0009988 cell-cell recognition;	GO:0005622 intracellular; GO:0005623 cell; GO:0005634 nucleus; GO:0005635 nuclear envelope; GO:0005737 cytoplasm; GO:0005783 endoplasmic reticulum; GO:0005789 endoplasmic reticulum membrane; GO:0012505 endomembrane system; GO:0016020	GO:0005488 binding; GO:0005509 calcium ion binding; GO:0005515 protein binding; GO:0043167 ion binding; GO:0043169 cation binding; GO:0044183 protein binding involved in protein folding; GO:0046872 metal ion binding; GO:0051082 unfolded protein binding;			O	KOG0675	Calnexin
P84243	Histone H3.3 OS=Homo sapiens OX=9606 GN=H3-3A PE=1 SV=2	H3-3A	0.749	0.000247761	Down	H3; histone H3	map05034 Alcoholism; map05131 Shigellosis; map05202 Transcriptional misregulation in cancer; map05322 Systemic lupus erythematosus				PF00125	Core histone H2A/H2B/H3/H4	B	KOG1745	Histones H3 and H4
Q8N961	Ankyrin repeat and BTB/POZ domain-containing protein 2 OS=Homo sapiens OX=9606 GN=ABTB2 PE=2 SV=2	ABTB2	0.744	0.000968484	Down	ABTB2; ankyrin repeat and BTB/POZ domain-containing protein 2		GO:0006508 proteolysis; GO:0006511 ubiquitin-dependent protein catabolic process; GO:0006807 nitrogen compound metabolic process; GO:0008152 metabolic process; GO:0009056 catabolic process; GO:0009057 macromolecule catabolic process; GO:0009636	GO:0000151 ubiquitin ligase complex; GO:0005622 intracellular; GO:0005623 cell; GO:0005634 nucleus; GO:0005654 nucleoplasm; GO:0005737 cytoplasm; GO:0019005 SCF ubiquitin ligase complex; GO:0031461 cullin-RING ubiquitin ligase complex;	GO:0005488 binding; GO:0005515 protein binding; GO:0019899 enzyme binding; GO:0031625 ubiquitin protein ligase binding; GO:0044389 ubiquitin-like protein ligase binding;	PF00651	BTB/POZ domain			
P54687	Branched-chain-amino-acid aminotransferase, cytosolic OS=Homo sapiens OX=9606 GN=BCAT1 PE=1 SV=3	BCAT1	0.74	0.000367333	Down	E2.6.1.42, ilvE; branched-chain amino acid aminotransferase [EC:2.6.1.42]	map00270 Cysteine and methionine metabolism; map00280 Valine, leucine and isoleucine degradation; map00290 Valine, leucine and isoleucine biosynthesis; map00770 Pantothenate and CoA biosynthesis; map01100 Metabolic pathways; map01210 2-Oxocarboxylic acid metabolism; map01230 Biosynthesis of amino acids	GO:0000082 G1/S transition of mitotic cell cycle; GO:0000278 mitotic cell cycle; GO:0006082 organic acid metabolic process; GO:0006520 cellular amino acid metabolic process; GO:0006551 leucine metabolic process; GO:0006573 valine metabolic process; GO:0006807 nitrogen compound metabolic process;	GO:0005622 intracellular; GO:0005623 cell; GO:0005737 cytoplasm; GO:0005739 mitochondrion; GO:0005829 cytosol; GO:0043226 organelle; GO:0043227 membrane-bounded organelle; GO:0043229 intracellular organelle; GO:0043231 intracellular	GO:0003824 catalytic activity; GO:0004084 branched-chain-amino-acid transaminase activity; GO:0005488 binding; GO:0005515 protein binding; GO:0008483 transaminase activity; GO:0016740 transferase activity; GO:0016769 transferase activity, transferring nitrogenous groups; GO:00428	PF01063	Aminotransferase class IV	E	KOG0975	Branched chain aminotransferase BCAT1, pyridoxal phosphate enzymes type IV superfamily
Q9UBK2	Peroxisome proliferator-activated receptor gamma coactivator 1-alpha OS=Homo sapiens OX=9606 GN=PPARGC1A PE=1 SV=1	PPARGC1A	0.739	0.00010333	Down	PPARGC1A, PGC1A; alpha peroxisome proliferator-activated receptor gamma coactivator 1-alpha	map04152 AMPK signaling pathway; map04211 Longevity regulating pathway; map04371 Apelin signaling pathway; map04714 Thermogenesis; map04910 Insulin signaling pathway; map04920 Adipocytokine signaling pathway; map04922 Glucagon signaling pathway; map04931 Insulin resistance; map05016 Huntington disease	GO:0000302 response to reactive oxygen species; GO:0000422 mitophagy; GO:0001101 response to acid chemical; GO:0001659 temperature homeostasis; GO:0001666 response to hypoxia; GO:0001678 cellular glucose homeostasis; GO:0001932	GO:0000228 nuclear chromosome; GO:0000428 DNA-directed RNA polymerase complex; GO:0000785 chromatin; GO:0000790 nuclear chromatin; GO:0000791 euchromatin; GO:0005622 intracellular; GO:0005623 cell; GO:0005634 nucleus; GO:0005654 nucleoplas	GO:0003676 nucleic acid binding; GO:0003677 DNA binding; GO:0003682 chromatin binding; GO:0003712 transcription cofactor activity; GO:0003713 transcription coactivator activity; GO:0003723 RNA binding; GO:0005102 receptor binding; GO:0005488 binding; GO:00055	PF00076	RNA recognition motif. (a.k.a. RRM, RBD, or RNP domain)			

P17302	Gap junction alpha-1 protein OS=Homo sapiens OX=9606 GN=GJA1 PE=1 SV=2	GJA1	0.738	0.000321727	Down	GJA1, CX43; gap junction alpha-1 protein	map04540 Gap junction; map05131 Shigellosis; map05412 Arrhythmogenic right ventricular cardiomyopathy (ARVC)	GO:0000003 reproduction; GO:0000902 cell morphogenesis; GO:0001101 response to acid chemical; GO:0001503 ossification; GO:0001508 action potential; GO:0001558 regulation of cell growth; GO:0001568 blood vessel development; GO:00016	GO:0000139 Golgi membrane; GO:0000323 lytic vacuole; GO:0005622 intracellular; GO:0005623 cell; GO:0005634 nucleus; GO:0005654 nucleoplasm; GO:0005737 cytoplasm; GO:0005739 mitochondrion; GO:0005740 mitochondrial envelope; GO:00057	GO:0005102 receptor binding; GO:0005215 transporter activity; GO:0005243 gap junction channel activity; GO:0005488 binding; GO:0005515 protein binding; GO:0008092 cytoskeletal protein binding; GO:0015075 ion transmembrane transporter activity; GO:0015267 channel activity;					
P36969	Phospholipid hydroperoxide glutathione peroxidase OS=Homo sapiens OX=9606 GN=GPX4 PE=1 SV=3	GPX4	0.735	0.000170344	Down	GPX4; phospholipid-hydroperoxide glutathione peroxidase [EC:1.11.1.12]	map00480 Glutathione metabolism; map01100 Metabolic pathways; map04216 Ferroptosis	GO:0000003 reproduction; GO:0001676 long-chain fatty acid metabolic process; GO:0006082 organic acid metabolic process; GO:0006325 chromatin organization; GO:0006518 peptide metabolic process; GO:0006575 cellular modified amino acid metabolic process; GO:00066	GO:0005622 intracellular; GO:0005623 cell; GO:0005634 nucleus; GO:0005635 nuclear envelope; GO:0005654 nucleoplasm; GO:0005737 cytoplasm; GO:0005739 mitochondrion; GO:0005829 cytosol; GO:0012505 endomembrane system; GO:0031967	GO:0003824 catalytic activity; GO:0004601 peroxidase activity; GO:0004602 glutathione peroxidase activity; GO:0005488 binding; GO:0005515 protein binding; GO:0008430 selenium binding; GO:0016209 antioxidant activity; GO:0016491 oxidoreductase activity; GO:00166			O	KOG1651	Glutathione peroxidase
Q8WXK1	Ankyrin repeat and SOCS box protein 15 OS=Homo sapiens OX=9606 GN=ASB15 PE=1 SV=3	ASB15	0.729	0.000517826	Down	ASB15; ankyrin repeat and SOCS box protein 15		GO:0006464 cellular protein modification process; GO:0006807 nitrogen compound metabolic process; GO:0008152 metabolic process; GO:0009987 cellular process; GO:0016567 protein ubiquitination; GO:0019538 protein metabolic process; GO:0032446 protein modification by small protein conjugatio	GO:0000151 ubiquitin ligase complex; GO:0005622 intracellular; GO:0005623 cell; GO:0005634 nucleus; GO:0005737 cytoplasm; GO:0005829 cytosol; GO:0032991 macromolecular complex; GO:0043226 organelle; GO:0043227 membrane-bounded organelle;	GO:0003824 catalytic activity; GO:0004842 ubiquitin-protein transferase activity; GO:0005488 binding; GO:0005515 protein binding; GO:0016740 transferase activity; GO:0019787 ubiquitin-like protein transferase activity; GO:0019899 enzyme binding; GO:0031625 ubiquitin protein	PF07525	SOCS box	S	KOG0504	FOG: Ankyrin repeat
Q8IZW8	Tensin-4 OS=Homo sapiens OX=9606 GN=TNS4 PE=1 SV=3	TNS4	0.724	0.000144962	Down	TNS; tensin		GO:0008104 protein localization; GO:0033036 macromolecule localization; GO:0051179 localization;	GO:0005622 intracellular; GO:0005623 cell; GO:0005737 cytoplasm; GO:0005829 cytosol; GO:0005912 adherens junction; GO:0005924 cell-substrate adherens junction; GO:0005925 focal adhesion; GO:0030054 cell junction; GO:0030055 cell-substrate junction; GO:00444		PF00017	SH2 domain	TZ	KOG1930	Focal adhesion protein Tensin, contains PTB domain

P07093	Glia-derived nexin OS=Homo sapiens OX=9606 GN=SERPINE2 PE=1 SV=1	SERPINE2	0.721	0.000266524	Down	SERPINE2; glia-derived nexin		GO:0000003 reproduction; GO:0001558 regulation of cell growth; GO:0002028 regulation of sodium ion transport; GO:0003006 developmental process involved in reproduction; GO:0003008 system process; GO:0006810 transport; GO:0006950	GO:0005576 extracellular region; GO:0005615 extracellular space; GO:0005622 intracellular; GO:0005623 cell; GO:0005737 cytoplasm; GO:0005829 cytosol; GO:0005886 plasma membrane; GO:0009897 external side of plasma membrane; GO:0009986 cell surface; GO:00125	GO:0004857 enzyme inhibitor activity; GO:0004866 endopeptidase inhibitor activity; GO:0004867 serine-type endopeptidase inhibitor activity; GO:0005102 receptor binding; GO:0005488 binding; GO:0005515 protein binding; GO:0005539 glycosaminoglycan binding; GO:00082	PF00079	Serpin (serine protease inhibitor)	V	KOG2392	Serpin
Q8IXZ2	Zinc finger CCCH domain-containing protein 3 OS=Homo sapiens OX=9606 GN=ZC3H3 PE=1 SV=3	ZC3H3	0.721	0.009743318	Down			GO:0006139 nucleobase-containing compound metabolic process; GO:0006355 regulation of transcription, DNA-templated; GO:0006357 regulation of transcription from RNA polymerase II promoter; GO:0006378 mRNA polyadenylation; GO:0006396 RNA processing;	GO:0005622 intracellular; GO:0005623 cell; GO:0005634 nucleus; GO:0005654 nucleoplasm; GO:0005847 mRNA cleavage and polyadenylation specificity factor complex; GO:0005849 mRNA cleavage factor complex; GO:0031974 membrane-enclosed lumen;	GO:0000981 RNA polymerase II transcription factor activity, sequence-specific DNA binding; GO:0003676 nucleic acid binding; GO:0003700 transcription factor activity, sequence-specific DNA binding; GO:0003723 RNA binding; GO:0003824 catalytic activity; GO:00045			K	KOG1492	C3H1-type Zn-finger protein
Q8TCB7	tRNA N(3)-methylcytidine methyltransferase METTL6 OS=Homo sapiens OX=9606 GN=METTL6 PE=1 SV=2	METTL6	0.718	0.001447015	Down	METTL6; methyltransferase-like protein 6 [EC:2.1.1.-]		GO:0001510 RNA methylation; GO:0002946 tRNA C5-cytosine methylation; GO:0006139 nucleobase-containing compound metabolic process; GO:0006396 RNA processing; GO:0006399 tRNA metabolic process; GO:0006400 tRNA modification; GO:0006725 cellular aromatic		GO:0003824 catalytic activity; GO:0008168 methyltransferase activity; GO:0008173 RNA methyltransferase activity; GO:0008175 tRNA methyltransferase activity; GO:0008757 S-adenosylmethionine-dependent methyltransferase activity; GO:0016427 tRNA (cytosine) methyltransferase	PF08242	Methyltransferase domain	S	KOG2361	Predicted methyltransferase
Q96ME7	Zinc finger protein 512 OS=Homo sapiens OX=9606 GN=ZNF512 PE=1 SV=2	ZNF512	0.717	0.005552252	Down			GO:0006355 regulation of transcription, DNA-templated; GO:0006357 regulation of transcription from RNA polymerase II promoter; GO:0009889 regulation of biosynthetic process; GO:0010468 regulation of gene expression; GO:0010556 regulation	GO:0005622 intracellular; GO:0005623 cell; GO:0005634 nucleus; GO:0043226 organelle; GO:0043227 membrane-bounded organelle; GO:0043229 intracellular organelle; GO:0043231 intracellular membrane-bounded organelle; GO:0044424 intracellular part;	GO:0000981 RNA polymerase II transcription factor activity, sequence-specific DNA binding; GO:0003700 transcription factor activity, sequence-specific DNA binding;	PF00096	Zinc finger, C2H2 type	K	KOG1721	FOG: Zn-finger

Q6PK04	Coiled-coil domain-containing protein 137 OS=Homo sapiens OX=9606 GN=CCDC137 PE=1 SV=1	CCDC137	0.716	8.54197E-06	Down				GO:0001650 fibrillar center; GO:0005622 intracellular; GO:0005623 cell; GO:0005634 nucleus; GO:0005694 chromosome; GO:0005730 nucleolus; GO:0031974 membrane-enclosed lumen; GO:0031981 nuclear lumen; GO:0043226 organelle; GO:0043227						
P07204	Thrombomodulin OS=Homo sapiens OX=9606 GN=THBD PE=1 SV=2	THBD	0.716	0.000281238	Down	THBD, CD141; thrombomodulin	map04610 Complement and coagulation cascades; map04933 AGE-RAGE signaling pathway in diabetic complications; map05418 Fluid shear stress and atherosclerosis	GO:0000003 reproduction; GO:0006950 response to stress; GO:0007154 cell communication; GO:0007165 signal transduction; GO:0007565 female pregnancy; GO:0007596 blood coagulation; GO:0007599 hemostasis; GO:0009611 response to wounding; GO:00099	GO:0005576 extracellular region; GO:0005615 extracellular space; GO:0005622 intracellular; GO:0005623 cell; GO:0005737 cytoplasm; GO:0005773 vacuole; GO:0005774 vacuolar membrane; GO:0005886 plasma membrane; GO:0005887 integral component of plasma membrane;	GO:0005488 binding; GO:0005509 calcium ion binding; GO:0038023 signaling receptor activity; GO:0043167 ion binding; GO:0043169 cation binding; GO:0046872 metal ion binding; GO:0060089 molecular transducer activity;	PF00059; PF12662; PF09064; PF07645	Lectin C-type domain; Complement Clr-like EGF-like; Thrombomodulin like fifth domain, EGF-like; Calcium-binding EGF domain			
P01011	Alpha-1-antichymotrypsin OS=Homo sapiens OX=9606 GN=SERPINA3 PE=1 SV=2	SERPINA3	0.715	0.000476191	Down	SERPINA; serpin A		GO:0001775 cell activation; GO:0001894 tissue homeostasis; GO:0002237 response to molecule of bacterial origin; GO:0002252 immune effector process; GO:0002263 cell activation involved in immune response; GO:0002274 myeloid leukocyte activation; GO:0002275 myeloid cell activation	GO:0000323 lytic vacuole; GO:0005576 extracellular region; GO:0005615 extracellular space; GO:0005622 intracellular; GO:0005623 cell; GO:0005634 nucleus; GO:0005737 cytoplasm; GO:0005764 lysosome; GO:0005766 primary lysosome; GO:0005773 vacuole; GO:00057	GO:0003676 nucleic acid binding; GO:0003677 DNA binding; GO:0004857 enzyme inhibitor activity; GO:0004866 endopeptidase inhibitor activity; GO:0004867 serine-type endopeptidase inhibitor activity; GO:0004869 cysteine-type endopeptidase inhibitor	PF00079	Serpin (serine protease inhibitor)	V	KOG2392	Serpin
Q71DI3	Histone H3.2 OS=Homo sapiens OX=9606 GN=H3C15 PE=1 SV=3	H3C15	0.711	0.01414388	Down						PF00125	Core histone H2A/H2B/H3/H4	B	KOG1745	Histones H3 and H4
Q7Z2Y8	Interferon-induced very large GTPase 1 OS=Homo sapiens OX=9606 GN=GVINP1 PE=2 SV=2	GVINP1	0.706	0.000299693	Down										
Q16557	Pregnancy-specific beta-1-glycoprotein 3 OS=Homo sapiens OX=9606 GN=PSG3 PE=2 SV=2	PSG3	0.704	0.029632543	Down	PSG, CD66f; pregnancy specific beta-1-glycoprotein		GO:0000003 reproduction; GO:0002376 immune system process; GO:0006928 movement of cell or subcellular component; GO:0006950 response to stress; GO:0006952 defense response; GO:0007155 cell adhesion; GO:0007156 homophilic cell adhesion via plasma	GO:0005576 extracellular region; GO:0005623 cell; GO:0005886 plasma membrane; GO:0005887 integral component of plasma membrane; GO:0009897 external side of plasma membrane; GO:0009986 cell surface; GO:0016020 membrane; GO:0016021 integral component of membrane; GO:00163	GO:0005488 binding; GO:0005515 protein binding; GO:0005543 phospholipid binding; GO:0008289 lipid binding; GO:0034235 GPI anchor binding; GO:0035091 phosphatidylinositol binding; GO:0042802 identical protein binding; GO:0042803 protein homodimerization activity;	PF07686; PF13927; PF13895	Immunoglobulin V-set domain; Immunoglobulin domain; Immunoglobulin domain			

Q9UL62	Short transient receptor potential channel 5 OS=Homo sapiens OX=9606 GN=TRPC5 PE=1 SV=1	TRPC5	0.702	0.001720148	Down	TRPC5; transient receptor potential cation channel subfamily C member 5	map04360 Axon guidance; map04929 GnRH secretion	GO:0000041 transition metal ion transport; GO:0001558 regulation of cell growth; GO:0001932 regulation of protein phosphorylation; GO:0001934 positive regulation of protein phosphorylation; GO:0006810 transport; GO:0006811 ion transport; GO:0006812 cation transport;	GO:0005622 intracellular; GO:0005623 cell; GO:0005737 cytoplasm; GO:0005886 plasma membrane; GO:0005887 integral component of plasma membrane; GO:0016020 membrane; GO:0016021 integral component of membrane; GO:0030424 axon; GO:0030425 dendrite; GO:00304	GO:0003779 actin binding; GO:0005215 transporter activity; GO:0005216 ion channel activity; GO:0005261 cation channel activity; GO:0005262 calcium channel activity; GO:0005488 binding; GO:0005515 protein binding; GO:0008092 cytoskeletal protein binding; GO:0008324 cation			P	KOG3609	Receptor-activated Ca2+-permeable cation channels (STRPC family)
Q9UDX3	SEC14-like protein 4 OS=Homo sapiens OX=9606 GN=SEC14L4 PE=1 SV=1	SEC14L4	0.698	0.003311787	Down						PF00650	CRAL/TRIO domain	I	KOG1471	Phosphatidylinositol transfer protein SEC14 and related proteins
Q8WVN8	Ubiquitin-conjugating enzyme E2 Q2 OS=Homo sapiens OX=9606 GN=UBE2Q2 PE=1 SV=1	UBE2Q2	0.697	0.000186125	Down	UBE2Q; ubiquitin-conjugating enzyme E2 Q [EC:2.3.2.23]	map04120 Ubiquitin mediated proteolysis	GO:0000209 protein polyubiquitination; GO:0006464 cellular protein modification process; GO:0006807 nitrogen compound metabolic process; GO:0008152 metabolic process; GO:0009987 cellular process; GO:0016567 protein ubiquitination; GO:0019538 protein metabolic process; GO:0032446 protein	GO:0005622 intracellular; GO:0005623 cell; GO:0005737 cytoplasm; GO:0005829 cytosol; GO:0044424 intracellular part; GO:0044444 cytoplasmic part; GO:0044464 cell part;	GO:0003824 catalytic activity; GO:0004842 ubiquitin-protein transferase activity; GO:0016740 transferase activity; GO:0019787 ubiquitin-like protein transferase activity;	PF00179	Ubiquitin-conjugating enzyme	O	KOG0897	Predicted ubiquitin-conjugating enzyme
O60244	Mediator of RNA polymerase II transcription subunit 14 OS=Homo sapiens OX=9606 GN=MED14 PE=1 SV=2	MED14	0.685	0.000538264	Down	MED14, RGR1; mediator of RNA polymerase II transcription subunit 14	map04919 Thyroid hormone signaling pathway	GO:0006139 nucleobase-containing compound metabolic process; GO:0006351 transcription, DNA-templated; GO:0006352 DNA-templated transcription, initiation; GO:0006355 regulation of transcription, DNA-templated; GO:0006357 regulation of transcription from	GO:0000428 DNA-directed RNA polymerase complex; GO:0005622 intracellular; GO:0005623 cell; GO:0005634 nucleus; GO:0005654 nucleoplasm; GO:0005667 transcription factor complex; GO:0016591 DNA-directed RNA polymerase II, holoenzyme;	GO:0003712 transcription cofactor activity; GO:0003713 transcription coactivator activity; GO:0005102 receptor binding; GO:0005488 binding; GO:0005515 protein binding; GO:0008134 transcription factor binding; GO:0030374 ligand-dependent nuclear receptor transcription			K	KOG1875	Thyroid hormone receptor-associated coactivator complex component (TRAP170)
P05121	Plasminogen activator inhibitor 1 OS=Homo sapiens OX=9606 GN=SERPINE1 PE=1 SV=1	SERPINE1	0.682	0.001678734	Down	SERPINE1, PAI1; plasminogen activator inhibitor 1	map04066 HIF-1 signaling pathway; map04115 p53 signaling pathway; map04218 Cellular senescence; map04371 Apelin signaling pathway; map04390 Hippo signaling pathway; map04610 Complement and coagulation cascades; map04933 AGE-RAGE signaling pathway in diabetic complications; map05142 Chagas disease (American trypanosomiasis)	GO:0000003 reproduction; GO:0000302 response to reactive oxygen species; GO:0001300 chronological cell aging; GO:0001525 angiogenesis; GO:0001568 blood vessel development; GO:0001666 response to hypoxia; GO:0001678 cellular glucose	GO:0005576 extracellular region; GO:0005615 extracellular space; GO:0005622 intracellular; GO:0005623 cell; GO:0005737 cytoplasm; GO:0005886 plasma membrane; GO:0012505 endomembrane system; GO:0016020 membrane; GO:0030141 secretory	GO:0002020 protease binding; GO:0004857 enzyme inhibitor activity; GO:0004866 endopeptidase inhibitor activity; GO:0004867 serine-type endopeptidase inhibitor activity; GO:0005102 receptor binding; GO:0005488 binding; GO:0005515 protein binding; GO:0019899 enzyme binding;	PF00079	Serpin (serine protease inhibitor)	V	KOG2392	Serpin

P83731	60S ribosomal protein L24 OS=Homo sapiens OX=9606 GN=RPL24 PE=1 SV=1	RPL24	0.678	5.58813E-05	Down	RP-L24e, RPL24; large subunit ribosomal protein L24e	map03010 Ribosome	GO:0000027 ribosomal large subunit assembly; GO:0000075 cell cycle checkpoint; GO:0000184 nuclear-transcribed mRNA catabolic process, nonsense-mediated decay; GO:0000278 mitotic cell cycle; GO:0000280 nuclear division; GO:0000902 cell morphogenesis; GO:0000904 cell	GO:0005622 intracellular; GO:0005623 cell; GO:0005737 cytoplasm; GO:0005783 endoplasmic reticulum; GO:0005829 cytosol; GO:0005840 ribosome; GO:0005844 polysome; GO:0012505 endomembrane system; GO:0015934 large ribosomal subunit;	GO:0003676 nucleic acid binding; GO:0003723 RNA binding; GO:0003735 structural constituent of ribosome; GO:0005198 structural molecule activity; GO:0005488 binding; GO:0097159 organic cyclic compound binding; GO:1901363 heterocyclic compound binding;	PF01246	Ribosomal protein L24e	J	KOG1722	60s ribosomal protein L24
Q9BRS8	La-related protein 6 OS=Homo sapiens OX=9606 GN=LARP6 PE=1 SV=1	LARP6	0.671	0.000885051	Down	LARP6; la-related protein 6		GO:0006417 regulation of translation; GO:0009889 regulation of biosynthetic process; GO:0009891 positive regulation of biosynthetic process; GO:0009893 positive regulation of metabolic process; GO:0010468 regulation of gene expression; GO:0010556 regulation	GO:0005622 intracellular; GO:0005623 cell; GO:0005634 nucleus; GO:0005737 cytoplasm; GO:0005844 polysome; GO:0032991 macromolecular complex; GO:0043226 organelle; GO:0043227 membrane-bounded organelle; GO:0043229 intracellular organelle;	GO:0003676 nucleic acid binding; GO:0003723 RNA binding; GO:0003729 mRNA binding; GO:0005488 binding; GO:0005515 protein binding; GO:0008092 cytoskeletal protein binding; GO:0017022 myosin binding; GO:0035613 RNA stem-loop binding; GO:0048027 mRNA 5'-UTR binding;	PF05383; PF12901	La domain; SUZ-C motif	A	KOG1855	Predicted RNA-binding protein
P04233	HLA class II histocompatibility antigen gamma chain OS=Homo sapiens OX=9606 GN=CD74 PE=1 SV=3	CD74	0.661	0.001672452	Down	CD74, DHLAG; CD74 antigen	map04612 Antigen processing and presentation; map05152 Tuberculosis; map05168 Herpes simplex virus 1 infection	GO:0000187 activation of MAPK activity; GO:0001516 prostaglandin biosynthetic process; GO:0001775 cell activation; GO:0001817 regulation of cytokine production; GO:0001819 positive regulation of cytokine production; GO:0001932 regulation of protein phosphorylation; GO:00019	GO:0000139 Golgi membrane; GO:0000323 lytic vacuole; GO:0005622 intracellular; GO:0005623 cell; GO:0005737 cytoplasm; GO:0005764 lysosome; GO:0005765 lysosomal membrane; GO:0005768 endosome; GO:0005770 late endosome; GO:0005771 multivesicu	GO:0001540 beta-amyloid binding; GO:0004888 transmembrane signaling receptor activity; GO:0004896 cytokine receptor activity; GO:0005102 receptor binding; GO:0005488 binding; GO:0005515 protein binding; GO:0019899 enzyme binding; GO:0019955 cytokine binding; GO:0023023 MHC	PF09307; PF08831; PF00086	CLIP, MHC2 interacting; Class II MHC-associated invariant chain trimerisation domain; Thyroglobulin type-1 repeat	U	KOG1214	Nidogen and related basement membrane protein proteins
Q96BT3	Centromere protein T OS=Homo sapiens OX=9606 GN=CENPT PE=1 SV=2	CENPT	0.64	0.012158142	Down	CENPT; centromere protein T		GO:0000278 mitotic cell cycle; GO:0006323 DNA packaging; GO:0006325 chromatin organization; GO:0006333 chromatin assembly or disassembly; GO:0006334 nucleosome assembly; GO:0006336 DNA replication-independent nucleosome assembly; GO:0006338	GO:0000228 nuclear chromosome; GO:0000775 chromosome, centromeric region; GO:0000776 kinetochore; GO:0000785 chromatin; GO:0000786 nucleosome; GO:0000788 nuclear nucleosome; GO:0000790 nuclear chromatin; GO:0005622 intracellula	GO:0000981 RNA polymerase II transcription factor activity, sequence-specific DNA binding; GO:0003676 nucleic acid binding; GO:0003677 DNA binding; GO:0003700 transcription factor activity, sequence-specific DNA binding; GO:0005488 binding; GO:0005515 protein					

Q01995	Transgelin OS=Homo sapiens OX=9606 GN=TAGLN PE=1 SV=4	TAGLN	0.622	0.000180841	Down	TAGLN; transgelin		GO:0007275 multicellular organism development; GO:0007517 muscle organ development; GO:0009888 tissue development; GO:0009987 cellular process; GO:0030154 cell differentiation; GO:0030855 epithelial cell differentiation; GO:0032501			PF00307	Calponin homology (CH) domain	Z	KOG2046	Calponin
P48594	Serpin B4 OS=Homo sapiens OX=9606 GN=SERPINB4 PE=1 SV=2	SERPINB4	0.563	0.006545233	Down	SERPINB; serpin B	map05146 Amoebiasis	GO:0001910 regulation of leukocyte mediated cytotoxicity; GO:0001911 negative regulation of leukocyte mediated cytotoxicity; GO:0002682 regulation of immune system process; GO:0002683 negative regulation of immune system process; GO:0002697 regulation	GO:0005576 extracellular region; GO:0005615 extracellular space; GO:0005622 intracellular; GO:0005623 cell; GO:0044421 extracellular region part; GO:0044464 cell part;	GO:0002020 protease binding; GO:0004857 enzyme inhibitor activity; GO:0004866 endopeptidase inhibitor activity; GO:0004867 serine-type endopeptidase inhibitor activity; GO:0005488 binding; GO:0005515 protein binding; GO:0019899 enzyme binding; GO:0030234 enzyme regulator	PF00079	Serpin (serine protease inhibitor)	V	KOG2392	Serpin
P16402	Histone H1.3 OS=Homo sapiens OX=9606 GN=H1-3 PE=1 SV=2	H1-3	0.549	0.006413376	Down	H1_5; histone H1/5			GO:0000228 nuclear chromosome; GO:0000785 chromatin; GO:0000790 nuclear chromatin; GO:0000791 euchromatin; GO:0000792 heterochromatin; GO:0005622 intracellular; GO:0005623 cell; GO:0005634 nucleus; GO:0005694 chromosome; GO:00057	GO:0003676 nucleic acid binding; GO:0003677 DNA binding; GO:0003682 chromatin binding; GO:0005488 binding; GO:0031490 chromatin DNA binding; GO:0097159 organic cyclic compound binding; GO:1901363 heterocyclic compound binding;	PF00538	linker histone H1 and H5 family	B	KOG4012	Histone H1
P16401	Histone H1.5 OS=Homo sapiens OX=9606 GN=H1-5 PE=1 SV=3	H1-5	0.549	2.47218E-05	Down	H1_5; histone H1/5		GO:0000122 negative regulation of transcription from RNA polymerase II promoter; GO:0001558 regulation of cell growth; GO:0006325 chromatin organization; GO:0006355 regulation of transcription, DNA-templated; GO:0006357 regulation of	GO:0000228 nuclear chromosome; GO:0000785 chromatin; GO:0000790 nuclear chromatin; GO:0000792 heterochromatin; GO:0005622 intracellular; GO:0005623 cell; GO:0005634 nucleus; GO:0005694 chromosome; GO:0005720 nuclear heterochromatin; GO:00319	GO:0003676 nucleic acid binding; GO:0003677 DNA binding; GO:0003682 chromatin binding; GO:0005488 binding; GO:0005515 protein binding; GO:0019899 enzyme binding; GO:0031490 chromatin DNA binding; GO:0042826 histone deacetylase binding; GO:0097159 organic cyclic	PF00538	linker histone H1 and H5 family	B	KOG4012	Histone H1

Q9BPU6	Dihydropyrimidinase -related protein 5 OS=Homo sapiens OX=9606 GN=DPYSL5 PE=1 SV=1	DPYSL5	0.545	2.43761E-05	Down	DPYSL5, CRAM; dihydropyrimidinase-like 5	map04360 Axon guidance	GO:0000902 cell morphogenesis; GO:0000904 cell morphogenesis involved in differentiation; GO:0006928 movement of cell or subcellular component; GO:0006935 chemotaxis; GO:0007154 cell communication; GO:0007165 signal transduction; GO:00072	GO:0005622 intracellular; GO:0005623 cell; GO:0005737 cytoplasm; GO:0005829 cytosol; GO:0030425 dendrite; GO:0032991 macromolecular complex; GO:0036477 somatodendritic compartment; GO:0042995 cell projection; GO:0043005 neuron projection;		PF01979	Amidohydrolase family	F	KOG2584	Dihydroorotase and related enzymes
P10412	Histone H1.4 OS=Homo sapiens OX=9606 GN=H1-4 PE=1 SV=2	H1-4	0.525	0.000440036	Down	H1_5; histone H1/5		GO:0000122 negative regulation of transcription from RNA polymerase II promoter; GO:0006323 DNA packaging; GO:0006325 chromatin organization; GO:0006333 chromatin assembly or disassembly; GO:0006355 regulation of transcription, DNA-	GO:0000228 nuclear chromosome; GO:0000785 chromatin; GO:0000790 nuclear chromatin; GO:0000791 euchromatin; GO:0005622 intracellular; GO:0005623 cell; GO:0005634 nucleus; GO:0005694 chromosome; GO:0005719 nuclear euchromatin; GO:00319	GO:0003676 nucleic acid binding; GO:0003677 DNA binding; GO:0003682 chromatin binding; GO:0005488 binding; GO:0031490 chromatin DNA binding; GO:0097159 organic cyclic compound binding; GO:1901363 heterocyclic compound binding;	PF00538	linker histone H1 and H5 family	B	KOG4012	Histone H1
P16403	Histone H1.2 OS=Homo sapiens OX=9606 GN=H1-2 PE=1 SV=2	H1-2	0.482	0.000207801	Down	H1_5; histone H1/5		GO:0000122 negative regulation of transcription from RNA polymerase II promoter; GO:0006323 DNA packaging; GO:0006325 chromatin organization; GO:0006333 chromatin assembly or disassembly; GO:0006355 regulation of transcription, DNA-	GO:0000228 nuclear chromosome; GO:0000785 chromatin; GO:0000790 nuclear chromatin; GO:0000791 euchromatin; GO:0005622 intracellular; GO:0005623 cell; GO:0005634 nucleus; GO:0005694 chromosome; GO:0005719 nuclear euchromatin; GO:00319	GO:0003676 nucleic acid binding; GO:0003677 DNA binding; GO:0003682 chromatin binding; GO:0005488 binding; GO:0031490 chromatin DNA binding; GO:0097159 organic cyclic compound binding; GO:1901363 heterocyclic compound binding;	PF00538	linker histone H1 and H5 family	B	KOG4012	Histone H1
P07305	Histone H1.0 OS=Homo sapiens OX=9606 GN=H1-0 PE=1 SV=3	H1-0	0.446	0.000112036	Down	H1_5; histone H1/5		GO:0000737 DNA catabolic process, endonucleolytic; GO:0006139 nucleobase-containing compound metabolic process; GO:0006259 DNA metabolic process; GO:0006308 DNA catabolic process; GO:0006309 apoptotic DNA fragmentation; GO:0006725 cellular aromatic compound	GO:0000228 nuclear chromosome; GO:0000785 chromatin; GO:0000790 nuclear chromatin; GO:0000791 euchromatin; GO:0005622 intracellular; GO:0005623 cell; GO:0005634 nucleus; GO:0005654 nucleoplasm; GO:0005694 chromosome; GO:00057	GO:0003676 nucleic acid binding; GO:0003677 DNA binding; GO:0003680 AT DNA binding; GO:0003682 chromatin binding; GO:0003690 double-stranded DNA binding; GO:0005488 binding; GO:0031490 chromatin DNA binding; GO:0043565 sequence-specific	PF00538	linker histone H1 and H5 family	B	KOG4012	Histone H1
			1.2												
			0.769230769												
